# Construction of 1,3‐Nonadjacent Stereogenic Centers Through Enantioselective Addition of α‐Thioacetamides to α‐Substituted Vinyl Sulfones Catalyzed by Chiral Strong Brønsted Base

**DOI:** 10.1002/advs.202308020

**Published:** 2023-12-21

**Authors:** Azusa Kondoh, Rihaku Ojima, Sho Ishikawa, Masahiro Terada

**Affiliations:** ^1^ Research and Analytical Center for Giant Molecules Graduate School of Science Tohoku University Aramaki, Aoba‐ku Sendai 980–8578 Japan; ^2^ Department of Chemistry Graduate School of Science Tohoku University Aramaki, Aoba‐ku Sendai 980–8578 Japan

**Keywords:** asymmetric synthesis, Brønsted base catalysis, enantioselective addition, nonadjacent stereogenic center, protonation

## Abstract

An enantioselective addition reaction for the construction of 1,3‐nonadjacent stereogenic centers is developed by means of a chiral strong Brønsted base catalyst. The chiral sodium ureate catalyst efficiently promoted the reaction of α‐thioacetamides as less acidic pronucleophiles with vinyl sulfones having a variety of α‐substituents including aryl, alkyl and halo groups, and α‐phenylacrylates, accomplishing the construction of various 1,3‐nonadjacent stereogenic centers in highly diastereo‐ and enantioselective manners. This is a rare example of the construction of 1,3‐nonadjacent stereogenic centers with less acidic pronucleophiles. In addition, the application of Michael acceptors having various types of α‐substituents in a single catalyst system is achieved for the first time, demonstrating the utility of the present catalyst system for the construction of 1,3‐nonadjacent stereogenic centers.

## Graphical abstract

An enantioselective addition reaction of α‐thioacetamides as less acidic pronucleophiles with vinyl sulfones having a variety of α‐substituents including aryl, alkyl, and halo groups, and α‐phenylacrylates is developed by means of the chiral sodium ureate as a chiral strong Brønsted base catalyst, accomplishing the construction of various 1,3‐nonadjacent stereogenic centers in highly diastereo‐ and enantioselective manners.

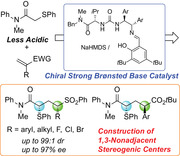

## 1 Introduction

**Scheme 1:**
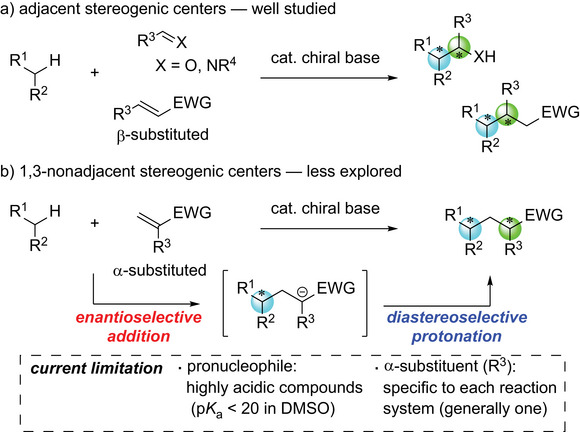
Construction of two stereogenic centers under chiral Brønsted base catalysis.

The enantioselective addition reaction catalyzed by a chiral Brønsted base is one of the most fundamental and efficient methodologies for the direct synthesis of enantio‐enriched compounds from readily available starting materials.^[^ ^1^ ^]^ When two stereogenic centers are constructed using this methodology, two adjacent centers generally result through the reactions of α‐di‐, or trisubstituted carbon pronucleophiles with electrophilic unsaturated compounds, such as aldehydes, ketones, imines, and β‐substituted Michael acceptors (**Scheme** **1**a). The two stereogenic centers are constructed in a single elementary process, i.e., the nucleophilic attack of an anionic nucleophile to an electrophile, forming a new carbon‐carbon bond in diastereo‐ and enantioselective manners. This methodology also allows for the construction of 1,3‐nonadjacent stereogenic centers, commonly found in various biologically active compounds by the addition reactions of α‐di‐ or trisubstituted carbon pronucleophiles with α‐substituted Michael acceptors (Scheme 1b). In this case, the two stereogenic centers are constructed in two separate elementary processes: the enantioselective addition of an anionic nucleophile to an electrophile, and the subsequent diastereoselective protonation of the anionic intermediate. Nonetheless, the reactions involving the construction of 1,3‐nonadjacent stereogenic centers in a highly stereoselective manner are still scarce, indicating the difficulty in controlling the stereoselectivities of both processes.^[^ ^6^, ^7^, ^8^, ^9^ ^]^ In addition, the reported reactions are limited to those of highly acidic pronucleophiles because of the inherent weak basicity of chiral organic bases, such as chiral tertiary amines^[^ ^2^ ^]^ and guanidines,^[^ ^3^ ^]^ which are commonly used as chiral Brønsted base catalysts in this type of enantioselective reaction. Thus, the construction of 1,3‐nonadjacent stereogenic centers with less acidic pronucleophiles, particularly those having p*K*_a_ values higher than 20 in DMSO (dimethyl sulfoxide), have never been achieved. Furthermore, the α‐substituents on Michael acceptors are highly specific to each reaction system. In most reports, one specific substituent is employed. As a seminal work, Kobayashi and co‐workers investigated the application of acrylates having different types of α‐substituents including alkyl, phenyl, and chloro groups under the catalysis of chiral calcium complexes.^[^ ^4^ ^]^ Even in this report, only the reaction with α‐phenyl acrylate provided the adduct with high diastereo‐ and enantioselectivities (dr (diastereomeric ratio) = 91:9, 84% ee). Therefore, the development of reactions of less acidic pronucleophiles with Michael acceptors having various types of α‐substituents is a challenging issue and the establishment of a new efficient catalyst system that overcomes the current limitation of both pronucleophiles and electrophiles is highly anticipated.

**Scheme 2:**
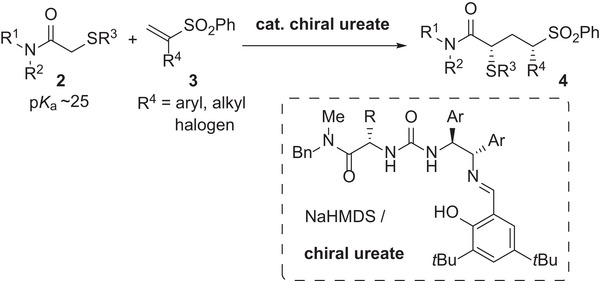
Enantioselective addition of α‐thioacetamides with vinyl sulfones having various α‐substituents.

We have been focusing on the development of chiral Brønsted base catalysts having much stronger basicity than those of conventional chiral catalysts to expand the scope of applicable pronucleophiles and develop new enantioselective transformations.^[^ ^13^, ^20^, ^21^ ^]^ We previously developed new chiral strong Brønsted base catalysts to promote a series of enantioselective addition reactions of less acidic pronucleophiles, which involves the construction of two adjacent stereogenic centers in highly stereoselective manners.^[^ ^8^ ^]^ After successfully constructing two adjacent stereogenic centers, we envisioned the construction of 1,3‐nonadjacent stereogenic centers through the unprecedented reactions of less acidic pronucleophiles with α‐substituted Michael acceptors. Herein, we report the enantioselective addition reaction of α‐thioacetamides 2 as less acidic pronucleophiles with vinyl sulfones 3 having various α‐substituents including aryl, alkyl, and halo groups (**Scheme** **2**). Chiral sodium ureate as a chiral strong Brønsted base catalyst^[^ ^8d^ ^]^ efficiently promoted the reaction, constructing the 1,3‐nonadjacent stereogenic centers in highly diastereo‐ and enantioselective manners.

## 2 Results and Discussion

We began our investigation by evaluating the addition reaction of α‐phenylthioacetamide **2a**, whose p*K*_a_ value in DMSO would be ≈25,^[^ ^9^ ^]^ as a primary pronucleophile with vinyl sulfone **3a** having a phenyl group at the α position as an electrophile (**Table** **1**). In preliminary experiments, **2a** was treated with **3a** in the presence of 10 mol.% achiral organic phosphazene bases having different basicities in toluene at 0 °C for 30 min. While the use of P1‐*t*Bu (p*K*_BH+_ = 15.7 in DMSO)^[^ ^10^ ^]^ resulted in full recovery of **2a**, the use of stronger organic bases P2‐*t*Bu (p*K*_BH+_ = 21.5 in DMSO) and P4‐*t*Bu (p*K*_BH+_ = 30.3 in DMSO) provided corresponding adduct **4aa** in 56% and 94% NMR yields, respectively, as a ≈1:1 diastereomeric mixture (entries 1–3). These results clearly suggest not only that the use of a catalyst having sufficient strong basicity is required to promote the reaction, but also that catalyst control is required to construct the stereogenic center α to the sulfonyl group in a diastereoselective manner. Next, the sodium ureate generated in situ by treatment of achiral *N,N’*‐dialkyl urea **1a** with an equivalent amount of NaHMDS (HMDS = hexamethyldisilazide) was tested (entry 4). In this case, **4aa** was obtained in quantitative yield with a much higher diastereomeric ratio (dr = 90:10) than that obtained when using the phosphazenes. Consequently, we investigated the enantioselective addition reaction of **2a** with **3a** using chiral sodium ureates as chiral strong Brønsted base catalysts. Chiral urea **1b** having an isopropyl group at the α position of an amide moiety and phenyl groups on the ethylene linker was applied as the precatalyst. The corresponding chiral ureate was generated in situ by treating **1b** with 2 equivalents of NaHMDS for the deprotonation of both urea and the more acidic phenol moieties. The reaction proceeded efficiently, and **4aa** was obtained in nearly quantitative yield. In addition, the diastereomeric ratio of **4aa** was high (dr = 93:7), and substantial 64% ee was observed for the major diastereomer (entry 5). A brief screening of solvents was then conducted (entries 6–8). The use of ethereal solvents, such as diethyl ether and THF, decreased the diastereoselectivity. On the other hand, only a small amount of the adduct was formed when using ethyl acetate. The choice of countercation of the chiral ureate was critical to the catalyst activity. When KHMDS was used instead of NaHMDS, the reaction proceeded, but **4aa** was obtained in racemic form with low diastereoselectivity (entry 9). The lithium ureate did not promote the reaction at all, and **2a** was fully recovered (entry 10). These results suggest that the countercation of the ureate is highly influential for catalytic activity and stereocontrol, which would attribute to its size and Lewis acidity.^[^ ^8d^ ^]^ The screening of substituents on the chiral ureas was then carried out (entries 11–13). Introduction of *tert*‐butyl and benzyl groups to the α position of an amide moiety was detrimental to the diastereoselectivities (entries 11 and 12). On the other hand, **1e** possessing 1‐naphthyl groups on the ethylene linker markedly improved the enantioselectivity (entry 13). Finally, the stereoselectivity was further improved by decreasing the reaction temperature to −20 °C, providing **4aa** in 89% yield with 99:1 dr and 94% ee (entry 14). The absolute configuration of the major diastereomer of **4aa** was determined to be (2*S*,4*S*) by single‐crystal X‐ray diffraction analysis of an analogous compound prepared by the enantioselective addition reaction followed by derivatization.^[^ ^11^ ^]^

**Table 1:** Screening of reaction conditions.

Entry	1^a)^	Base [mol.%]	Solvent	Yield [%]^b)^	Dr^c)^	Ee [%]^d)^
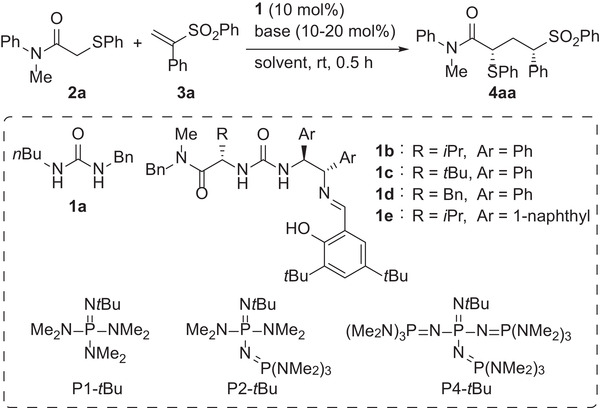						
1	‐	P1‐*t*Bu (10)	Toluene	(<1)	‐	‐
2	‐	P2‐*t*Bu (10)	Toluene	(56)	51:49	‐
3	‐	P4‐*t*Bu (10)	Toluene	(94)	58:42	‐
4	**1a**	NaHMDS (10)	Toluene	(>99)	90:10	‐
5	**1b**	NaHMDS (20)	Toluene	98	93:7	64
6	**1b**	NaHMDS (20)	Et_2_O	91	73:27	80
7	**1b**	NaHMDS (20)	THF	99	59:41	60
8	**1b**	NaHMDS (20)	AcOEt	(<5)	58:42	‐
9	**1b**	KHMDS (20)	Toluene	91	55:45	<1
10	**1b**	LiHMDS (20)	Toluene	<1	‐	‐
11	**1c**	NaHMDS (20)	Toluene	(99)	86:14	64
12	**1d**	NaHMDS (20)	Toluene	99	72:28	64
13	**1e**	NaHMDS (20)	Toluene	98	95:5	92
14^e)^	**1e**	NaHMDS (20)	Toluene	89	99:1	94

^a)^ Conditions: **2a** (0.10 mmol.), **3a** (0.12 mmol.), **1** (0.010 mmol.) with base (0.010–0.020 mmol.), or base (0.010 mmol.) without **1**, toluene (1.0 mL), 0 °C, 0.5 h

^b)^ Isolated yields. NMR yields are shown in parentheses

^c)^ Diastereomeric ratio of **4aa** was determined by ^1^H NMR analysis of the crude mixture

^d)^ Enantiomeric excess of **4aa** was determined by chiral stationary phase SFC analysis

^e)^ Performed at −20 °C.

**Scheme 3:**
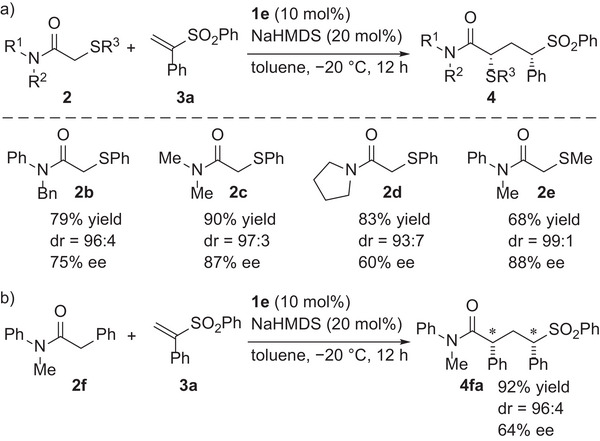
Scope of acetamide derivatives.

With the optimized reaction conditions in hand, the scopes of pronucleophiles and Michael acceptors was investigated. First, α‐thioacetamides having different substituents were examined (**Scheme** **3**a). In addition to *N*‐benzyl‐*N‐*phenyl α‐phenylthioacetamide (**2b**), *N*,*N*‐dialkyl variants **2c** and **2d** underwent the reaction smoothly. While the diastereoselectivity was high in all cases, the enantioselectivity was affected by the substituents on the nitrogen. α‐Methylthioacetamide **2e** was also applicable, and the corresponding **4ea** was obtained with high diastereo‐ and enantioselectivities. However, the reaction of α‐thioacetamides having an additional substituent at the α position, such as α‐thiopropionamide, did not proceed, and the construction of 1,3‐nonadjacent stereogenic centers containing a tetrasubstituted carbon failed. α‐Phenylacetamide **2f** was then applied as a pronucleophile (Scheme 3b). The reaction of **2f** with **3a** provided a promising result. **4fa** was obtained in a highly diastereoselective manner although with moderate ee.^[^ ^12^ ^]^ On the other hand, the use of α‐phenoxyacetamides and α‐fluoroacetamides as a pronucleophile resulted in the recovery of starting materials presumably because of the lower acidity of these pronucleophiles than that of α‐phenylthioacetamides.

**Scheme 4:**
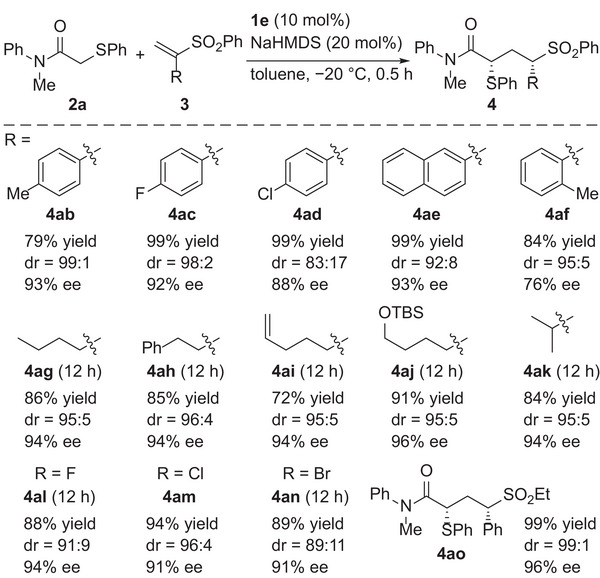
Scope of α‐substituted vinyl sulfones.

**Scheme 5:**
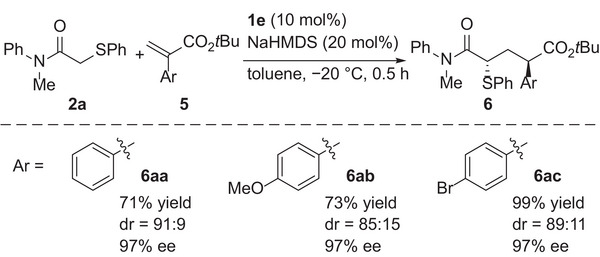
Reaction of *α*‐substituted acrylates.

Next, the scope of α‐substituted vinyl sulfones was investigated (**Scheme** **4**). First, various aryl groups were tested as the α‐substituent. Vinyl sulfones **3b** and **3c** having *para*‐tolyl and 4‐fluorophenyl groups provided corresponding adducts **4ab** and **4ac** in good yields with high diastereo‐ and enantioselectivities, respectively. The reaction of 4‐chlorophenyl‐substituted **3d** also provided the adduct in high yield with high enantioselectivity albeit with slightly lower diastereoselectivity. 2‐Naphthyl‐substituted **3e** underwent the reaction without any problem to provide **4ae** in high yield with high diastereo‐ and enantioselectivities. On the other hand, application of **3f** having an *ortho*‐tolyl group resulted in the formation of **4af** with moderate enantioselectivity. In addition to aryl groups, alkyl groups were applicable as α‐substituents. **3g**‐**3j** having a variety of primary alkyl groups underwent the reaction smoothly, and the corresponding adducts were obtained in high yields with high stereoselectivities. The reaction of **3k** having a secondary alkyl group also proceeded in a highly stereoselective manner. Vinyl sulfones **3l**‐**3n** containing a series of halo groups as an α‐substituent were then examined. In all cases, the reaction proceeded smoothly, and 1,3‐nonadjacent stereogenic centers, one of which is a α‐halo substituted one, were successfully constructed in a highly stereoselective manner.^[^ ^13^ ^]^ Ethyl vinyl sulfone derivative **3o** provided **4ao** in high yield with high diastereo‐ and enantioselectivities. Further investigation of α‐substituted Michael acceptors revealed that α‐aryl acrylates **5** were also suitable electrophiles in this catalyst system. The reaction of **2a** with acrylates **5a**‐**5c** provided **6aa**‐**6ac** in good yields with high diastereo‐ and enantioselectivities (**Scheme** **5**). It should be emphasized that catalyst systems that permit the use of Michael acceptors having such a wide range of α‐substituents and different electron‐withdrawing groups have never been reported. The absolute configuration of the major diastereomer of **6aa** was determined to be (2*R*,4*S*) by single‐crystal X‐ray diffraction analysis of its derivative **9aa** (vide infra), which means that the absolute configuration at the position α to the *tert*‐butoxycarbonyl group was opposite to that at the position α to the sulfonyl group of **4aa**.^[^ ^14^ ^]^

**Scheme 6:**
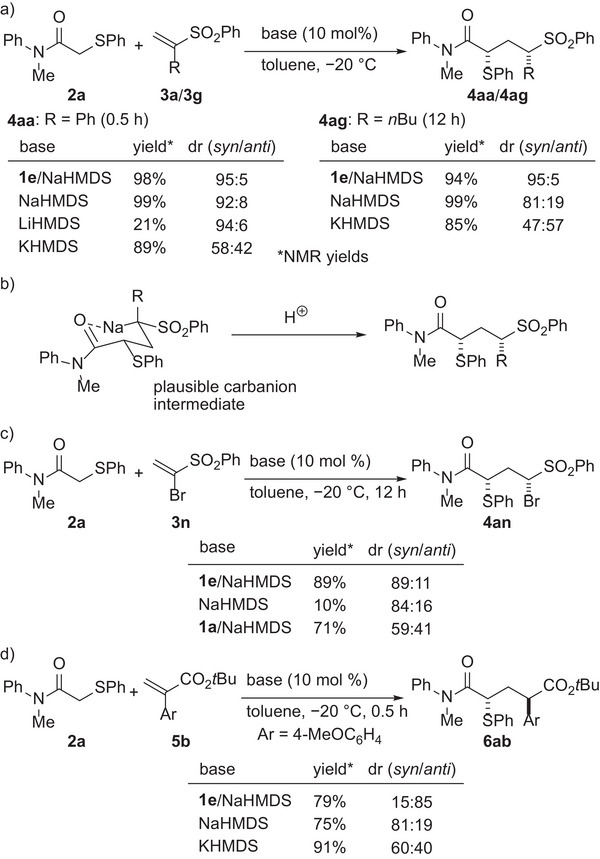
Control Experiments.

At this stage, control experiments were conducted to gain insight into the key factors for the diastereocontrol in the protonation process (**Scheme** **6**). The initial study of the reaction of **2a** with α‐phenyl‐substituted **3a** using achiral bases as well as chiral ureates indicated that the diastereoselectivity of the reaction was highly dependent on the choice of catalysts (Table 1). While achiral phosphazene bases and chiral potassium ureate provided **4aa** as a ≈1:1 diastereomeric mixture, both chiral and achiral sodium ureates provided **4aa** with good to high 1,3‐*syn* selectivity. Thus, we presumed that the choice of the countercation of the α‐sulfonyl carbanion intermediate generated through the addition of the amide enolate to the vinyl sulfone would be the key to achieving the high diastereoselectivity. Based on this hypothesis, the reaction of **2a** with **3a** was examined with a catalytic amount of hexamethyldisilazides (HMDS) having different alkaline metal cations (Scheme 6a). The use of NaHMDS resulted in the formation of **4aa** with the same level of diastereoselectivity as the sodium ureates. LiHMDS also provided **4aa** with high diastereoselectivity, albeit in low yield, while chiral lithium ureates did not promote the reaction (Table 1, entry 10). On the other hand, the use of KHMDS resulted in low diastereoselectivity, which is consistent with the result of potassium ureate (Table 1, entry 9). These results suggest that the sodium cation plays a key role in the diastereoselective protonation process as we expected. A similar trend was observed in the reaction of **2a** with α‐alkyl‐substituted **3g**. Chiral sodium ureate and NaHMDS provided **4ag** with good to high diastereoselectivities while the use of KHMDS resulted in low diastereoselectivity. A plausible conformation of the α‐sulfonyl carbanion intermediate is shown in Scheme 6b, where a sodium cation interacts not only with the anionic carbon center but also with the amide oxygen to form a chair‐like six‐membered cyclic structure. The formation of the cyclic structure is also supported by the fact that the diastereoselectivity was markedly reduced when using coordinating solvents, such as ethereal solvents and ethyl acetate (Table 1, entries 6–8). In contrast, the reaction of **2a** with α‐bromo substituted **3n** showed a different pattern of behavior (Scheme 6c). Whereas the use of NaHMDS as a catalyst resulted in very low yield of **4an** with good diastereoselectivity, achiral sodium ureate generated in situ from **1a** efficiently promoted the reaction but with low diastereoselectivity. Thus, in the reaction of **2a** with **3n**, the conjugate acid of the chiral Brønsted base catalyst, namely chiral urea, would predominantly control the stereoselectivity of the protonation process. In addition, the difference in the yields between NaHMDS and sodium ureates indicates that the urea facilitates catalyst turn‐over, presumably by serving as an effective proton source. The reaction of **2a** with α‐aryl acrylate **5b** was then evaluated with NaHMDS and KHMDS (Scheme 6d). Interestingly, the use of these achiral bases provided the 1,3‐*syn* adduct as the major diastereomer, while chiral sodium ureate afforded the 1,3‐*anti* adduct as the major diastereomer. These results suggest that the diastereoselectivity of the reaction with **5** is also controlled by the conjugate acid of the chiral Brønsted base catalyst to provide the corresponding 1,3‐*anti* adduct while suppressing the effect of the sodium cation that induces 1,3‐*syn* selectivity.^[^ ^15^ ^]^ Overall, the control experiments indicate that the key factor for the diastereocontrol in the protonation process is different depending on the α‐substituents and electron‐withdrawing groups of Michael acceptors. Nevertheless, the employment of the chiral sodium ureate as a chiral Brønsted base catalyst allowed the application of an unprecedented wide range of Michael acceptors, demonstrating the high potential of the present catalyst system for the construction of 1,3‐nonadjacent stereogenic centers.

**Scheme 7:**
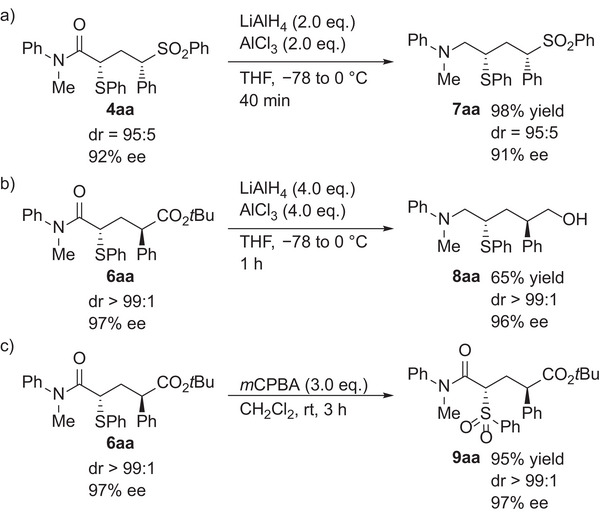
Derivatization of Adducts.

Finally, derivatization of the adducts was conducted (**Scheme** **7**). The reduction of **4aa** using LiAlH_4_ and AlCl_3_ provided corresponding amine **7aa** (Scheme 7a).^[^ ^16^ ^]^ The reduction of **6aa** under similar conditions afforded corresponding amino alcohol **8aa** (Scheme 7b). The phenythio group of **6aa** was convertible to a phenylsulfonyl group by treating with *m*CPBA (*meta*‐chloroperoxybenzoic acid) (Scheme 7c). Importantly, erosion of the diastereomeric ratio and enantiomeric excess did not occur in any case.

## 3 Conclusion

In conclusion, we have developed an enantioselective addition reaction for the construction of 1,3‐nonadjacent stereogenic centers by means of a chiral strong Brønsted base. The chiral sodium ureate catalyst efficiently promoted the reaction of α‐thioacetamides as less acidic pronucleophiles with vinyl sulfones having a variety of α‐substituents including aryl, alkyl, and halo groups, and α‐aryl acrylates, accomplishing the construction of various 1,3‐nonadjacent stereogenic centers in highly diastereo‐ and enantioselective manners. This is a rare example of the construction of 1,3‐nonadjacent stereogenic centers with less acidic pronucleophiles. In addition, the application of such a wide range of Michael acceptors in a single catalyst system was achieved for the first time. Further studies on the application of chiral ureate catalysts, including the development of new enantioselective reactions and mechanistic studies, are in progress

## 4 Experimental Section

### Typical Procedure for Enantioselective Addition Reactions of α‐Thioacetamides Catalyzed by Chiral Ureate

Reaction of **2a** with **3a** is representative (Table 1, entry 14). To a solution of **1e** (7.8 mg, 0.010 mmol.) in toluene (0.50 mL) was added a solution of NaHMDS in THF (1.0 m, 20 µL, 0.020 mmol.) at room temperature. The mixture was cooled to −20 °C and stirred for 10 min. Then **2a** (26 mg, 0.10 mmol.) was added at −20 °C. After stirring for 5 min, **3a** (29 mg, 0.12 mmol.) was added at −20 °C, and the reaction mixture was stirred for 30 min. The reaction was quenched with sat. aq. NH_4_Cl, and the product was extracted with AcOEt. The combined organic layer was washed with brine, dried over Na_2_SO_4_ and concentrated under reduced pressure. The residue was purified by silica gel column chromatography (hexane/AcOEt = 3:1 to 2:1) to afford **4aa** (45 mg, 0.089 mmol., 99% yield, 99:1 dr, and 94% ee) as a colorless sticky oil. CCDC 2272299, 2272322, 2291617, and 2272321 contain the supplementary crystallographic data for this paper. These data can be obtained free of charge from The Cambridge Crystallographic Data Center via www.ccdc.cam.ac.uk/data_request/cif.

## Conflict of Interest

The authors declare no conflict of interest.

## Supporting information

Supporting Information

## Data Availability

The data that support the findings of this study are available in the supplementary material of this article.
