# Supplementary material for: Construction of 1,3‐Nonadjacent Stereogenic Centers Through Enantioselective Addition of α‐Thioacetamides to α‐Substituted Vinyl Sulfones Catalyzed by Chiral Strong Brønsted Base
Source: Adv Sci (Weinh). 2023 Dec 21;11(9):2308020. doi: 10.1002/advs.202308020 (PMC10916638; doi:10.1002/advs.202308020)
Supplement: Supplementary file 1 — Supporting Information [file ADVS-11-2308020-s001.pdf]

ADVANCED SCIENCE
Open Access

Supporting Information

for *Adv. Sci.*, DOI 10.1002/advs.202308020

Construction of 1,3-Nonadjacent Stereogenic Centers Through Enantioselective Addition of α-Thioacetamides to α-Substituted Vinyl Sulfones Catalyzed by Chiral Strong Brønsted Base

Azusa Kondoh, Rihaku Ojima, Sho Ishikawa and Masahiro Terada**

Supporting Information

Construction of 1,3-Nonadjacent Stereogenic Centers through Enantioselective Addition of α-Thioacetamides to α-Substituted Vinyl Sulfones Catalyzed by Chiral Strong Brønsted Base

Azusa Kondoh,*[,a] Rihaku Ojima,[b] Sho Ishikawa[b] and Masahiro Terada*[,b]

[a]Research and Analytical Center for Giant Molecules, Graduate School of Science Tohoku University, Sendai 980-8578, Japan

[b]Department of Chemistry, Graduate School of Science, Tohoku University, Sendai 980-8578, Japan

Contents

1.	General Information	S2
2.	Experimental Procedure and Analytical Data	S3
3.	References	S24
4.	^{1}H NMR and ^{13}C NMR Spectra	S25
5.	HPLC Chart	S62
6.	X-ray Structure Reports	S91

1. General Information

Unless otherwise noted, the reactions were carried out with dried glassware under argon or nitrogen atmosphere. ^{1}H NMR spectra were recorded on a JEOL JNM-ECA600 (600 MHz) spectrometer. Chemical shifts are reported in ppm from the solvent resonance or tetramethylsilane (TMS) as the internal standard ($CDCl_3$: 7.26 ppm, TMS: 0.00 ppm). Data are reported as follows: chemical shift, integration, multiplicity (s = singlet, d = doublet, t = triplet, q = quartet, m = multiplet, br = broad) and coupling constants (Hz). ^{13}C NMR spectra were recorded on a JEOL JNM-ECA600 (150 MHz) spectrometer with complete proton decoupling. Chemical shifts are reported in ppm from the solvent resonance as the internal standard ($CDCl_3$: 77.0 ppm). ^{19}F NMR spectra were recorded on a JEOL JNM-ECA600 (565 MHz) spectrometer. Chemical shifts are reported in ppm from the $C_6F_5CF_3$ (−67.2 ppm) resonance as the external standard. Analytical thin layer chromatography (TLC) was performed on Merck precoated TLC plates (silica gel 60 GF_{254}, 0.25 mm). Flash column chromatography was performed on silica gel 60N (spherical, neutral, 40-50 μm; Kanto Chemical Co., Inc.). Optically rotations were measured on a Jasco P-1020 digital polarimeter with a sodium lamp and reported as follows; $[\alpha]^{T°C}{}_{D}$ (c = g/100 mL, solvent). HPLC was performed on JASCO HPLC systems consisting of the following: pump, PU-2080 plus; degasser, DG-2080-53; mixer, MX-2080-32; UV/Vis detector, UV2077 plus; CD detector, CD-2095; Oven, CO-2067 plus. SFC was performed on JASCO SFC systems consisting of the following: HPLC pump, PU-2080 plus; CO_2 delivery pump, PU-2080-CO_2 plus; solvent selection unit, LV-2080-03; Back Pressure Regulators, BP-2080 and BP-2080 plus; Photodiode detector, MD-2018 plus; Oven, CO-4065. Infrared spectra were recorded on a JASCO FT/IR-4100 spectrometer. High resolution mass spectra analysis was performed on a Bruker Daltonics solariX 9.4T FT-ICR-MS spectrometer at Research and Analytical Center for Giant Molecules, Graduate School of Science, Tohoku University.

Materials: Unless otherwise noted, materials were purchased from Wako Pure Chemical Industries, Ltd., Tokyo Chemical Industry Co., LTD., Aldrich Inc., and other commercial suppliers and were used without purification. Dichloromethane, tetrahydrofuran, diethyl ether and toluene were supplied from Kanto Chemical Co., Inc. as “Dehydrated solvent system”. Other solvents were purchased from commercial suppliers as dehydrated solvents, and used under argon atmosphere.

2. Experimental Procedure and Analytical Data

2-1. Precatalyst Synthesis

Chiral ureas **1** were prepared according to our previous report.[S1]

2-2. Substrate Synthesis

α-Thioacetamides **2a**, **2c**, **2d**, **2e** and **2g** were prepared according to our previous report.[S1]

Preparation of 2b

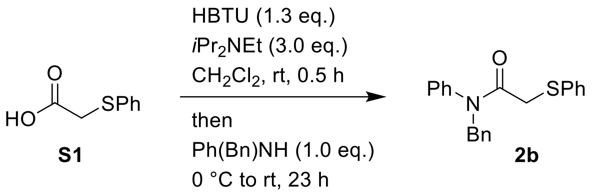

To a solution of (phenylthio)acetic acid (**S1**, 0.51 g, 3.0 mmol) and *i*Pr_2NEt (1.6 mL, 9.0 mmol) in CH_2Cl_2 (10 mL) was added HBTU (1.5 g, 3.9 mmol) at 0 °C. After stirring at 0 °C for 0.5 h, a solution of *N*-benzylaniline (0.55 g, 3.0 mmol) in CH_2Cl_2 (8.0 mL) was added dropwise to the mixture. The resulting mixture was stirred at 0 °C for 1 h, then warmed to room temperature, and additionally stirred for 22 h. The reaction was quenched with sat. aq. NH_4Cl, and the product was extracted with CH_2Cl_2. The combined organic layer was washed with sat. aq. $NaHCO_3$ and brine, dried over Na_2SO_4 and concentrated under reduced pressure. The crude mixture was purified by silica gel column chromatography (hexane/AcOEt = 4:1) to afford **2b** (0.65 g, 2.4 mmol, 80%) as a brown oil.

***N*-Benzyl-*N*-phenyl-2-(phenylthio)acetamide (2b):**

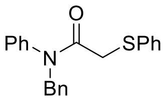

Brown oil; ^{1}H NMR (600 MHz, $CDCl_3$) δ 7.34 (d, J = 7.2 Hz, 2H), 7.32-7.28 (m, 3H), 7.27-7.22 (m, 5H), 7.21-7.18 (m, 1H), 7.17-7.14 (m, 2H), 6.94-6.90 (m, 2H), 4.88 (s, 2H), 3.51 (s, 2H); ^{13}C NMR (150 MHz, $CDCl_3$) δ 168.4, 141.5, 136.9, 135.3, 130.2, 129.5, 128.8 (2C), 128.31, 128.25, 128.2, 127.4, 126.7, 53.3, 37.4; IR (ATR): 3060, 3031, 1656, 1595, 1495, 1481, 1438, 1410, 1391, 699 cm^{-1}; HRMS (FD+) Calcd for $C_{21}H_{19}NOS$ $[M]^+$ 333.1187, Found 333.1186.

2-(4-Bromophenylthio)-1-morpholinoethan-1-one (2g):

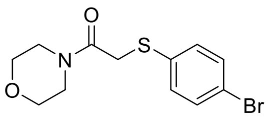

White solid; ^{1}H NMR (600 MHz, $CDCl_3$) δ 7.43 (d, J = 9.0 Hz, 2H), 7.32 (d, J = 9.0 Hz, 2H), 3.72 (s, 2H), 3.71-3.65 (m, 4H), 3.62-3.59 (m, 2H), 3.51-3.48 (m, 2H); ^{13}C NMR (150 MHz, $CDCl_3$) δ 166.8, 133.8, 132.2, 131.8, 121.2, 66.8, 66.5, 46.8, 42.3, 36.2; IR (ATR): 2962, 2922, 2855, 1642, 1474, 1436,1274, 1114, 1092, 1007 cm^{-1}; HRMS (FD+) Calcd for $C_{12}H_{14}BrNO_2S$ $[M]^+$ 314.9929, Found 314.9928; mp. 104.0-106.0 ºC.

Preparation of 3a, 3c and 3f

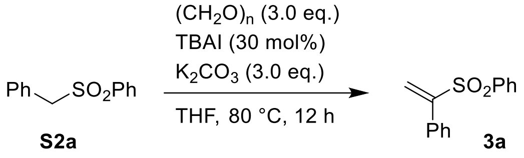

Synthesis of **3a** is representative.

To a solution of benzyl phenyl sulfone (**S2a**, 0.93 g, 4.0 mmol) in THF (4.0 mL) were added paraformaldehyde (0.36 g, 12.0 mmol), tetrabutylammonium iodide (TBAI, 0.44 g, 1.2 mmol), and K_2CO_3 (1.7 g, 12.0 mmol) at room temperature. The reaction mixture was then heated at 80 °C for 12 h. After cooled to room temperature, the reaction was quenched with H_2O, and the product was extracted with AcOEt. The combined organic layer was washed with brine, dried over Na_2SO_4 and concentrated under reduced pressure. The crude mixture was purified by silica gel column chromatography (hexane/AcOEt = 10:1) to afford **3a** (0.74 g, 3.0 mmol, 76%) as a white powder.

^{1}H NMR data of **3a**, **3c** and **3f** were matched with those reported in literature.[S2]

Preparation of 3b, 3d and 3e

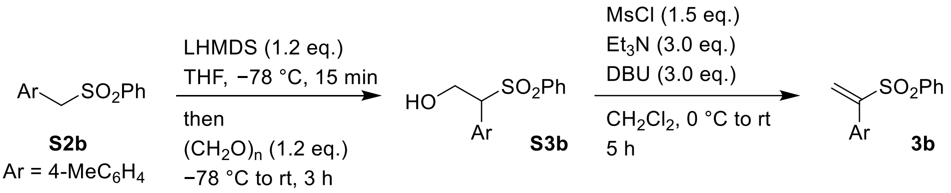

Synthesis of **3b** is representative.

To a solution of 4-methylbenzyl phenyl sulfone (**S2b**, 0.43 g, 1.8 mmol) in THF (15 mL) was added LHMDS in THF (1.0 M, 2.1 mL, 2.1 mmol) dropwise at −78 °C, and the solution was stirred at that temperature for 15 min. Paraformaldehyde (63 mg, 2.1 mmol) was then added, and the resulting mixture was warmed to room temperature and stirred for 3 h. The reaction was quenched with sat. aq. NH_4Cl, and the product was extracted with AcOEt. The combined organic layer was washed with brine, dried over Na_2SO_4 and concentrated under reduced pressure. The crude mixture was purified by silica gel column chromatography (hexane/AcOEt = 1:1) to afford **S3b** (0.28 g, 1.0 mmol, 57%) as a white powder.

To a solution of **S3b** (0.28 g, 1.0 mmol), triethylamine (0.42 mL, 3.0 mmol) and DBU (0.45 mL, 3.0 mmol) in CH_2Cl_2 (4.0 mL) was added methanesulfonyl chloride (0.12 mL, 1.5 mmol) at 0 °C, and the resulting mixture was stirred at room temperature for 5 h. The reaction was quenched with sat. aq. NH_4Cl, and the product was extracted with CH_2Cl_2. The combined organic layer was washed with sat. aq. $NaHCO_3$ and brine, dried over Na_2SO_4 and concentrated under reduced pressure. The crude mixture was purified by silica gel column chromatography (hexane/AcOEt = 10:1) to afford **3b** (0.23 g, 0.91 mmol, 91%) as a yellow powder.

^{1}H NMR data of **3b**, **3d** and **3e** were matched with those reported in literature.[S2]

Preparation of 3g-3k

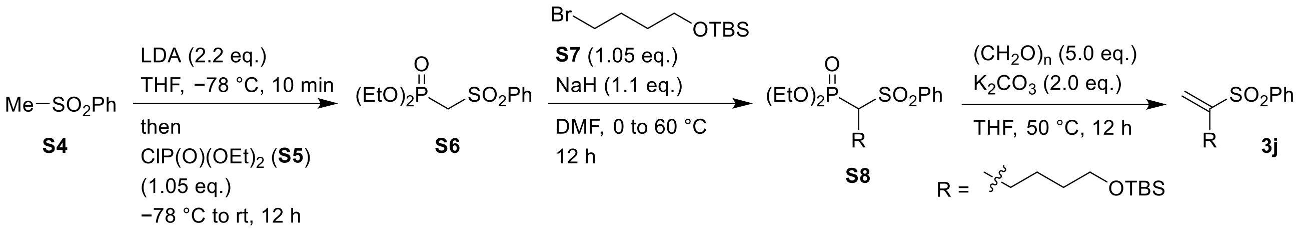

Synthesis of **3j** is representative.

*Synthesis of **S6***

To a solution of diisopropylamine (1.6 mL, 11.0 mmol) in THF (10 mL) was added *n*BuLi in hexane (1.6 M, 6.9 mL, 11.0 mmol) dropwise at −78 °C, and the solution was stirred at that temperature for 15 min. A solution of methyl phenyl sulfone (**S4**, 0.78 g, 5.0 mmol) in THF (5.0 mL) was then added dropwise at −78 °C. After stirred for 10 min, a solution of diethyl chlorophosphate (**S5**, 0.76 mL, 5.3 mmol) in THF (5.0 mL) was added dropwise at −78 °C. The resulting mixture was warmed to room temperature and stirred for 12 h. The reaction was quenched with sat. aq. NH_4Cl, and the product was extracted with AcOEt. The combined organic layer was washed with brine, dried over Na_2SO_4 and concentrated under reduced pressure. The residue was purified by silica gel column chromatography (hexane/AcOEt = 1:2 to 0:1) to afford **S6** (1.4 g, 4.7 mmol, 94%) as a colorless oil.
^{1}H NMR data of **S6** were matched with those reported in literature.[S3]

*Synthesis of **3j***

To a solution of **S6** (0.29 g, 1.0 mmol) in DMF (3.0 mL) was added NaH (44 mg, 1.1 mmol) at 0 °C, and the mixture was stirred for 15 min at that temperature. A solution of **S7** (0.28 g, 1.1 mmol) in DMF (0.50 mL) was then added to the mixture at 0 °C. The resulting mixture was heated at 60 °C and stirred for 12 h. After cooled to room temperature, the reaction was quenched with H_2O, and the product was extracted with AcOEt. The combined organic layer was washed with brine, dried over Na_2SO_4 and concentrated under reduced pressure. The residue was purified by silica gel column chromatography (hexane/AcOEt = 1:1) to afford **S8** (0.22 g, 0.46 mmol, 46%) as a colorless oil.
To a solution of **S8** (0.21 g, 0.44 mmol) in THF (1.0 mL) were added paraformaldehyde (66 mg, 2.2 mmol) and K_2CO_3 (0.18 g, 1.3 mmol) was added Cs_2CO_3 (0.42 g, 1.3 mmol) at room temperature. After stirring at 50 °C for 12 h, the reaction was quenched with H_2O, and the product was extracted with AcOEt. The combined organic layer was washed with brine, dried over Na_2SO_4 and concentrated under reduced pressure. The residue was purified by silica gel column chromatography (hexane/AcOEt = 10:1) to afford **3j** (0.12 g, 0.35 mmol, 80%) as a colorless oil.

^{1}H NMR data of **3g** and **3h** were matched with those reported in literature.[S4,S5]

1,6-Heptadien-2-yl Phenyl Sulfone (3i):

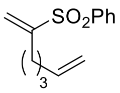

Colorless oil; ^{1}H NMR (600 MHz, $CDCl_3$) δ 7.88 (dd, J = 7.8, 1.2 Hz, 2H), 7.63 (tt, J = 7.8, 1.2 Hz, 1H), 7.55 (dd, J = 7.8, 7.8 Hz, 2H), 6.39 (d, J = 1.2 Hz, 1H), 5.74 (td, J = 1.8, 1.2 Hz, 1H), 5.72-5.65 (m, 1H), 4.96-4.92 (m, 2H), 2.26-2.23 (m, 2H), 2.03-1.98 (m, 2H), 1.59-1.53 (m, 2H); ^{13}C NMR (150 MHz, $CDCl_3$) δ 150.4, 138.9, 137.5, 133.4, 129.2, 128.2, 123.2, 115.4, 32.7, 28.5, 26.7; IR (ATR): 2978, 2934,

2865, 1446, 1304, 1170, 1137, 1082, 747, 689 cm^{-1}; HRMS (FD+) Calcd for $C_{13}H_{16}O_2S$ $[M]^+$ 236.0871, Found 236.0870.

6-(*tert*-Butyldimethylsiloxy)-1-hexen-2-yl Phenyl Sulfone (3j):

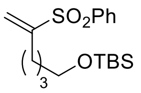

Colorless oil; ^{1}H NMR (600 MHz, $CDCl_3$) δ 7.88 (d, J = 7.2 Hz, 2H), 7.63 (t, J = 7.2 Hz, 1H), 7.54 (dd, J = 7.2, 7.2 Hz, 2H), 6.39 (s, 1H), 5.75 (s, 1H), 3.54 (t, J = 6.0 Hz, 2H), 2.25 (t, J = 7.2 Hz, 2H), 1.55-1.49 (m, 2H), 1.49-1.42 (m, 2H), 0.86 (s, 9H), 0.01 (s, 6H); ^{13}C NMR (150 MHz, $CDCl_3$) δ 150.5, 138.9, 133.4, 129.1, 128.2, 123.0, 62.4, 31.8, 28.8, 25.9, 23.8, 18.3, −5.4; IR (ATR): 2938, 2863, 1447, 1305, 1135, 1082, 796, 749, 689 cm^{-1}; HRMS (FD+) Calcd for $C_{18}H_{30}O_3SSi$ $[M]^+$ 355.1763, Found 355.1763.

3-Methyl-1-buten-2-yl Phenyl Sulfone (3k):

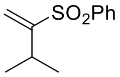

Colorless oil; ^{1}H NMR (600 MHz, $CDCl_3$) δ 7.88 (d, J = 7.8 Hz, 2H), 7.62 (t, J = 7.8 Hz, 1H), 7.54 (dd, J = 7.8, 7.8 Hz, 2H), 6.40 (d, J = 0.6 Hz, 1H), 5.83 (d, J = 0.6 Hz, 1H), 2.64 (sept, J = 6.6 Hz, 1H), 1.04 (d, J = 6.6 Hz, 6H); ^{13}C NMR (150 MHz, $CDCl_3$) δ 157.1, 139.3, 133.3, 129.1, 128.1, 122.0, 28.5, 23.1; IR (ATR): 2970, 2933, 1447, 1304, 1192, 1154, 1126, 1080, 747, 690 cm^{-1}; HRMS (FD+) Calcd for $C_{11}H_{14}O_2S$ $[M]^+$ 210.0715, Found 210.0709.

Preparation of 3l

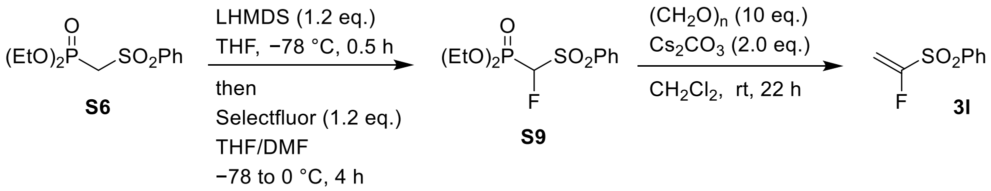

To a solution of **S6** (0.29 g, 1.0 mmol) in THF (3.0 mL) was added LHMDS in THF (1.0 M, 1.2 mL, 1.2 mmol) dropwise at −78 °C, and the mixture was stirred for 30 min at that temperature. A solution of Selectfluor (0.43 g, 1.2 mmol) in DMF (2.0 mL) was then added dropwise to the mixture at −78 °C. The resulting mixture was warmed to 0 °C and stirred for 4 h. The reaction was quenched with sat. aq. NH_4Cl, and the product was extracted with AcOEt. The combined organic layer was washed with brine, dried over Na_2SO_4 and concentrated under reduced pressure. The residue was purified by silica gel column chromatography (hexane/AcOEt = 1:1) to afford **S9** (0.20 g, 0.64 mmol, 64%) as a colorless oil.

To a solution of **S9** (0.20 g, 0.64 mmol) and paraformaldehyde (0.19 g, 6.4 mmol) in CH_2Cl_2 (6.0 mL) was added Cs_2CO_3 (0.42 g, 1.3 mmol) at room temperature. After stirring for 22 h, the reaction was quenched with H_2O, and the product was extracted with CH_2Cl_2. The combined organic layer was washed with brine, dried over Na_2SO_4 and concentrated under reduced pressure. The residue was purified by silica gel column chromatography (hexane/AcOEt = 10:1) to afford **3l** (0.11 g, 0.57 mmol, 90%) as a colorless oil.

^{1}H NMR data of **3l** were identical to those reported in the literature.[S6]

Preparation of 3m

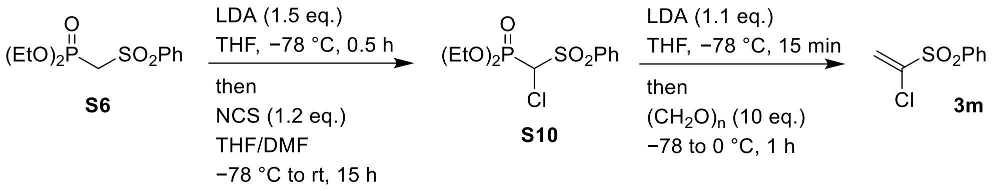

To a solution of diisopropylamine (0.50 mL, 3.5 mmol) in THF (8.0 mL) was added *n*BuLi in hexane (1.6 M, 2.2 mL, 3.5 mmol) dropwise at −78 °C, and the mixture was stirred for 15 min at that temperature. A solution of **S6** (0.69 g, 2.4 mmol) in THF (4.0 mL) was then added dropwise at −78 °C. After stirring for 30 min, *N*-chlorosuccinimide (NCS, 0.32 g, 2.8 mmol) was then added portionwise to the mixture at −78 °C. The resulting mixture was warmed to room temperature and stirred for 19 h. The reaction was quenched with sat. aq. NH_4Cl, and the product was extracted with AcOEt. The combined organic layer was washed with brine, dried over Na_2SO_4 and concentrated under reduced pressure. The residue was purified by silica gel column chromatography (hexane/AcOEt = 2:1 to 1:1) to afford **S10** (0.51 g, 1.6 mmol, 66%) as a brown oil.
To a solution of diisopropylamine (0.22 mL, 1.6 mmol) in THF (4.0 mL) was added *n*BuLi in hexane (1.6 M, 0.89 mL, 1.4 mmol) dropwise at −78 °C, and the mixture was stirred for 15 min at that temperature. A solution of **S10** (0.43 g, 1.3 mmol) in THF (4.0 mL) was then added dropwise at −78 °C. After stirred for 10 min, paraformaldehyde (0.39 g, 13 mmol) was added portionwise at that temperature. The resulting mixture was warmed to 0 °C and additionally stirred for 1 h. The reaction was quenched with sat. aq. NH_4Cl, and the product was extracted with AcOEt. The combined organic layer was washed with brine, dried over Na_2SO_4 and concentrated under reduced pressure. The residue was purified by silica gel column chromatography (hexane/AcOEt = 5:1) to afford **3m** (0.16 g, 0.80 mmol, 62%) as a colorless oil.

1-Chlorovinyl Phenyl Sulfone (3m):

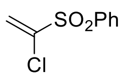

Colorless oil; ^{1}H NMR (600 MHz, $CDCl_3$) δ 7.97 (dd, *J* = 7.8, 1.2 Hz, 2H), 7.70 (tt, *J* = 7.8, 1.2 Hz, 1H), 7.59 (dd, *J* = 7.8, 7.8 Hz, 2H), 6.74 (d, *J* = 2.4 Hz, 1H), 6.08 (d, *J* = 2.4 Hz, 1H); ^{13}C NMR (150 MHz, $CDCl_3$) δ 140.2, 136.3, 134.4, 129.2, 129.0, 124.0; IR (ATR): 3415, 3117, 3066, 1738, 1448, 1326, 1311, 1189, 1160, 1074 cm^{-1}; HRMS (ESI) Calcd for $C_6H_7ClO_2S$ $[M+Na]^+$ 224.9748, Found 224.9748.

Preparation of 3n

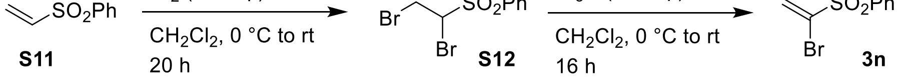

To a solution of phenyl vinyl sulfone (**S11**, 0.34 g, 2.0 mmol), in CH_2Cl_2 (3.0 mL) was added bromine (0.15 g, 3.0 mmol) at 0 °C. The resulting mixture was stirred at room temperature for 20 h. The volatile materials were removed under reduced pressure, and the crude mixture containing **S12** was then dissolved to CH_2Cl_2 (10 mL). Triethylamine (0.28 mL, 2.4 mmol) was added to the solution at 0 °C. The resulting mixture was stirred at room temperature for 16 h. The reaction was quenched with H_2O, and the product was extracted with CH_2Cl_2. The

combined organic layer was washed with sat. aq. $NaHCO_3$ and brine, dried over Na_2SO_4 and concentrated under reduced pressure. The crude mixture was purified by silica gel column chromatography (hexane/AcOEt = 5:1) to afford **3n** (0.48 g, 2.0 mmol, 97%) as a white powder.

^{1}H NMR data of **3n** were matched with those reported in literature.[S7]

Preparation of 3o[21]

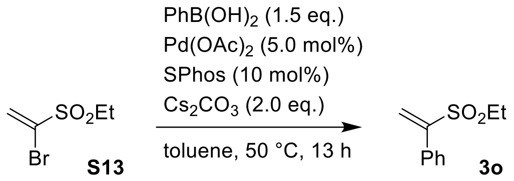

A mixture of **S13** (0.30 g, 1.5 mmol), phenylboronic acid (0.27 g, 2.3 mmol), $Pd(OAc)_2$ (17 mg, 0.075 mmol), SPhos (62 mg, 0.15 mmol) and Cs_2CO_3 (0.98 g, 3.0 mmol) in toluene (10 mL) was stirred at 50 °C for 13 h. The reaction was quenched with sat. aq. NH_4Cl, and the product was extracted with AcOEt. The combined organic layer was washed with brine, dried over Na_2SO_4 and concentrated under reduced pressure. The crude mixture was purified by silica gel column chromatography (hexane/AcOEt = 5:1) to afford **3o** (0.27 g, 1.4 mmol, 90%) as a yellow oil.

^{1}H NMR data of **3o** were matched with those reported in literature.[S8]

Preparation of 5a[22]

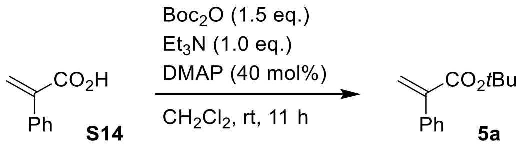

To a solution of 2-phenylacrylic acid (**S14**, 0.44 g, 3.0 mmol), Boc_2O (0.98 g, 4.5 mmol) in CH_2Cl_2 (6.0 mL) were added triethylamine (0.42 mL, 3.0 mmol) and DMAP (0.15 g, 1.2 mmol). The resulting mixture was stirred at room temperature for 11 h. The reaction was quenched with H_2O, and the product was extracted with CH_2Cl_2. The combined organic layer was washed with brine, dried over Na_2SO_4 and concentrated under reduced pressure. The crude mixture was purified by silica gel column chromatography (hexane/AcOEt = 20:1) to afford **5a** (0.52 g, 2.5 mmol, 83%) as a colorless oil.

^{1}H NMR data of **5a** were matched with those reported in literature.[S9]

Preparation of 5b and 5c

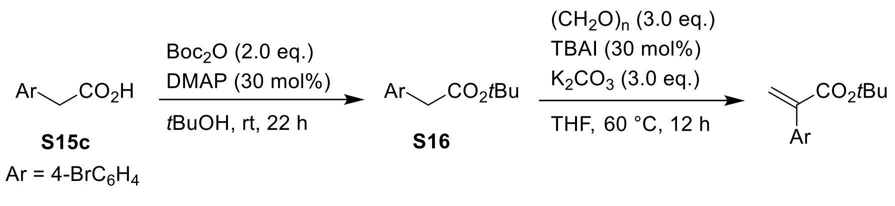

Synthesis of **5c** is representative.

To a solution of **S15c** (0.65 g, 3.0 mmol) in *t*BuOH (10 mL) were added Boc_2O (1.3 g, 6.0 mmol) and DMAP (0.11 g, 0.90 mmol). The resulting mixture was stirred at room temperature for 22 h. The reaction was quenched with H_2O, and the product was extracted with AcOEt. The combined organic layer was washed with brine, dried over Na_2SO_4 and concentrated under reduced pressure. The crude mixture was purified by silica gel column chromatography (hexane/AcOEt = 10:1) to afford **S16** (0.66 g, 2.4 mmol, 81%) as a yellow oil.

To a solution of **S16** (0.16 g, 0.60 mmol) in THF (3.0 mL) were added paraformaldehyde (54 mg, 1.8 mmol), tetrabutylammonium iodide (TBAI, 67 mg, 0.18 mmol), and K_2CO_3 (0.25 g, 1.8 mmol) at room temperature. The reaction mixture was then heated at 70 °C for 12 h. After cooled to room temperature, the reaction was quenched with H_2O, and the product was extracted with AcOEt. The combined organic layer was washed with brine, dried over Na_2SO_4 and concentrated under reduced pressure. The crude mixture was purified by silica gel column chromatography (hexane/AcOEt = 20:1) to afford **5c** (0.11 g, 0.38 mmol, 63%) as a colorless oil.

^{1}H NMR data of **5b** were matched with those reported in literature.[S9]

***tert*-Butyl 2-(4-Bromophenyl)acrylate (5c):**

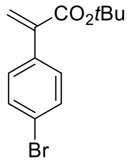

Colorless oil; ^{1}H NMR (600 MHz, $CDCl_3$) δ 7.47 (d, J = 9.0 Hz, 2H), 7.28 (d, J = 9.0 Hz, 2H), 6.27 (d, J = 1.2 Hz, 1H), 5.82 (d, J = 1.2 Hz, 1H), 1.52 (s, 9H); ^{13}C NMR (150 MHz, $CDCl_3$) δ 165.5, 141.8, 136.0, 131.1, 130.0, 126.2, 122.1, 81.5, 28.1; IR (ATR): 2978, 2932, 1713, 1488, 1368, 1333, 1157, 1086, 1011, 831 cm^{-1}; HRMS (FD+) Calcd for $C_{13}H_{15}BrO_2$ $[M]^+$ 282.0255, Found 282.0255.

2-3. Typical Procedure for Enantioselective Addition Reactions of α-Thioacetamides Catalyzed by Chiral Ureate 1

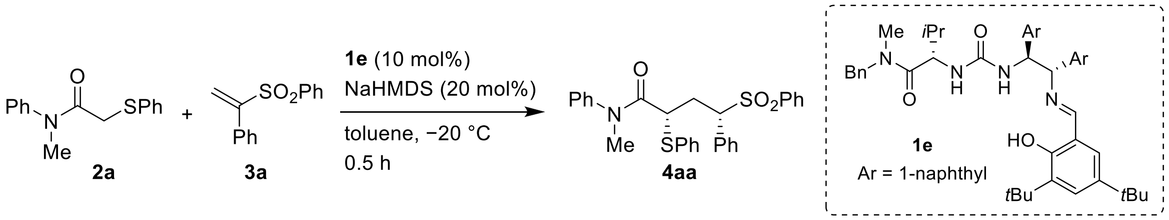

Reaction of **2a** with **3a** is representative (Table 1, entry 14).

To a solution of **1e** (7.8 mg, 0.010 mmol) in toluene (0.50 mL) was added a solution of NaHMDS in THF (1.0 M, 20 µL, 0.020 mmol) at room temperature. The mixture was cooled to −20 °C and stirred for 10 min. Then **2a** (26 mg, 0.10 mmol) was added at −20 °C. After stirring for 5 min, **3a** (29 mg, 0.12 mmol) was added at −20 °C, and the reaction mixture was stirred for 30 min. The reaction was quenched with sat. aq. NH_4Cl, and the product was extracted with AcOEt. The combined organic layer was washed with brine, dried over Na_2SO_4 and concentrated under reduced pressure. The residue was purified by silica gel column chromatography (hexane/AcOEt = 3:1 to 2:1) to afford **4aa** (45 mg, 0.089 mmol, 99% yield, 99:1 dr, 94% ee) as a colorless sticky oil.

(2*S*,4*S*)-*N*-Methyl-*N*,4-diphenyl-4-(phenylsulfonyl)-2-(phenylthio)butanamide (4aa):

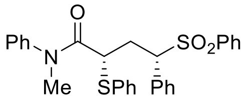

Purification with silica gel column chromatography (hexane/AcOEt = 3:1 to 2:1); 45 mg, 89% yield, dr = 99:1, diastereomers are not separable by silica gel column chromatography; Colorless sticky oil; SFC analysis DAICEL Chiralpak IC-3/SFC 4.6×150 mm (CO_2/MeOH = 80/20, 3.0 mL/min, 220 nm, 40 °C) 14.0 (major of major diastereomer), 16.7 (minor of major diastereomer) min; 94% ee; Optical rotation $[\alpha]_D^{21}$ = −108.5 (*c* 0.090, $CHCl_3$); ^1H NMR for major diastereomer (600 MHz, $CDCl_3$) δ 7.55 (t, *J* = 7.8 Hz, 1H), 7.53 (d, *J* = 7.8 Hz, 2H), 7.39 (dd, *J* = 7.8, 7.8 Hz, 2H), 7.36 (t, *J* = 7.8 Hz, 1H), 7.25-7.21 (m, 3H), 7.20-7.15 (m, 3H), 7.07 (brs, 2H), 6.92-6.89 (m, 4H), 6.84-6.14 (brs, 2H), 4.31 (dd, *J* = 10.8, 4.8 Hz, 1H), 3.46 (dd, *J* = 10.2, 4.8 Hz, 1H), 3.16 (s, 3H), 2.90 (ddd, *J* = 13.8, 10.2, 4.8 Hz, 1H), 2.40 (ddd, *J* = 13.8, 10.8, 4.8 Hz, 1H); ^{13}C NMR for major diastereomer (150 MHz, $CDCl_3$) δ 168.8, 142.3, 137.3, 133.5, 133.2, 132.1, 131.2, 130.3, 129.3, 129.0 (2C), 128.9, 128.73, 128.69, 128.2, 127.7, 127.4, 67.8, 45.1, 37.6, 30.3; IR (ATR): 3415, 3061, 3032, 1655, 1496, 1447, 1386, 1306, 1219, 1146 cm^{-1}; HRMS (ESI) Calcd for $C_{29}H_{27}NO_3S_2$ $[M+Na]^+$ 524.1325, Found 524.1325.

The absolute configuration of major diastereomer of **4aa** was determined to be (2*S*,4*S*) by single-crystal X-ray diffraction analysis of analogous compound **10gb**, which was prepared by the enantioselective addition of **2g** to **3b** followed by oxidation using *m*CPBA.

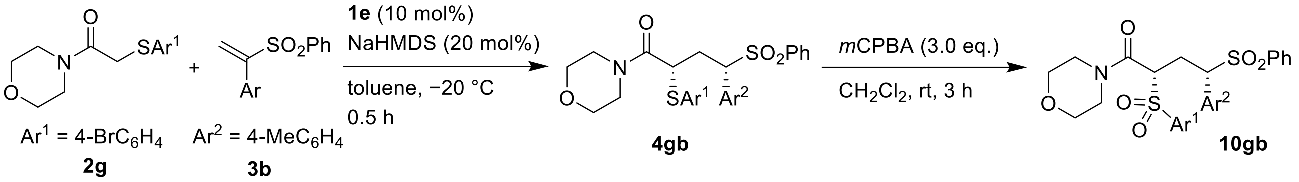

(2*S*,4*S*)-2-((4-Bromophenyl)thio)-4-(4-methylphenyl)-1-morpholino-4-(phenylsulfonyl)butan-1-one (4gb):

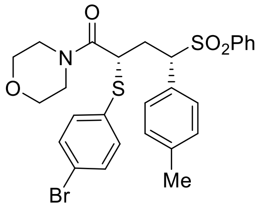

Purification with silica gel column chromatography (hexane/AcOEt = 1:1); 54 mg, 93% yield, dr = 93:7, diastereomers are not separable by silica gel column chromatography; White solid; HPLC analysis DAICEL Chiralcel AD-3 4.6×250 mm (hexane/*i*PrOH= 90/10, 1.0 mL/min, 254 nm, 40 °C) 62.2 (major of major diastereomer), 51.6 (minor of major diastereomer) min; 91% ee; Optical rotation $[\alpha]_D^{24.7}$ = −54.3 (*c* 0.105, $CHCl_3$); IR (ATR): 2922, 2856, 1645, 1446, 1306, 1146, 1116, 1085, 1010, 754 cm^{-1}; ^1H NMR for major diastereomer (600 MHz, $CDCl_3$) δ 7.57-7.53 (m, 3H), 7.42-7.38 (m, 4H), 7.21 (d, *J* = 8.4 Hz, 2H), 7.06 (d, *J* = 7.8 Hz, 2H), 6.95 (d, *J* = 7.8 Hz, 2H), 4.31 (dd, *J* = 9.6, 6.0 Hz, 1H), 3.71 (dd, *J* = 9.6, 6.0 Hz, 1H), 3.58-3.52 (m, 3H), 3.48-3.43 (m, 1H), 3.41-3.37 (m, 1H), 3.29-3.24 (m, 1H), 3.14-3.09 (m, 1H), 2.93-2.88 (m, 1H), 2.83 (ddd, *J* = 15.0, 9.6, 6.0 Hz, 1H), 2.48 (ddd, *J* = 15.0, 9.6, 6.0 Hz, 1H), 2.32 (s, 3H); ^{13}C NMR for major diastereomer (150 MHz, $CDCl_3$) δ 167.5, 139.1, 137.3, 136.0, 133.6, 132.2, 129.8, 129.7, 129.4, 128.9, 128.7, 128.5, 123.6, 67.5, 66.7, 66.1, 46.0, 43.4, 42.3, 30.6, 21.2; HRMS (FD+) Calcd for $C_{27}H_{28}BrNO_4S_2$ [M] 573.0643, Found 573.0643; mp. 75.0-77.0 ºC.

*Transformation of **4gb** to **10gb***

To a solution of **4gb** (50 mg, 0.088 mmol, dr = 93:7, 91% ee) in CH_2Cl_2 (1.0 mL) was added *m*CPBA (ca.70%, 57 mg, 0.23 mmol). The reaction mixture was stirred at room temperature for 3 h. The reaction was quenched with sat.

aq. $NaHCO_3$. The product was extracted with CH_2Cl_2, and the combined organic layer was dried over Na_2SO_4, and concentrated under reduced pressure. The residue was purified by silica gel column chromatography (hexane/AcOEt = 1:1) to afford **10gb** (53 mg, 0.087 mmol, 99%, dr = 93:7, 92% ee) as a white solid.

(2*S*,4*S*)-2-((4-Bromophenyl)sulfonyl)-4-(4-methylphenyl)-1-morpholino-4-(phenylsulfonyl)butan-1-one (10gb):

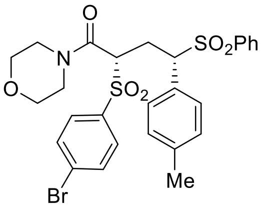

Purification with silica gel column chromatography (hexane/AcOEt = 1:1); 53 mg, 99% yield, dr = 93:7, diastereomers are not separable by silica gel column chromatography; SFC analysis DAICEL Chiralpak IA-3/SFC 4.6×150 mm (CO_2/MeOH = 90/10, 3.0 mL/min, 254 nm, 40 °C) 6.4 (major of major diastereomer), 8.9 (minor of major diastereomer) min; 92% ee; Optical rotation $[\alpha]_D^{24.6}$ = 12.1 (*c* 0.0850, $CHCl_3$); ^{1}H NMR for major diastereomer (600 MHz, $CDCl_3$) δ 7.69 (d, *J* = 8.4 Hz, 2H), 7.61 (d, *J* = 8.4 Hz, 2H), 7.56 (t, *J* = 7.2 Hz, 1H), 7.50 (d, *J* = 7.2 Hz, 2H), 7.38 (dd, *J* = 7.2, 7.2 Hz, 2H), 7.11 (d, *J* = 7.2 Hz, 2H), 6.93 (d, *J* = 7.2 Hz, 2H), 4.13 (dd, *J* = 10.8, 4.2 Hz, 1H), 4.02 (dd, *J* = 11.4, 5.4 Hz, 1H) 3.74-3.69 (m, 1H), 3.64-3.56 (m, 2H), 3.55-3.50 (m, 1H), 3.36-3.22 (m, 3H), 2.81-2.70 (m, 2H), 2.64 (ddd, *J* = 13.2, 10.8, 5.4 Hz, 1H), 2.34 (s, 3H); ^{13}C NMR for major diastereomer (150 MHz, $CDCl_3$) δ 161.6, 139.7, 136.8, 134.2, 133.8, 132.3, 131.2, 130.4, 129.8, 129.7, 128.9, 128.8, 127.2, 66.7, 66.4, 65.9, 62.7, 46.7, 42.8, 26.4, 21.2; IR (ATR): 2923, 2858, 1650, 1574, 1446, 1320, 1147, 1117, 1083, 786 cm^{-1}; HRMS (FD+) Calcd for $C_{27}H_{28}BrNO_6S_2$ [M] 605.0541, Found 605.0540; mp. 184.0-186.0 ºC.

ORTEP drawing of (2*S*,4*S*)-**10gb** showing thermal elipsoids at the 50% probability level. CCDC No. 2272299. Recrystallization from hexane/AcOEt.

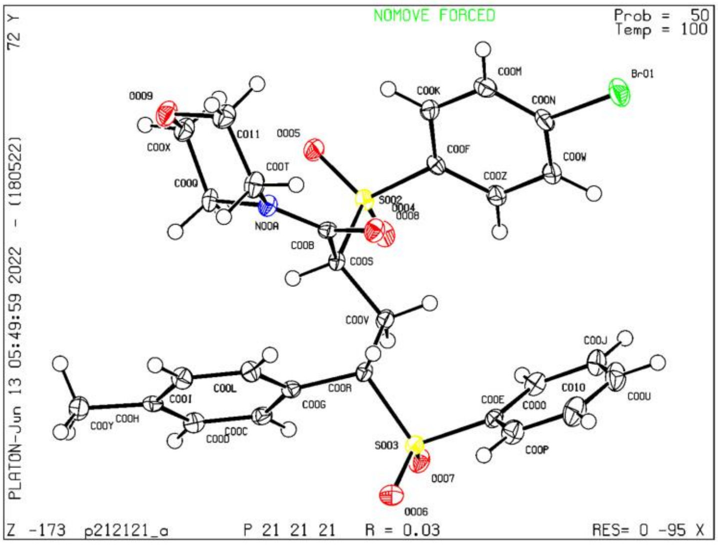

(2*S*,4*S*)-*N*-Benzyl-*N*,4-diphenyl-4-(phenylsulfonyl)-2-(phenylthio)butanamide (4ba):

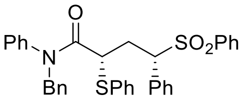

Purification with silica gel column chromatography (hexane/AcOEt = 3:1) followed by gel permeation chromatography; 45 mg, 79% yield, dr = 96:4, diastereomers are not separable by silica gel column chromatography; Colorless sticky oil; SFC analysis DAICEL Chiralpak IC-3/SFC 4.6×150 mm (CO_2/MeOH = 80/20, 3.0 mL/min, 254 nm, 40 °C) 9.6 (major of major diastereomer), 13.0 (minor of major diastereomer) min; 72% ee; Optical rotation $[\alpha]_D^{24.4}$ = −50.2 (*c* 0.110, $CHCl_3$); ^{1}H NMR for major diastereomer (600 MHz, $CDCl_3$) δ 7.58-7.54 (m, 3H), 7.41 (dd, *J* = 8.4, 7.2 Hz, 2H), 7.32 (t, *J* = 7.8 Hz, 1H), 7.29-7.23 (m, 6H), 7.18-7.10 (m, 7H), 7.09-6.79 (brs, 2H), 6.90 (dd, *J* = 8.4, 1.2 Hz, 2H), 6.83 (d, *J* = 6.6 Hz, 2H), 4.91 (d, *J* = 14.4 Hz, 1H), 4.57 (d, *J* = 14.4 Hz, 1H), 4.33 (dd, *J* = 11.4, 4.8 Hz, 1H), 3.36 (dd, *J* = 10.8, 4.8 Hz, 1H), 2.87 (ddd, *J* = 13.8, 10.8, 4.8 Hz, 1H), 2.41 (ddd, *J* = 13.8, 11.4, 4.8 Hz, 1H); ^{13}C NMR for major diastereomer (150 MHz, $CDCl_3$) δ 168.5, 140.5, 137.3, 136.9, 133.5, 133.3, 131.8, 130.9, 130.3, 129.0, 128.92, 128.91 (2C), 128.8, 128.7 (3C), 128.2 (2C), 127.8, 127.5, 67.9, 53.3, 44.9, 30.3; IR (ATR): 3061, 2929, 1654, 1495, 1398, 1319, 1307, 1147, 794, 699 cm^{-1}; HRMS (FD+) Calcd for $C_{35}H_{31}NO_3S_2$ [M] 577.1745, Found 577.1743.
The absolute configuration of the major diastereomer was assigned as (2*S*,4*S*) by analogy.

(2*S*,4*S*)-*N*,*N*-Dimethyl-4-phenyl-4-(phenylsulfonyl)-2-(phenylthio)butanamide (4ca):

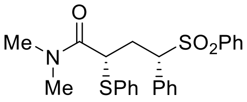

Purification with silica gel column chromatography (hexane/AcOEt = 1:1); 40 mg, 90% yield, dr = 97:3, diastereomers are not separable by silica gel column chromatography; White solid; HPLC analysis DAICEL Chiralcel AD-3 4.6×250 mm (hexane/*i*PrOH= 90/10, 0.95 mL/min, 254 nm, 30 °C) 39.4 (major of major diastereomer), 37.2 (minor of major diastereomer) min; 87% ee; Optical rotation $[\alpha]_D^{19.3}$ = −115.0 (*c* 0.210, $CHCl_3$); ^{1}H NMR for major diastereomer (600 MHz, $CDCl_3$) δ 7.56 (d, *J* = 7.8 Hz, 2H), 7.54 (t, *J* = 7.2 Hz, 1H), 7.39 (dd, *J* = 7.8, 7.8 Hz, 2H), 7.36 (d, *J* = 7.8 Hz, 2H), 7.33-7.27 (m, 4H), 7.24 (dd, *J* = 7.8, 7.8 Hz, 2H), 7.05 (d, *J* = 7.2 Hz, 2H), 4.33 (dd, *J* = 9.6, 5.4 Hz, 1H), 3.75 (dd, *J* = 15.0, 5.4 Hz, 1H), 2.89 (ddd, *J* = 15.0, 9.6, 5.4 Hz, 1H), 2.84 (s, 3H), 2.53 (ddd, *J* = 15.0, 9.6, 5.4 Hz, 1H), 2.48 (s, 3H); ^{13}C NMR for major diastereomer (150 MHz, $CDCl_3$) δ 169.0, 137.4, 134.2, 133.5, 131.8, 131.5, 129.9, 129.0, 128.93, 128.88, 128.7 (2C), 128.6, 67.9, 44.4, 36.8, 35.9, 30.7; IR (ATR): 3061, 2931, 1645, 1446, 1322, 1306, 1146, 1084, 751, 690 cm^{-1}; HRMS (FD+) Calcd for $C_{24}H_{25}NO_3S_2$ [M] 439.1276, Found 439.1274; mp. 124.0-126.0 °C.
The absolute configuration of the major diastereomer was assigned as (2*S*,4*S*) by analogy.

(2*S*,4*S*)-4-Phenyl-4-(phenylsulfonyl)-2-(phenylthio)-1-(pyrrolidin-1-yl)butan-1-one (4da):

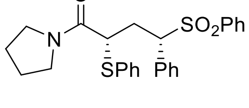

Purification with silica gel column chromatography (hexane/AcOEt = 1:1); 39 mg, 83% yield, dr = 93:7, diastereomers were separated by preparative HPLC before analysis; White solid; SFC analysis DAICEL Chiralpak IF-3/SFC 4.6×150 mm (CO_2/MeOH = 80/20, 3.0 mL/min, 254 nm, 40 °C) 9.9 (major), 8.2 (minor) min; 60% ee; Optical rotation $[\alpha]_D^{19.6}$ = −82.6 (*c* 0.14, $CHCl_3$); ^{1}H NMR (600 MHz, $CDCl_3$) δ 7.58 (d, *J* = 7.2 Hz, 2H), 7.54 (t, *J* = 7.2 Hz, 1H), 7.42-7.37 (m, 4H), 7.33-7.25 (m, 4H), 7.24 (dd, *J* = 7.8, 7.2 Hz, 2H), 7.06 (d, *J* = 7.2 Hz, 2H), 4.36 (dd, *J* = 10.2, 6.0 Hz, 1H), 3.57 (dd, *J* = 10.2, 6.0 Hz, 1H), 3.34 (dd, *J* = 6.6, 6.6 Hz, 2H), 2.95-2.90 (m, 1H), 2.86 (ddd, *J* = 13.8, 10.2, 6.0 Hz, 1H), 2.54 (ddd, *J* = 13.8, 10.2, 6.0 Hz, 1H), 2.30-2.24 (m, 1H), 1.72-1.57 (m, 4H); ^{13}C NMR (150 MHz, $CDCl_3$) δ 167.4, 137.4, 134.2, 133.5, 131.71, 131.68, 130.0, 129.0, 128.93, 128.89, 128.73, 128.67, 128.6, 67.9, 46.5, 45.90, 45.85, 30.3, 25.7,

24.1; IR (ATR): 2972, 2874, 1639, 1440, 1322, 1305, 1146, 1085, 751, 690 cm^{-1}; HRMS (FD+) Calcd for $C_{26}H_{27}NO_3S_2$ [M] 465.1432, Found 465.1431; mp. 162.0-164.0 ºC.
The absolute configuration of the major diastereomer was assigned as (2*S*,4*S*) by analogy.

(2*S*,4*S*)-*N*-Methyl-2-(methylthio)-*N*,4-diphenyl-4-(phenylsulfonyl)butanamide (4ea):

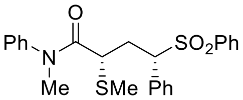

Purification with silica gel column chromatography (hexane/AcOEt = 3:1 to 2:1); 30 mg, 68% yield, dr = 99:1, diastereomers are not separable by silica gel column chromatography; Colorless sticky oil; SFC analysis DAICEL Chiralpak IF-3/SFC 4.6×150 mm (CO_2/MeOH = 90/10, 3.0 mL/min, 220 nm, 40 °C) 9.1 (minor of major diastereomer), 10.4 (major of major diastereomer) min; 88% ee; Optical rotation $[\alpha]_D^{19.6}$ = −72.6 (*c* 0.115, $CHCl_3$); ^{1}H NMR for major diastereomer (600 MHz, $CDCl_3$) δ 7.57-7.53 (m, 3H), 7.39 (dd, *J* =7.8, 7.8 Hz, 2H), 7.37 (t, *J* = 7.8 Hz, 1H), 7.28-7.24 (m, 2H), 7.16 (t, *J* = 7.8 Hz, 1H), 7.09 (brs, 2H), 7.04 (d, *J* = 7.2 Hz, 2H), 6.59 (brs, 2H), 4.36 (dd, *J* = 10.8, 4.2 Hz, 1H), 3.14 (s, 3H), 3.04-2.99 (m, 2H), 2.45-2.39 (m, 1H), 1.97 (s, 3H); ^{13}C NMR for major diastereomer (150 MHz, $CDCl_3$) δ 168.8, 142.3, 137.2, 133.5, 131.6, 130.3, 129.2, 129.0, 128.9, 128.8, 128.6, 127.7, 127.3, 68.1, 39.5, 37.4, 28.7, 11.8; IR (ATR): 3062, 2923, 1650, 1595, 1496, 1384, 1306, 1146, 791, 698 cm^{-1}; HRMS (FD+) Calcd for $C_{24}H_{25}NO_3S_2$ [M] 439.1276, Found 439.1274.
The absolute configuration of the major diastereomer was assigned as (2*S*,4*S*) by analogy.

(2*R*,4*S*) - or (2*S*,4*R*)-*N*-Methyl-*N*,2,4-triphenyl-4-(phenylsulfonyl)butanamide (4fa):

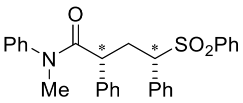

Purification with silica gel column chromatography (hexane/AcOEt = 3:1); 43 mg, 92% yield, dr = 96:4, diastereomers were separated by silica gel column chromatography; White solid; HPLC analysis DAICEL Chiralcel OD-3 4.6×250 mm (hexane/*i*PrOH= 90/10, 1.0 mL/min, 220 nm, 30 °C) 12.3 (major), 14.2 (minor) min; 64% ee; Optical rotation $[\alpha]_D^{24.6}$ = −71.4 (*c* 0.110, $CHCl_3$); ^{1}H NMR (600 MHz, $CDCl_3$) δ 7.57 (d, *J* = 7.2 Hz, 2H), 7.55 (t, *J* = 7.2 Hz,1H), 7.41-7.37 (m, 3H), 7.31 (dd, *J* = 7.8, 7.8 Hz, 2H), 7.18-7.10 (m, 6H), 6.97 (dd, *J* = 7.8, 7.8 Hz, 2H), 6.77 (d, *J* = 7.2 Hz, 2H), 6.09 (brs, 2H), 4.48 (dd, *J* = 11.4, 4.8 Hz, 1H), 3.36 (dd, *J* = 11.4, 4.8Hz, 1H), 3.09 (s, 3H), 2.99 (ddd, *J* = 13.8, 11.4, 4.8 Hz, 1H), 2.33 (ddd, *J* = 13.8, 11.4, 4.8 Hz, 1H); ^{13}C NMR (150 MHz, $CDCl_3$) δ 171.0, 142.4, 139.1, 137.5, 133.4, 131.9, 130.3, 129.0 (2C), 128.8, 128.7, 128.6, 128.4, 127.7, 127.6, 127.5, 127.1, 68.7, 46.2, 37.4, 32.5; IR (ATR): 3060, 3027, 1654, 1596, 1496, 1447, 1307, 1147, 753, 698 cm^{-1}; HRMS (FD+) Calcd for $C_{29}H_{27}NO_3S$ [M] 469.1712, Found 469.1710; mp. 157.0-159.0 °C.
The 1,3-*syn* configuration was confirmed by the single-crystal X-ray diffraction analysis of the racemic **4fa**.

ORTEP drawing of (2*R**,4*S**)-**4fa** showing thermal elipsoids at the 50% probability level. CCDC No. 2272322. Recrystallization from hexane/methanol.

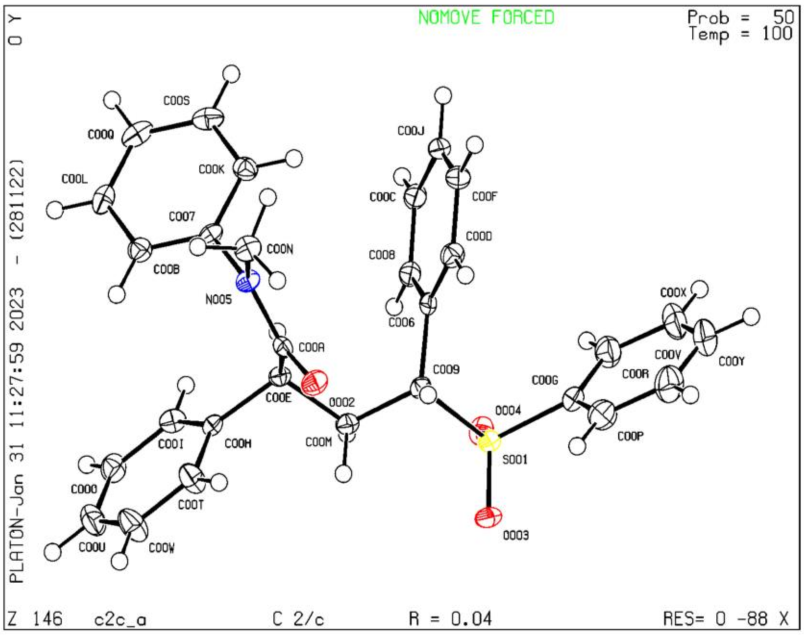

(2*S*,4*S*)-*N*-Methyl-4-(4-methylphenyl)-*N*-phenyl-4-(phenylsulfonyl)-2-(phenylthio)butanamide (4ab):

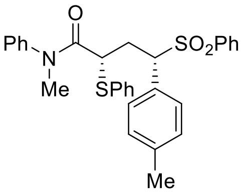

Purification with silica gel column chromatography (hexane/AcOEt = 3:1 to 2:1); 41 mg, 79% yield, dr = 99:1, diastereomers are not separable by silica gel column chromatography; Pale yellow sticky oil; SFC analysis DAICEL Chiralpak IF-3/SFC 4.6×150 mm (CO_2/MeOH = 80/20, 3.0 mL/min, 220 nm, 40 °C) 6.0 (minor of major diastereomer), 8.3 (major of major diastereomer) min; 93% ee; Optical rotation $[\alpha]_D^{19.6}$ = −170.3 (*c* 0.080, $CHCl_3$); ^{1}H NMR for major diastereomer (600 MHz, $CDCl_3$) δ 7.57-7.57 (m, 3H), 7.40 (dd, *J* = 7.8, 7.8 Hz, 2H), 7.24 (t, *J* = 7.8 Hz, 1H), 7.19-7.15 (m, 3H), 7.08-7.03 (m, 4H), 6.90 (d, *J* = 7.8 Hz, 2H), 6.80 (d, *J* = 7.8 Hz, 2H), 6.78-6.01 (brs, 2H), 4.28 (dd, *J* = 11.4, 4.8 Hz, 1H), 3.45 (dd, *J* = 13.8, 4.8 Hz, 1H), 3.15 (s, 3H), 2.84 (ddd, *J* = 13.8, 11.4, 4.8 Hz, 1H), 2.40 (s, 3H), 2.37 (ddd, *J* = 13.8, 11.4, 4.8 Hz, 1H); ^{13}C NMR for major diastereomer (150 MHz, $CDCl_3$) δ 168.7, 142.3, 138.9, 137.3, 133.4, 133.1, 132.1, 130.1, 129.4, 129.1, 128.9, 128.8, 128.6, 128.1, 127.9, 127.7, 127.4, 67.5, 44.9, 37.6, 30.2, 21.2; IR (ATR): 3060, 2925, 1654, 1496, 1439, 1385, 1307, 1146, 788, 758 cm^{-1}; HRMS (ESI) Calcd for $C_{30}H_{29}NO_3S_2$ $[M+Na]^+$ 538.1481, Found 538.1481.

The absolute configuration of the major diastereomer was assigned as (2*S*,4*S*) by analogy.

(2*S*,4*S*)-4-(4-Fluorophenyl)-*N*-methyl-*N*-phenyl-4-(phenylsulfonyl)-2-(phenylthio)butanamide (4ac):

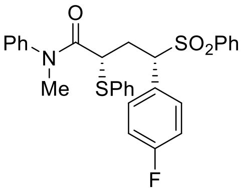

Purification with silica gel column chromatography (hexane/AcOEt = 2:1); 52 mg, 99% yield, dr = 98:2, diastereomers are not separable by silica gel column chromatography; Pale yellow sticky oil; SFC analysis DAICEL Chiralpak IC-3/SFC 4.6×150 mm (CO_2/MeOH = 80/20, 3.0 mL/min, 254 nm, 40 °C) 8.6 (major of major diastereomer), 10.0 (minor of major diastereomer) min; 92% ee; Optical rotation $[\alpha]_D^{19.1}$ = −107.3 (*c* 0.175, $CHCl_3$); ^{1}H NMR for major diastereomer (600 MHz, $CDCl_3$) δ 7.57 (tt, *J* = 7.8, 1.2 Hz, 1H), 7.54 (dd, *J* = 8.4, 1.2 Hz, 2H),

7.41 (dd, J = 7.2, 7.2 Hz, 2H), 7.26 (tt, J = 7.8, 1.2 Hz, 1H), 7.23 (tt, J = 7.2, 1.2 Hz, 1H), 7.18 (dd, J = 7.8, 7.8 Hz, 2H), 7.14 (dd, J = 7.2, 7.2 Hz, 2H), 6.94-6.90 (m, 4H), 6.88-6.85 (m, 2H), 6.85-6.30 (brs, 2H), 4.28 (dd, J = 11.4, 4.8 Hz, 1H), 3.44 (dd, J = 14.4, 4.8 Hz, 1H), 3.18 (s, 3H), 2.89 (ddd, J = 14.4, 11.4, 4.8 Hz, 1H), 2.33 (ddd, J = 14.4, 11.4, 4.8 Hz, 1H); ^{13}C NMR for major diastereomer (150 MHz, $CDCl_3$) δ 168.7, 163.1 (d, J = 248.4 Hz), 142.2, 137.0, 133.6, 133.3, 132.0, 131.8 (d, J = 7.2 Hz), 129.3, 128.88, 128.85, 128.8, 128.3, 127.9, 127.3, 127.1, 115.6 (d, J = 21.6 Hz), 66.9, 45.1, 37.6, 30.4; ^{19}F NMR for major diastereomer (565 MHz, $CDCl_3$) δ −111.9; IR (ATR): 3061, 2932, 1654, 1509, 1146, 784, 757, 696, 688, 602 cm^{-1}; HRMS (ESI) Calcd for $C_{29}H_{26}FNO_3S_2$ [M+Na]$^+$ 542.1230, Found 542.1231.

The absolute configuration of the major diastereomer was assigned as (2*S*,4*S*) by analogy.

(2*S*,4*S*)-4-(4-Chlorophenyl)-*N*-methyl-*N*-phenyl-4-(phenylsulfonyl)-2-(phenylthio)butanamide (4ad):

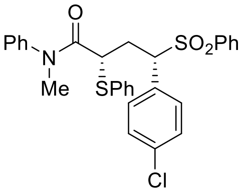

Purification with silica gel column chromatography (hexane/AcOEt = 3:1 to 2:1); 53 mg, 99% yield, dr = 83:17, diastereomers are not separable by silica gel column chromatography; Pale yellow sticky oil; SFC analysis DAICEL Chiralpak IC-3/SFC 4.6×150 mm (CO_2/MeOH = 80/20, 3.0 mL/min, 254 nm, 40 °C) 12.1 (major of major diastereomer), 14.0 (minor of major diastereomer) min; 88% ee; Optical rotation $[\alpha]_D^{18.5}$ = −106.6 (*c* 0.175, $CHCl_3$); ^{1}H NMR for major diastereomer (600 MHz, $CDCl_3$) δ 7.58 (t, J = 7.8 Hz, 1H), 7.55 (d, J = 7.8 Hz, 2H), 7.43 (dd, J = 7.8, 7.8 Hz, 2H), 7.26-7.13 (m, 8H), 6.92 (dd, J = 8.4, 1.2 Hz, 2H), 6.82 (d, J = 8.4 Hz, 2H), 6.88-6.25 (brs, 2H), 4.27 (dd, J = 10.2, 4.8 Hz, 1H), 3.40 (dd, J = 14.4, 4.8 Hz, 1H), 3.17 (s, 3H), 2.87 (ddd, J = 14.4, 10.2, 5.4 Hz, 1H), 2.33 (ddd, J = 13.8, 10.8, 4.8 Hz, 1H); ^{13}C NMR for major diastereomer (150 MHz, $CDCl_3$) δ 168.6, 142.2, 137.0, 135.2, 133.7, 133.3, 131.9, 131.5, 129.8, 129.4, 128.93, 128.91, 128.89, 128.86, 128.4, 128.0, 127.3, 67.0, 45.0, 37.6, 30.3; IR (ATR): 3061, 2934, 1655, 1595, 1494, 1307, 1147, 1084, 794 cm^{-1}; HRMS (FD+) Calcd for $C_{29}H_{26}ClNO_3S_2$ [M]$^+$ 535.1043, Found 535.1040.

The absolute configuration of the major diastereomer was assigned as (2*S*,4*S*) by analogy.

(2*S*,4*S*)-*N*-Methyl-4-(naphthalen-2-yl)-*N*-phenyl-4-(phenylsulfonyl)-2-(phenylthio)butanamide (4ae):

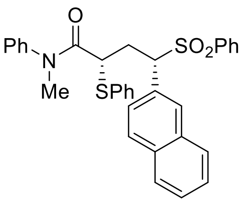

Purification with silica gel column chromatography (hexane/AcOEt = 3:1 to 2:1); 55 mg, 99% yield, dr = 92:8, diastereomers are not separable by silica gel column chromatography; Pale yellow sticky oil; SFC analysis DAICEL Chiralpak IC-3/SFC 4.6×150 mm (CO_2/MeOH = 80/20, 3.0 mL/min, 254 nm, 40 °C) 21.5 (major of major diastereomer), 25.0 (minor of major diastereomer) min; 93% ee; Optical rotation $[\alpha]_D^{19.4}$ = −217.4 (*c* 0.155, $CHCl_3$); ^{1}H NMR for major diastereomer (600 MHz, $CDCl_3$) δ 7.87 (d, J = 7.8 Hz, 1H), 7.70 (d, J = 8.4 Hz, 1H), 7.62 (d, J = 8.4 Hz, 1H), 7.59-7.50 (m, 6H), 7.37 (dd, J = 8.4, 7.2 Hz, 2H), 7.32 (s, 1H), 7.29-7.26 (m, 2H), 7.18 (dd, J = 7.8, 7.8 Hz, 2H), 7.01 (dd, J = 7.2, 1.2 Hz, 1H), 6.94 (dd, J = 7.8, 7.8 Hz, 1H), 6.88 (d, J = 7.8 Hz, 2H), 6.80-6.30 (br, 2H), 4.47 (dd, J = 12.0, 4.8 Hz, 1H), 3.43 (dd, J = 10.8, 4.2 Hz, 1H), 3.12 (s, 3H), 2.97 (ddd, J = 13.8, 10.8, 4.8 Hz, 1H), 2.51 (ddd, J = 14.4, 12.0, 6.0 Hz, 1H); ^{13}C NMR for major diastereomer (150 MHz, $CDCl_3$) δ 168.6, 142.0, 137.2, 133.5, 133.3, 133.04, 133.00, 132.2, 130.1, 129.0 128.94, 128.90 (2C), 128.87, 128.8, 128.7, 128.4, 128.2, 128.0, 127.5, 127.1, 126.9, 126.5, 67.8, 45.1, 37.5, 30.3; IR (ATR): 3056, 2937, 1654, 1307, 1146, 784, 755, 741, 698, 688 cm^{-1}; HRMS (ESI) Calcd for $C_{33}H_{29}NO_3S_2$ [M+Na]$^+$ 574.1481, Found 574.1481.

The absolute configuration of the major diastereomer was assigned as (2*S*,4*S*) by analogy.

(2*S*,4*S*)-*N*-Methyl-4-(2-methylphenyl)-*N*-phenyl-4-(phenylsulfonyl)-2-(phenylthio)butanamide (4af):

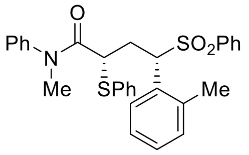

Purification with silica gel column chromatography (hexane/AcOEt = 3:1); 44 mg, 84% yield, dr = 95:5, diastereomers are not separable by silica gel column chromatography; Pale yellow sticky oil; SFC analysis DAICEL Chiralpak IF-3/SFC 4.6×150 mm (CO_2/MeOH = 80/20, 3.0 mL/min, 254 nm, 40 °C) 5.6 (minor of major diastereomer), 6.9 (major of major diastereomer) min; 76% ee; Optical rotation $[\alpha]_D^{19.6}$ = −63.4 (*c* 0.115, $CHCl_3$); ^{1}H NMR for major diastereomer (600 MHz, $CDCl_3$) δ 7.59-7.54 (m, 3H), 7.40 (dd, *J* = 7.8, 7.8 Hz, 2H), 7.29 (t, *J* = 7.8 Hz, 1H), 7.24 (t, *J* = 7.8 Hz, 1H), 7.19-7.15 (m, 4H), 7.13 (t, *J* = 7.8 Hz, 1H), 7.10-7.03 (m, 3H), 6.89 (d, *J* = 8.4 Hz, 2H), 6.70-6.10 (brs, 2H), 4.75 (dd, *J* = 10.8, 4.8 Hz, 1H), 3.39 (dd, *J* = 10.8, 4.8 Hz, 1H), 3.15 (s, 3H), 2.82 (ddd, *J* = 13.8, 10.8, 4.8 Hz, 1H), 2.41 (ddd, *J* = 13.8, 10.8, 4.8 Hz, 1H), 1.99 (s, 3H); ^{13}C NMR for major diastereomer (150 MHz, $CDCl_3$) δ 168.6, 142.1, 139.2, 137.8, 133.5, 133.0, 132.0, 130.7, 129.5, 129.2, 128.9, 128.82, 128.77 (2C), 128.71, 128.1, 127.7, 127.4, 126.7, 62.4, 44.5, 37.5, 37.5, 30.8, 19.1; IR (ATR): 3061, 2933, 1653, 1496, 1445, 1439, 1384, 1321, 1306, 1146 cm^{-1}; HRMS (ESI) Calcd for $C_{30}H_{29}NO_3S_2$ $[M+Na]^+$ 538.1481, Found 538.1481.
The absolute configuration of the major diastereomer was assigned as (2*S*,4*S*) by analogy.

(2*S*,4*R*)-*N*-Methyl-*N*-phenyl-4-(phenylsulfonyl)-2-(phenylthio)octanamide (4ag):

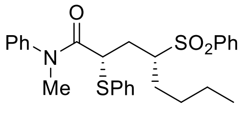

Purification with silica gel column chromatography (hexane/AcOEt = 3:1) followed by gel permeation chromatography; 41 mg, 86% yield, dr = 95:5, diastereomers were separated by silica gel column chromatography; Colorless oil; SFC analysis DAICEL Chiralpak IF-3/SFC 4.6×150 mm (CO_2/MeOH = 80/20, 3.0 mL/min, 254 nm, 40 °C) 3.2 (major), 3.9 (minor) min; 94% ee; Optical rotation $[\alpha]_D^{21.8}$ = −77.0 (*c* 0.135, $CHCl_3$); ^{1}H NMR (600 MHz, $CDCl_3$) δ 7.61-7.57 (m, 3H), 7.46 (dd, *J* = 7.8, 7.8 Hz, 2H), 7.39-7.32 (m, 3H), 7.24 (tt, *J* = 7.2, 1.8 Hz, 1H), 7.19 (dd, *J* = 7.8, 7.8 Hz, 2H), 7.13 (br, 2H), 7.10 (d, *J* = 7.8 Hz, 2H), 4.38 (dd, *J* = 10.2, 4.8 Hz, 1H), 3.29 (s, 3H), 3.19-3.13 (m, 1H), 2.46 (ddd, *J* = 15.6, 10.2, 4.8 Hz, 1H), 2.16 (ddd, *J* = 15.6, 10.2, 3.0 Hz, 1H), 1.57-1.48 (m, 1H), 1.37-1.25 (m, 2H), 1.18-1.00 (m, 3H), 0.73 (t, *J* = 7.2 Hz, 3H); ^{13}C NMR (150 MHz, $CDCl_3$) δ 171.0, 142.7, 137.7, 133.5, 133.3, 132.3, 129.7, 129.0, 128.9, 128.6, 128.0, 127.6, 127.5, 60.6, 46.4, 37.9, 30.9, 29.0, 27.9, 22.2, 13.5; IR (ATR): 2956, 2931, 1655, 1595, 1496, 1383, 1302, 1144, 795, 692 cm^{-1}; HRMS (FD+) Calcd for $C_{27}H_{31}NO_3S_2$ [M] 481.1745, Found 481.1744.
The absolute configuration of the major diastereomer was assigned as (2*S*,4*R*) by analogy.

(2*S*,4*R*)-*N*-Methyl-*N*,6-diphenyl-4-(phenylsulfonyl)-2-(phenylthio)hexanamide (4ah):

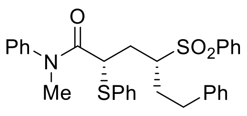

Purification with silica gel column chromatography (hexane/AcOEt = 3:1) followed by gel permeation chromatography; 45 mg, 85% yield, dr = 96:4, diastereomers were separated by silica gel column chromatography; Colorless oil; SFC analysis DAICEL Chiralpak IF-3/SFC 4.6×150 mm (CO_2/MeOH = 85/15, 3.0 mL/min, 254 nm, 40 °C) 8.8 (major), 11.7 (minor) min; 94% ee; Optical rotation $[\alpha]_D^{24.4}$ = −43.8 (*c* 0.110, $CHCl_3$); ^{1}H NMR (600 MHz, $CDCl_3$) δ 7.58 (t, *J* = 7.2 Hz, 1H), 7.50 (d, *J* = 7.2 Hz, 2H), 7.42 (dd, *J* = 7.8, 7.8 Hz, 2H), 7.37-7.32 (m, 3H), 7.22 (t, *J* = 7.8 Hz, 1H), 7.18-7.11 (m, 7H), 6.98 (d, *J* = 7.8 Hz, 2H), 6.88 (d, *J* = 7.8 Hz, 2H), 4.39 (dd, *J* = 9.6, 4.8 Hz, 1H), 3.29 (s, 3H), 3.23-3.18 (m, 1H), 2.71 (ddd, *J* = 14.4, 9.6, 4.8 Hz, 1H), 2.55 (ddd, *J* = 14.4, 7.2, 4.8 Hz, 1H), 2.43 (ddd, *J* = 16.2, 9.6, 7.8 Hz, 1H), 2.26

(ddd, *J* = 16.2, 9.6, 3.6 Hz, 1H), 1.91-1.85 (m, 1H), 1.71-1.63 (m, 1H); ^{13}C NMR (150 MHz, $CDCl_3$) δ 170.9, 142.6, 140.1, 137.5, 133.5, 133.3, 132.2, 129.8, 129.0, 128.9, 128.5, 128.4, 128.2, 128.0, 127.6, 127.5, 126.1, 59.8, 46.3, 37.8, 31.7, 31.1, 30.7; IR (ATR): 3060, 3027, 2934, 1654, 1595, 1496, 1304, 1142, 750, 698 cm^{-1}; HRMS (FD+) Calcd for $C_{32}H_{32}NO_3S_2$ [M] 529.1745, Found 529.1745.

The absolute configuration of the major diastereomer was assigned as (2*S*,4*R*) by analogy.

(2*S*,4*R*)-*N*-Methyl-*N*-phenyl-4-(phenylsulfonyl)-2-(phenylthio)non-8-enamide (4ai):

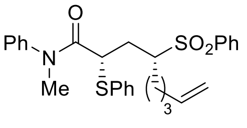

Purification with silica gel column chromatography (hexane/AcOEt = 3:1) followed by gel permeation chromatography; 36 mg, 72% yield, dr = 95:5, diastereomers were separated by silica gel column chromatography; Colorless oil; SFC analysis DAICEL Chiralpak IF-3/SFC 4.6×150 mm (CO_2/MeOH = 85/15, 3.0 mL/min, 254 nm, 40 °C) 4.6 (major), 5.7 (minor) min; 94% ee; Optical rotation $[\alpha]_D^{23.8}$ = −58.9 (*c* 0.125, $CHCl_3$); ^{1}H NMR (600 MHz, $CDCl_3$) δ 7.61-7.57 (m, 3H), 7.46 (dd, *J* = 7.8, 7.2 Hz, 2H), 7.40-7.32 (m, 3H), 7.24 (tt, *J* = 7.8, 1.2 Hz, 1H), 7.19 (dd, *J* = 7.8, 7.8 Hz, 2H), 7.16-7.11 (br, 2H), 7.08 (d, *J* = 7.8 Hz, 2H), 5.57 (ddt, *J* = 16.8, 10.2, 6.6 Hz, 1H), 4.87-4.80 (m, 2H), 4.38 (dd, *J* = 9.6, 4.8 Hz, 1H), 3.29 (s, 3H), 3.19-3.14 (m, 1H), 2.15 (ddd, *J* = 15.6, 9.6, 4.8 Hz, 1H), 2.47 (ddd, *J* = 15.6, 9.6, 4.8 Hz, 1H), 1.93-1.78 (m, 2H), 1.56-1.50 (m, 1H), 1.49-1.40 (m, 1H), 1.39-1.31 (m, 1H), 1.26-1.17 (m, 1H); ^{13}C NMR (150 MHz, $CDCl_3$) δ 170.9, 142.7, 137.6, 137.4, 133.5, 133.3, 132.2, 129.8, 128.98, 128.95, 128.6, 128.0, 127.6, 127.5, 115.2, 60.5, 46.3, 37.9, 33.1, 30.9, 28.6, 24.9; IR (ATR): 3061, 2931, 1655, 1595, 1496, 1446, 1384, 1302, 1143, 692 cm^{-1}; HRMS (FD+) Calcd for $C_{28}H_{31}NO_3S_2$ [M] 493.1745, Found 493.1744.

The absolute configuration of the major diastereomer was assigned as (2*S*,4*R*) by analogy.

(2*S*,4*R*)-8-(*tert*-Butyldimethylsiloxy)-*N*-methyl-*N*-phenyl-4-(phenylsulfonyl)-2-(phenylthio)octanamide (4aj):

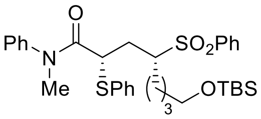

Purification with silica gel column chromatography (hexane/AcOEt = 3:1); 56 mg, 91% yield, dr = 95:5, diastereomers were separated by silica gel column chromatography; Pale yellow oil; SFC analysis DAICEL Chiralpak IF-3/SFC 4.6×150 mm (CO_2/MeOH = 80/20, 3.0 mL/min, 254 nm, 40 °C) 3.0 (major), 3.5 (minor) min; 96% ee; Optical rotation $[\alpha]_D^{19.6}$ = −45.0 (*c* 0.155, $CHCl_3$); ^{1}H NMR (600 MHz, $CDCl_3$) δ 7.59 (t, *J* = 7.2 Hz, 1H), 7.57 (d, *J* = 7.2 Hz, 2H), 7.45 (dd, *J* = 7.8, 7.8 Hz, 2H), 7.40-7.31 (m, 3H), 7.24 (t, *J* = 7.8 Hz, 1H), 7.18 (dd, *J* = 7.8, 7.8 Hz, 2H), 7.13 (br, 2H), 7.07 (d, *J* = 7.8 Hz, 2H), 4.40 (dd, *J* = 10.2, 4.2 Hz, 1H), 3.44 (t, *J* = 6.6 Hz, 2H), 3.29 (s, 3H), 3.19-3.13 (m, 1H), 2.46 (ddd, *J* = 15.6, 8.4, 4.2 Hz, 1H), 2.17 (ddd, *J* = 15.6, 10.2, 3.0 Hz, 1H), 1.56-1.49 (m, 1H), 1.45-1.23 (m, 4H), 1.20-1.10 (m, 1H), 0.85 (s, 9H), 0.00 (s, 3H), −0.01 (s, 3H); ^{13}C NMR (150 MHz, $CDCl_3$) δ 171.0, 142.7, 137.6, 133.46, 133.43, 132.1, 129.7, 128.96, 128.92, 128.5, 128.0, 127.6, 127.5, 62.5, 60.6, 46.3, 37.8, 32.3, 31.0, 29.2, 25.9, 22.3, 18.3, −5.4; IR (ATR): 2951, 2928, 2856, 1656, 1303, 1143, 1100, 1085, 835, 691 cm^{-1}; HRMS (FD+) Calcd for $C_{33}H_{45}NO_4S_2Si$ [M] 611.2559, Found 611.2557.

The absolute configuration of the major diastereomer was assigned as (2*S*,4*R*) by analogy.

(2*S*,4*S*)-*N*,5-Dimethyl-*N*-phenyl-4-(phenylsulfonyl)-2-(phenylthio)hexanamide (4ak):

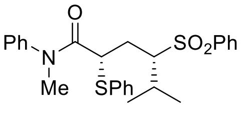

Purification with silica gel column chromatography (hexane/AcOEt = 3:1) followed by gel permeation chromatography; 40 mg, 84% yield, dr = 95:5, diastereomers were separated by silica gel column chromatography; White solid; SFC analysis DAICEL Chiralpak IC-3/SFC

4.6×150 mm (CO_2/MeOH = 80/20, 3.0 mL/min, 220 nm, 40 °C) 3.8 (major), 4.7 (minor) min; 94% ee; Optical rotation $[\alpha]_D^{24.4}$ = −57.0 (*c* 0.110, $CHCl_3$); ^{1}H NMR (600 MHz, $CDCl_3$) δ 7.57 (t, *J* = 7.8 Hz, 1H), 7.50 (d, *J* = 7.8 Hz, 2H), 7.42 (dd, *J* = 7.8, 7.8 Hz, 2H), 7.37-7.31 (m, 3H), 7.24 (t, *J* = 7.8 Hz, 1H), 7.18 (dd, *J* = 7.8, 7.8 Hz, 2H), 7.10-7.00 (m, 4H), 4.51 (dd, *J* = 11.4, 3.6 Hz, 1H), 3.28 (s, 3H), 3.27-3.23 (m, 1H), 2.52 (ddd, *J* = 16.2, 10.2, 3.6 Hz, 1H), 2.27 (ddd, *J* = 16.2, 11.4, 1.8 Hz, 1H), 1.88 (dsept, *J* = 6.6, 1.8 Hz, 1H), 0.92 (d, *J* = 6.6 Hz, 3H), 0.82 (d, *J* = 6.6 Hz, 3H); ^{13}C NMR (150 MHz, $CDCl_3$) δ 171.6, 142.7, 138.8, 133.8, 133.3, 132.1, 129.8, 129.0, 128.9, 128.1, 127.9, 127.6, 127.3, 64.8, 47.0, 37.9, 27.5, 26.2, 21.1, 15.9; IR (ATR): 2964, 2932, 1652, 1595, 1496, 1302, 1144, 789, 736, 692 cm^{-1}; HRMS (FD+) Calcd for $C_{26}H_{29}NO_3S_2$ [M] 467.1589, Found 467.1587; mp. 116.0-118.0 °C.

The absolute configuration of the major diastereomer was assigned as (2*S*,4*S*) by analogy.

(2*S*,4*S*)-4-Fluoro-*N*-methyl-*N*-phenyl-4-(phenylsulfonyl)-2-(phenylthio)butanamide (3al):

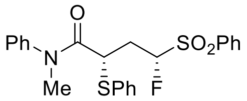

Purification with silica gel column chromatography (hexane/AcOEt = 3:1) followed by gel permeation chromatography; 39 mg, 88% yield, dr = 91:9, diastereomers are not separable by silica gel column chromatography; Colorless oil; HPLC analysis DAICEL Chiralcel AD-3 4.6×250 mm (hexane/*i*PrOH= 90/10, 1.0 mL/min, 254 nm, 30 °C) 37.1 (major of major diastereomer), 45.7 (minor of major diastereomer) min; 94% ee; Optical rotation $[\alpha]_D^{24.6}$ = −86.4 (*c* 0.145, $CHCl_3$); ^{1}H NMR for major diastereomer (600 MHz, $CDCl_3$) δ 7.88 (d, *J* = 7.8 Hz, 2H), 7.70 (tt, *J* = 7.8, 1.2 Hz, 1H), 7.58 (dd, *J* = 7.8, 7.8 Hz, 2H), 7.40-7.34 (m, 3H), 7.28 (tt, *J* = 7.8, 1.2 Hz, 1H), 7.22 (dd, *J* = 7.8, 7.8 Hz, 2H), 7.16 (d, *J* = 7.2 Hz, 2H), 7.13 (dd, *J* = 7.8, 1.2 Hz, 2H), 5.24 (ddd, *J* = 49.2, 10.2, 4.2 Hz, 1H), 3.83 (dd, *J* = 10.2, 4.2 Hz, 1H), 3.28 (s, 3H), 2.81 (dddd, *J* = 35.4, 13.8, 10.2, 4.2 Hz, 1H), 2.24 (dddd, *J* = 25.8, 13.8, 10.2, 4.2 Hz, 1H); ^{13}C NMR for major diastereomer (150 MHz, $CDCl_3$) δ 168.9, 142.6, 135.3, 134.6, 133.8, 131.4, 129.8, 129.5, 129.3, 129.0, 128.6, 128.3, 127.6, 100.2 (d, *J* = 215.4 Hz), 43.2 (d, *J* = 2.9 Hz), 37.8, 30.6 (d, *J* = 18.6 Hz); ^{19}F NMR for major diastereomer (565 MHz, $CDCl_3$) δ −180.1IR (ATR): 3407, 3062, 2937, 1654, 1595, 1496, 1387, 1330, 1310, 1155; HRMS (ESI) Calcd for $C_{23}H_{22}ClNO_3S_2$ $[M+Na]^+$ 466.0917, Found 466.0917.

The absolute configuration of the major diastereomer was assigned as (2*S*,4*S*) by analogy.

(2*S*,4*R*)-4-Chloro-*N*-methyl-*N*-phenyl-4-(phenylsulfonyl)-2-(phenylthio)butanamide (4am):

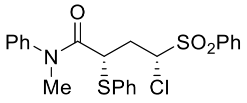

Purification with silica gel column chromatography (hexane/AcOEt = 2:1); 43 mg, 94% yield, dr = 96:4, diastereomers are not separable by silica gel column chromatography; Pale yellow oil; SFC analysis DAICEL Chiralpak IF-3/SFC 4.6×150 mm (CO_2/MeOH = 85/15, 3.0 mL/min, 254 nm, 40 °C) 5.6 (minor of major diastereomer), 6.4 (major of major diastereomer) min; 91% ee; Optical rotation $[\alpha]_D^{21}$ = −50.8 (*c* 0.130, $CHCl_3$); ^{1}H NMR for major diastereomer (600 MHz, $CDCl_3$) δ 7.88 (dd, *J* = 7.8, 1.2 Hz, 2H), 7.69 (t, *J* = 7.8, 7.8 Hz, 1H), 7.56 (dd, *J* = 7.8, 7.8 Hz, 2H), 7.40-7.33 (m, 3H), 7.29-7.25 (m, 3H), 7.21 (dd, *J* = 7.8, 7.2 Hz, 2H), 7.08 (dd, *J* = 7.8, 1.2 Hz, 2H), 4.80 (dd, *J* = 10.2, 4.2 Hz, 1H), 3.86 (dd, *J* = 10.2, 4.2 Hz, 1H), 3.28 (s, 3H), 2.99 (ddd, *J* = 13.8, 10.2, 4.2 Hz, 1H), 2.11 (ddd, *J* = 13.8, 10.2, 4.2 Hz, 1H); ^{13}C NMR for major diastereomer (150 MHz, $CDCl_3$) δ 168.5, 142.4, 135.1, 134.5, 133.2, 131.5, 129.8, 129.6, 129.1, 129.0, 128.4, 128.3, 127.7, 72.2, 44.3, 37.9, 33.8; IR (ATR): 3430, 3061, 3017, 1652, 1584, 1496, 1387, 1330, 1311, 1152 cm^{-1}; HRMS (ESI) Calcd for $C_{23}H_{22}ClNO_3S_2$ $[M+Na]^+$ 482.0622, Found 482.0622.

The absolute configuration of the major diastereomer was assigned as (2*S*,4*R*) by analogy.

(2*S*,4*R*)-4-Bromo-*N*-methyl-*N*-phenyl-4-(phenylsulfonyl)-2-(phenylthio)butanamide (4an):

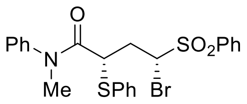

Purification with silica gel column chromatography (hexane/AcOEt = 3:1 to 2:1) followed by preparative HPLC; 45 mg, 89% yield, dr = 89:11, diastereomers are not separable by silica gel column chromatography; Colorless oil; SFC analysis DAICEL Chiralpak IF-3/SFC 4.6×150 mm (CO_2/MeOH = 85/15, 3.0 mL/min, 254 nm, 40 °C) 6.5 (minor of major diastereomer), 7.4 (major of major diastereomer) min; 91% ee; Optical rotation $[\alpha]_D^{21}$ = −11.9 (*c* 0.22, $CHCl_3$); ^{1}H NMR for major diastereomer (600 MHz, $CDCl_3$) δ 7.88 (dd, *J* = 7.8, 1.2 Hz, 2H), 7.68 (tt, *J* = 7.8, 1.2 Hz, 1H), 7.56 (dd, *J* = 7.8, 7,8 Hz, 2H), 7.41-7.30 (m, 5H), 7.25 (t, *J* = 7.8 Hz, 1H), 7.19 (dd, *J* = 7.8, 7.8 Hz, 2H), 7.07-7.04 (m, 2H), 4.85 (dd, *J* = 10.8, 4.2 Hz, 1H), 3.85 (dd, *J* = 10.8, 4.2 Hz, 1H), 3.27 (s, 3H), 2.92 (ddd, *J* = 13.8, 10.8, 4.2 Hz, 1H), 2.15 (ddd, *J* = 13.8, 10.8, 4.2 Hz, 1H); ^{13}C NMR for major diastereomer (150 MHz, $CDCl_3$) δ 168.4, 142.4, 135.1, 134.5, 133.1, 131.5, 129.7, 129.6, 129.1, 129.0, 128.4, 128.3, 127.8, 63.3, 45.3, 37.9, 34.8; IR (ATR): 3408, 3061, 3020, 1651, 1595, 1496, 1386, 1328, 1151, 1084 cm^{-1}; HRMS (ESI) Calcd for $C_{23}H_{22}BrNO_3S_2$ $[M+Na]^+$ 526.0117, Found 526.0117.

The absolute configuration of the major diastereomer was assigned as (2*S*,4*R*) by analogy.

The 1,3-*syn* configuration of adducts derived from **3n** was confirmed by the single-crystal X-ray diffraction analysis of the racemic **10gn** obtained by the oxidation of **4gn** using *m*CPBA.

(2*S*,4*R*)-4-Bromo-2-((4-bromophenyl)thio)-1-morpholino-4-(phenylsulfonyl)butan-1-one (4gn):

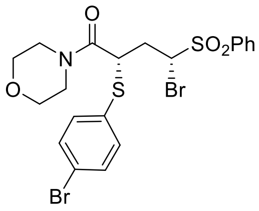

Purification with silica gel column chromatography (hexane/AcOEt = 3:2) diastereomers are not separable by silica gel column chromatography; 48 mg, 85% yield, dr = 92:8; White solid; HPLC analysis DAICEL Chiralcel OD-3 4.6×250 mm (hexane/*i*PrOH= 90/10, 1.0 mL/min, 254 nm, 30 °C) 28.0 (minor of major diastereomer), 35.5 (major of major diastereomer) min; 91% ee; Optical rotation $[\alpha]_D^{24.6}$ = 40.5 (*c* 0.115, $CHCl_3$); ^{1}H NMR for major diastereomer (600 MHz, $CDCl_3$) δ 7.92 (d, *J* = 7.2 Hz, 2H), 7.71 (t, *J* = 7.2 Hz, 1H), 7.58 (dd, *J* = 7.2, 7.2 Hz, 2H), 7.45 (d, *J* = 7.8 Hz, 2H), 7.25 (d, *J* = 7.8 Hz, 2H), 4.94 (dd, *J* = 10.8, 4.2 Hz, 1H), 4.09 (dd, *J* = 10.8, 4.2 Hz, 1H), 3.70-3.61 (m, 5H), 3.56-3.51 (m, 2H), 3.49-3.44 (m, 1H), 2.81 (ddd, *J* = 13.8, 10.2, 4.2 Hz, 1H), 2.33 (ddd, *J* = 13.8, 10.2, 4.2 Hz, 1H); ^{13}C NMR for major diastereomer (150 MHz, $CDCl_3$) δ 166.6, 136.0, 135.1, 134.7, 132.4, 129.7, 129.22, 129.16, 124.0. 66.8, 66.4, 63.5, 46.4, 43.6, 42.5, 33.9; IR (ATR): 2966, 2923, 2856, 1643, 1446, 1327, 1152, 1115, 1010, 756 cm^{-1}; HRMS (FD+) Calcd for $C_{20}H_{21}Br_2NO_4S_2$ [M] 560.9279, Found 560.9278; mp. 105.0-107.0 °C.

(2*S,4*R**)-4-Bromo-2-((4-bromophenyl)sulfonyl)-1-morpholino-4-(phenylsulfonyl)butan-1-one (10gn):**

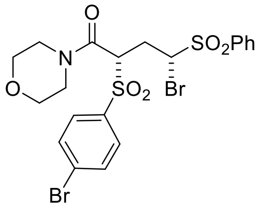

Purification with silica gel column chromatography (hexane/AcOEt = 1:1); Obtained as a single diastereomer; White solid; ^{1}H NMR (600 MHz, $CDCl_3$) δ 7.90 (d, *J* = 7.2 Hz, 2H), 7.73 (t, *J* = 7.2 Hz, 1H), 7.71 (d, *J* = 8.4 Hz, 2H), 7.62 (d, *J* = 8.4 Hz, 2H), 7.59 (dd, *J* = 7.8, 7.8 Hz, 2H), 4.75 (dd, *J* = 10.2, 4.2 Hz, 1H), 4.72 (dd, *J* = 10.2, 4.2 Hz, 1H), 3.84-3.64 (m, 6H), 3.61-3.55 (m, 1H), 3.53-3.47 (m, 1H), 2.75 (ddd, *J* = 13.8, 10.2, 4.2 Hz,

1H), 2.57 (ddd, *J* = 13.8, 10.2, 4.2 Hz, 1H); ^{13}C NMR (150 MHz, $CDCl_3$) δ 161.2, 134.9, 134.6, 134.2, 132.5, 131.1, 130.6, 129.8, 129.3, 66.5, 66.4, 63.5, 61.7, 47.4, 43.2, 29.9; IR (ATR): 2930, 2859, 1649, 1574, 1447, 1326, 1153, 1117, 1083, 747 cm^{-1}; HRMS (FD+) Calcd for $C_{20}H_{21}Br_2NO_6S_2$ [M] 592.9177, Found 592.9174; mp. 131.0-133.0 °C.

ORTEP drawing of (2*S**,4*R**)-**10gn** showing thermal elipsoids at the 50% probability level. CCDC No. 2291617. Recrystallization from hexane/2-butanone.

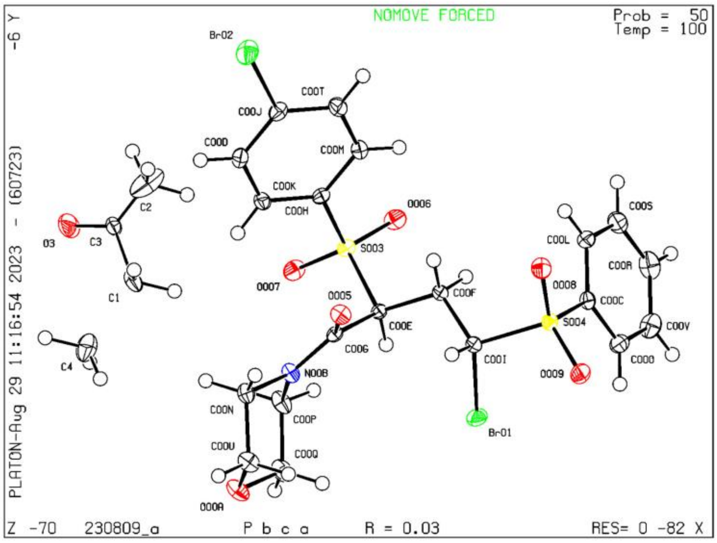

(2*S*,4*S*)-4-(Ethylsulfonyl)-*N*-methyl-*N*,4-diphenyl-2-(phenylthio)butanamide (4ao):

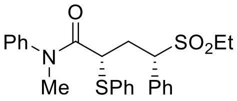

Purification with silica gel column chromatography (hexane/AcOEt = 2:1 to 1:1); 45 mg, 99% yield, dr = 99:1, diastereomers are not separable by silica gel column chromatography; Pale yellow sticky oil; SFC analysis DAICEL Chiralpak IC-3/SFC 4.6×150 mm (CO_2/MeOH = 80/20, 3.0 mL/min, 254 nm, 40 °C) 6.8 (major of major diastereomer), 14.0 (minor of major diastereomer) min; 96% ee; Optical rotation $[\alpha]_D^{19.6}$ = −112.0 (*c* 0.20, $CHCl_3$); ^{1}H NMR for major diastereomer (600 MHz, $CDCl_3$) δ 7.43 (t, *J* = 7.8 Hz, 1H), 7.35 (dd, *J* = 7.8, 7.2 Hz, 2H), 7.24 (t, *J* = 7.8 Hz, 1H), 7.20 (t, *J* = 7.8 Hz, 1H), 7.19-7.15 (m, 4H), 7.11 (br, 2H), 6.89 (d, *J* = 7.8 Hz, 2H), 6.83-6.40 (brs, 2H), 4.27 (dd, *J* = 11.4, 4.8 Hz, 1H), 3.45 (dd, *J* = 10.8, 4.2 Hz, 1H), 3.23 (s, 3H), 2.96 (ddd, *J* = 13.8, 10.8, 4.8 Hz, 1H), 2.87 (q, *J* = 7.8 Hz, 2H), 2.41 (ddd, *J* = 13.8, 10.8, 4.8 Hz, 1H), 1.33 (t, *J* = 7.8 Hz, 3H); ^{13}C NMR for major diastereomer (150 MHz, $CDCl_3$) δ 168.8, 142.2, 132.9, 132.1, 130.9, 129.9, 129.3, 129.14, 129.08, 128.9, 128.1, 127.8, 127.4, 64.0, 45.4, 45.2, 37.7, 30.6, 6.3; IR (ATR): 3060, 3038, 2979, 2941, 2883, 1654, 1595, 1496, 1454, 1439, 1419, 1385, 1320, 1305, 1274, 1234, 1131, 1025, 843, 796, 753, 700 cm^{-1}; HRMS (ESI) Calcd for $C_{25}H_{27}NO_3S_2$ $[M+Na]^+$ 476.1325, Found 476.1325.

The absolute configuration of the major diastereomer was assigned as (2*S*,4*S*) by analogy.

***tert*-Butyl (2*R*,4*S*)-5-(methyl(phenyl)amino)-5-oxo-2-phenyl-4-(phenylthio)pentanoate (6aa):**

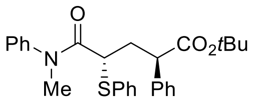

Purification with silica gel column chromatography (hexane/AcOEt = 4:1), 33 mg, 71% yield, dr = 91:9, diastereomers are not separable by silica gel column chromatography; Colorless sticky oil; SFC analysis DAICEL Chiralpak IF-3/SFC 4.6×150 mm (CO_2/MeOH = 90/10, 3.0 mL/min, 220 nm, 40 °C) 4.1 (minor of major diastereomer), 5.3 (major of major diastereomer) min; 97% ee; Optical rotation $[\alpha]_D^{21}$ = −54.6 (*c* 0.165, $CHCl_3$); ^1H NMR for major diastereomer (600 MHz, $CDCl_3$) δ 7.25-7.13 (m, 11H), 7.12-7.09 (m, 2H), 6.86 (d, *J* = 7.2 Hz, 2H), 3.64 (dd, *J* = 7.8, 7.8 Hz, 1H), 3.49 (dd, *J* = 7.8, 7.8 Hz, 1H), 3.23 (s, 3H), 2.46-2.40 (m, 1H), 2.39-2.33 (m, 1H), 1.30 (s, 9H); ^{13}C NMR for major diastereomer (150 MHz, $CDCl_3$) δ 172.2, 170.7, 142.8, 138.5, 133.19, 133.17, 129.5, 128.8, 128.4, 127.83, 127.77, 127.6, 127.2, 127.0, 80.8, 50.1, 46.7, 37.7, 36.0, 27.8; IR (ATR): 3415, 3005, 2979, 1722, 1657, 1496, 1383, 1369, 1220, 1147 cm^{-1}; HRMS (ESI) Calcd for $C_{28}H_{31}NO_3S$ $[M+Na]^+$ 484.1917, Found 484.1917.

The absolute configuration of major diastereomer of **4aa** was determined to be (2*R*,4*S*) by single-crystal X-ray diffraction analysis of **9aa**, which was prepared by the oxidation of **4aa** using *m*CPBA (vide infra).

***tert*-Butyl (2*R*,4*S*)-2-(4-methoxyphenyl)-5-(methyl(phenyl)amino)-5-oxo-4-(phenylthio)pentanoate (6ab):**

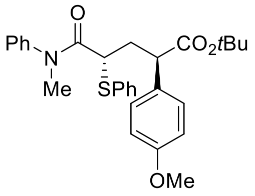

Purification with silica gel column chromatography (hexane/AcOEt = 4:1); 39 mg, 73% yield, dr = 85:15, diastereomers are not separable by silica gel column chromatography; Pale yellow oil; SFC analysis DAICEL Chiralpak IF-3/SFC 4.6×150 mm (CO_2/MeOH = 90/10, 3.0 mL/min, 254 nm, 40 °C) 4.9 (minor of major diastereomer), 5.8 (major of major diastereomer) min; 97% ee; Optical rotation $[\alpha]_D^{18.7}$ = −82.9 (*c* 0.105, $CHCl_3$); ^1H NMR for major diastereomer (600 MHz, $CDCl_3$) δ 7.25-7.11 (m, 8H), 7.05 (d, *J* = 9.0 Hz, 2H), 6.84 (d, *J* = 7.2 Hz, 2H), 6.74 (d, *J* = 9.0 Hz, 2H), 3.79 (s, 3H), 3.59 (t, *J* = 7.2 Hz, 1H), 3.47 (t, *J* = 7.2 Hz, 1H), 3.22 (s, 3H), 2.40 (ddd, *J* = 13.8, 7.2, 7.2 Hz, 1H), 2.32 (ddd, *J* = 13.8, 7.2, 7.2 Hz, 1H), 1.30 (s, 9H); ^{13}C NMR for major diastereomer (150 MHz, $CDCl_3$) δ 172.5, 170.8, 158.5, 142.8, 133.2, 133.1, 130.5, 129.4, 128.8, 128.7, 127.8, 127.5, 127.2, 113.7, 80.7, 55.2, 49.2, 46.6, 37.7, 36.0, 27.8; IR (ATR): 2976, 2933, 1720, 1655, 1595, 1511, 1496, 1368, 1250, 1145 cm^{-1}; HRMS (FD+) Calcd for $C_{29}H_{33}NO_4S$ [M] 491.2130, Found 491.2129.

The absolute configuration of the major diastereomer was assigned as (2*R*,4*S*) by analogy.

***tert*-Butyl (2*R*,4*S*)-2-(4-bromophenyl)-5-(methyl(phenyl)amino)-5-oxo-4-(phenylthio)pentanoate (6ac):**

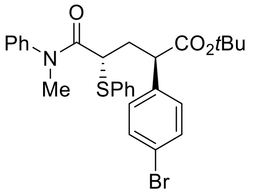

Purification with silica gel column chromatography (hexane/AcOEt = 4:1); 54 mg, 99% yield, dr = 89:11, diastereomers are not separable by silica gel column chromatography; Pale yellow oil; SFC analysis DAICEL Chiralpak IF-3/SFC 4.6×150 mm (CO_2/MeOH = 90/10, 3.0 mL/min, 220 nm, 40 °C) 5.5 (minor of major diastereomer), 6.6 (major of major diastereomer) min; 97% ee; Optical rotation $[\alpha]_D^{18.3}$ = −59.0 (*c* 0.175, $CHCl_3$); ^1H NMR for major diastereomer (600 MHz, $CDCl_3$) δ 7.31 (d, *J* = 7.8 Hz, 2H), 7.26-7.23 (m, 2H), 7.20 (dd, *J* = 7.8, 7.8 Hz, 2H), 7.18 (dd, *J* = 7.8, 7.2 Hz, 2H), 7.14 (d, *J* = 7.2 Hz, 2H), 6.99 (d, *J* = 8.4 Hz, 2H), 6.81 (d, *J* = 7.2 Hz, 2H), 3.62 (dd, *J* = 7.8, 7.2 Hz, 1H), 3.40 (dd, *J* = 7.8, 6.0 Hz, 1H), 3.22 (s, 3H), 2.40 (ddd, *J* = 14.4, 7.8, 7.2 Hz, 1H), 2.26 (ddd, *J* = 14.4, 7.8, 6.6 Hz, 1H), 1.31 (s, 9H); ^{13}C NMR for major diastereomer (150 MHz, $CDCl_3$) δ 171.7, 170.6, 142.7, 137.4, 133.4, 133.0, 132.8, 131.5, 129.5 (2C), 128.8, 128.0, 127.7, 127.1, 121.0, 81.1, 49.3, 46.4, 37.7, 35.9,

27.8; IR (ATR): 3060, 2977, 2933, 1723, 1656, 1595, 1495, 1368, 1146, 699 cm^{-1}; HRMS (FD+) Calcd for $C_{28}H_{30}BrNO_3S$ [M]$^+$ 539.1130, Found 539.1128.

The absolute configuration of the major diastereomer was assigned as (2*R*,4*S*) by analogy.

2-4. Procedure for Derivatization of Adducts 4 and 6

*Reduction of **4aa** to **7aa** (Scheme 7a)*

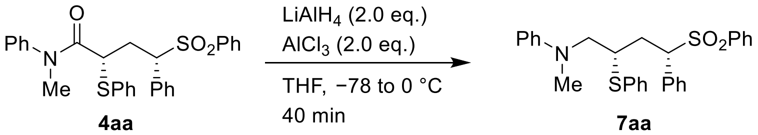

To a solution of $LiAlH_4$ (7.6 mg, 0.20 mmol) in THF (1.0 mL) was added $AlCl_3$ (27 mg, 0.20 mmol) and **4aa** (50 mg, 0.10 mmol, dr = 94:6, 92% ee) at −78 °C. The reaction mixture was warmed to 0 °C and stirred at that temperature for 40 min. The reaction was quenched with sat. aq. Rochelle salt and stirred vigorously for 15 min. The product was extracted with AcOEt, and the combined organic layer was dried over Na_2SO_4, and concentrated under reduced pressure. The residue was purified by silica gel column chromatography (hexane/AcOEt = 4:1) to afford **7aa** (48 mg, 0.098 mmol, 98%, dr = 95:5, 91% ee) as a colorless oil.

(2*S*,4*S*)-*N*-Methyl-*N*-(4-phenyl-4-(phenylsulfonyl)-2-(phenylthio)butyl)aniline (7aa):

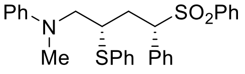

Purification with silica gel column chromatography (hexane/AcOEt = 4:1); 48 mg, 98% yield, dr = 95:5, diastereomers are not separable by silica gel column chromatography; Colorless oil; HPLC analysis DAICEL Chiralcel OD-3 4.6×250 mm (hexane/*i*PrOH= 94/6, 1.0 mL/min, 254 nm, 30 °C) 13.3 (major of major diastereomer), 40.2 (minor of major diastereomer) min; 91% ee; Optical rotation $[\alpha]_D^{24.6}$ = −36.8 (*c* 0.110, $CHCl_3$); ^1H NMR for major diastereomer (600 MHz, $CDCl_3$) δ 7.47 (t, *J* = 7.8 Hz, 1H), 7.33 (d, *J* = 7.2 Hz, 2H), 7.31-7.22 (m, 8H), 7.17 (dd, *J* = 7.2, 7.2 Hz, 2H), 7.13 (dd, *J* = 7.8, 7.8 Hz, 2H), 6.78 (d, *J* = 7.2 Hz, 2H), 6.71 (t, *J* = 7.2 Hz, 1H), 6.53 (d, *J* = 7.8 Hz, 2H), 4.45 (dd, *J* = 7.8, 6.0 Hz, 1H), 3.56-3.51 (m, 1H), 3.47-3.41 (m, 2H), 2.87 (s, 3H), 2.79 (ddd, *J* = 15.0, 7.2, 6.0 Hz, 1H), 2.34 (ddd, *J* = 15.0, 8.4, 6.0 Hz, 1H); ^{13}C NMR for major diastereomer (150 MHz, $CDCl_3$) δ 148.7, 136.8, 133.4, 133.21, 133.17, 132.4, 129.9, 129.3, 129.0 (2C), 128.7, 128.5, 128.3, 127.7, 116.8, 112.2, 68.6, 57.9, 45.2, 39.5, 32.5; IR (ATR): 3060, 2928, 1600, 1505, 1446, 1306, 1146, 1085, 749, 692 cm^{-1}; HRMS (FD+) Calcd for $C_{29}H_{29}NO_2S_2$ [M] 487.1640, Found 487.1639.

*Reduction of **6aa** to **8aa** (Scheme 7b)*

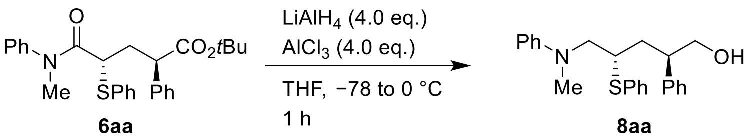

To a solution of $LiAlH_4$ (15 mg, 0.40 mmol) in THF (1.0 mL) was added $AlCl_3$ (53 mg, 0.40 mmol) and **6aa** (46 mg, 0.10 mmol, single diastereomer, 97% ee) at −78 °C. The reaction mixture was warmed to 0 °C and stirred at that temperature for 1 h. The reaction was quenched with sat. aq. Rochelle salt and stirred vigorously for 15 min.

The product was extracted with AcOEt, and the combined organic layer was dried over Na_2SO_4, and concentrated under reduced pressure. The residue was purified by silica gel column chromatography (hexane/AcOEt = 4:1) to afford **8aa** (25 mg, 0.065 mmol, 65%, single diastereomer, 96% ee) as a colorless oil.

(2*R*,4*S*)-5-(Methyl(phenyl)amino)-2-phenyl-4-(phenylthio)pentan-1-ol (8aa)

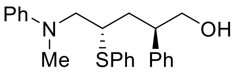

Purification with silica gel column chromatography (hexane/AcOEt = 4:1); 25 mg, 65% yield; Obtained as single diastereomer; Colorless oil; HPLC analysis DAICEL Chiralcel AD-3 4.6×250 mm (hexane/*i*PrOH= 90/10, 1.0 mL/min, 254 nm, 30 °C) 8.4 (major), 9.6 (minor) min; 96% ee; Optical rotation $[\alpha]_D^{24.6}$ = 27.7 (*c* 0.100, $CHCl_3$); ^{1}H NMR (600 MHz, $CDCl_3$) δ 7.26-7.16 (m, 8H), 7.13-7.08 (m, 4H), 6.65 (dd, *J* = 7.2, 7.2 Hz, 1H), 6.43 (d, *J* = 8.4 Hz, 2H), 3.69 (d, *J* = 6.6 Hz, 2H), 3.41-3.32 (m, 3H), 3.04-3.00 (m, 1H), 2.70 (s, 3H), 2.05 (ddd, *J* = 14.4, 12.0, 2.4 Hz, 1H), 1.75 (ddd, *J* = 14.4, 12.0, 2.4 Hz, 1H), 1.34 (brs, 1H); ^{13}C NMR (150 MHz, $CDCl_3$) δ 149.0, 140.6, 134.2, 132.7, 129.0, 128.8, 128.7, 128.2, 127.2, 127.0, 116.4, 112.1, 67.9, 58.5, 46.2, 45.3, 38.9, 33.7; IR (ATR): 3389, 3060, 3027, 2929, 2871, 1599, 1499, 1478, 746, 692 cm^{-1}; HRMS (FD+) Calcd for $C_{24}H_{27}NOS$ [M] 377.1813, Found 377.1813.

*Oxidation of **6aa** to **9aa** (Scheme 7c)*

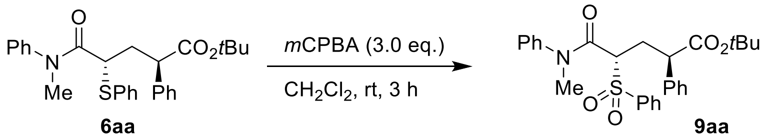

6aa **9aa**

To a solution of **6aa** (69 mg, 0.15 mmol, single diastereomer, 97% ee) in CH_2Cl_2 (2.0 mL) was added *m*CPBA (ca.70%, 0.11 g, 0.45 mmol). The reaction mixture was stirred at room temperature for 3 h. The reaction was quenched with sat. aq. $NaHCO_3$. The product was extracted with CH_2Cl_2, and the combined organic layer was dried over Na_2SO_4, and concentrated under reduced pressure. The residue was purified by silica gel column chromatography (hexane/AcOEt = 3:1) to afford **9aa** (70 mg, 0.14 mmol, 95%, single diastereomer, 97% ee) as a white solid.

***tert*-Butyl (2*R*,4*S*)-5-(methyl(phenyl)amino)-5-oxo-2-phenyl-4-(phenylsulfonyl)pentanoate (9aa):**

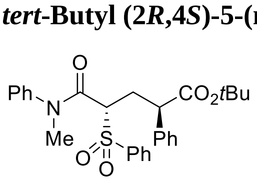

Purification with silica gel column chromatography (hexane/AcOEt = 3:1); 70 mg (0.15 mmol scale), 95% yield; Obtained as single diastereomer; White solid; SFC analysis DAICEL Chiralpak IC-3/SFC 4.6×150 mm (CO_2/MeOH = 90/10, 3.0 mL/min, 220 nm, 40 °C) 7.8 (major), 10.7 (minor) min; 97% ee; Optical rotation $[\alpha]_D^{24.6}$ = −50.1 (*c* 0.105, $CHCl_3$); ^{1}H NMR (600 MHz, $CDCl_3$) δ 7.79 (d, *J* = 7.2 Hz, 2H), 7.65 (t, *J* = 7.2 Hz, 1H), 7.53 (dd, *J* = 7.2, 7.2 Hz, 2H), 7.37-7.29 (br, 3H), 7.26-7.22 (m, 3H), 7.20-7.00 (m, 4H), 4.05 (dd, *J* = 7.8, 5.4 Hz, 1H), 3.56 (dd, *J* = 8.4, 6.6 Hz, 1H), 3.26 (s, 3H), 2.54 (ddd, *J* = 14.4, 8.4, 5.4 Hz, 1H), 2.29 (ddd, *J* = 13.8, 7.8, 6.6 Hz, 1H), 1.25 (s, 9H); ^{13}C NMR (150 MHz, $CDCl_3$) δ 171.3, 164.4, 142.3, 138.1, 137.2, 134.0, 129.9, 129.8, 128.8, 128.6, 128.1, 127.8, 127.7, 127.4, 81.2, 64.6, 49.4, 38.3, 32.3, 27.8; IR (ATR): 2979, 2937, 1725, 1661, 1596, 1496, 1385, 1310, 1149, 1082 cm^{-1}; HRMS (FD+) Calcd for $C_{28}H_{31}NO_5S$ [M] 493.1923, Found 493.1922; mp. 155.0-157.0 °C.

ORTEP drawing of (2*R*,4*S*)-**9aa** showing thermal elipsoids at the 50% probability level. CCDC No. 2272321. Recrystallization from hexane/methanol.

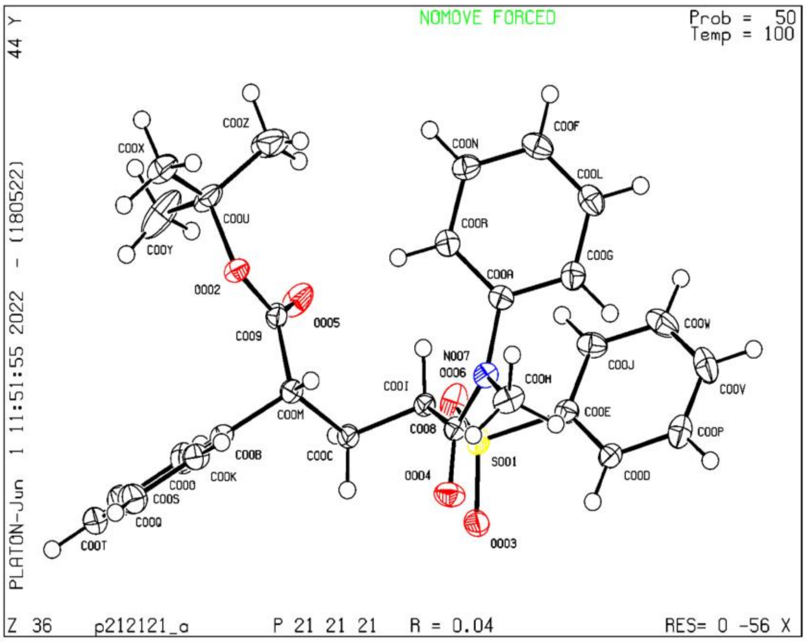

3. References

[S1] A. Kondoh, S. Ishikawa, M. Terada, *J. Am. Chem. Soc.* **2020**, *142*, 3724-3728.

[S2] F. Xiao, Y. Hu, H. Huang, F. Xu, G.-J. Deng, *Org. Biomol. Chem.* **2020**, *18*, 3527-3535.

[S3] C. D. Bray, G. Faveri, *J. Org. Chem.* **2010**, *75*, 4652-4655.

[S4] C.-N. Hsiao, H. Shechter, *J. Org. Chem.* **1988**, *53*, 2688-2699.

[S5] F. Wang, Y. Tang, X. Li, J. Chen, J. Yang, *Org. Lett.* **2022**, *24*, 7309-7314.

[S6] S. K. Mandal, A. K. Ghosh, R. Kumar, B. Zajc, *Org. Biomol. Chem.* **2012**, *10*, 3164-3167.

[S7] D. L. J. Clive, T. L. B. Boivin, A. G. Angoh, *J. Org. Chem.* **1987**, *52*, 4943-4953.

[S8] Y. Fang, M. Yuan, J. Zhang, L. Zhang, X. Jin, R. Li, J. Li, *Tetrahedron Lett.* **2016**, *57*, 1460-1463.

[S9] C. Wang, L. Zong, C.-H. Tan, *J. Am. Chem. Soc.* **2015**, *137*, 10677-10682.

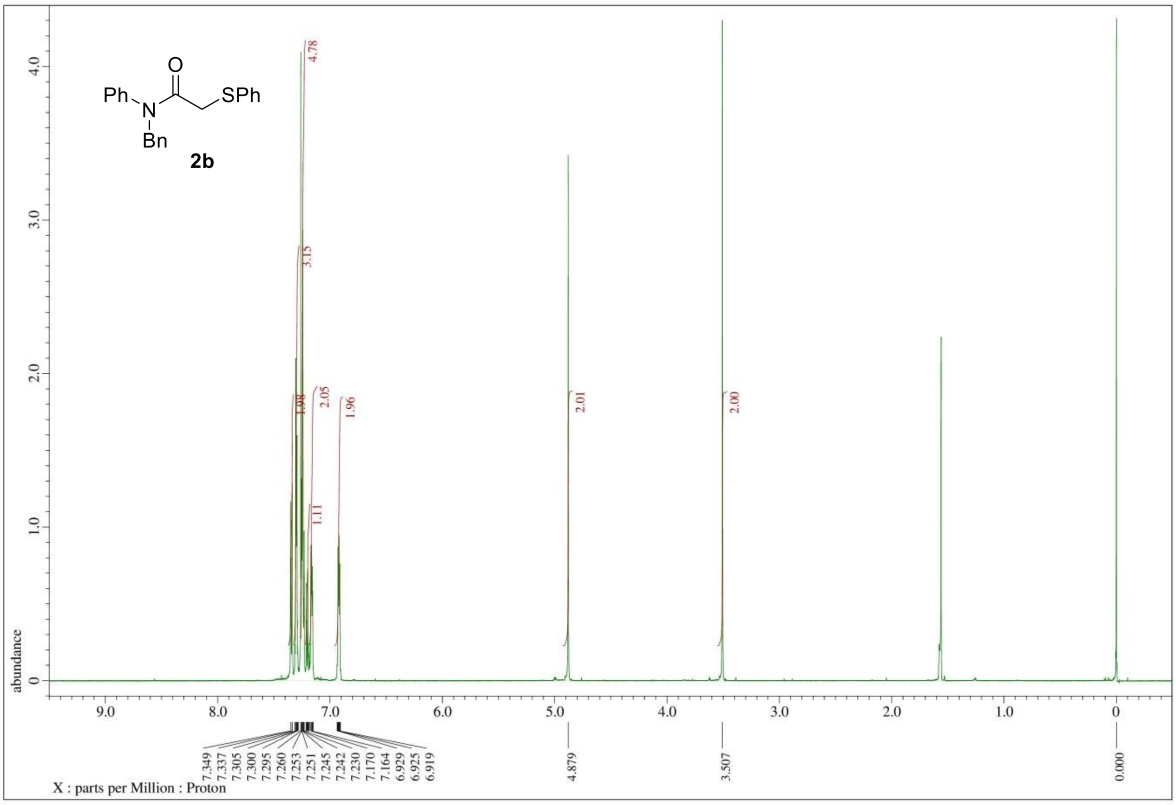

600 MHz, $CDCl_3$

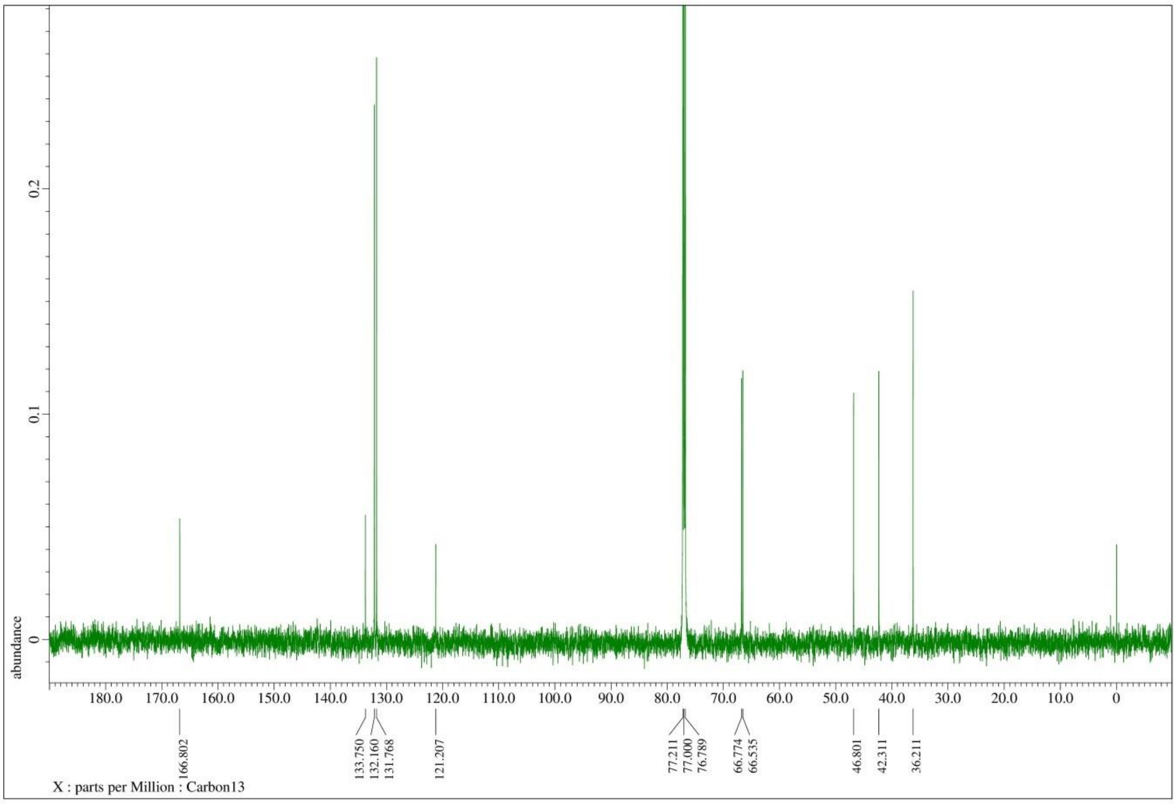

150 MHz, $CDCl_3$

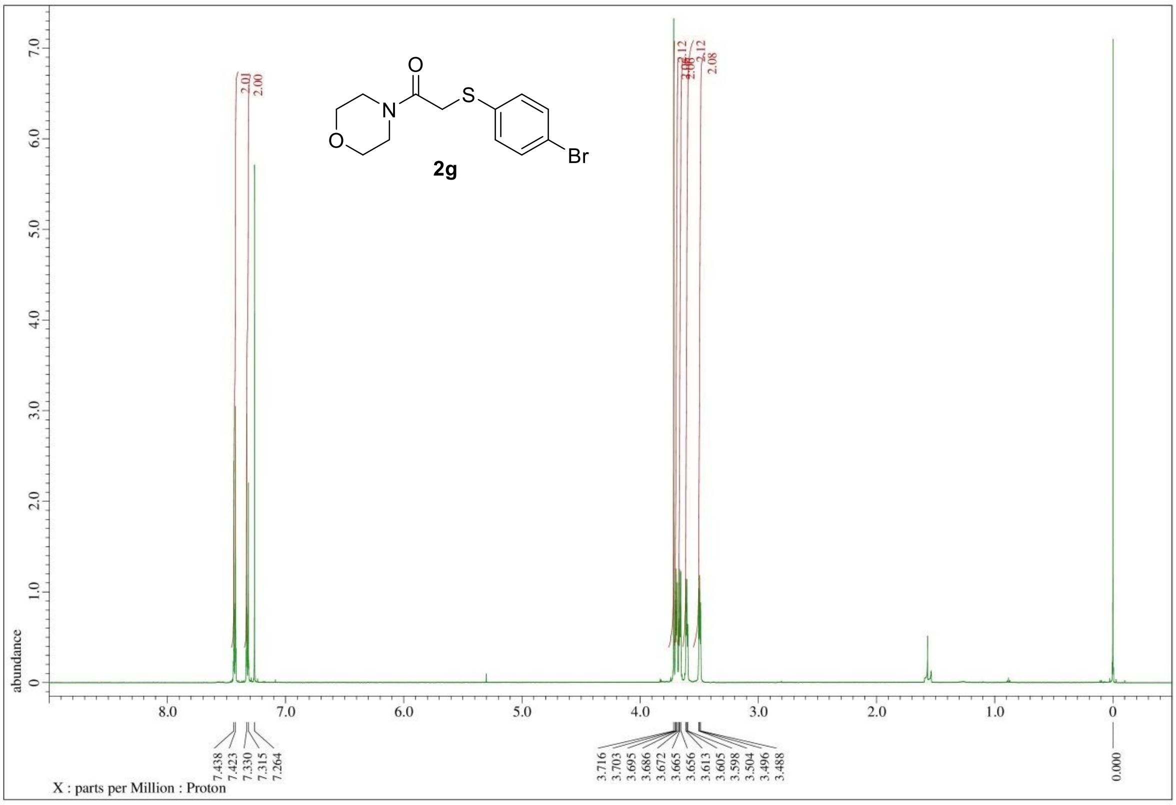

600 MHz, $CDCl_3$

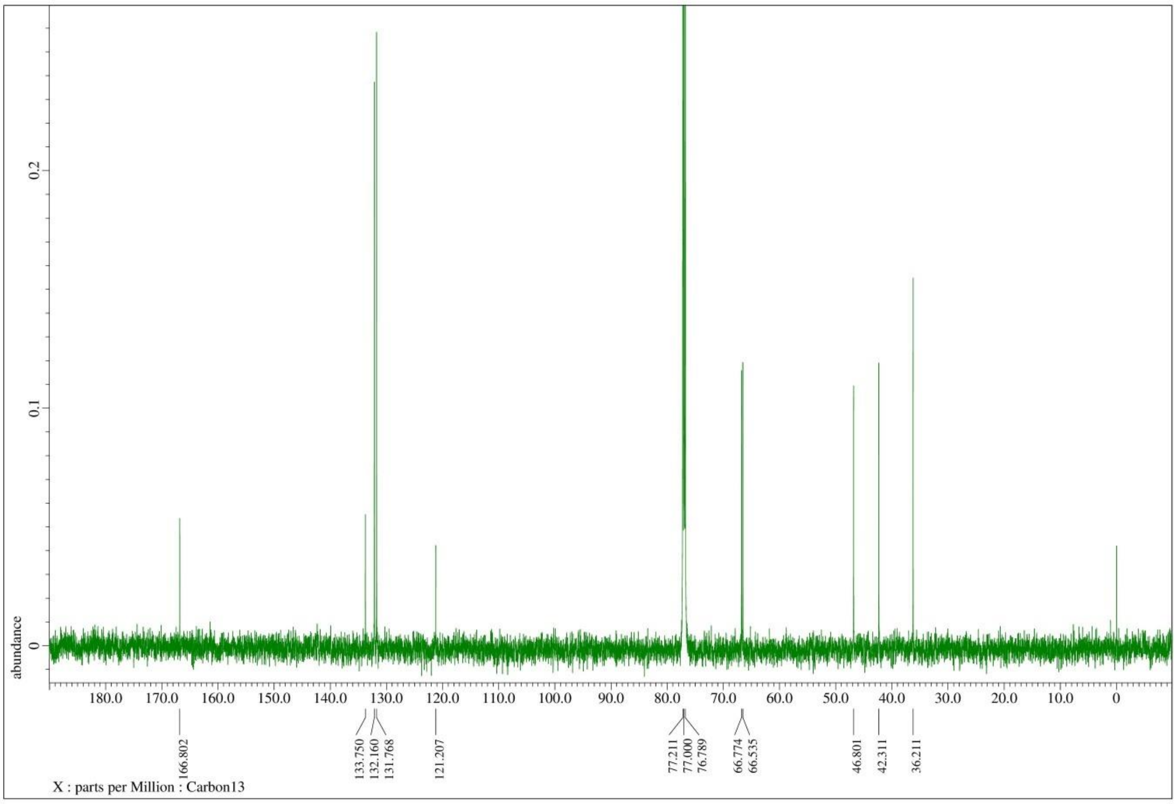

150 MHz, $CDCl_3$

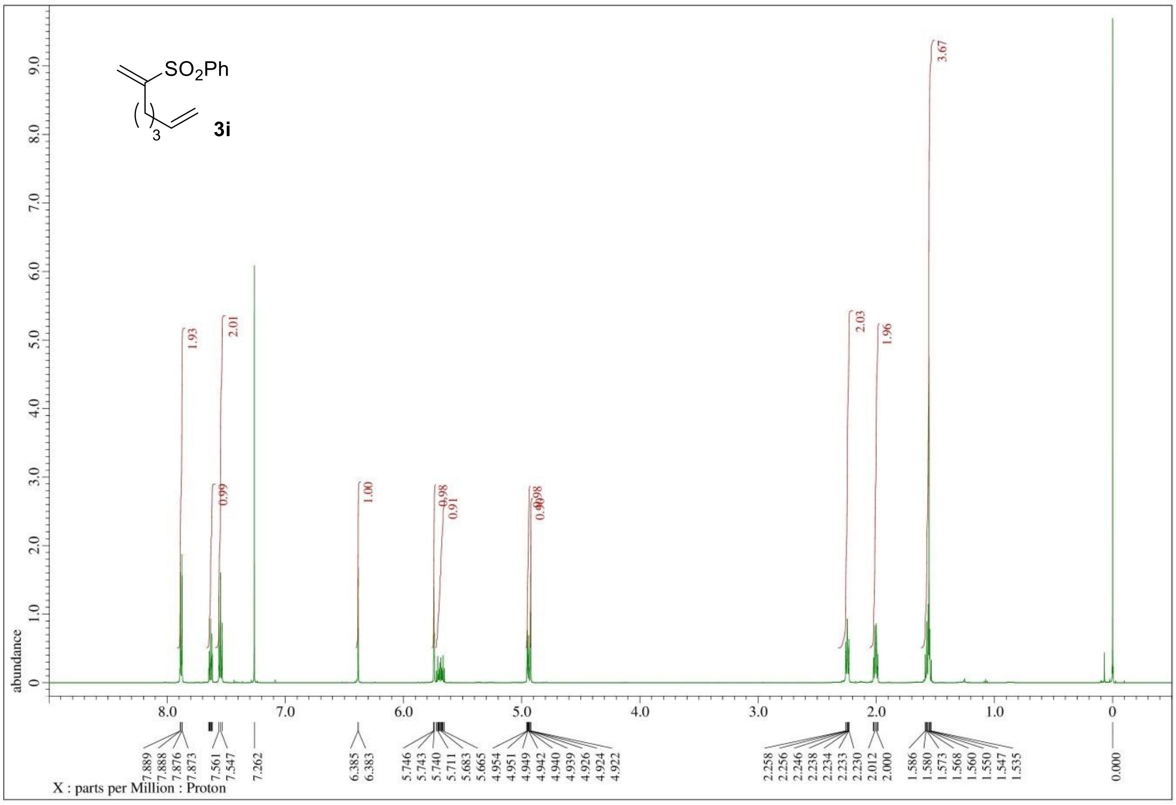

600 MHz, $CDCl_3$

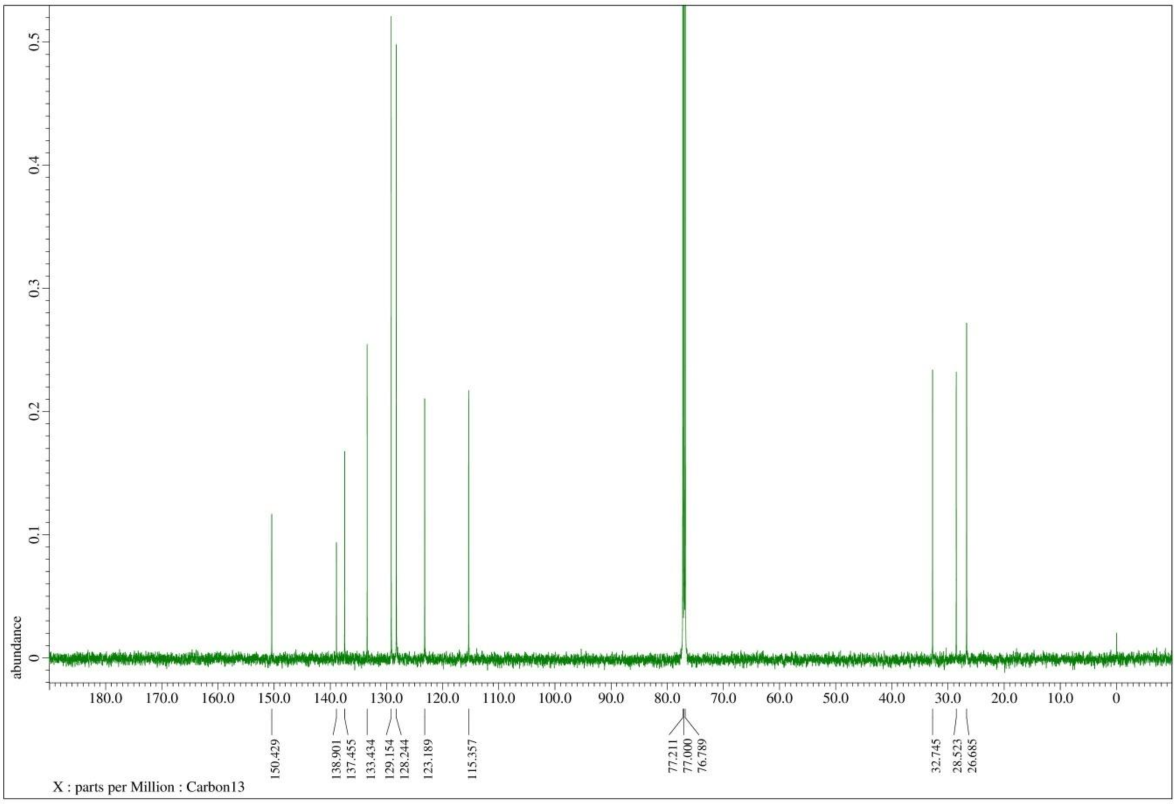

150 MHz, $CDCl_3$

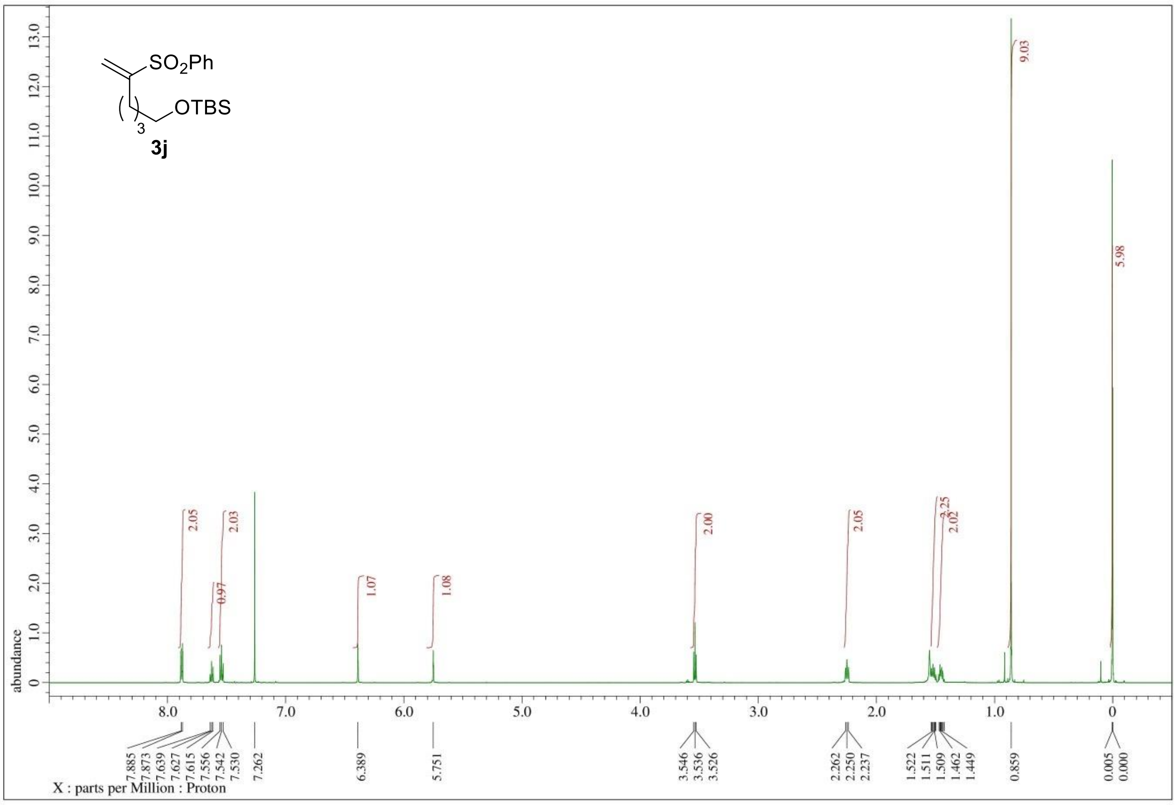

600 MHz, $CDCl_3$

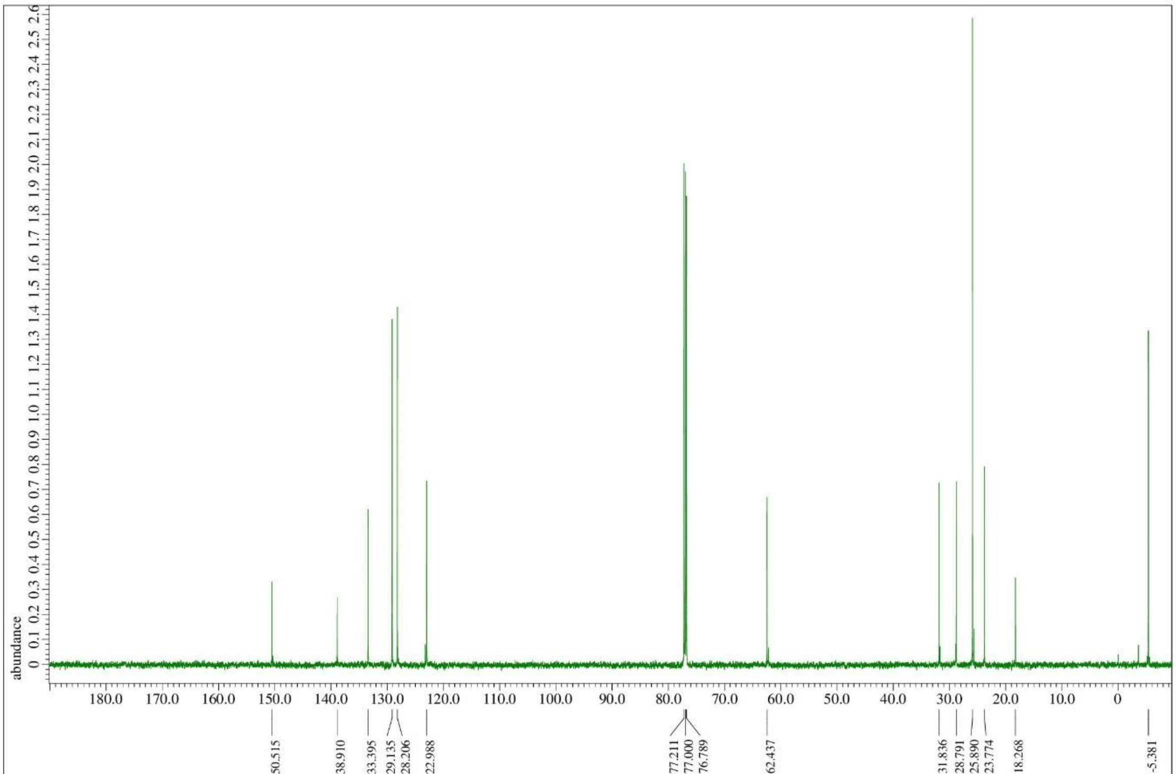

150 MHz, $CDCl_3$

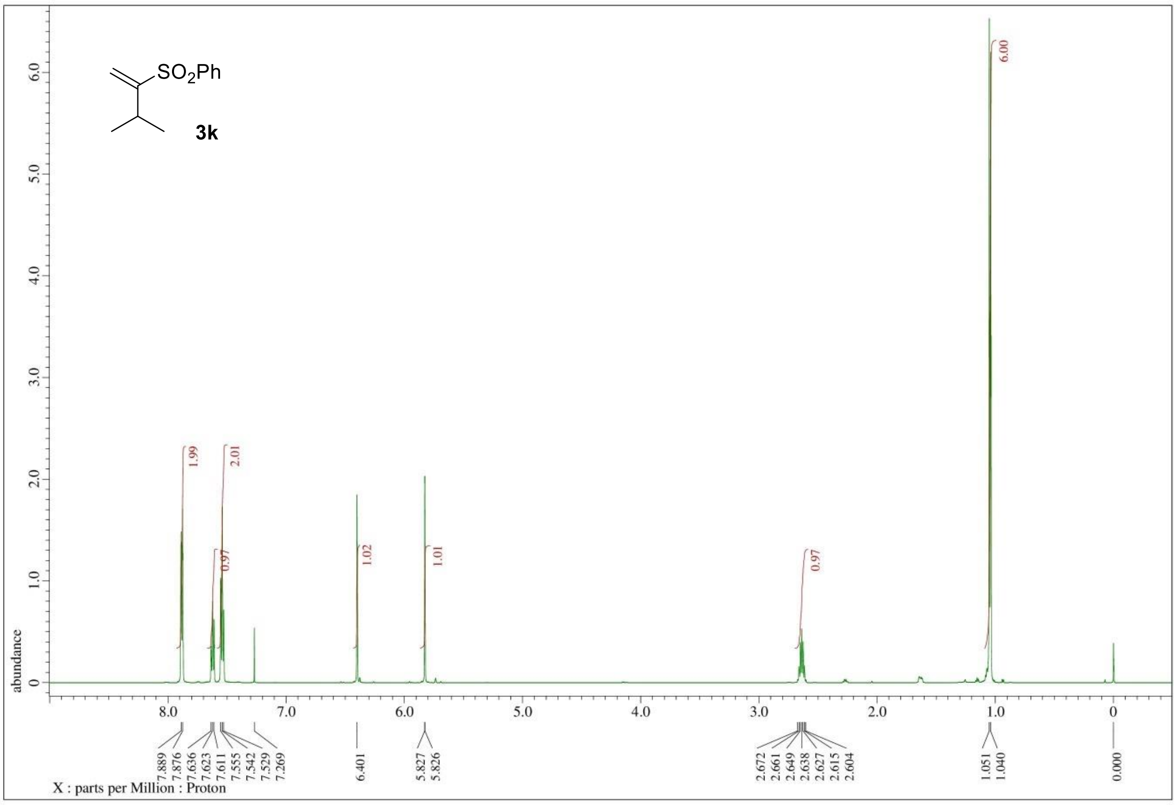

600 MHz, $CDCl_3$

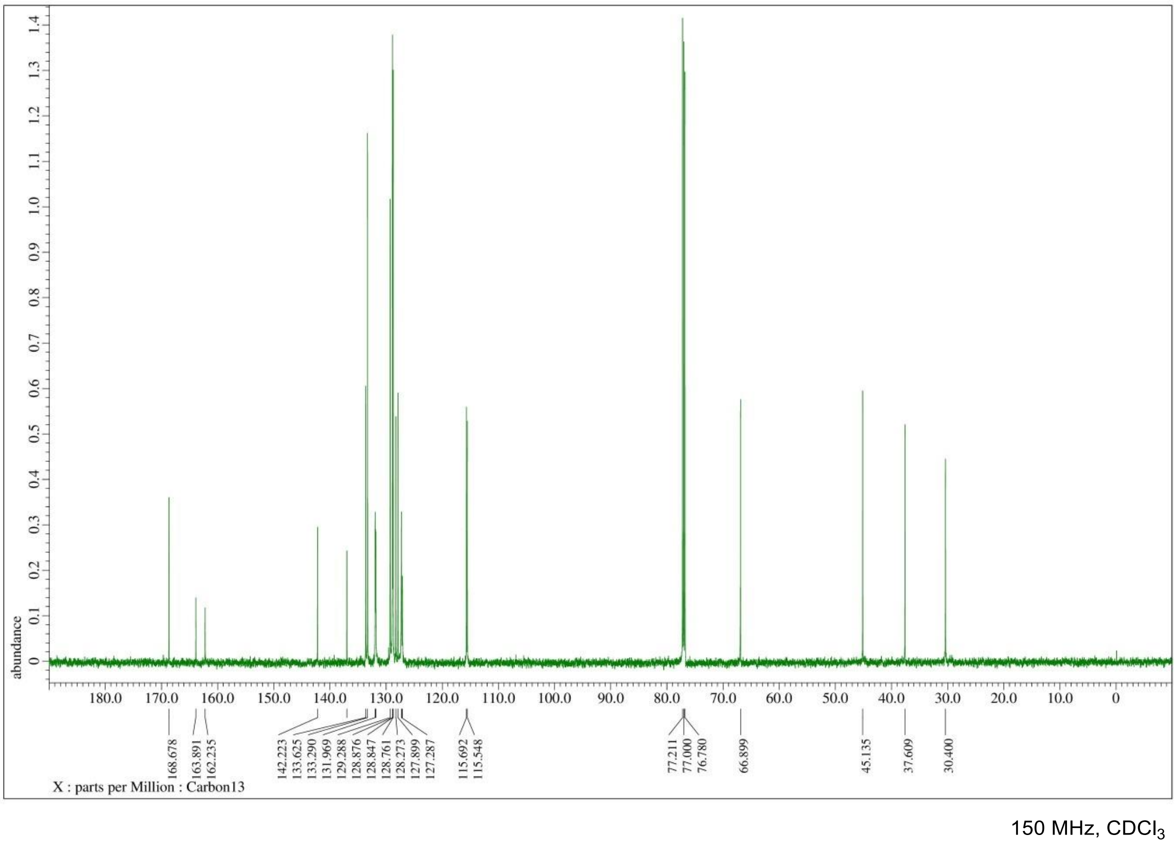

150 MHz, $CDCl_3$

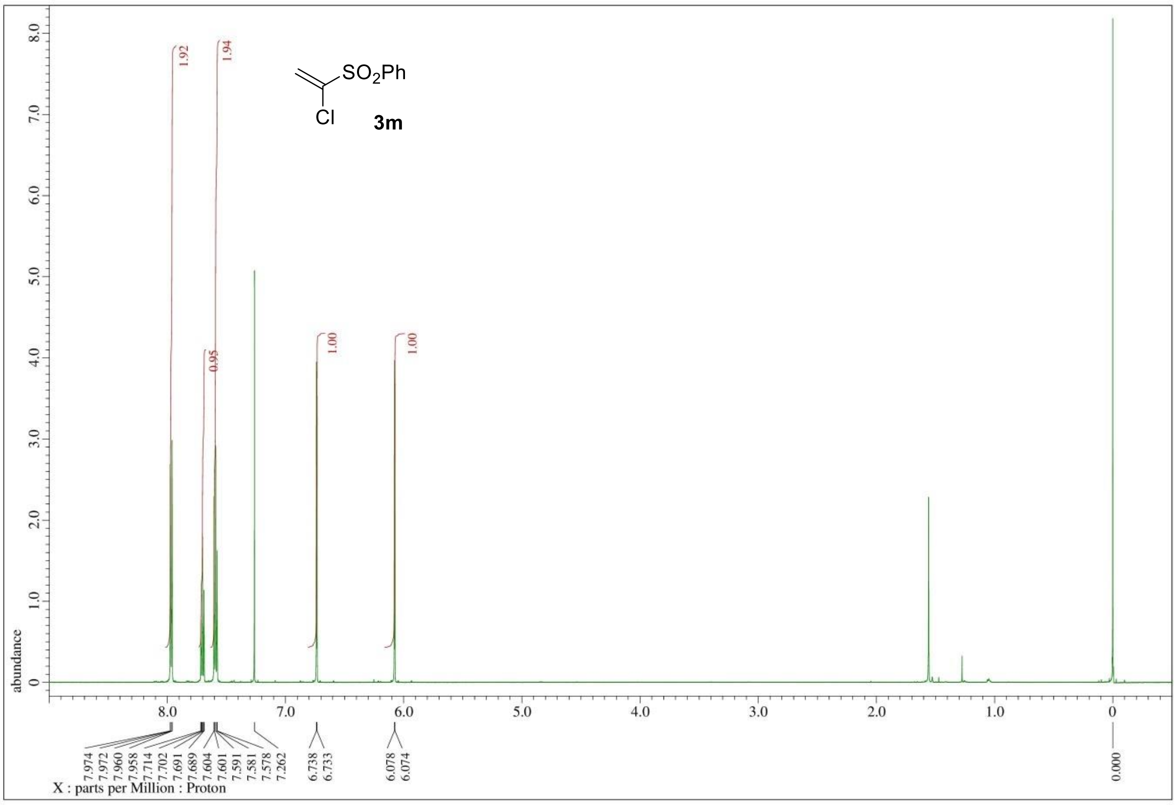

600 MHz, $CDCl_3$

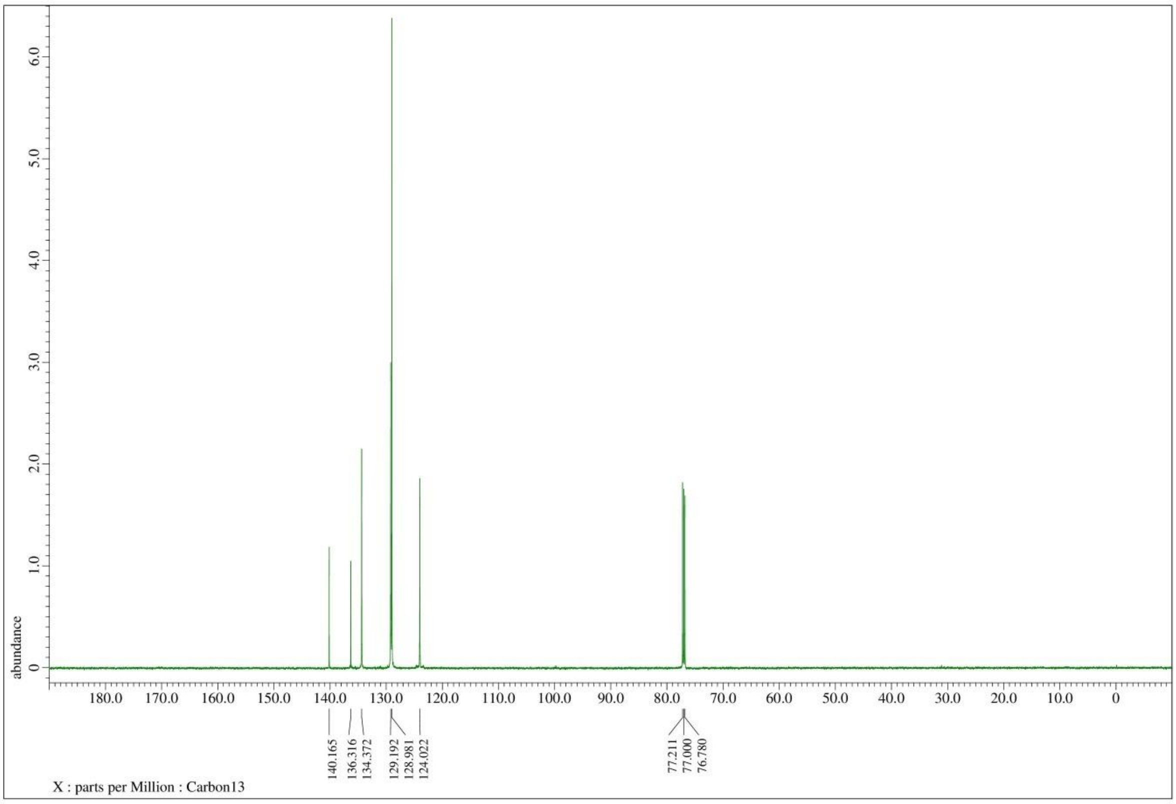

150 MHz, $CDCl_3$

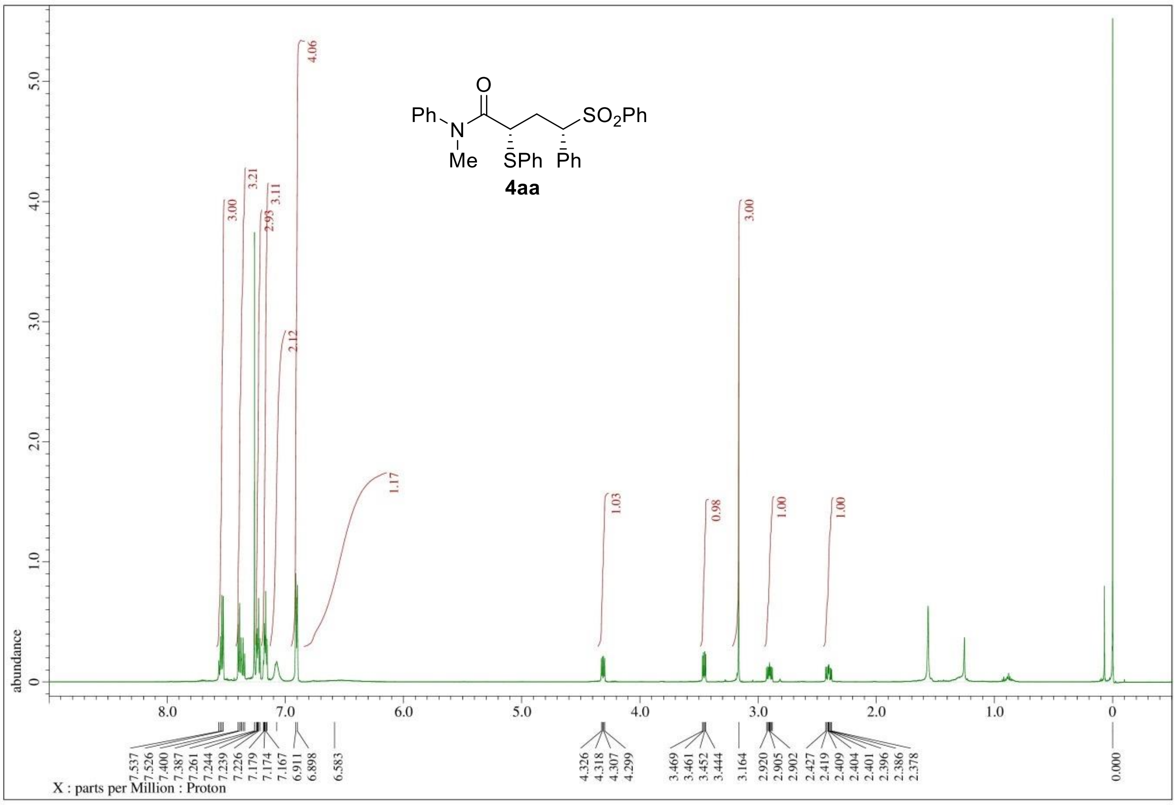

600 MHz, $CDCl_3$

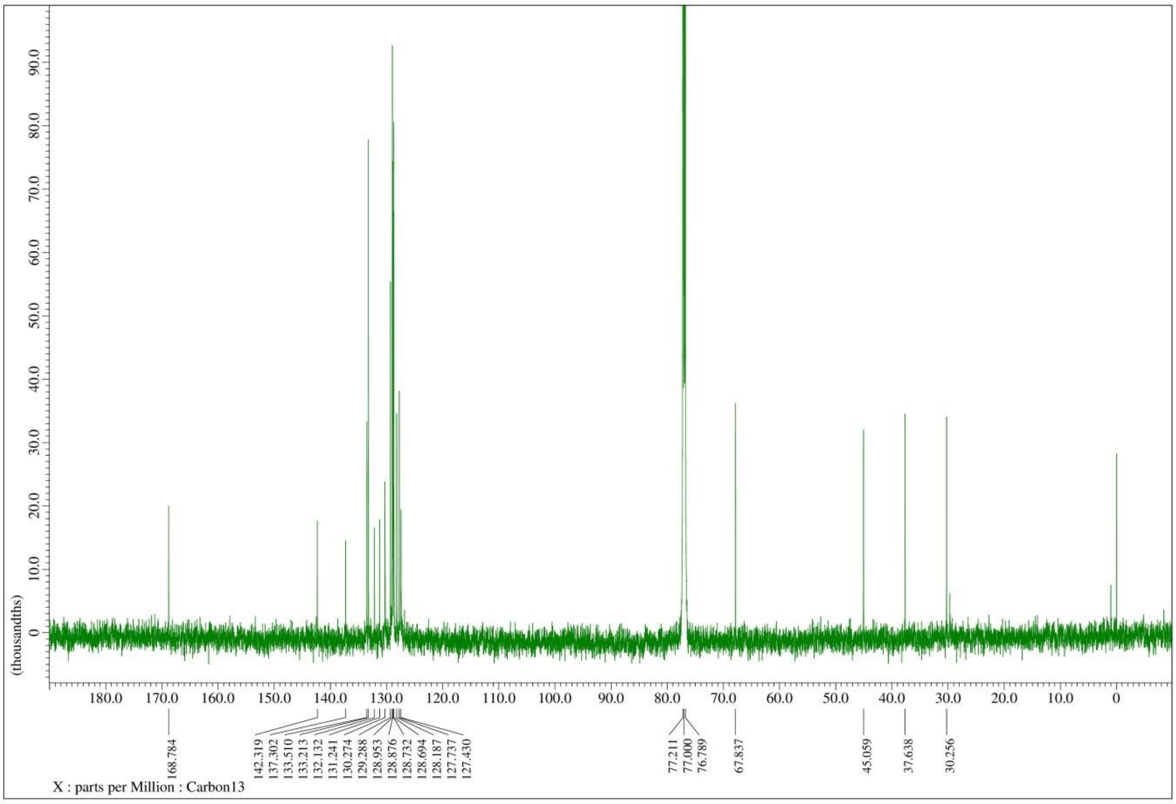

150 MHz, $CDCl_3$

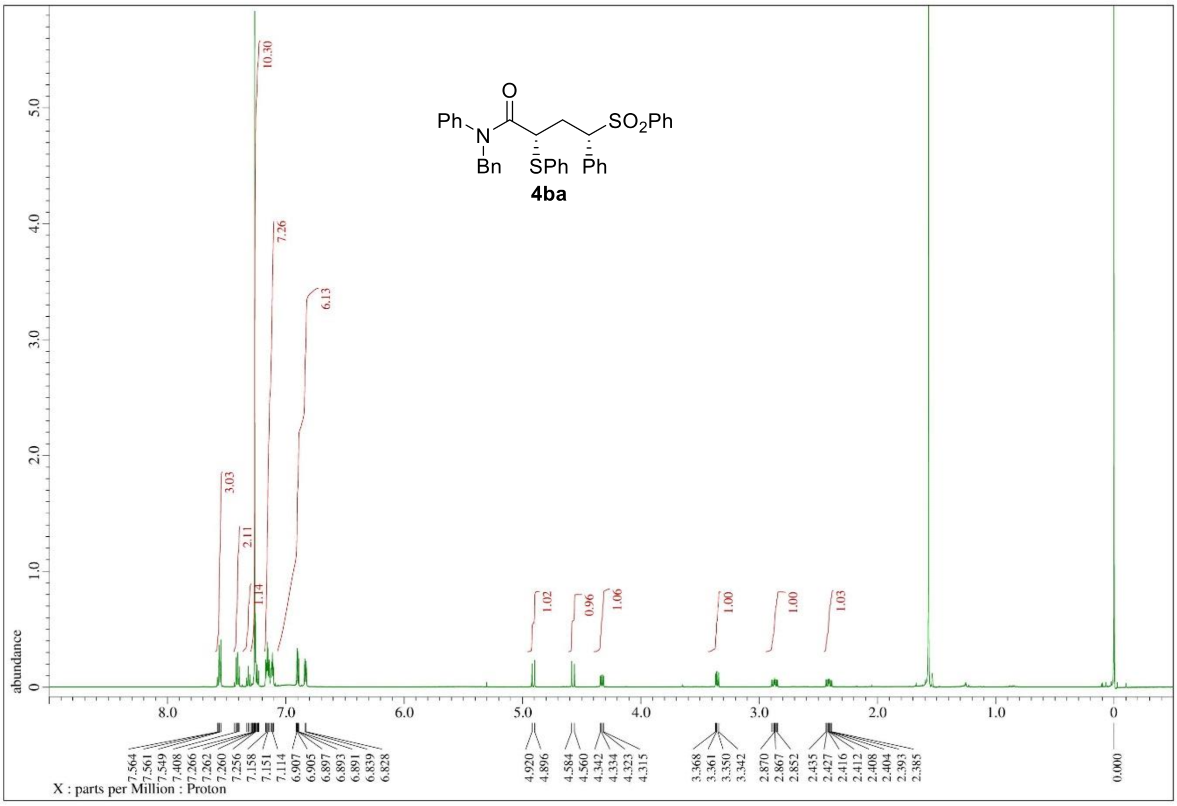

600 MHz, $CDCl_3$

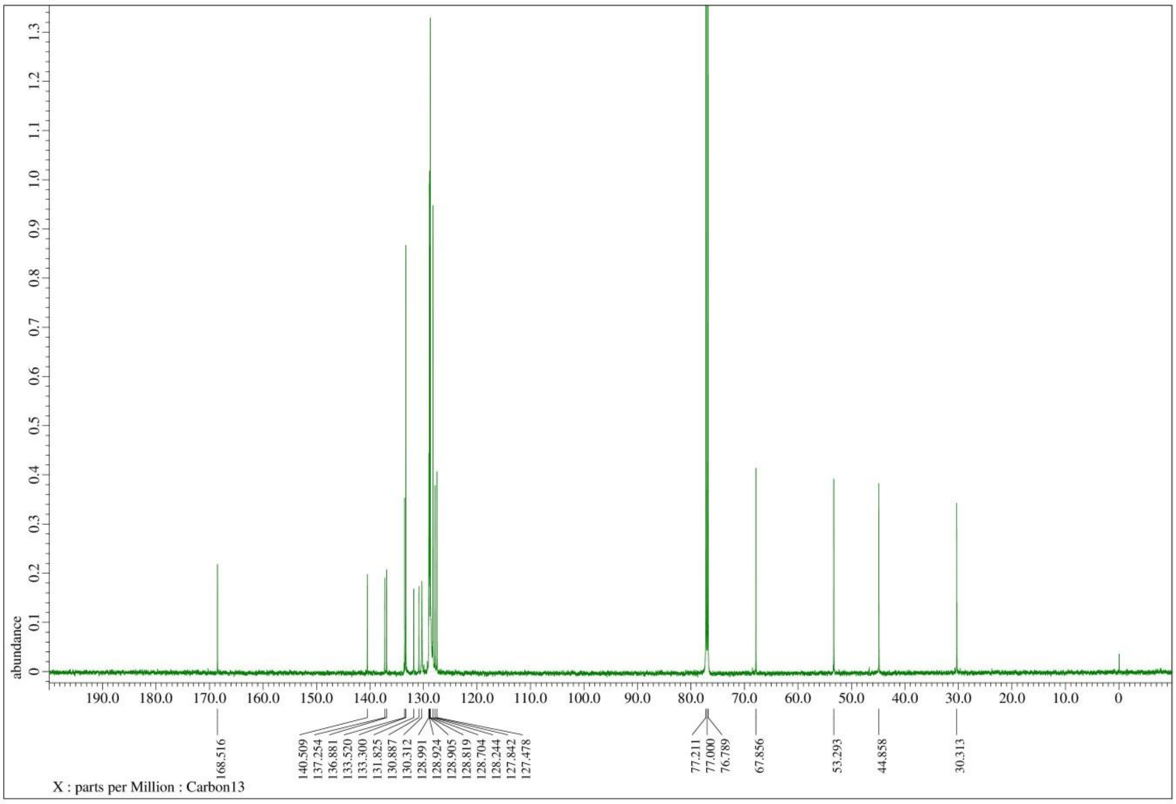

150 MHz, $CDCl_3$

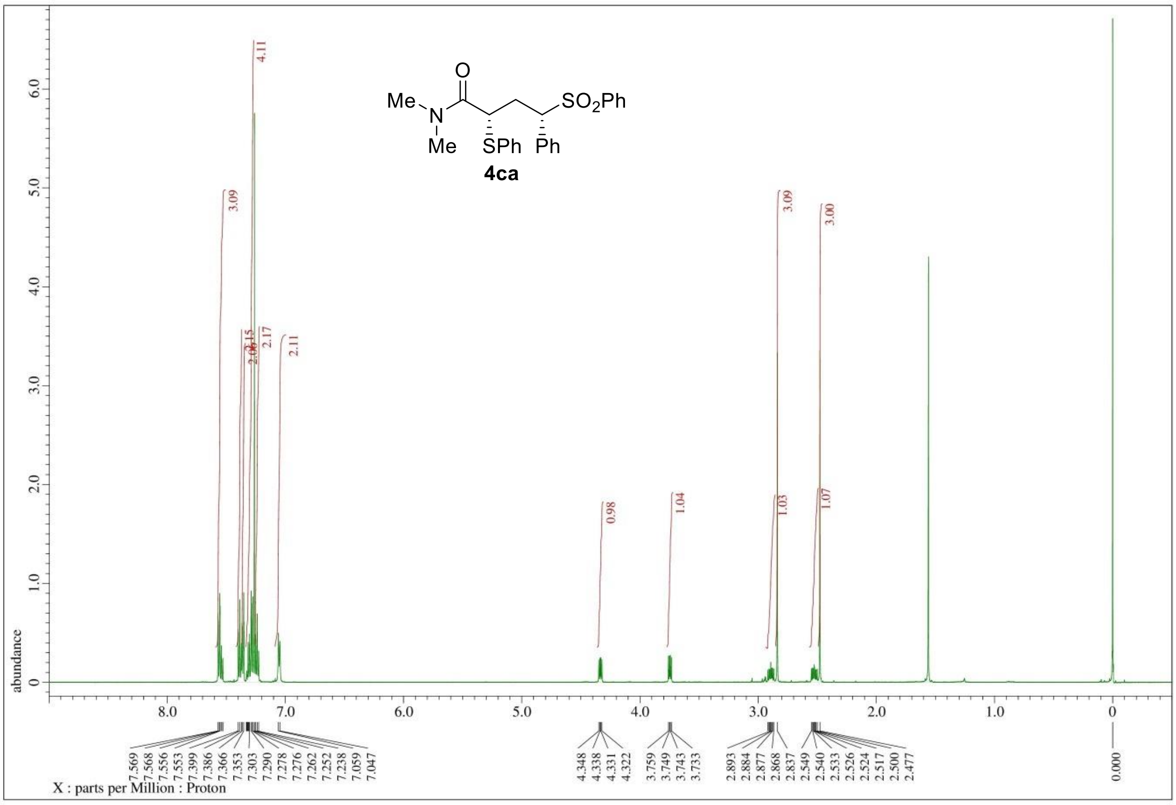

600 MHz, $CDCl_3$

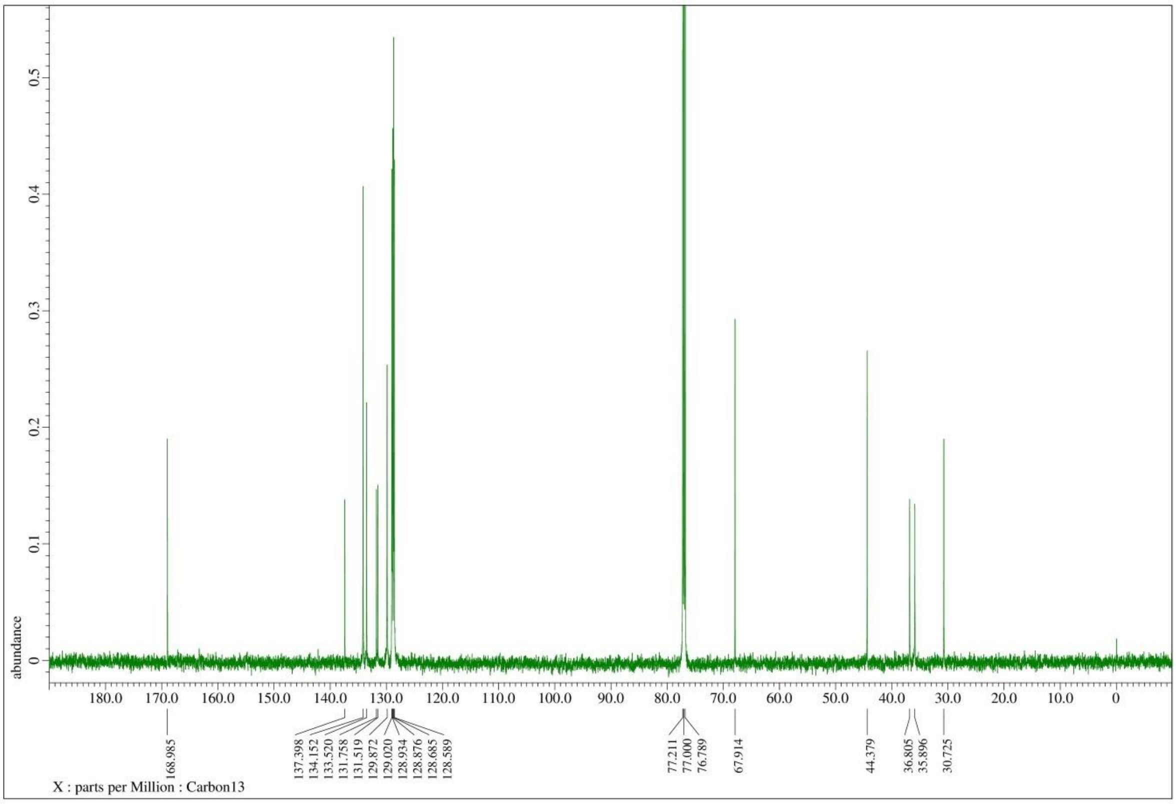

150 MHz, $CDCl_3$

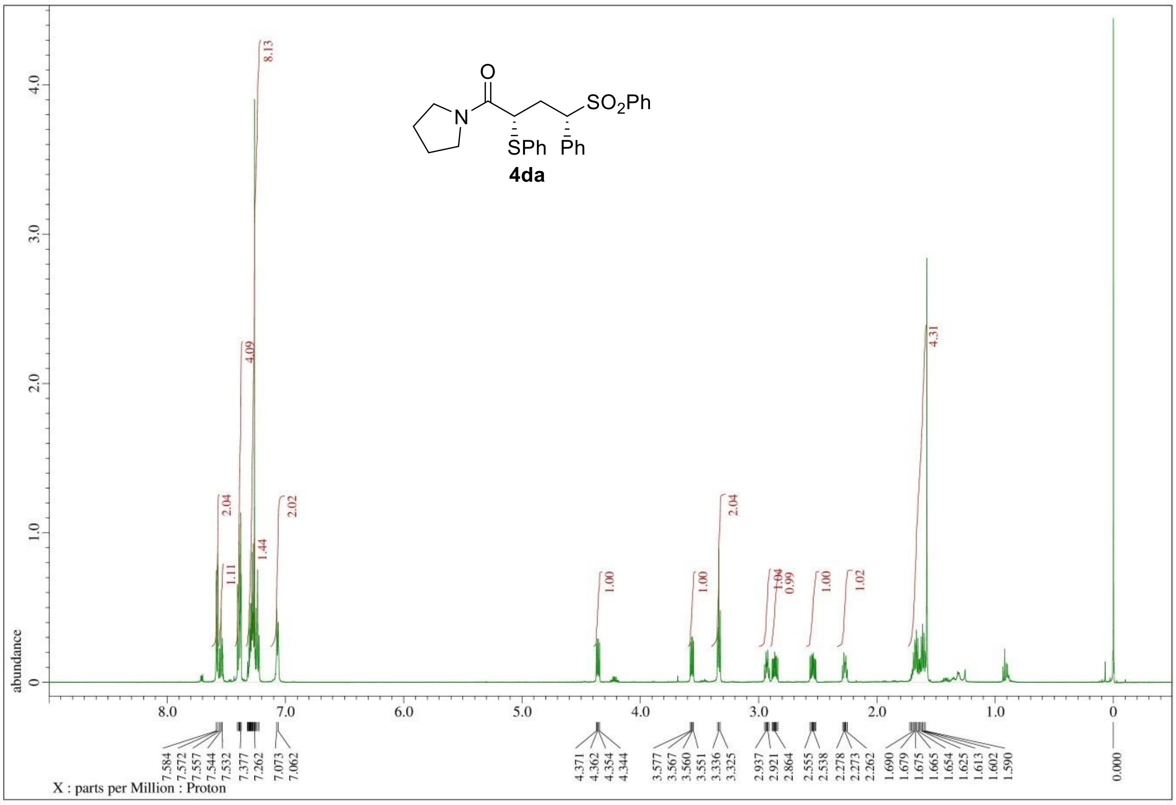

600 MHz, $CDCl_3$

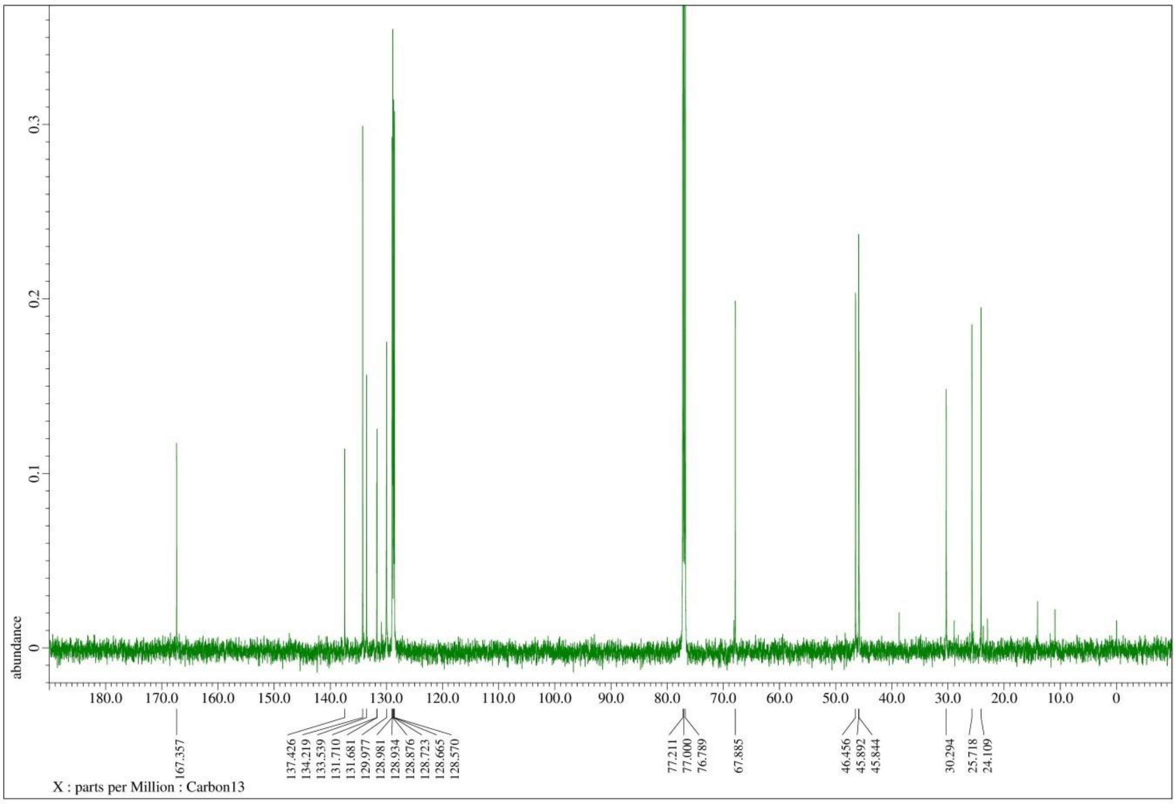

150 MHz, $CDCl_3$

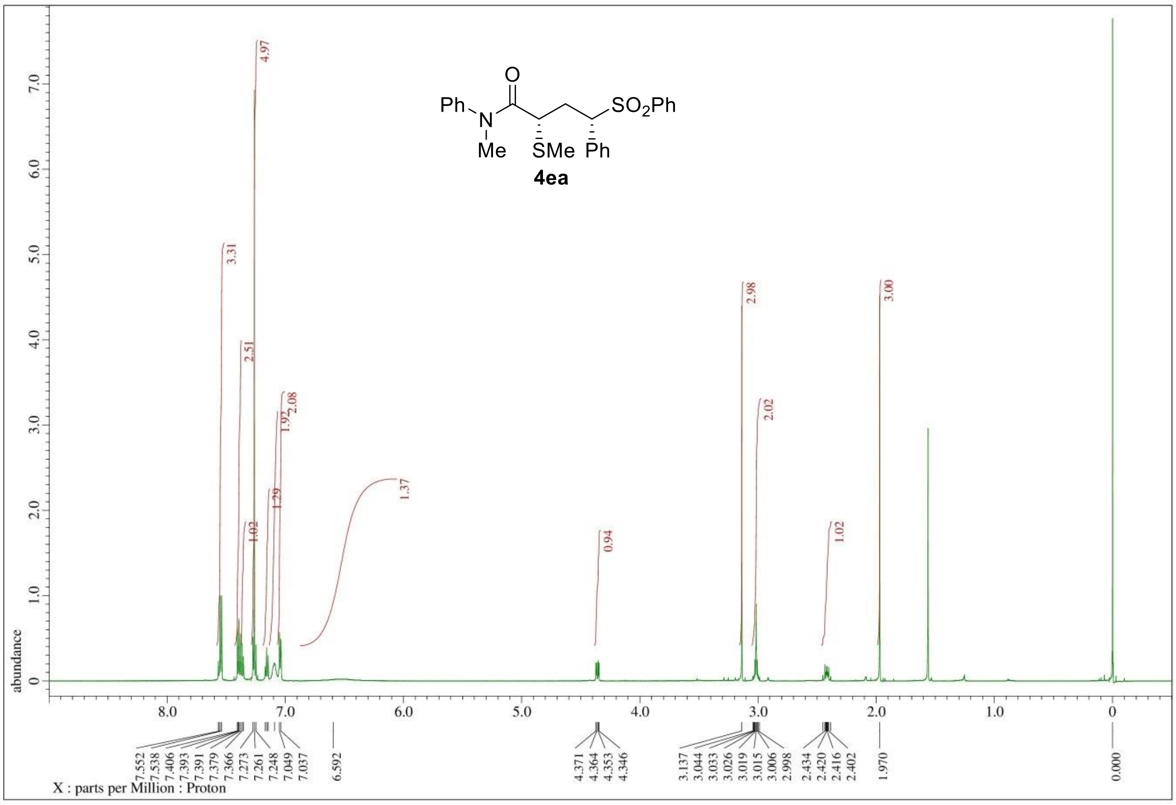

600 MHz, $CDCl_3$

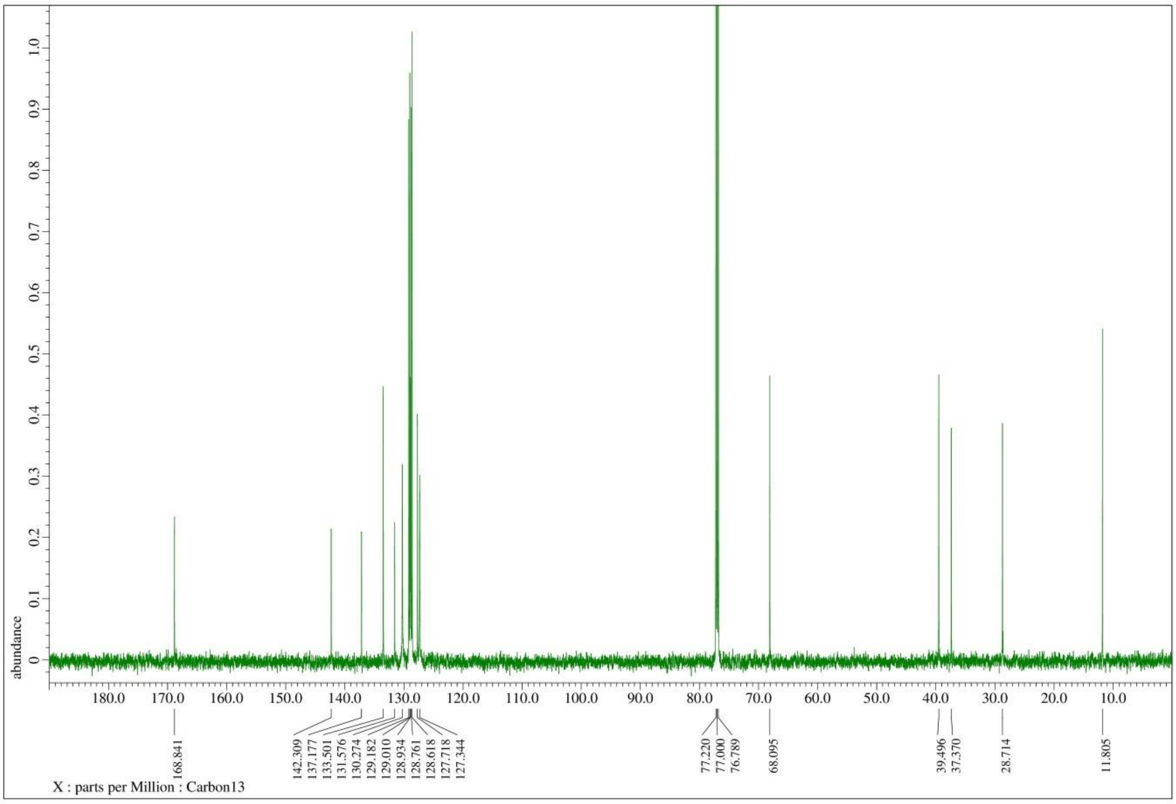

150 MHz, $CDCl_3$

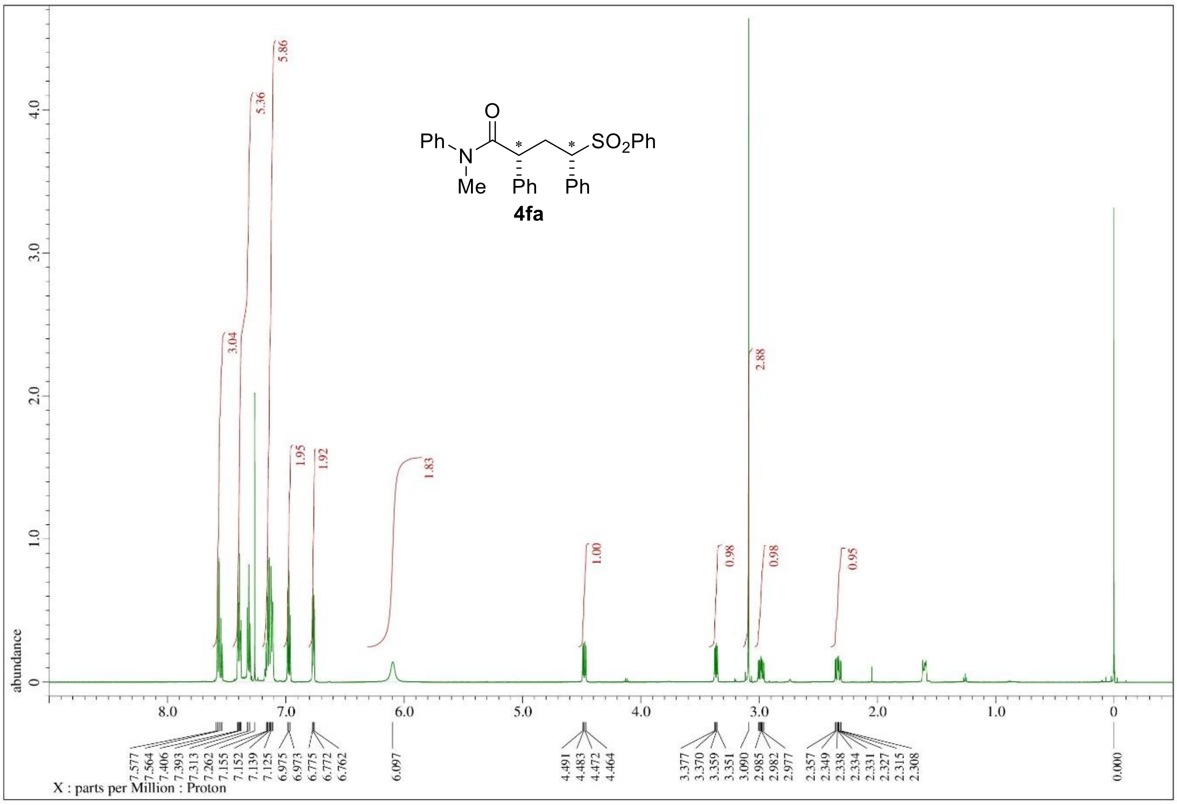

600 MHz, $CDCl_3$

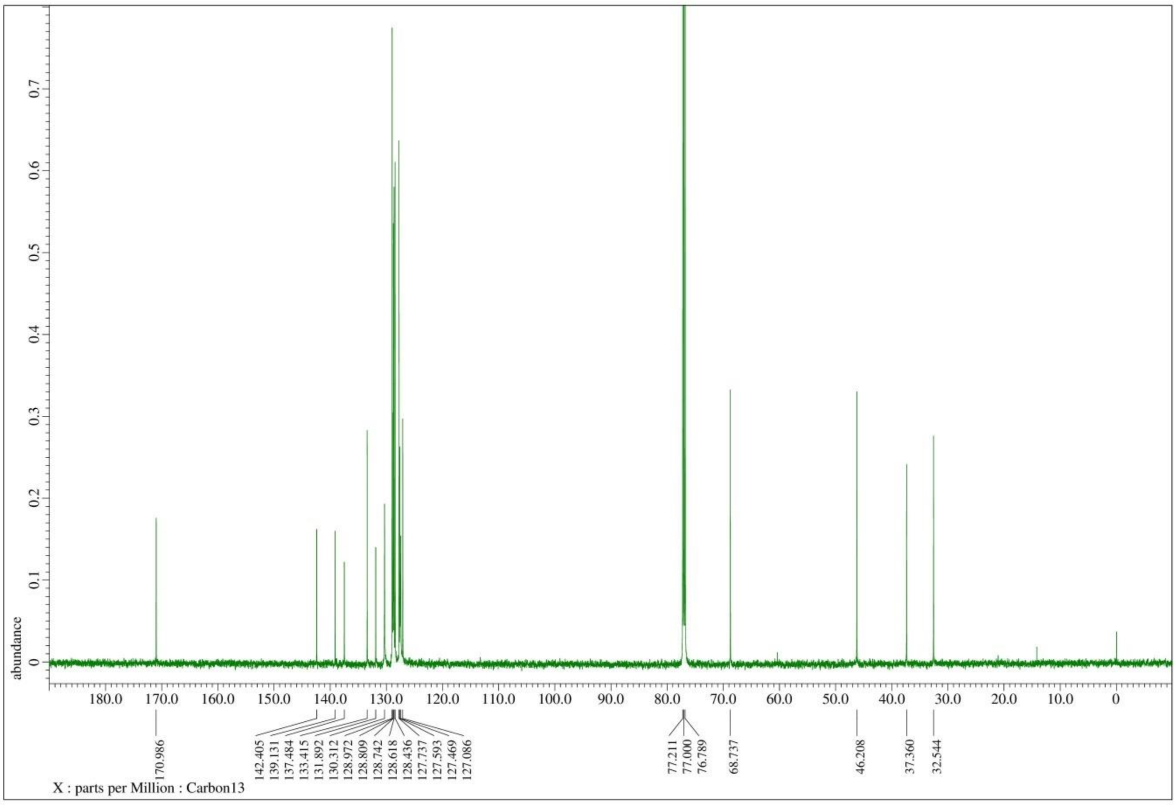

150 MHz, $CDCl_3$

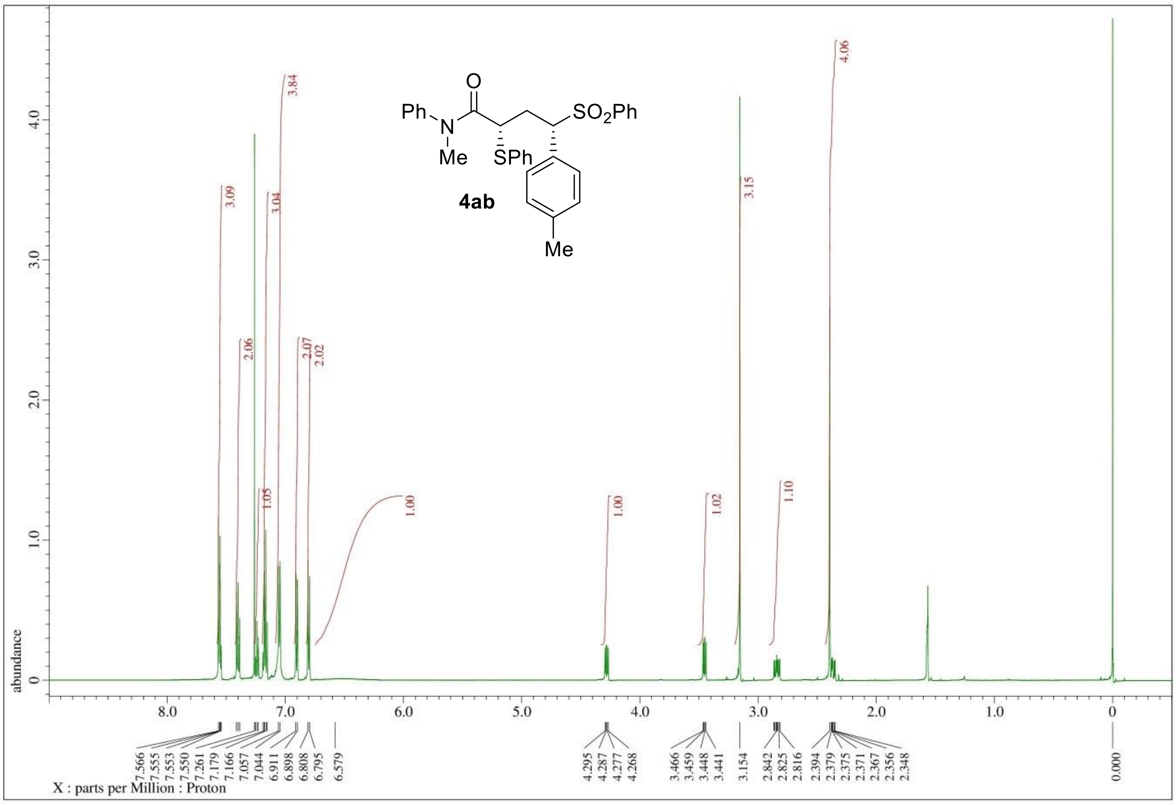

600 MHz, $CDCl_3$

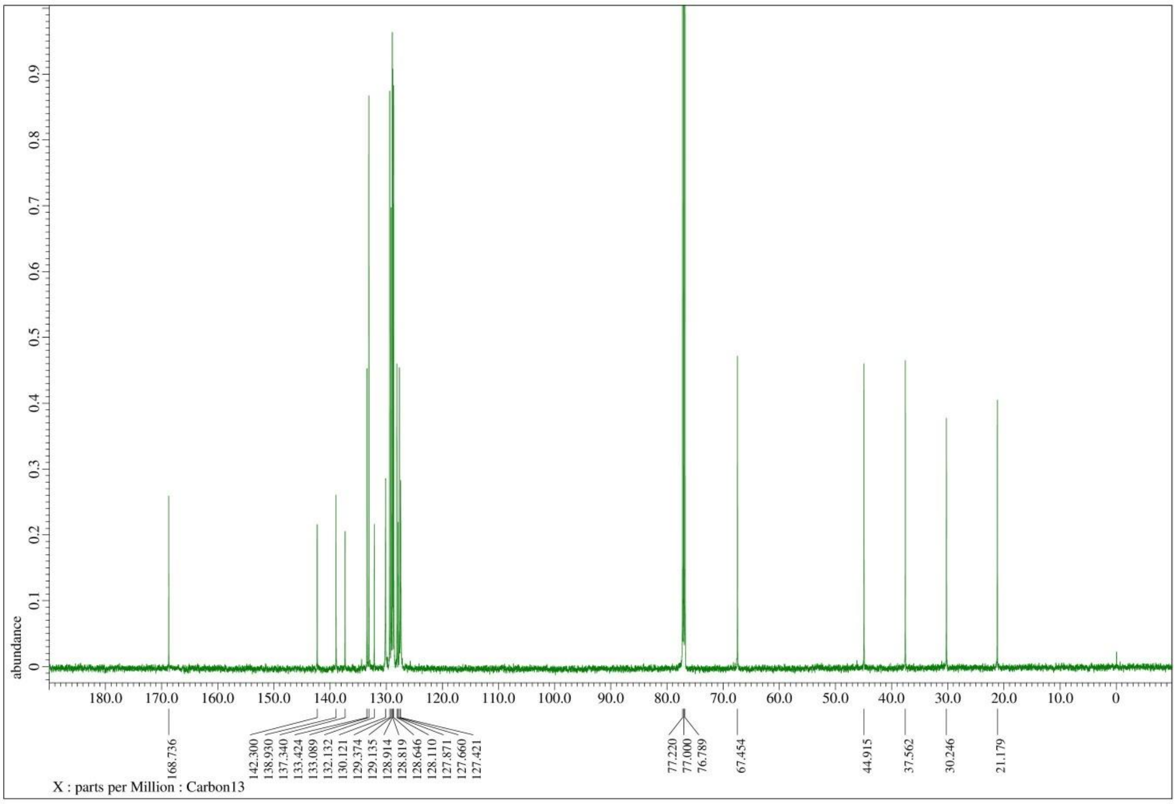

150 MHz, $CDCl_3$

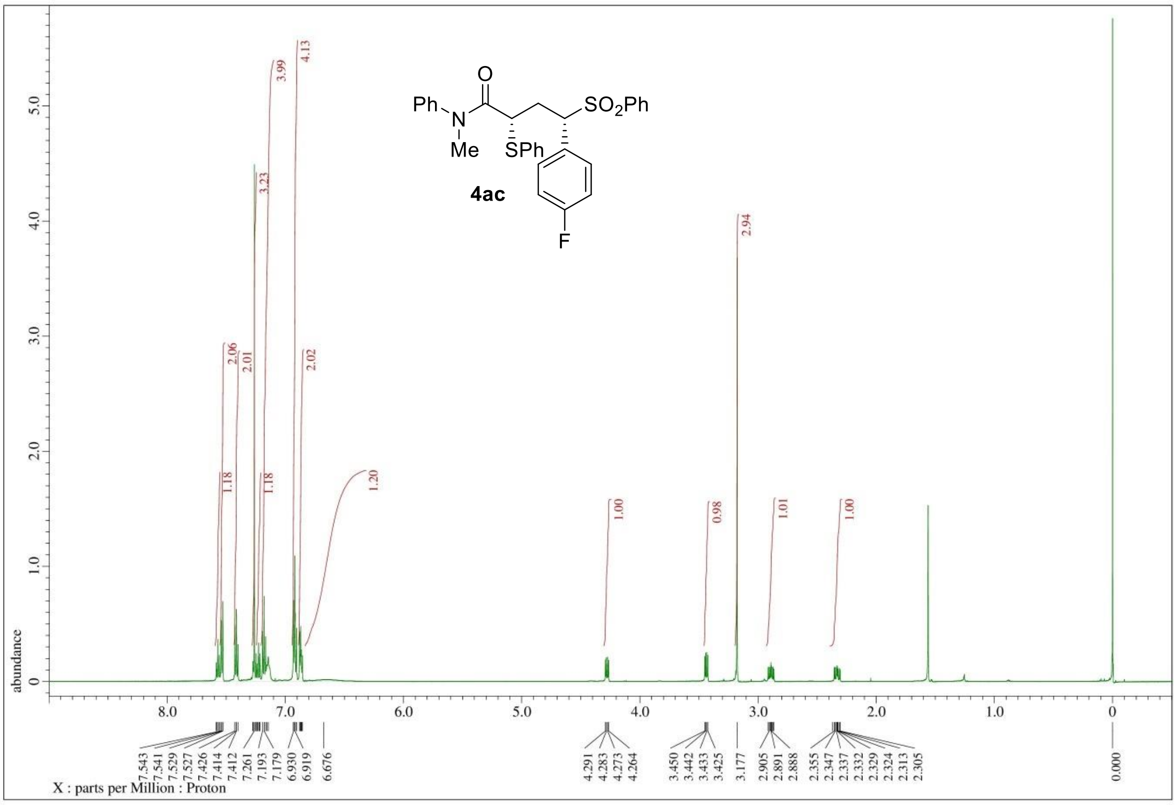

600 MHz, $CDCl_3$

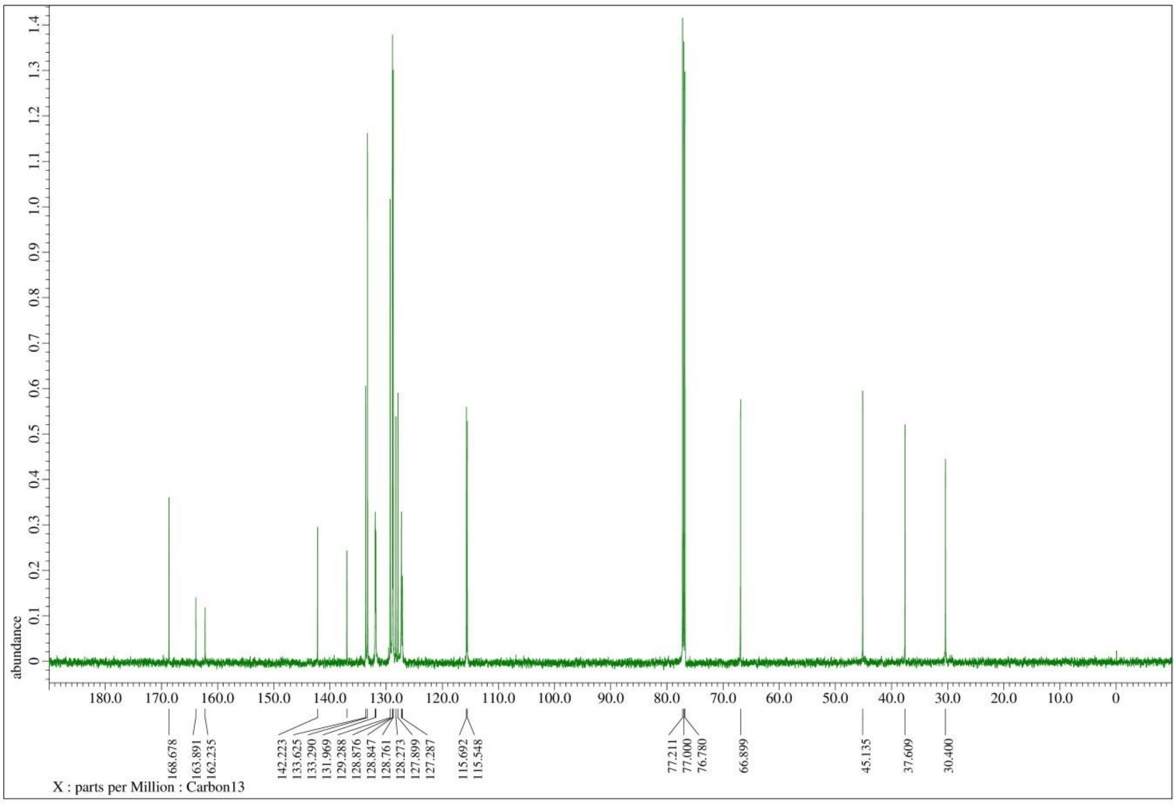

150 MHz, $CDCl_3$

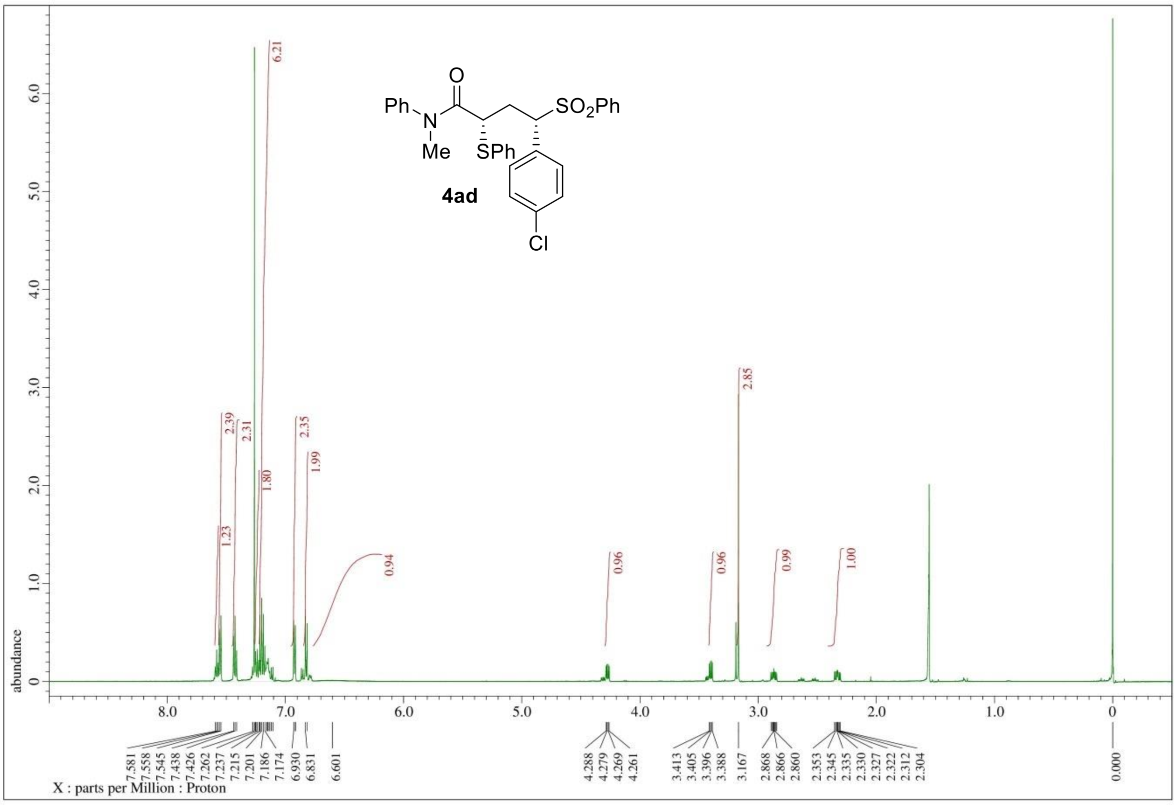

600 MHz, $CDCl_3$

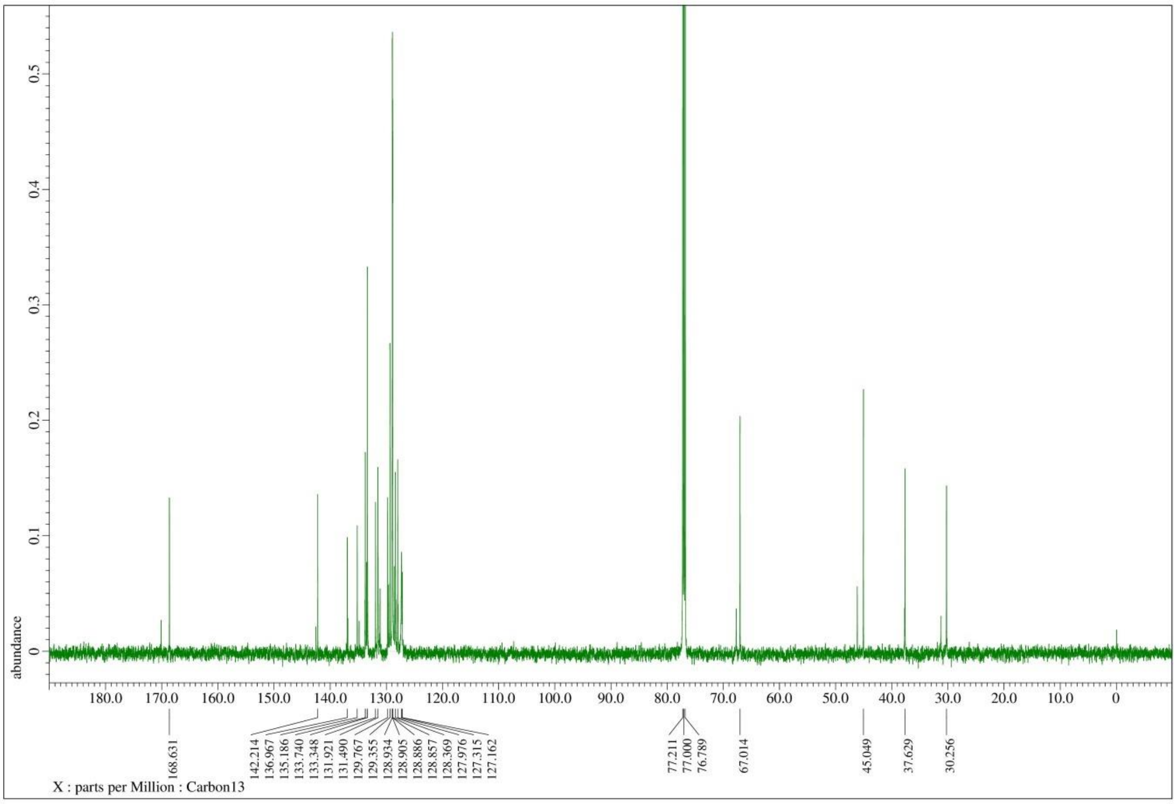

150 MHz, $CDCl_3$

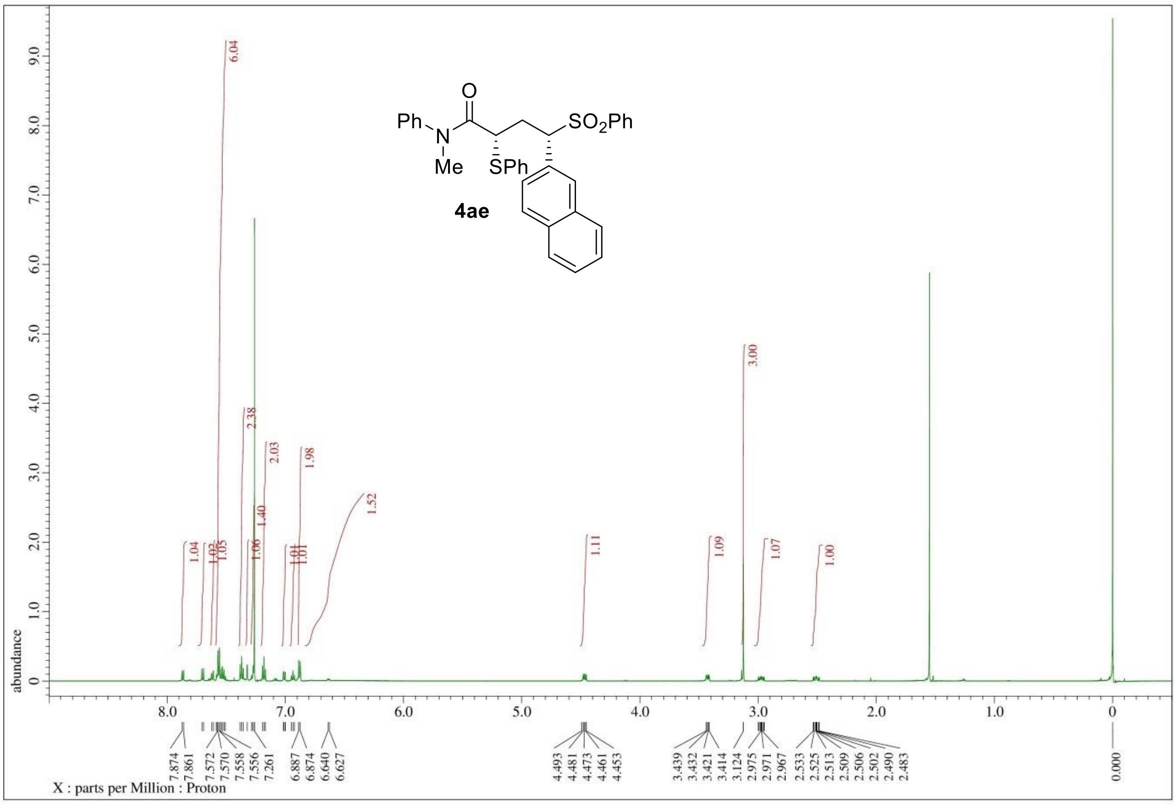

600 MHz, $CDCl_3$

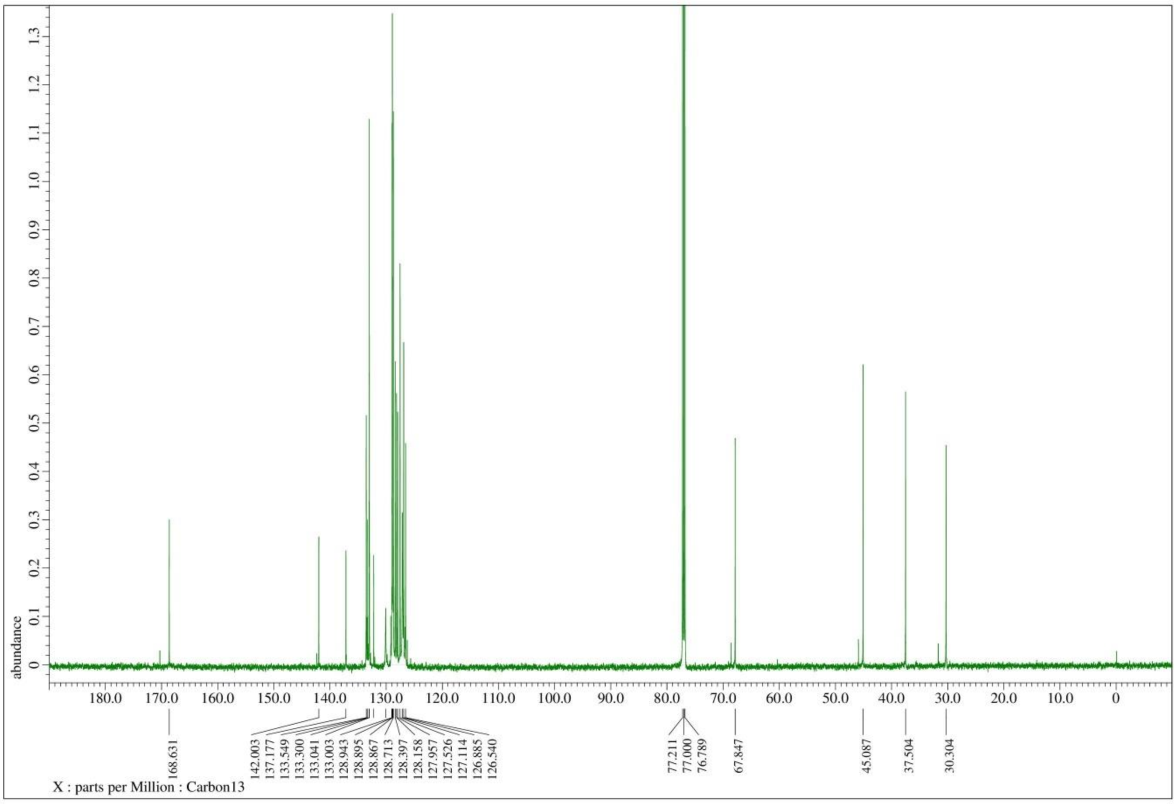

150 MHz, $CDCl_3$

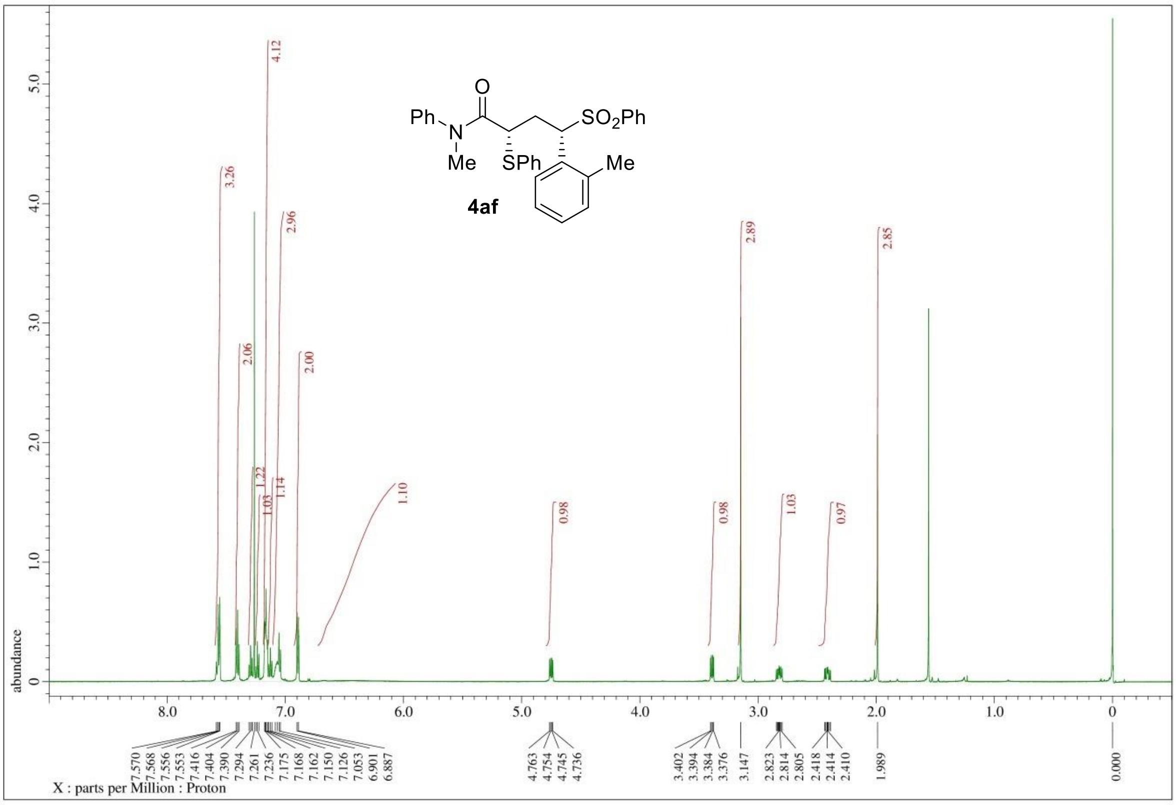

600 MHz, $CDCl_3$

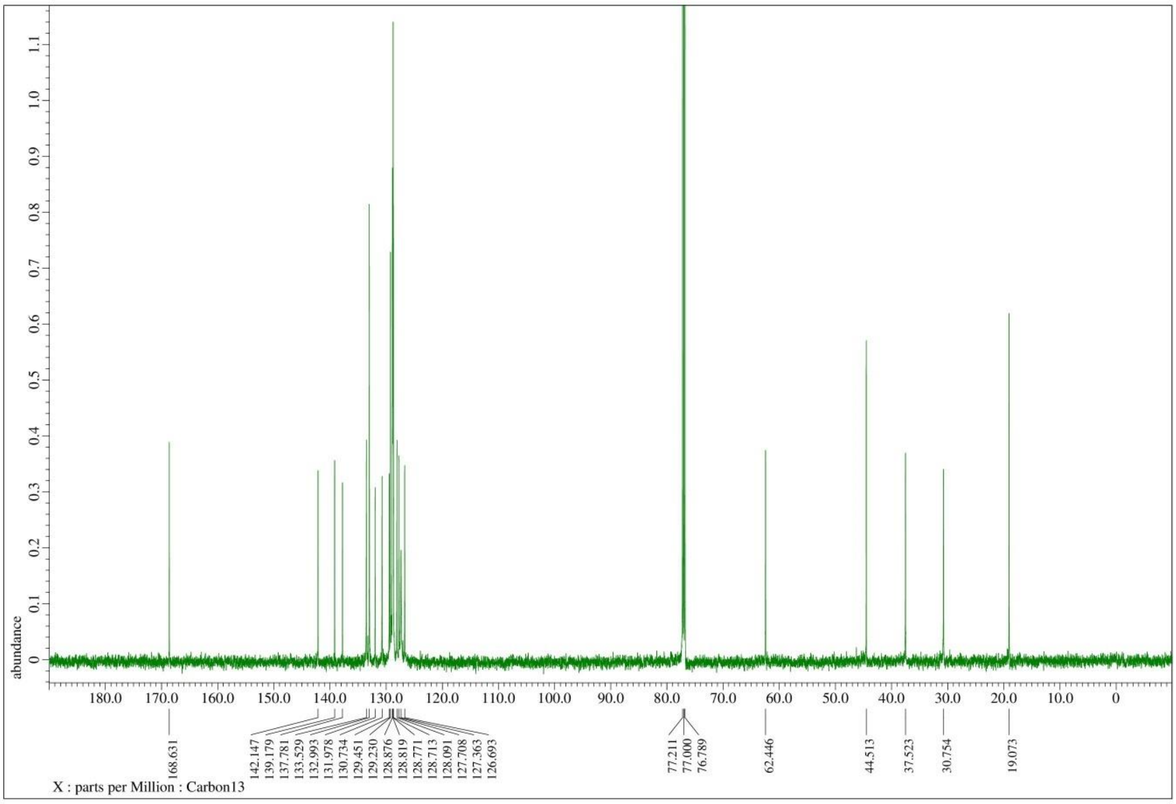

150 MHz, $CDCl_3$

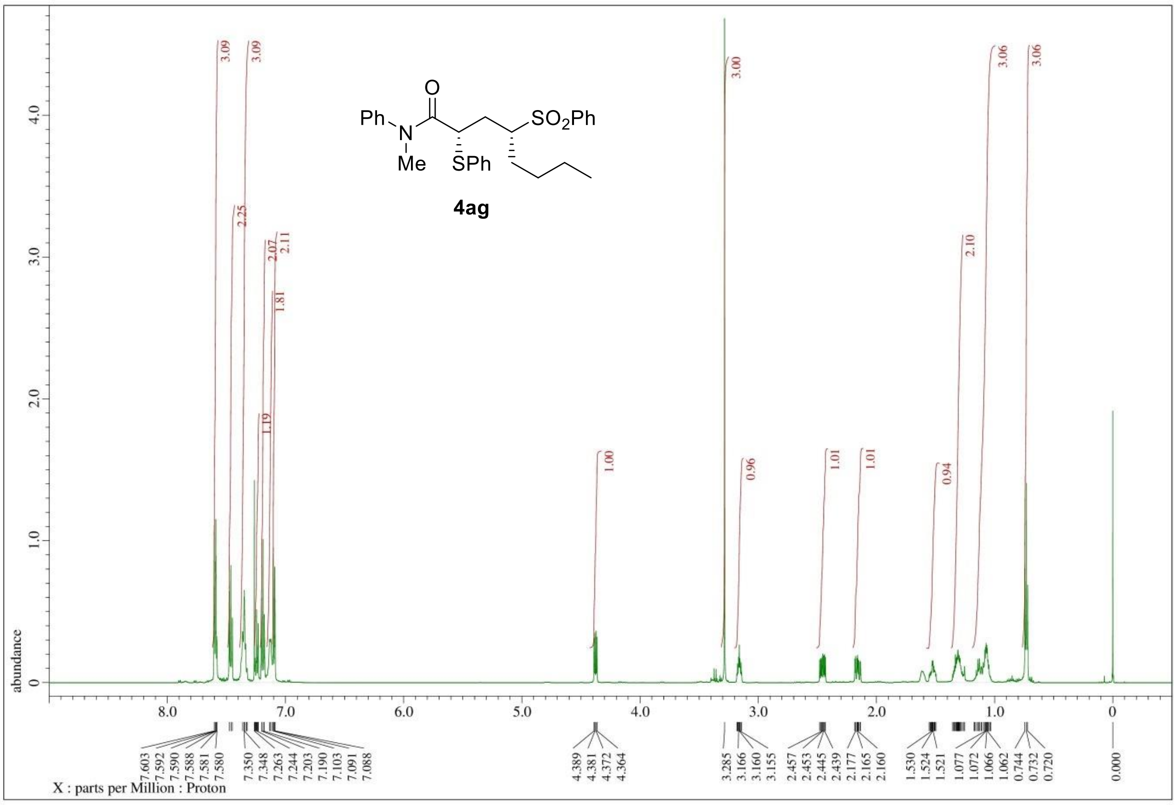

600 MHz, $CDCl_3$

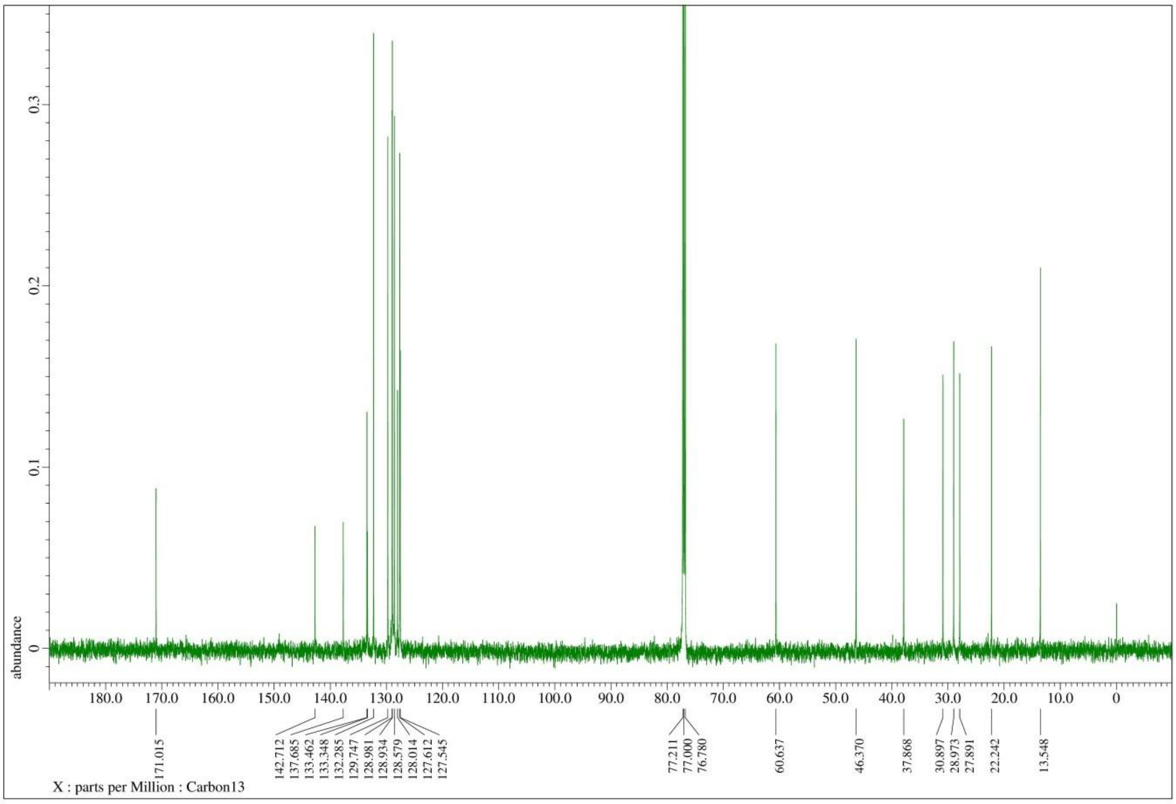

150 MHz, $CDCl_3$

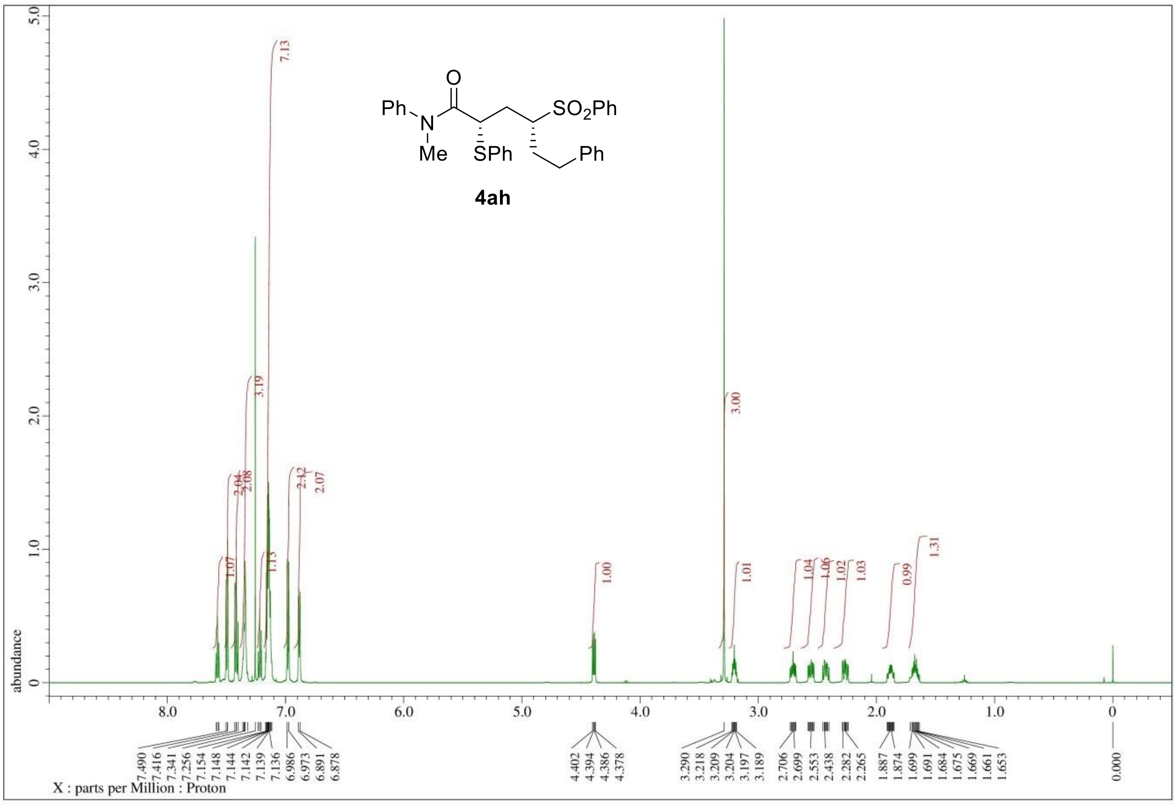

600 MHz, $CDCl_3$

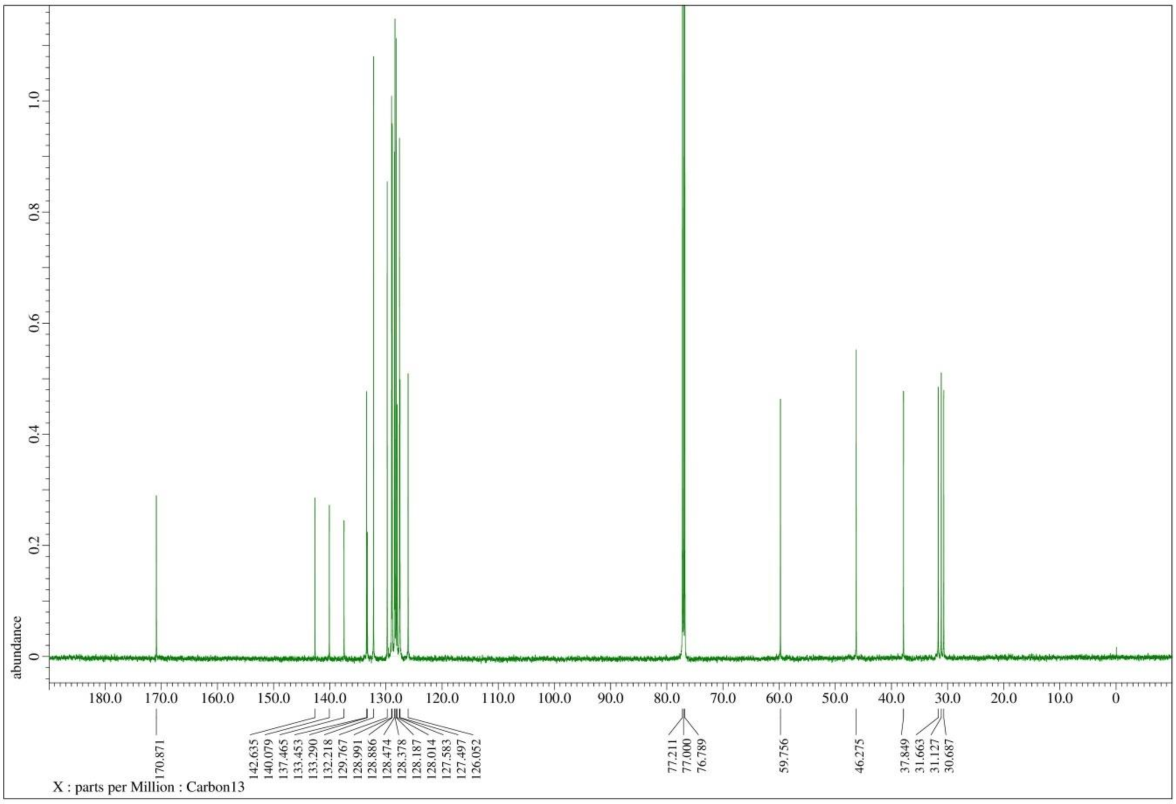

150 MHz, $CDCl_3$

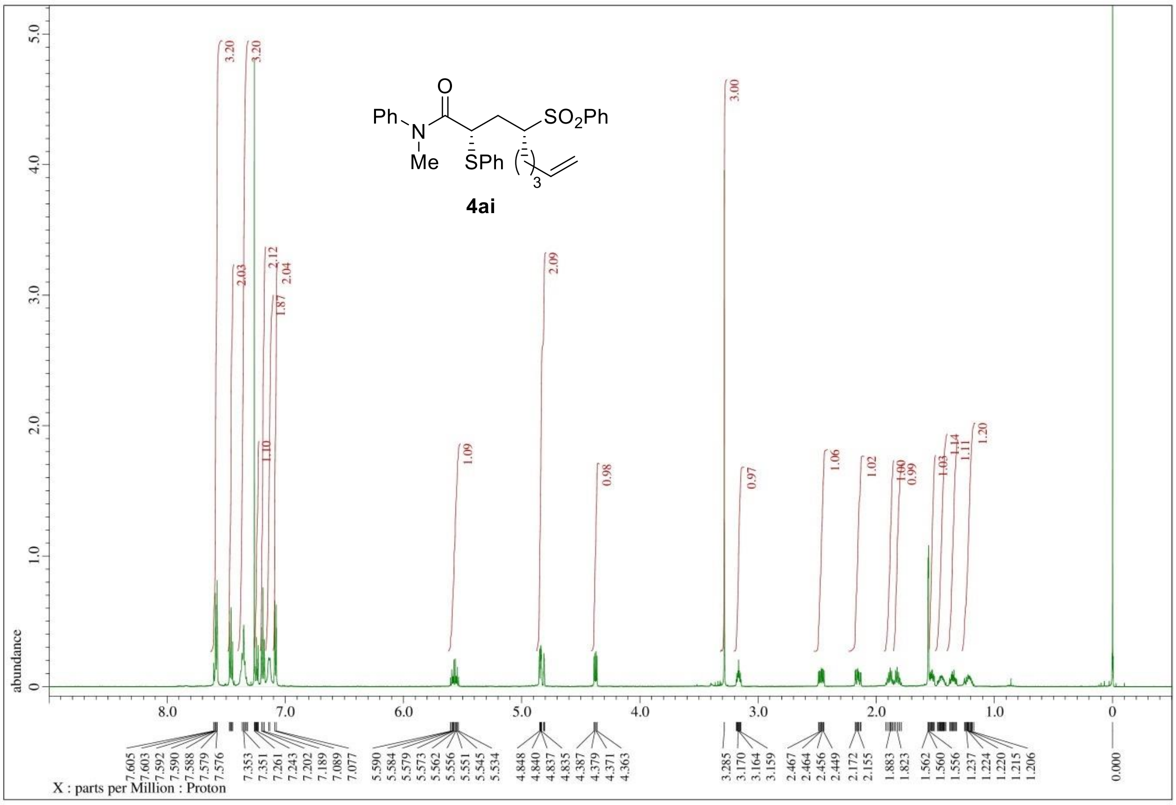

600 MHz, $CDCl_3$

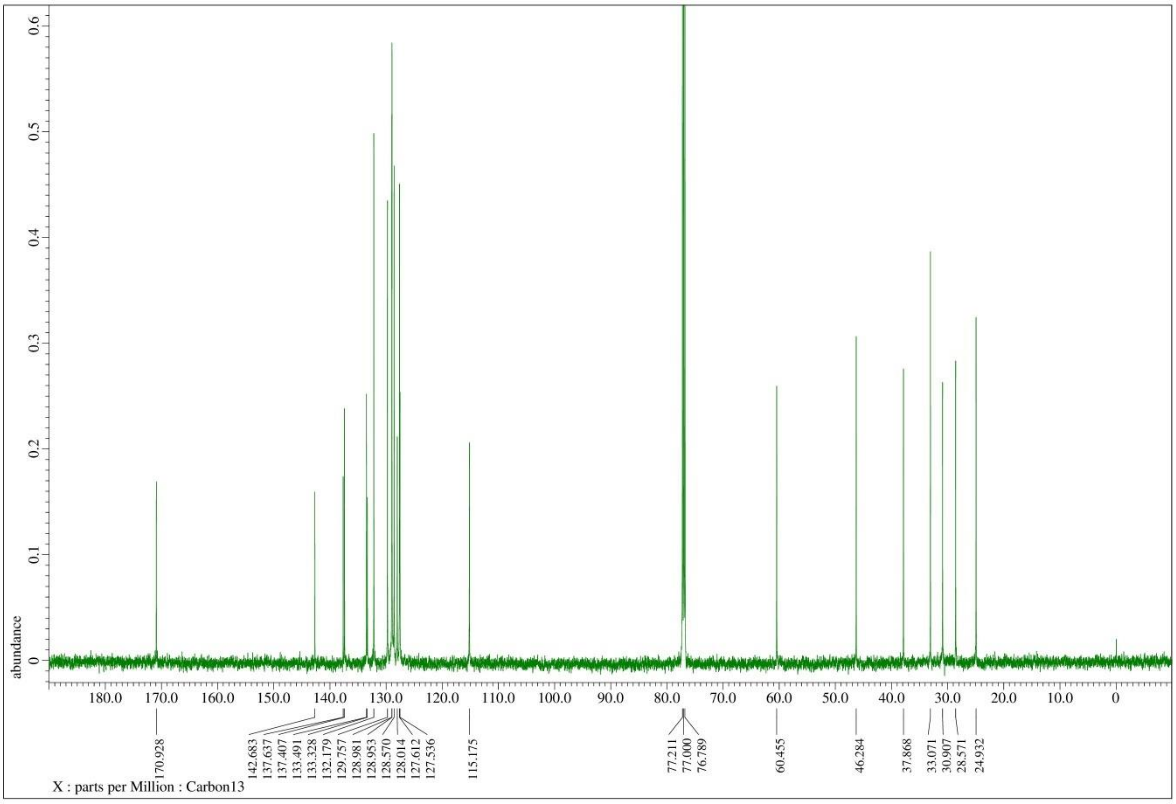

150 MHz, $CDCl_3$

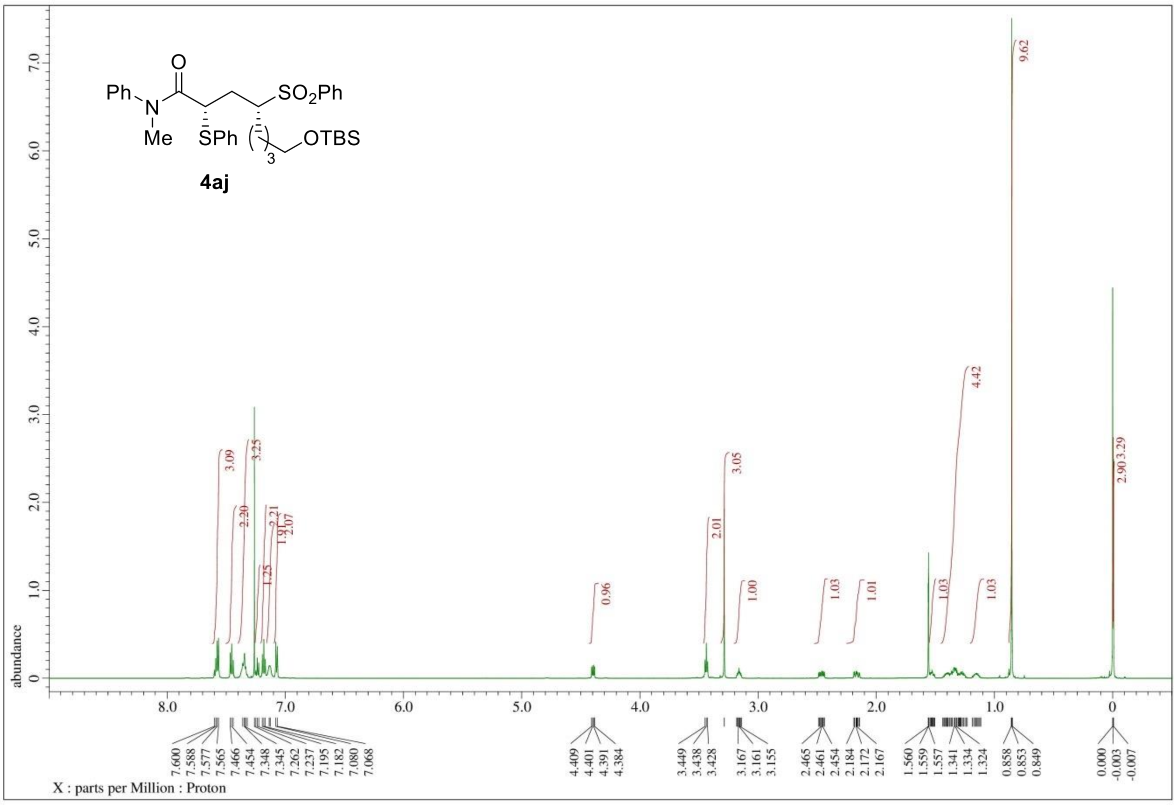

600 MHz, $CDCl_3$

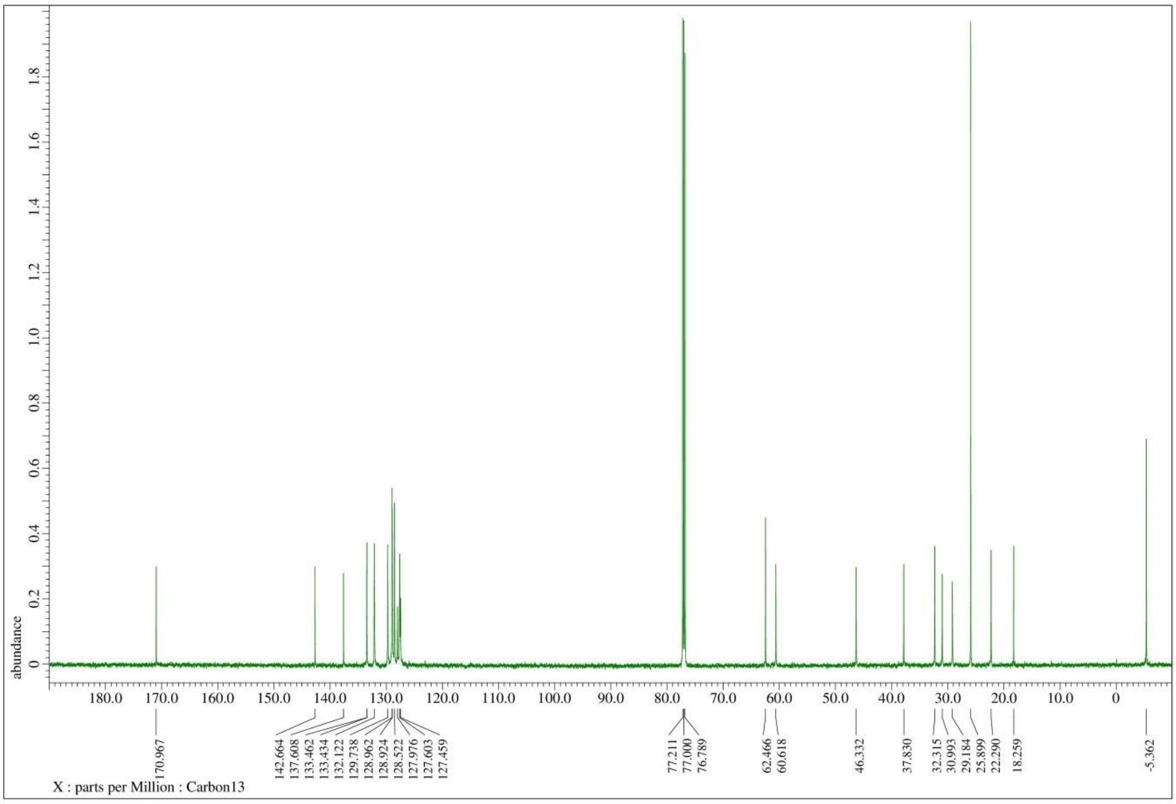

150 MHz, $CDCl_3$

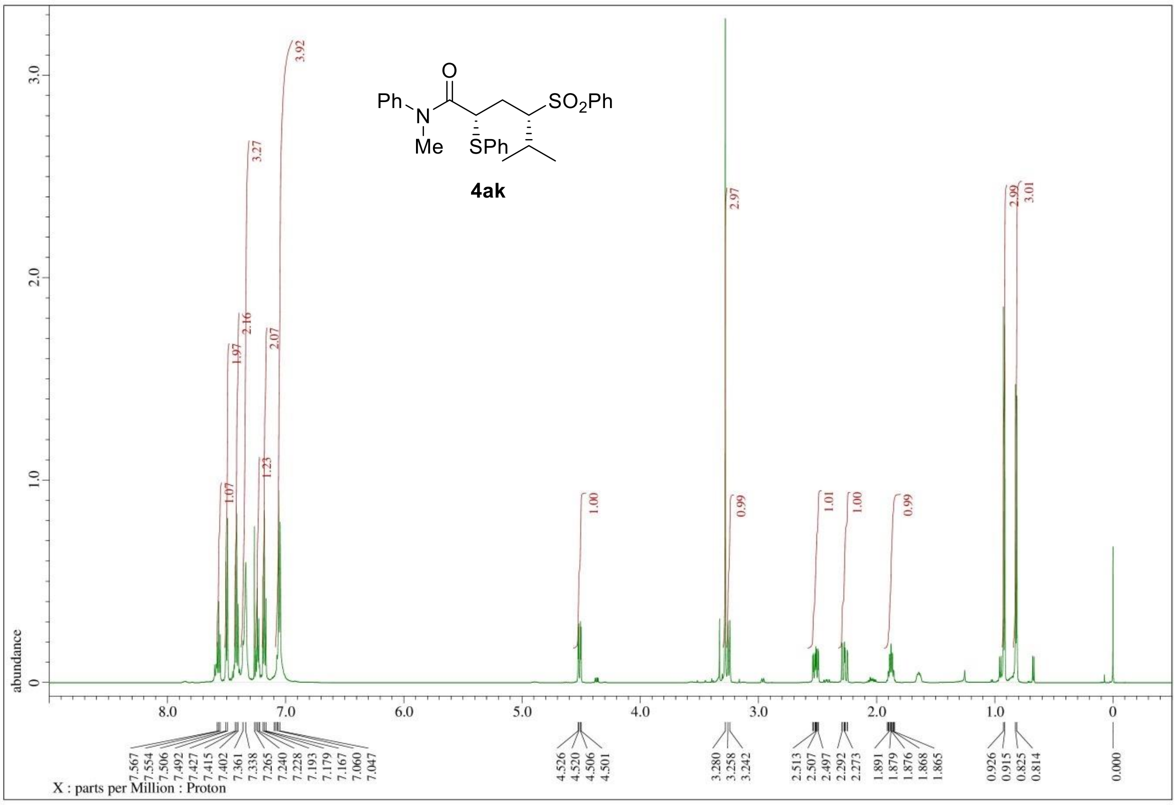

600 MHz, $CDCl_3$

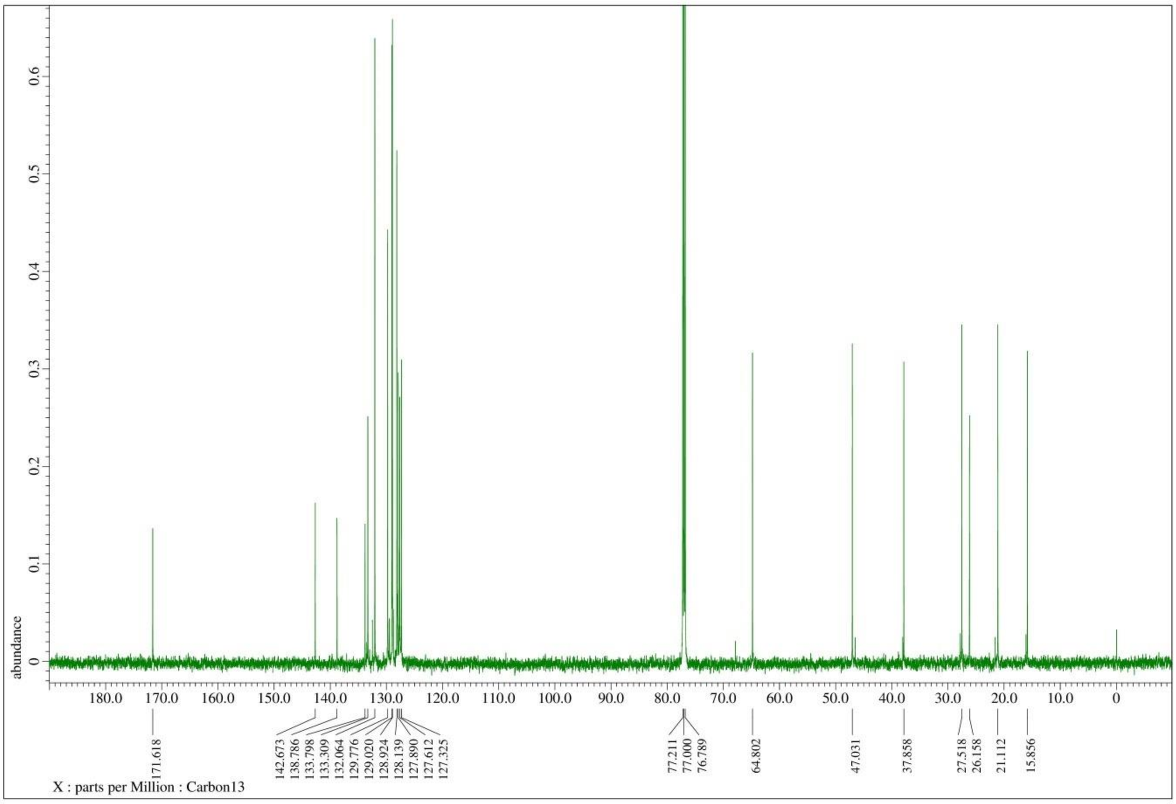

150 MHz, $CDCl_3$

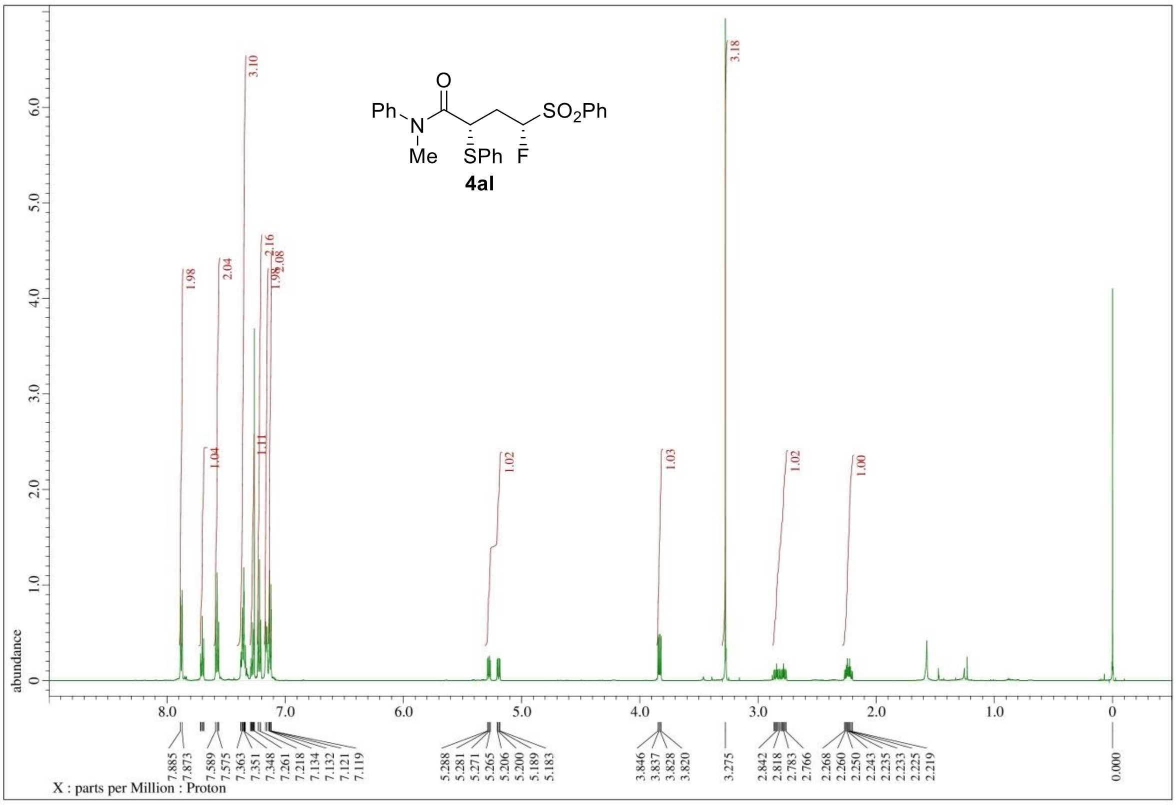

600 MHz, $CDCl_3$

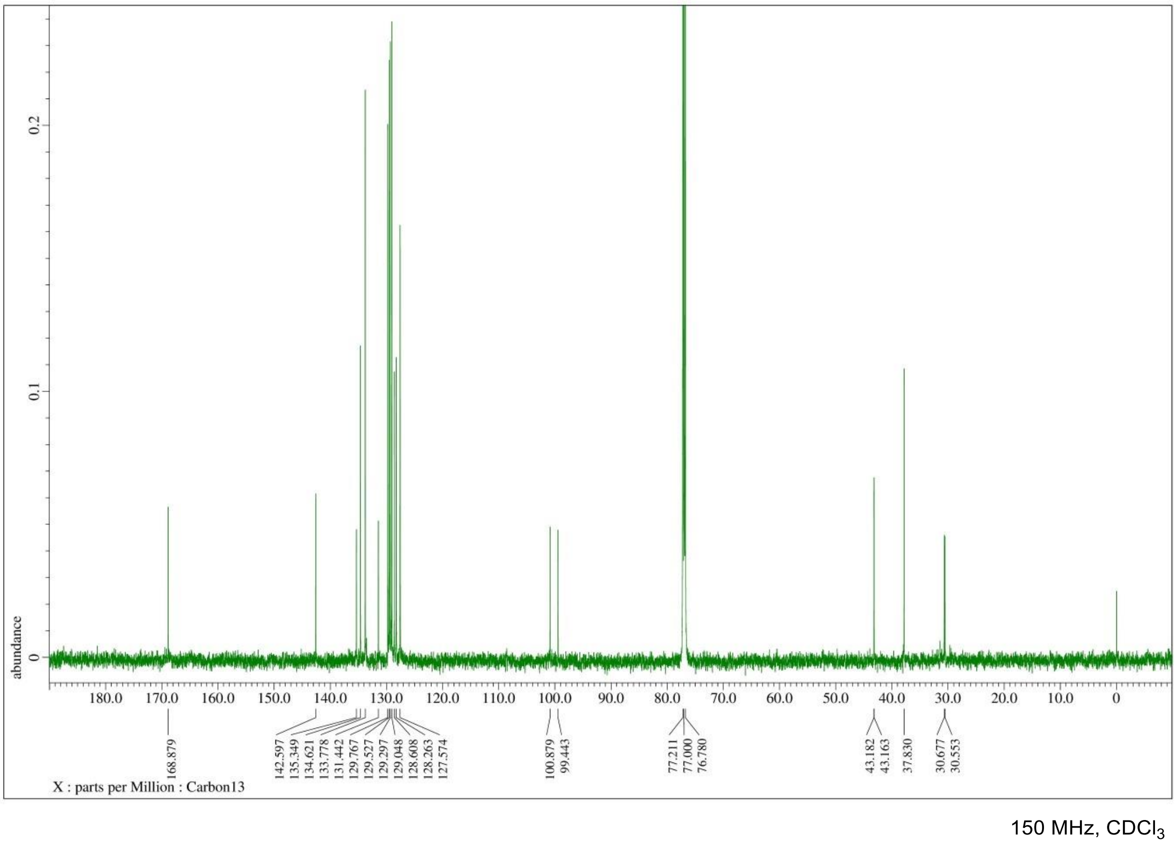

150 MHz, $CDCl_3$

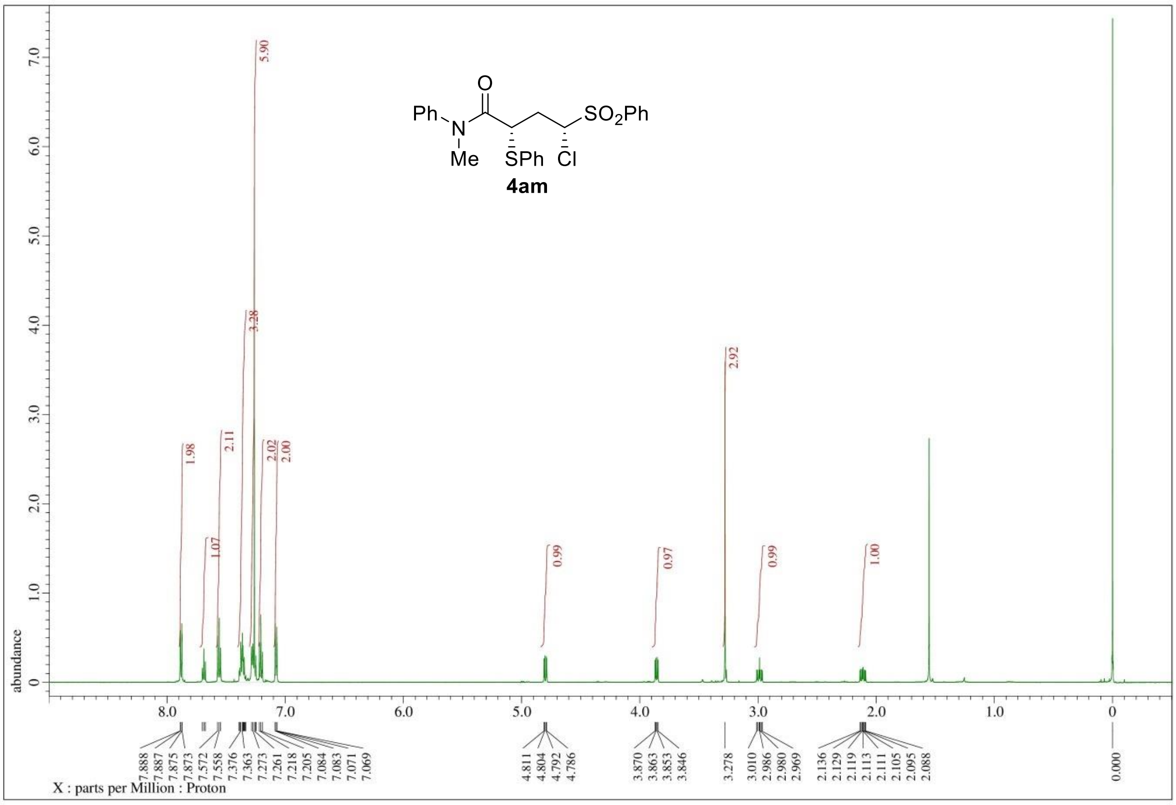

600 MHz, $CDCl_3$

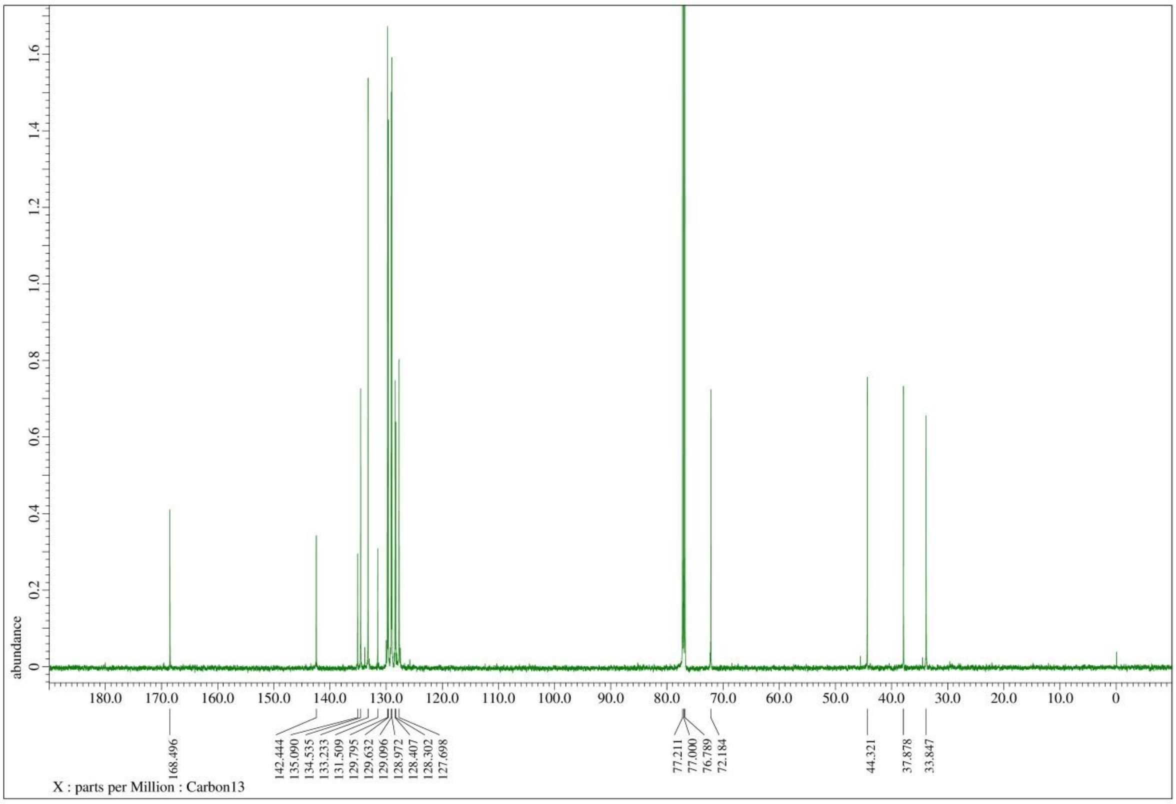

150 MHz, $CDCl_3$

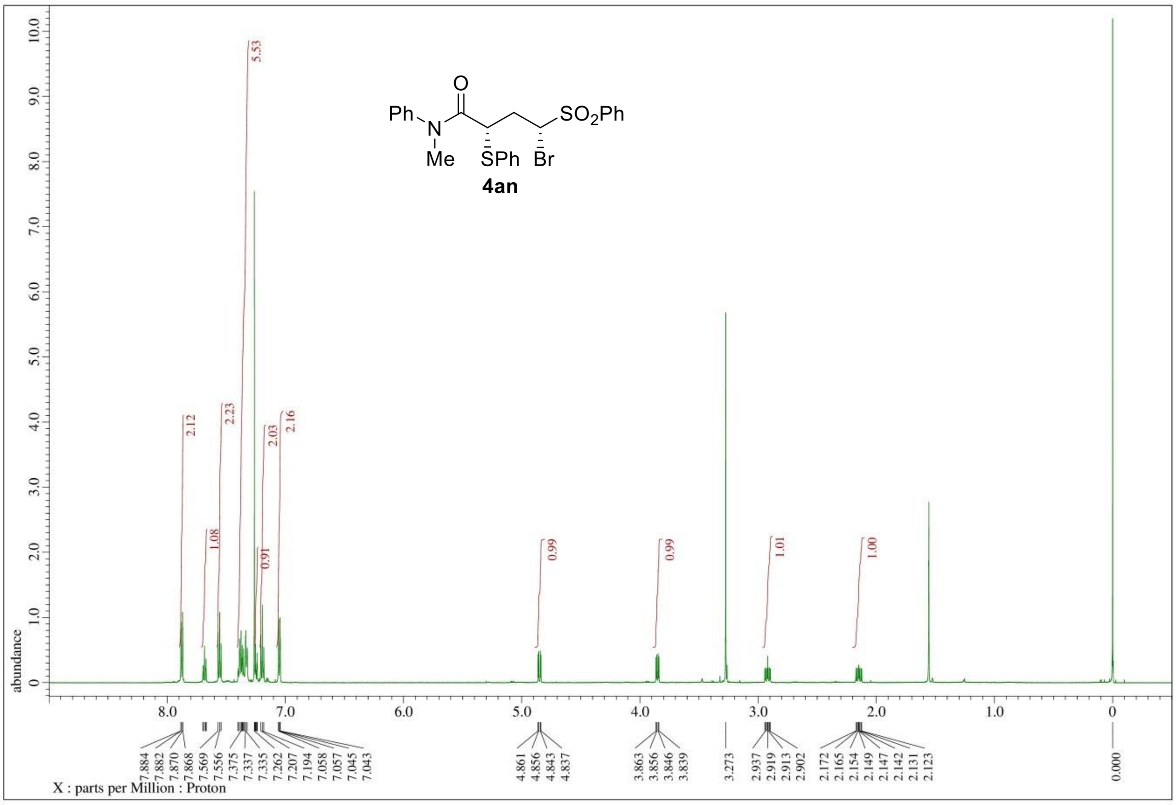

600 MHz, $CDCl_3$

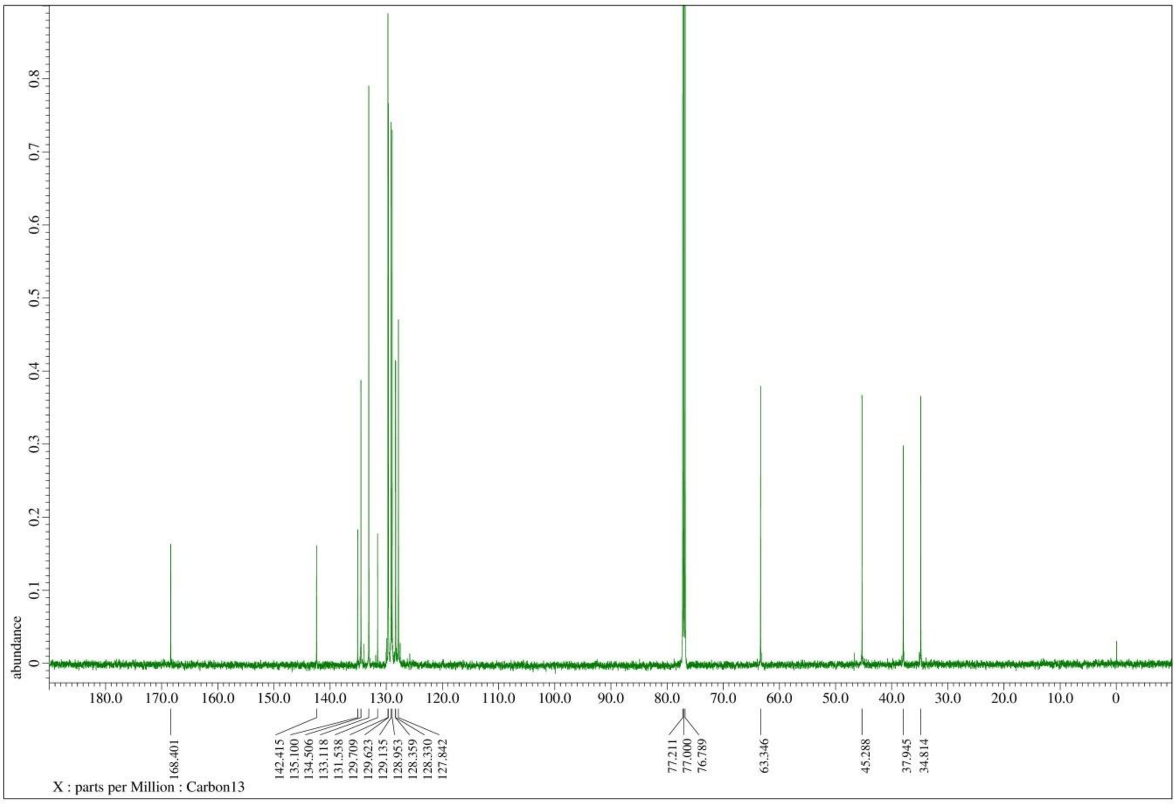

150 MHz, $CDCl_3$

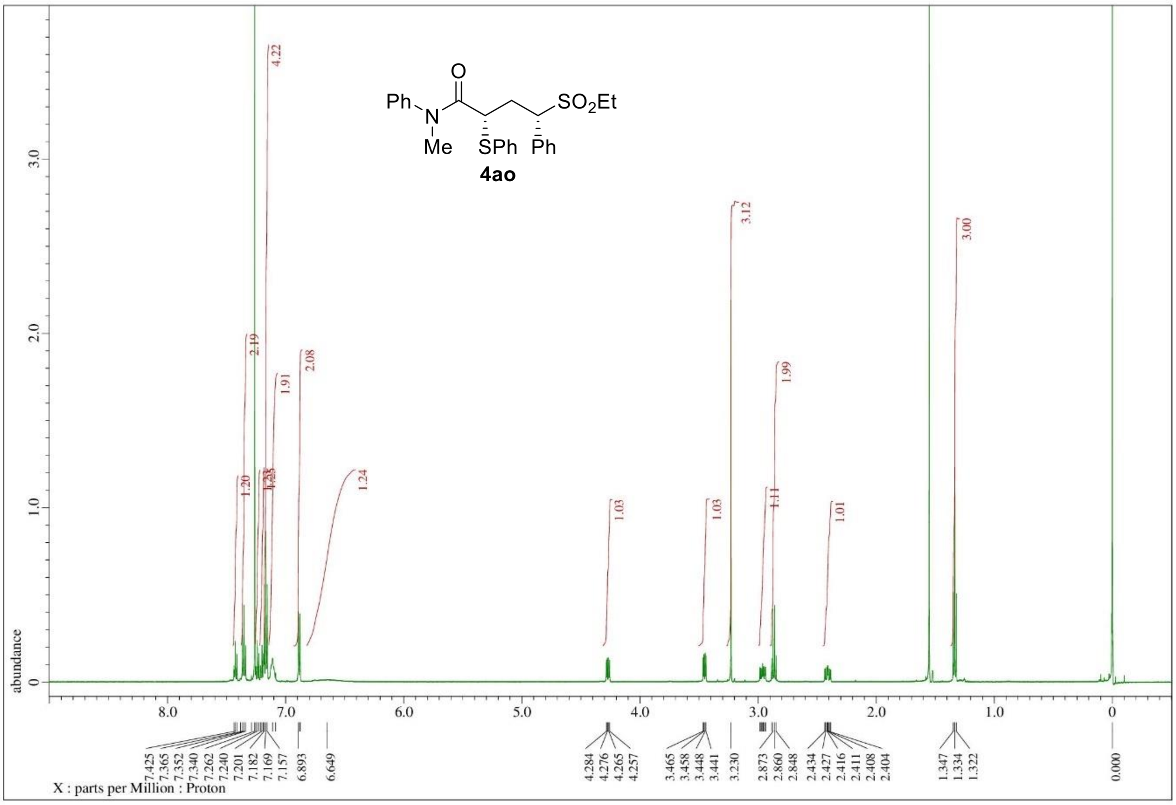

600 MHz, $CDCl_3$

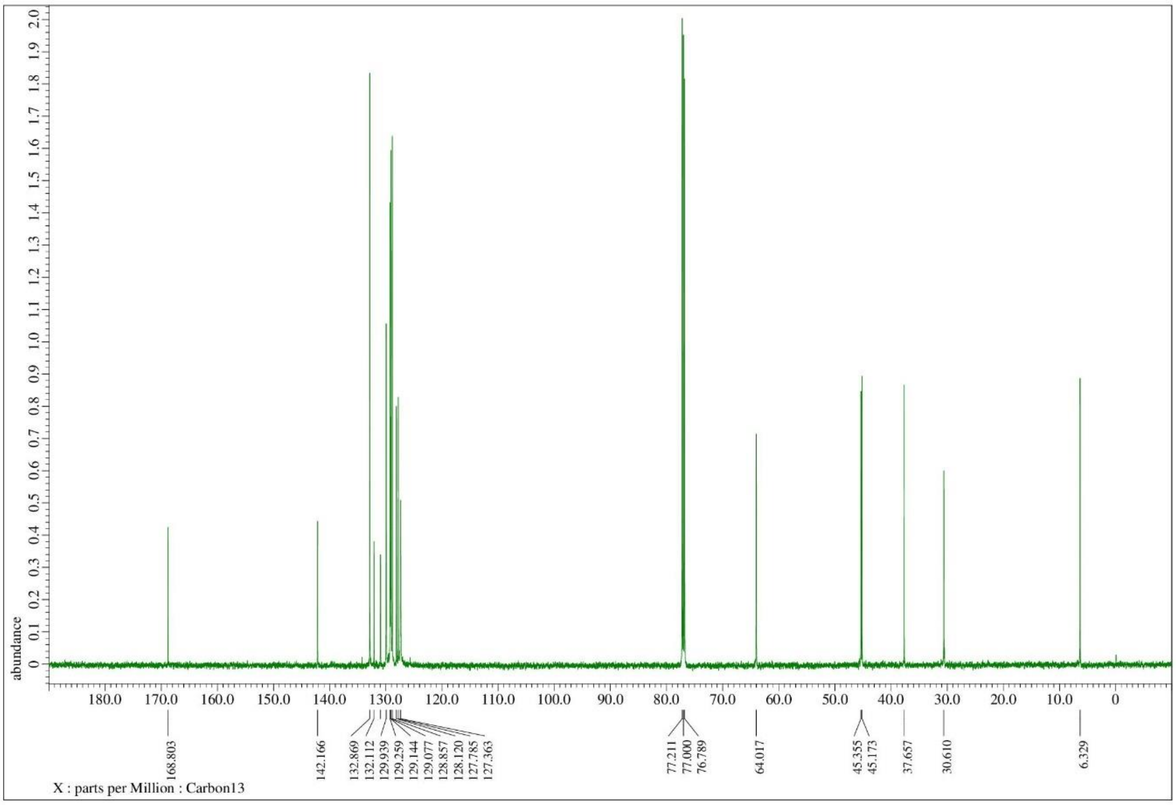

150 MHz, $CDCl_3$

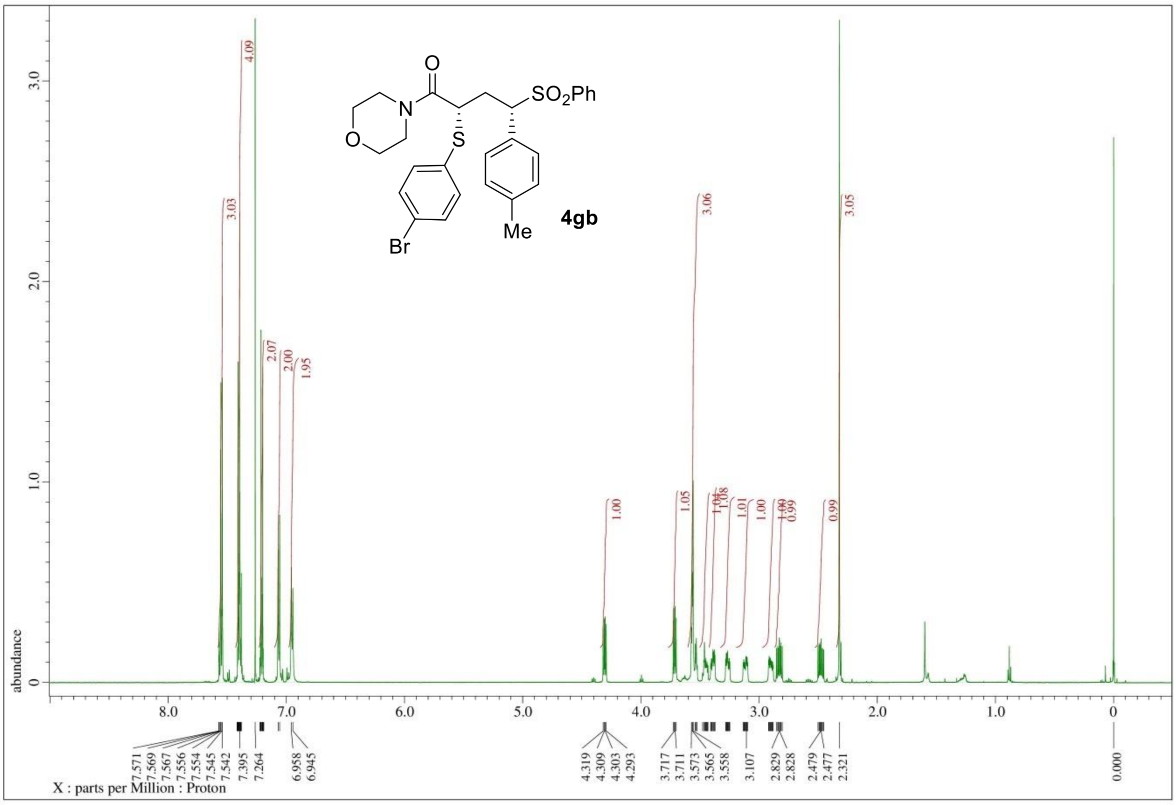

600 MHz, $CDCl_3$

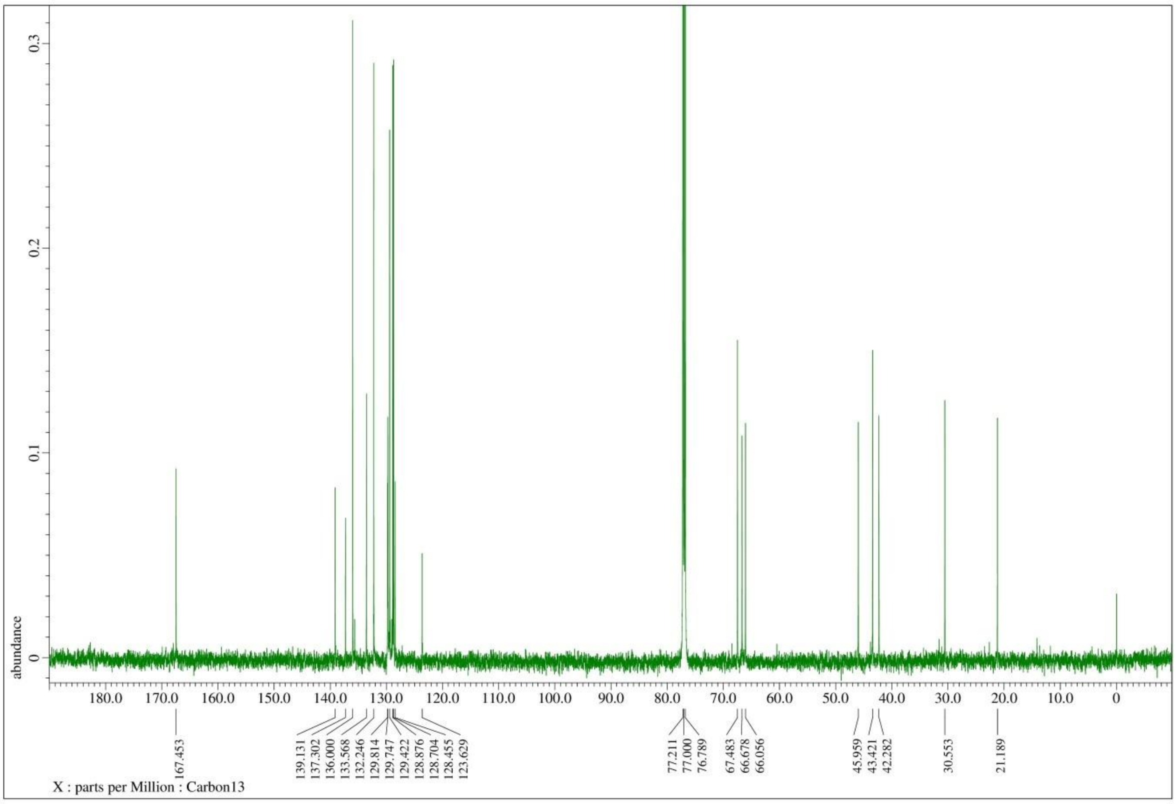

150 MHz, $CDCl_3$

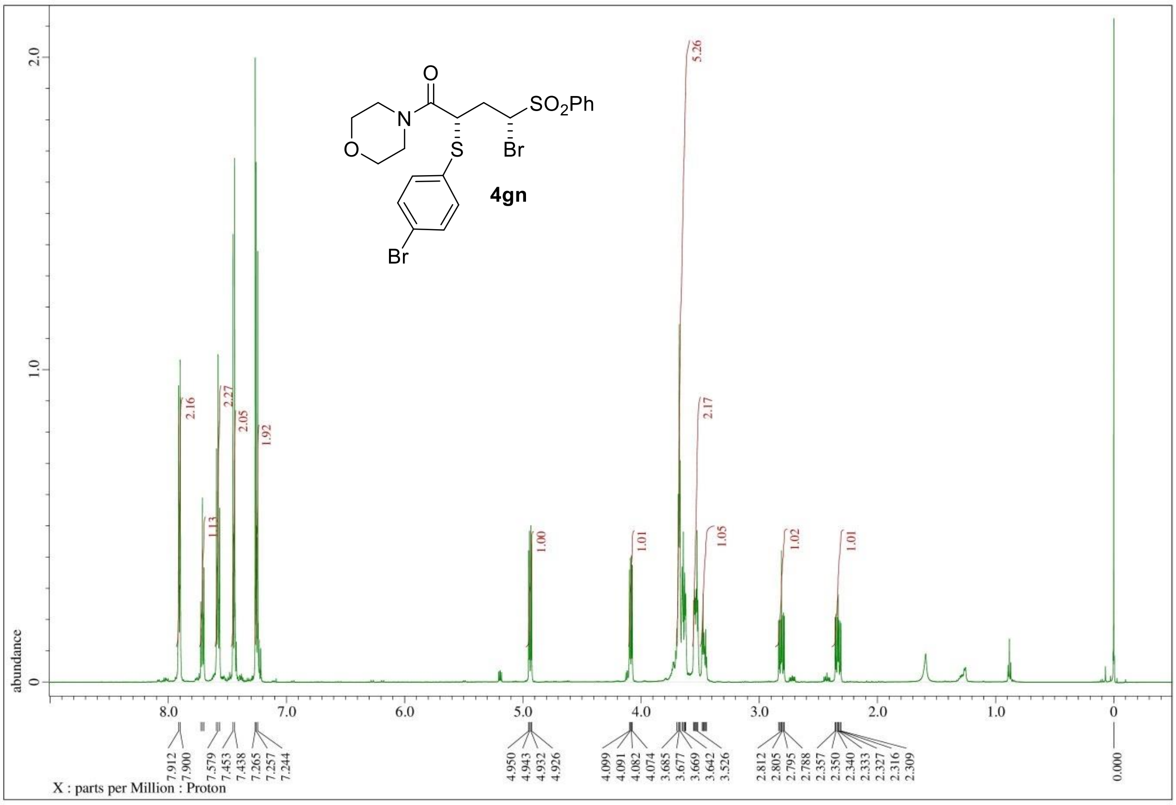

600 MHz, $CDCl_3$

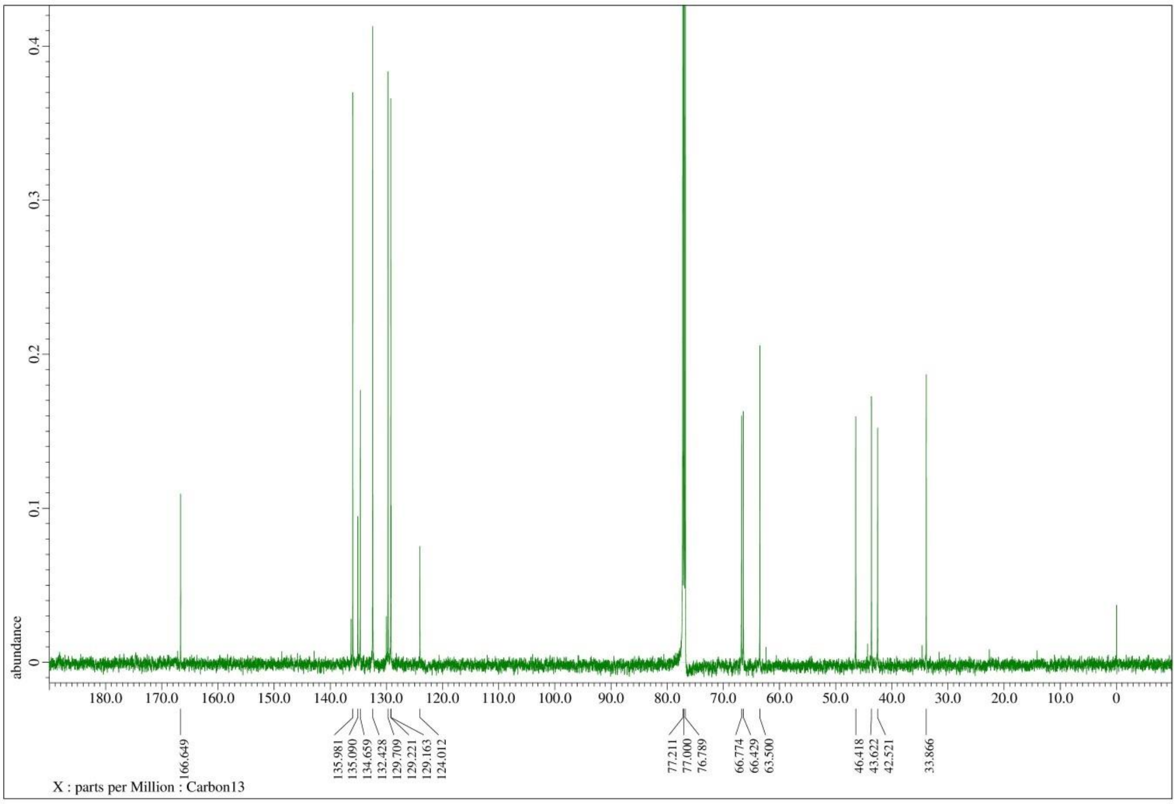

150 MHz, $CDCl_3$

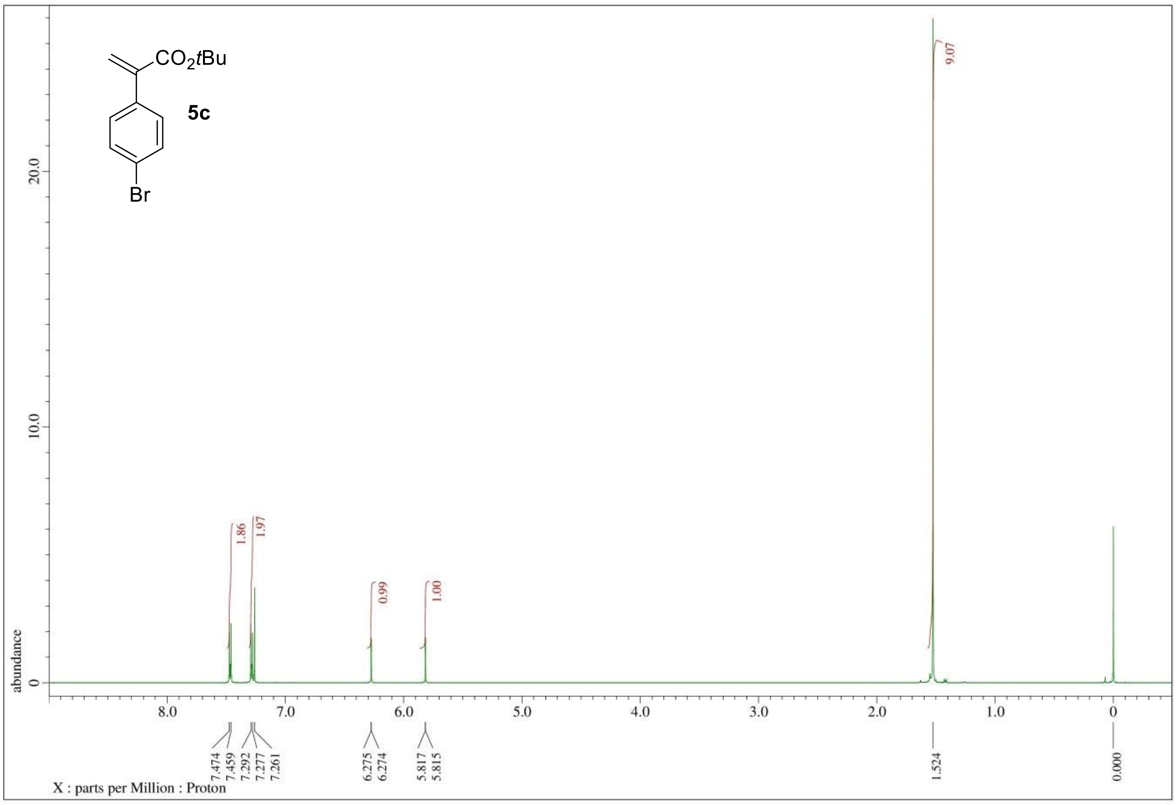

600 MHz, $CDCl_3$

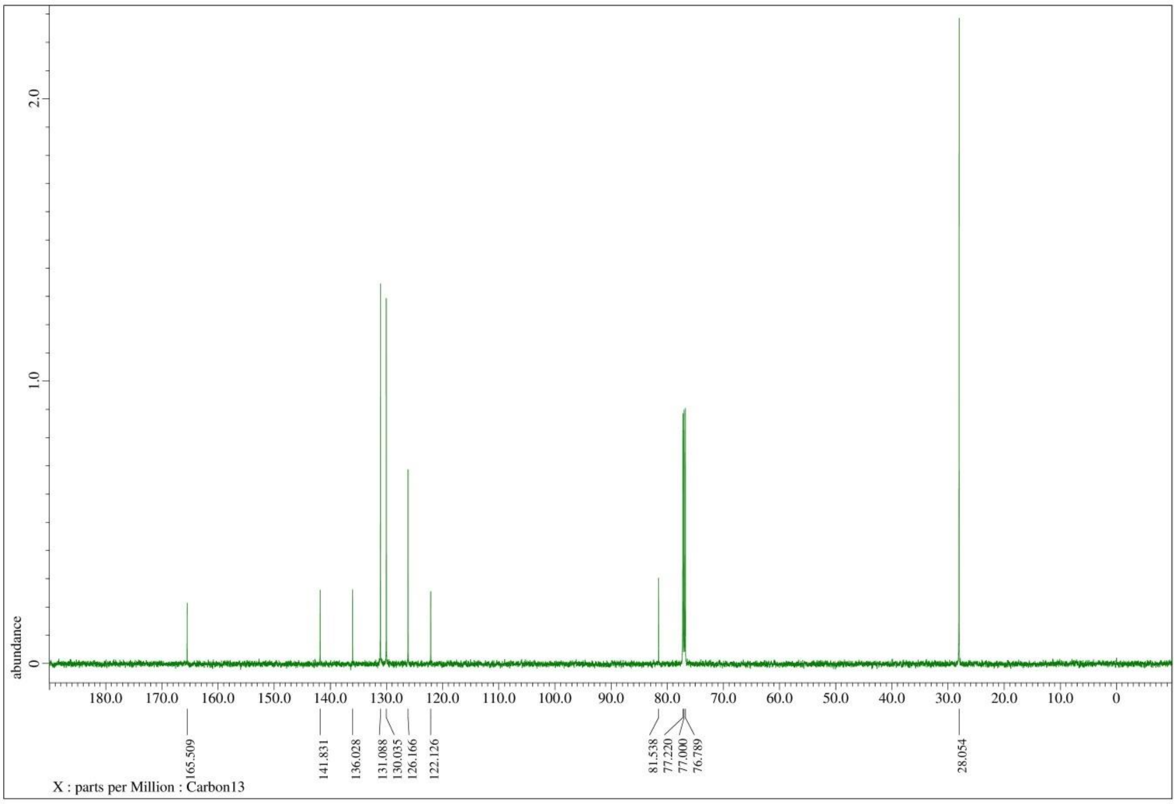

150 MHz, $CDCl_3$

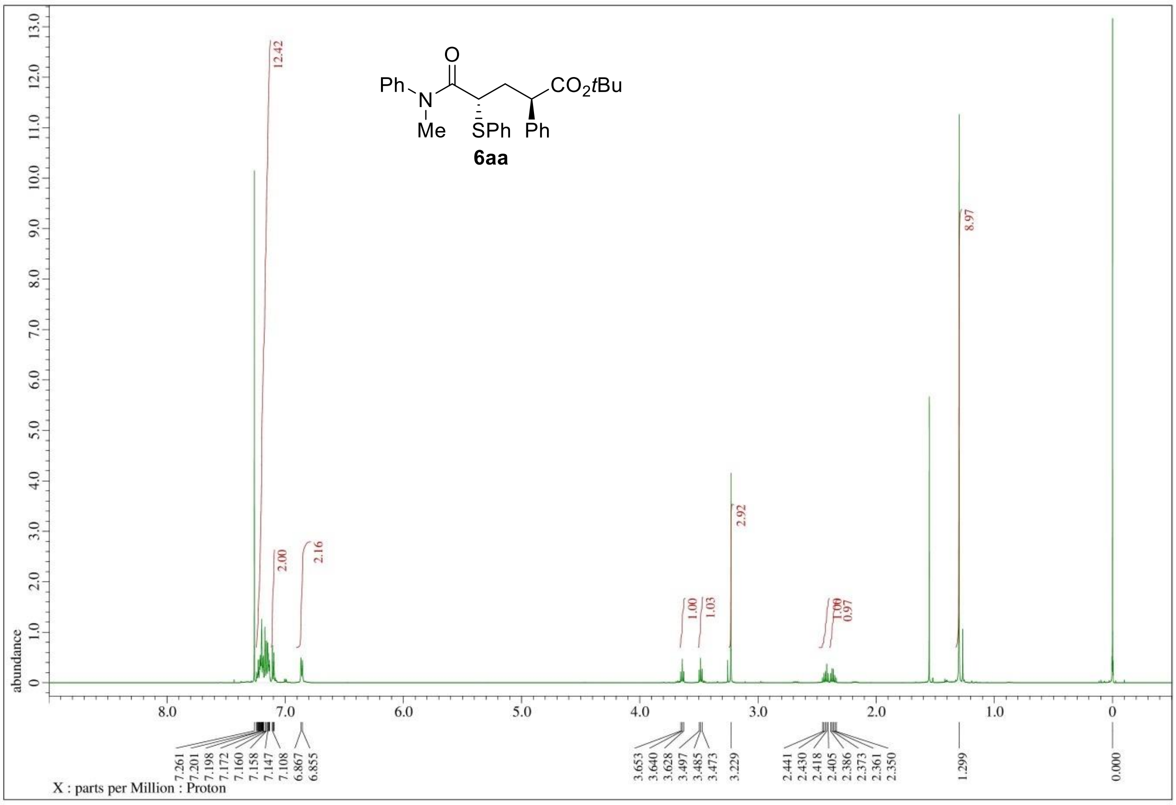

600 MHz, $CDCl_3$

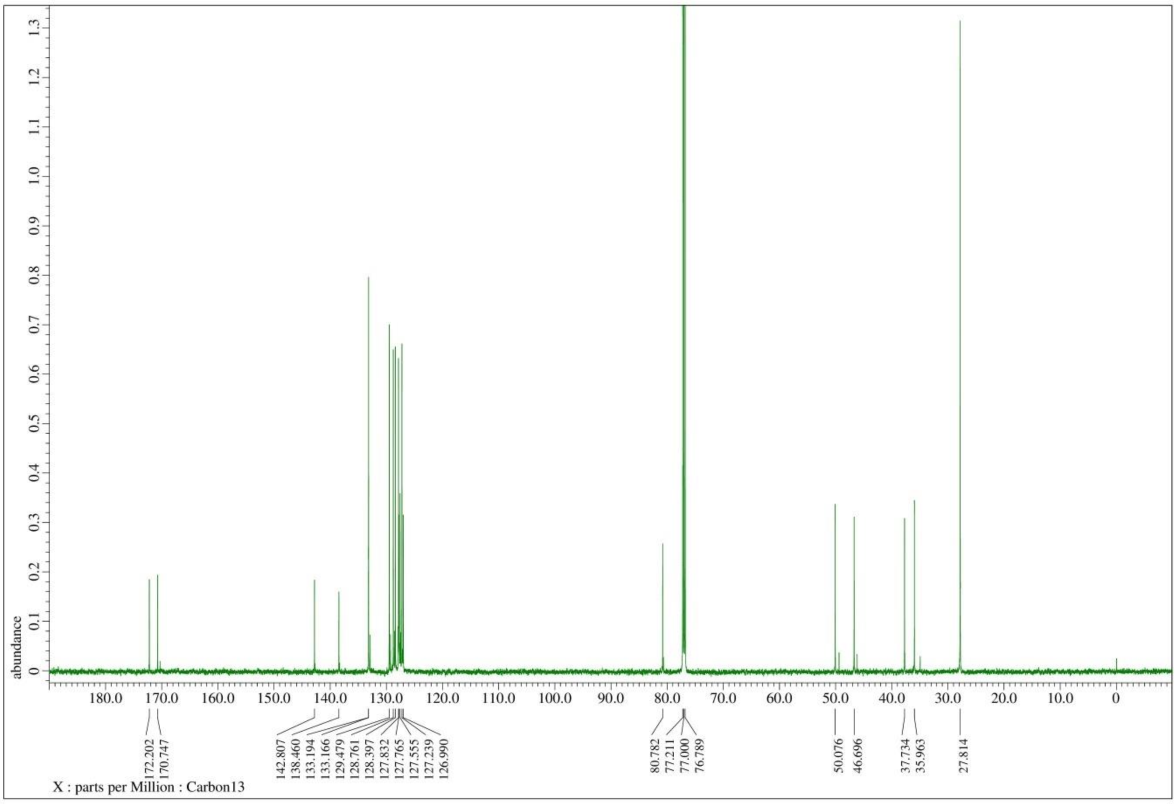

150 MHz, $CDCl_3$

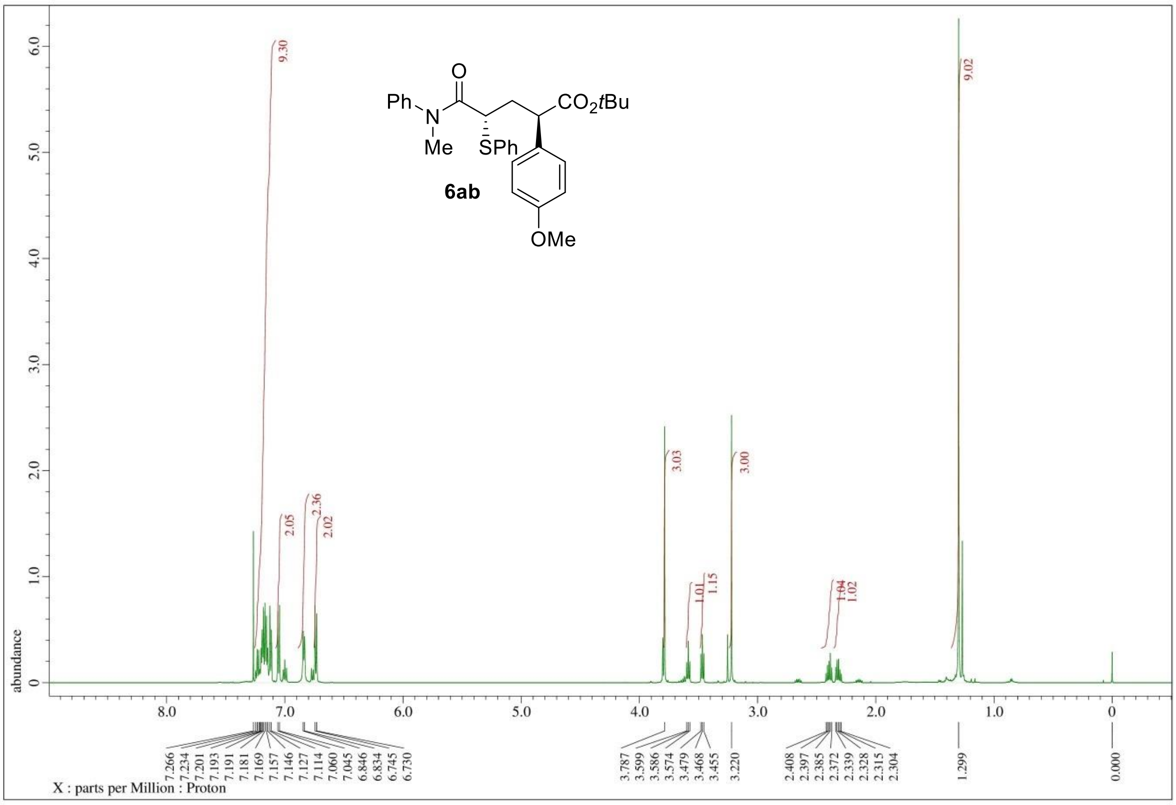

600 MHz, $CDCl_3$

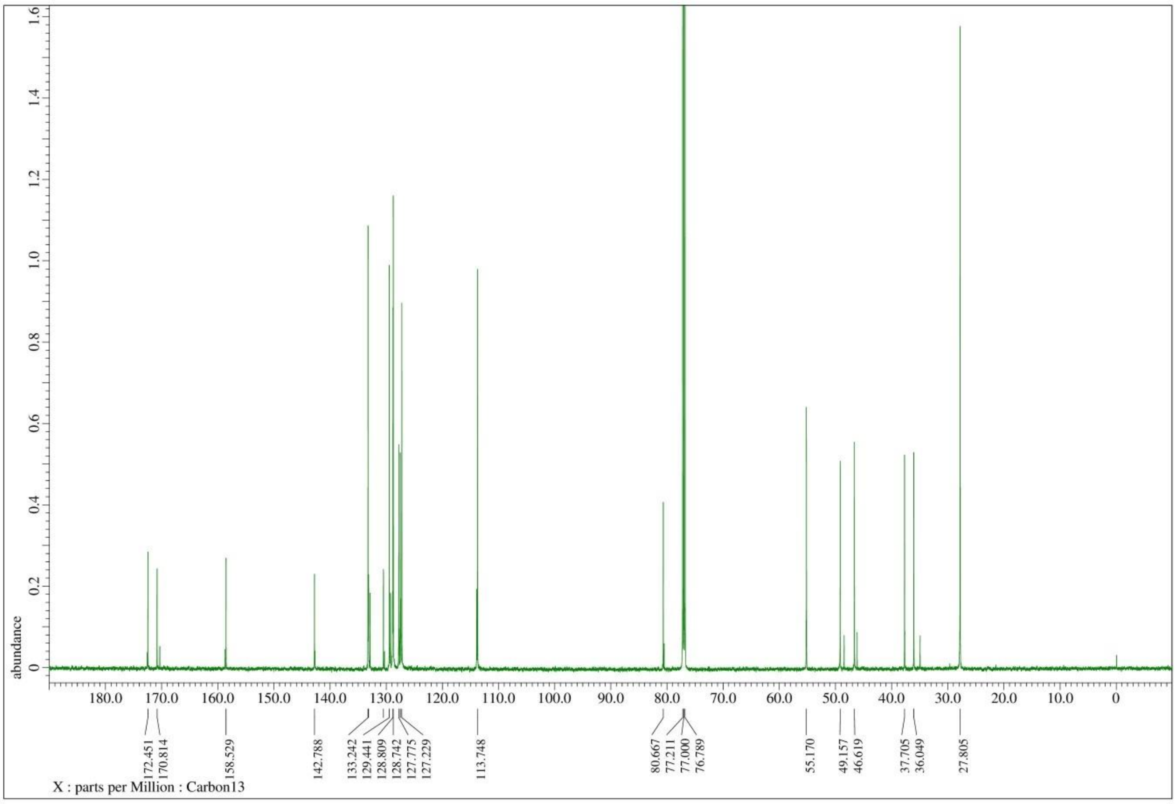

150 MHz, $CDCl_3$

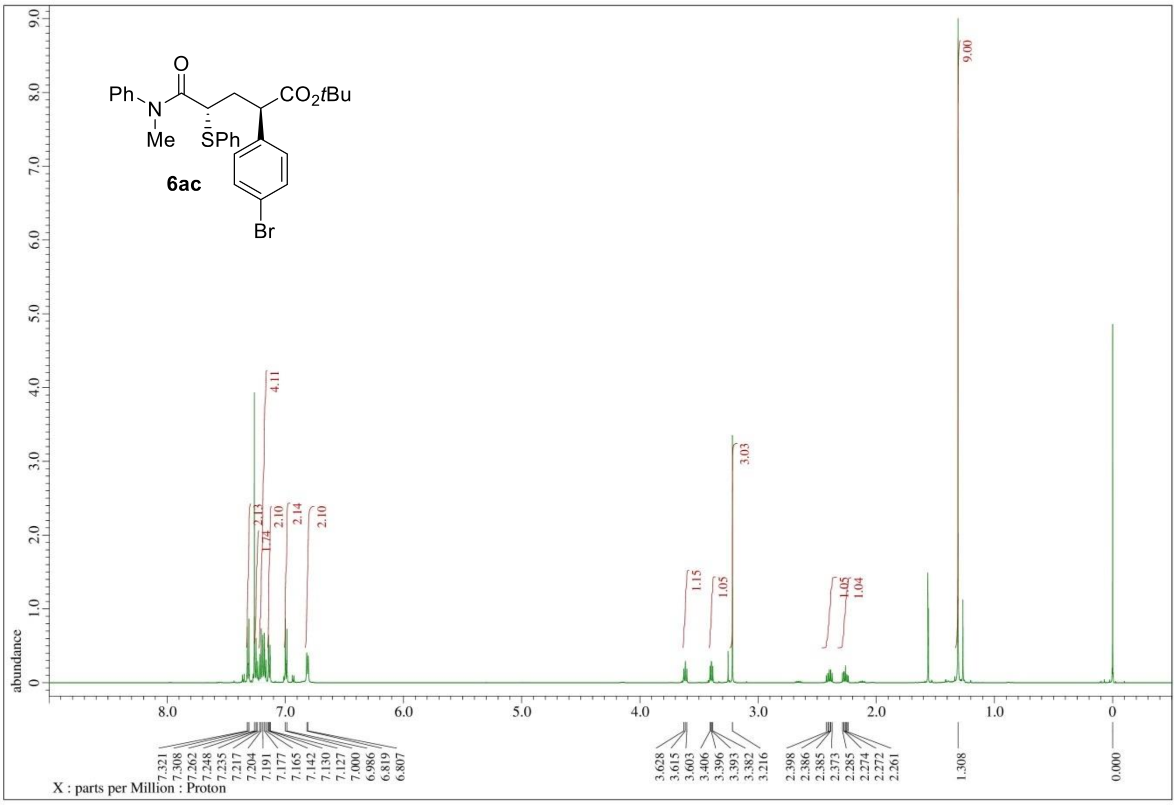

600 MHz, $CDCl_3$

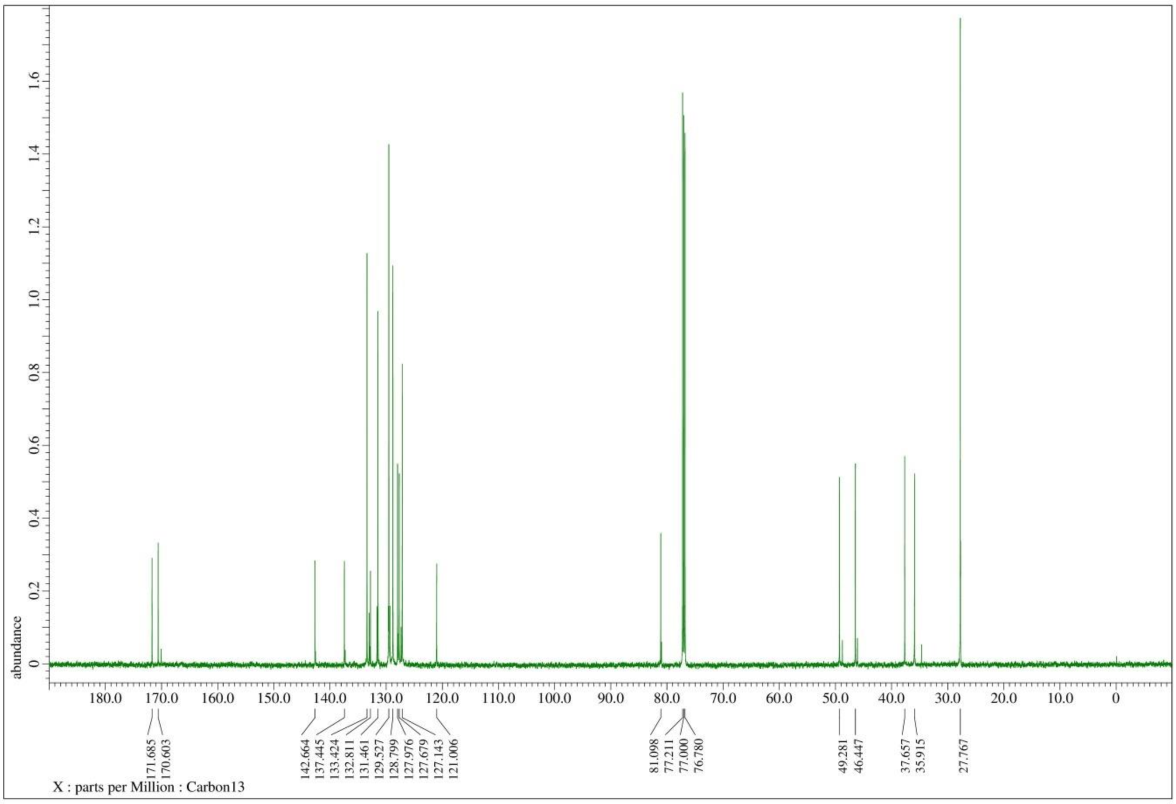

150 MHz, $CDCl_3$

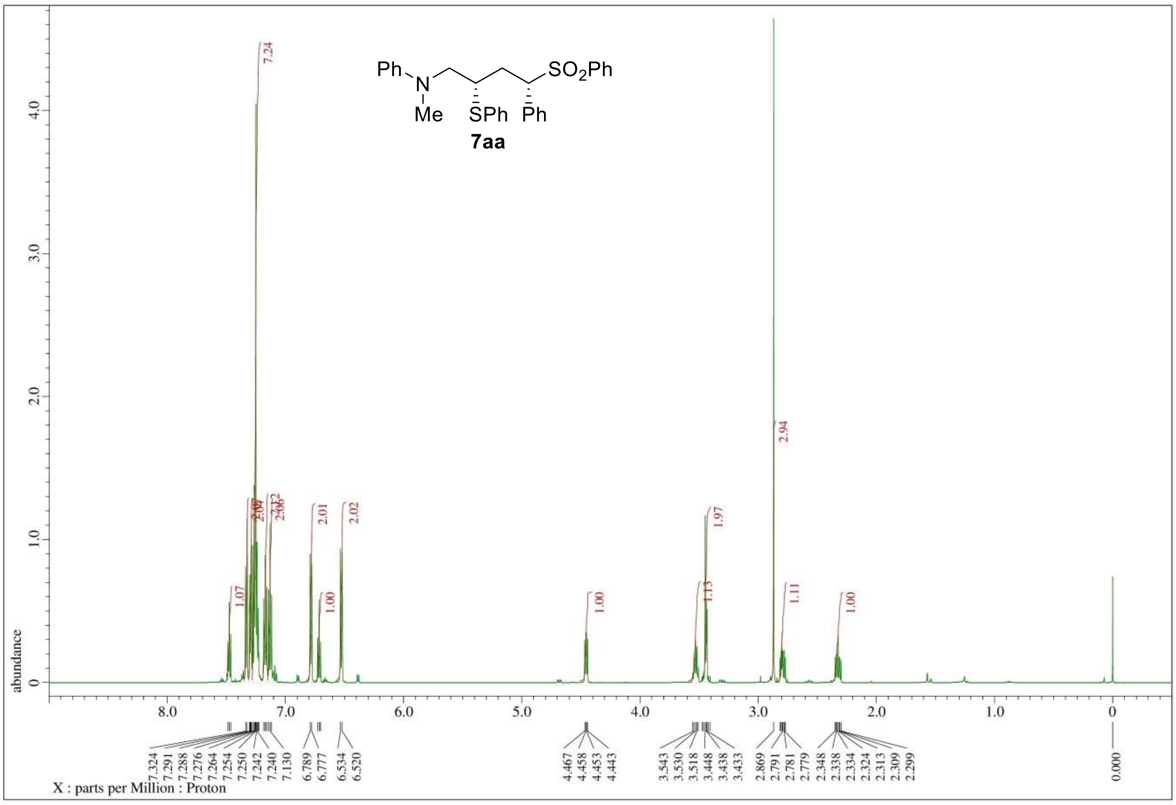

600 MHz, $CDCl_3$

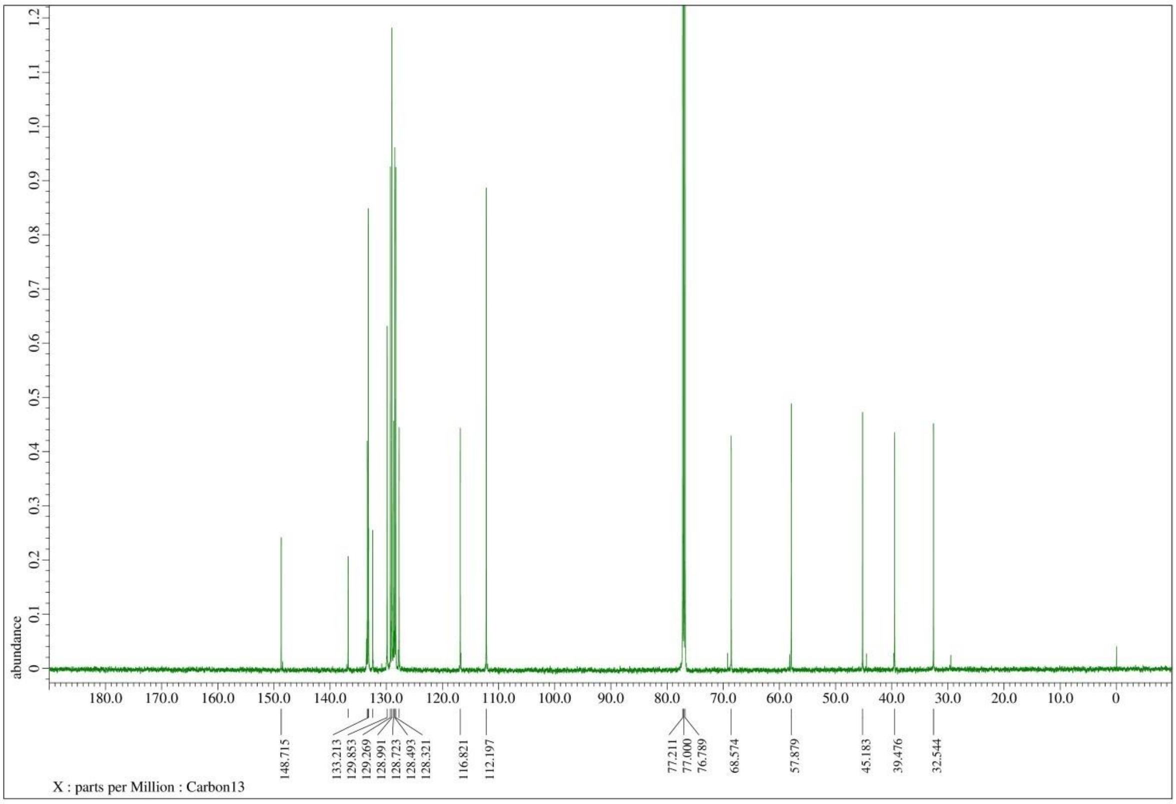

150 MHz, $CDCl_3$

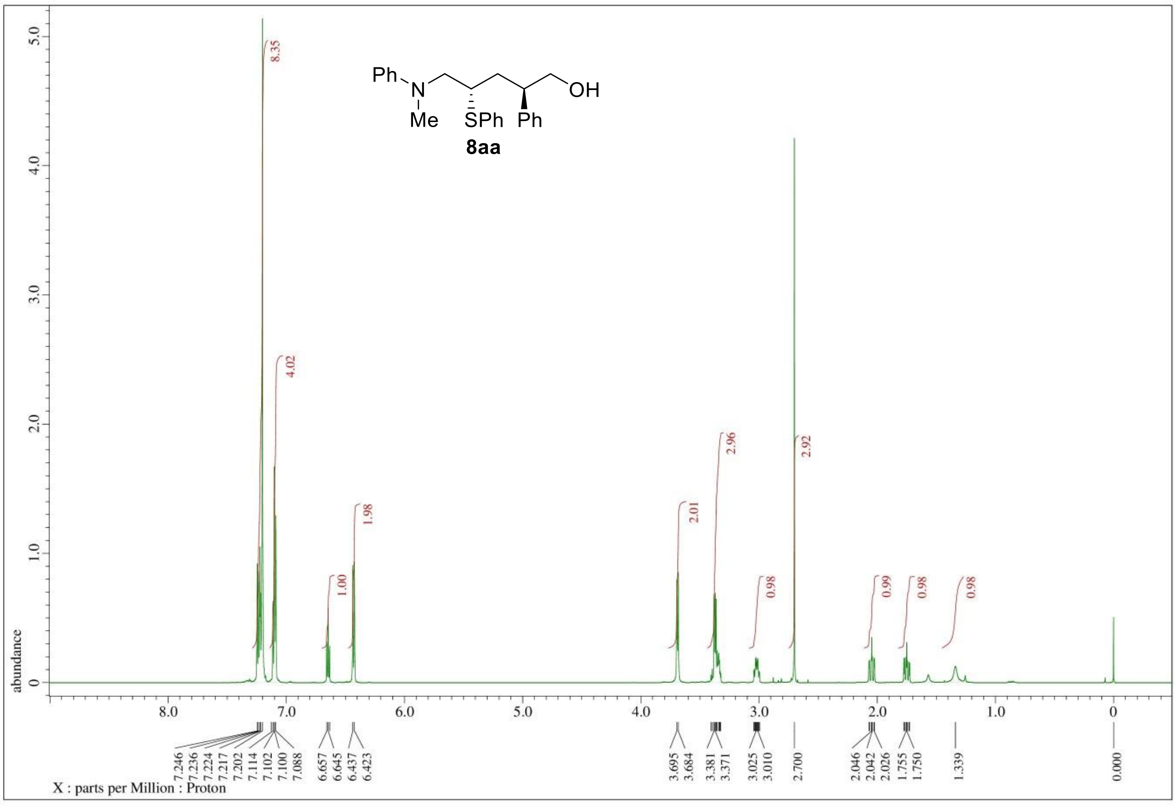

600 MHz, CDCl$_3$

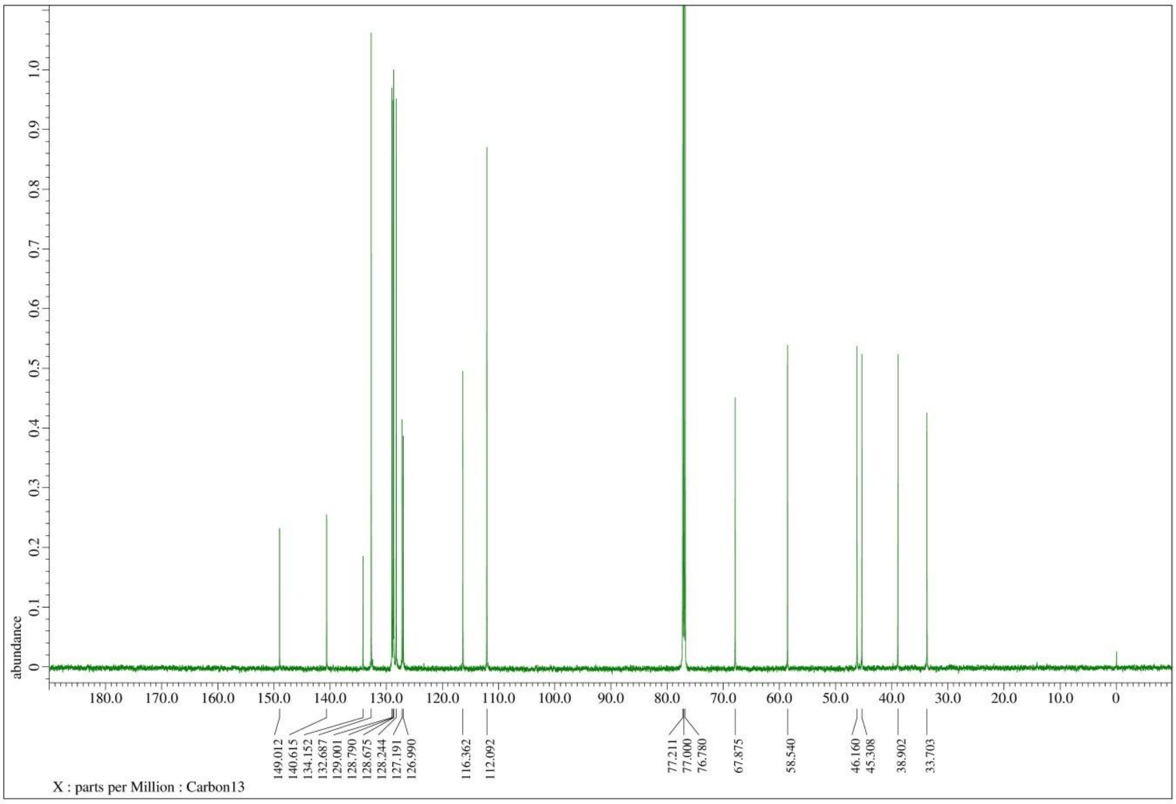

150 MHz, CDCl$_3$

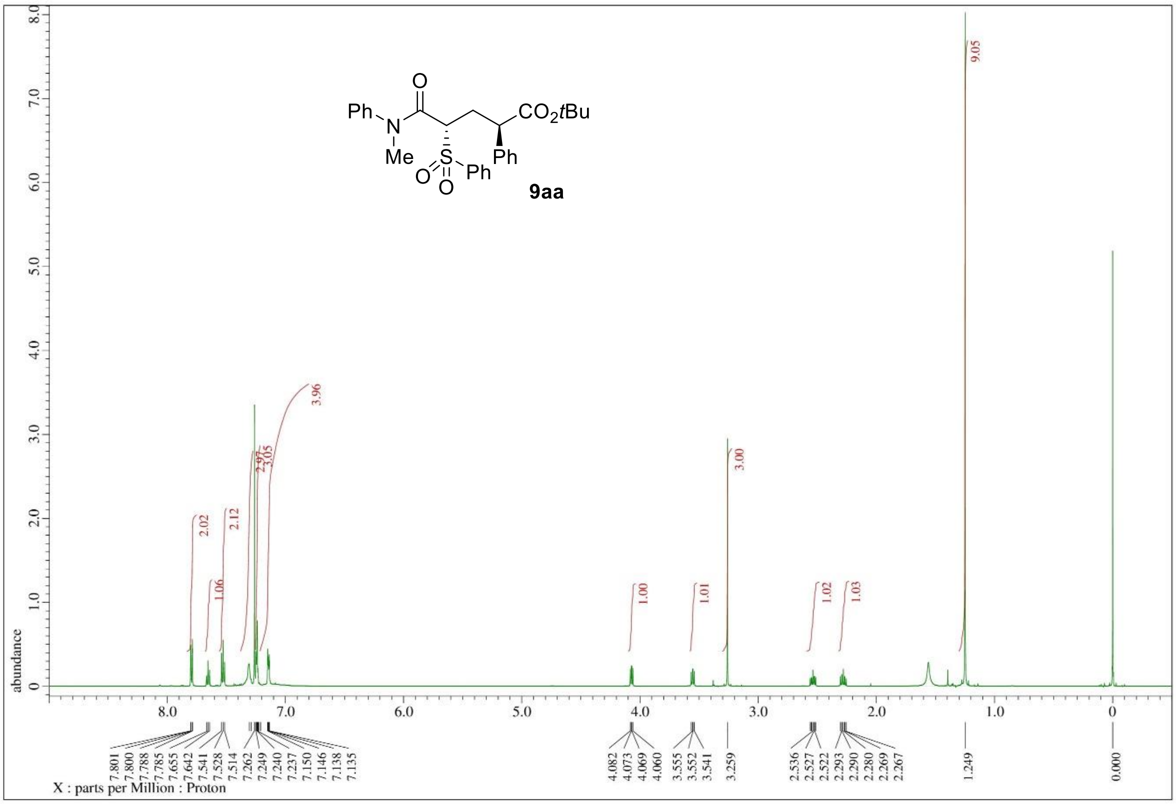

600 MHz, $CDCl_3$

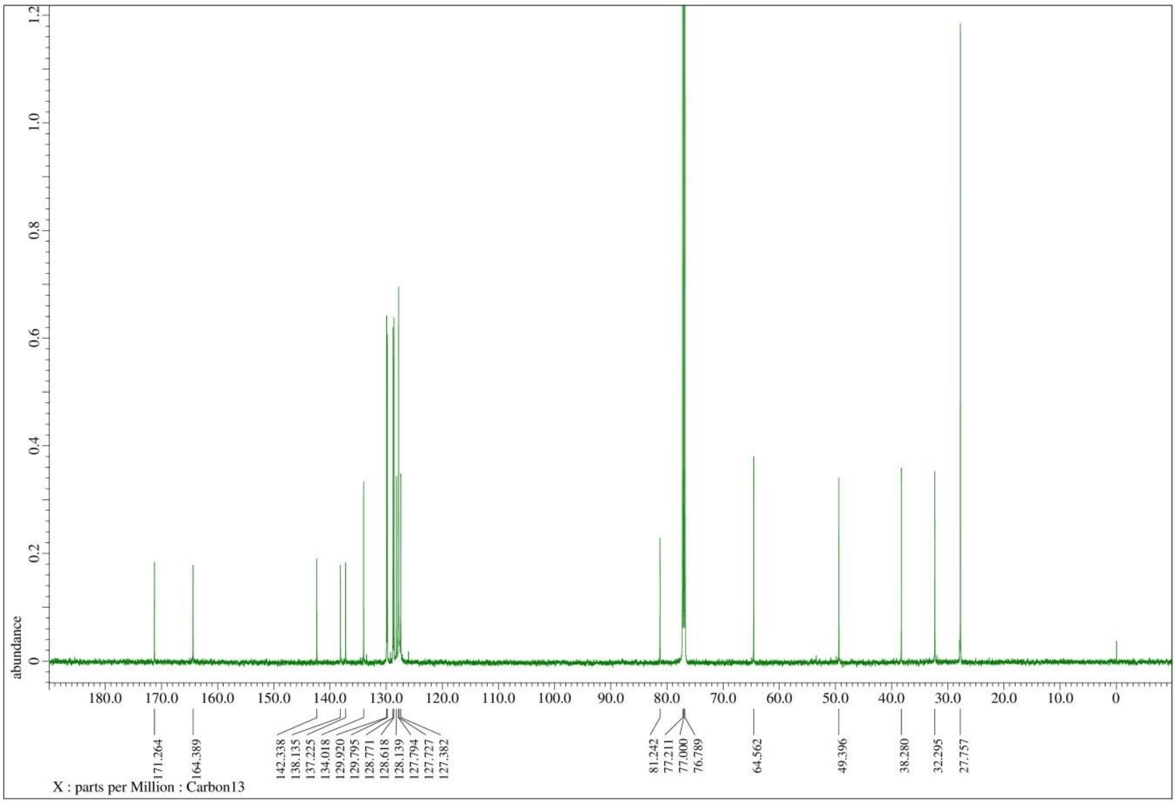

150 MHz, $CDCl_3$

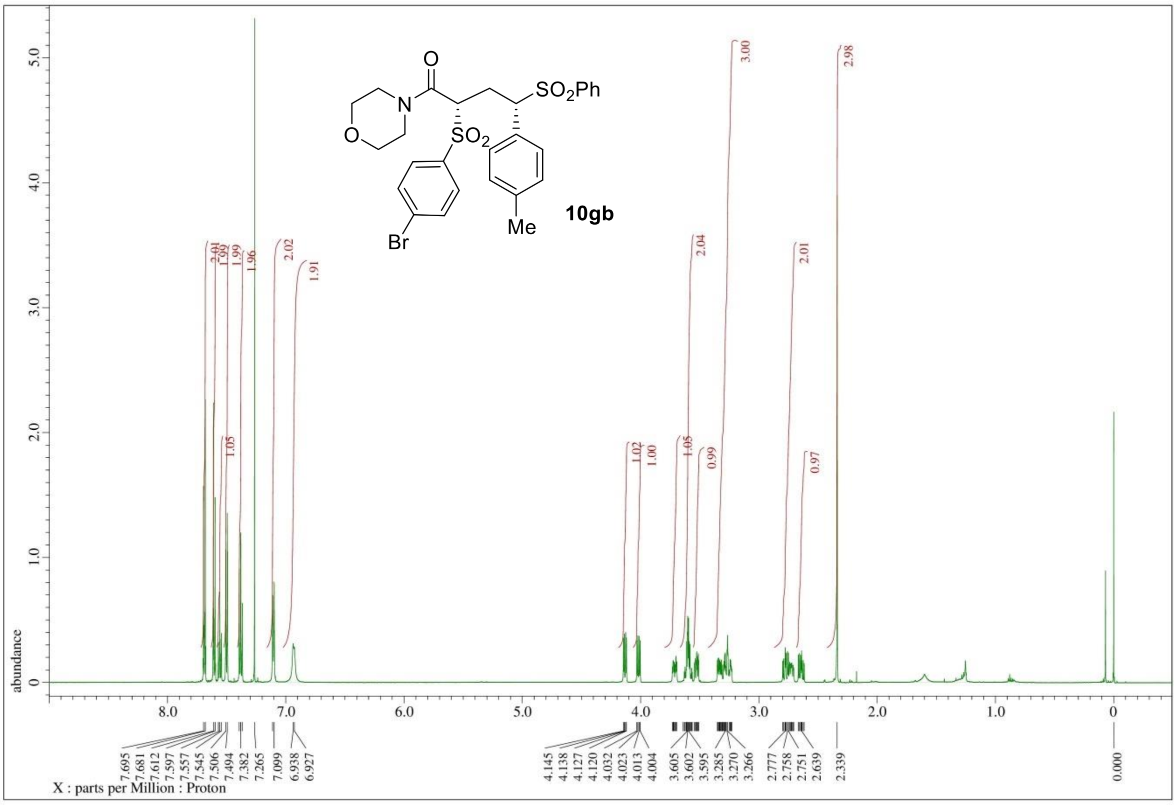

600 MHz, $CDCl_3$

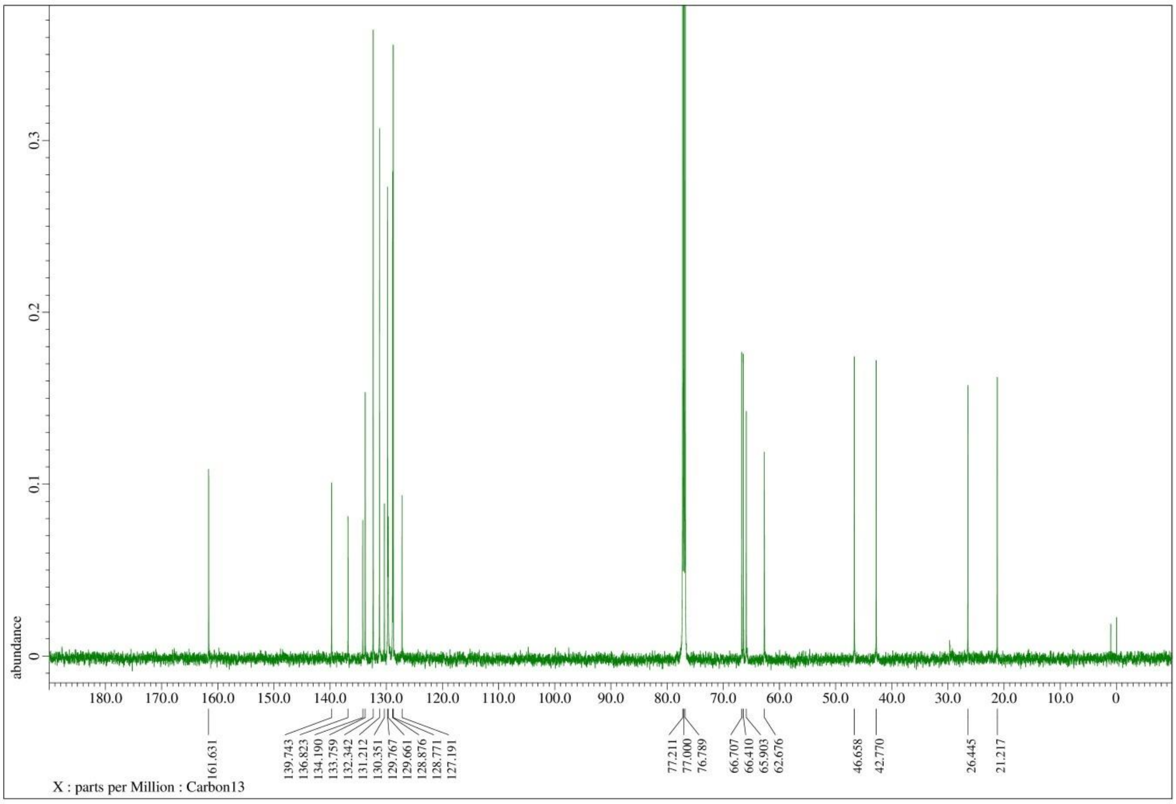

150 MHz, $CDCl_3$

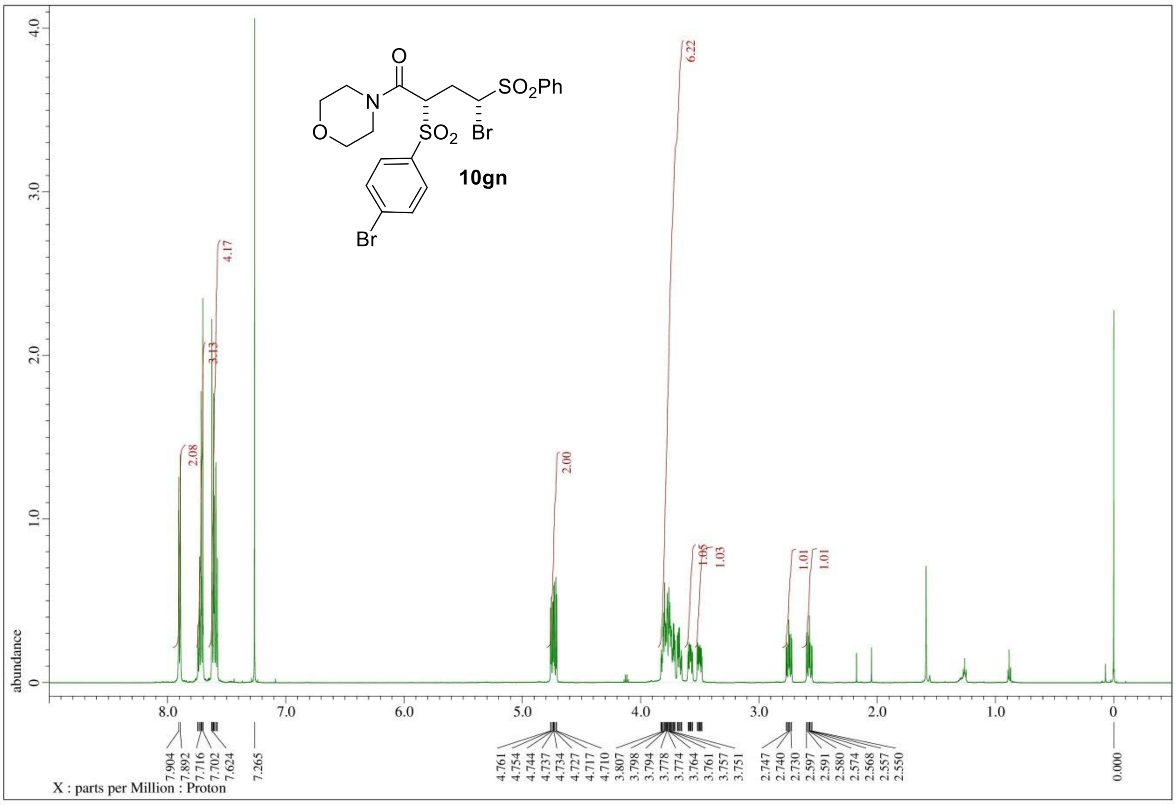

600 MHz, $CDCl_3$

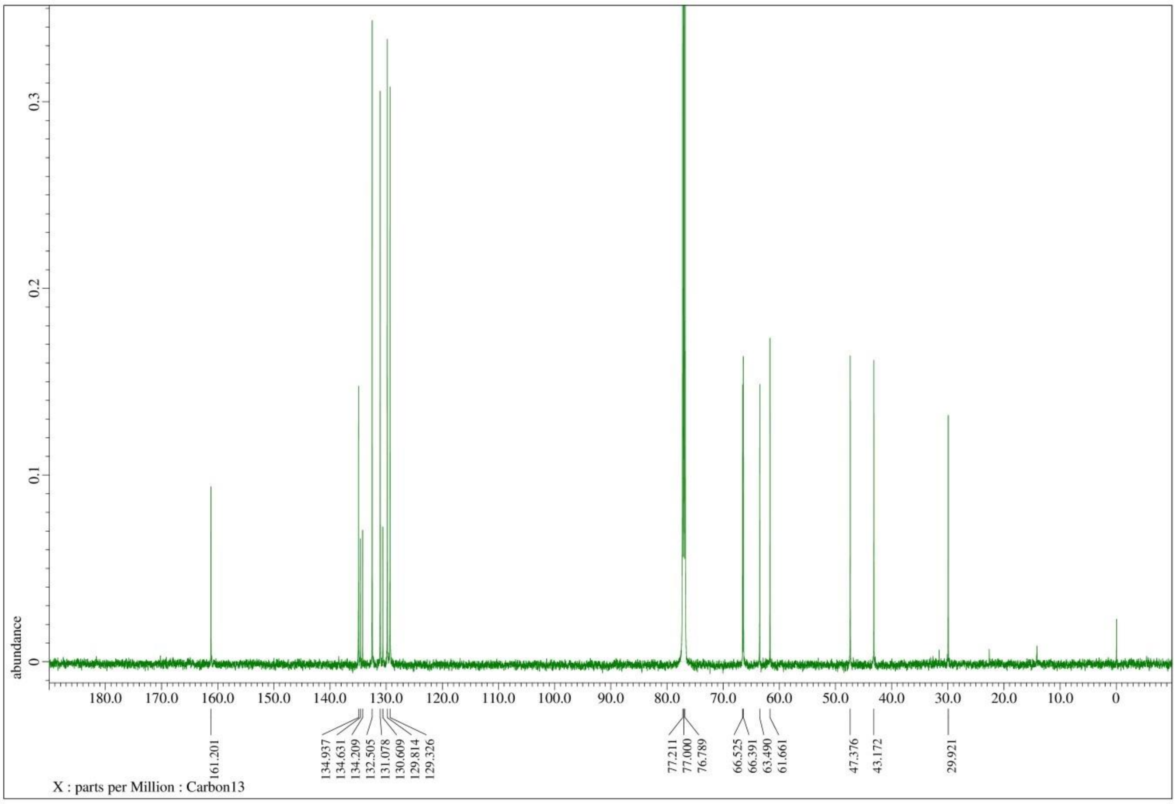

150 MHz, $CDCl_3$

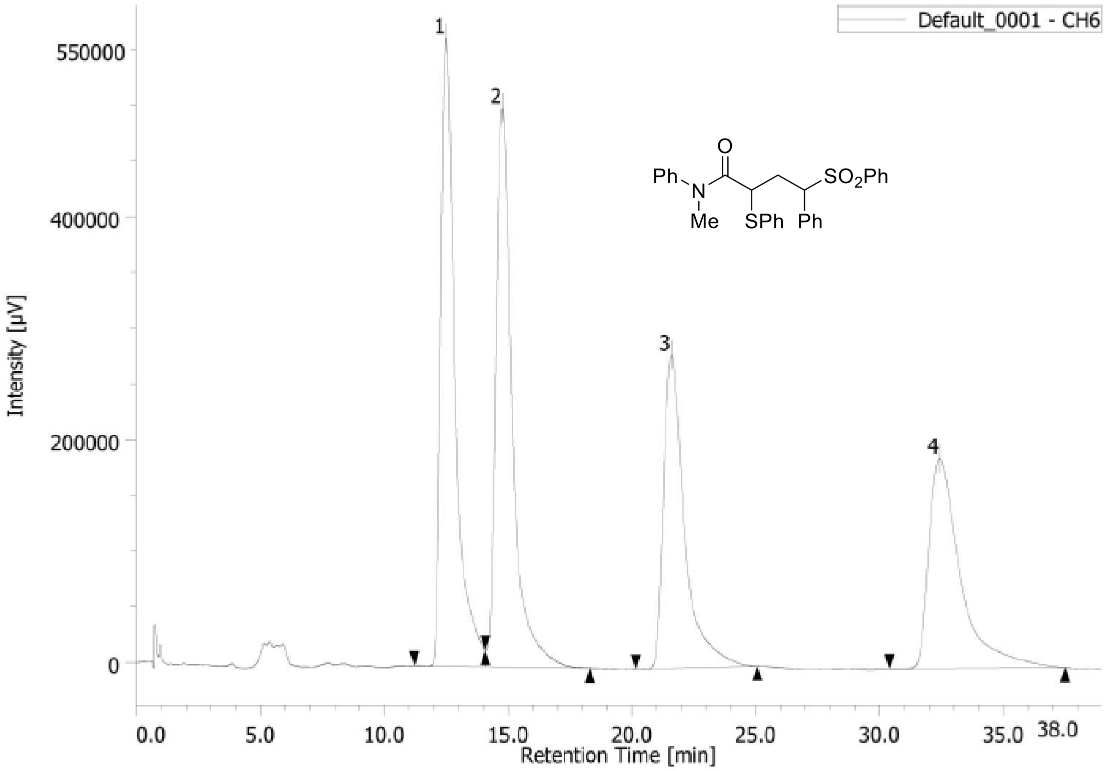

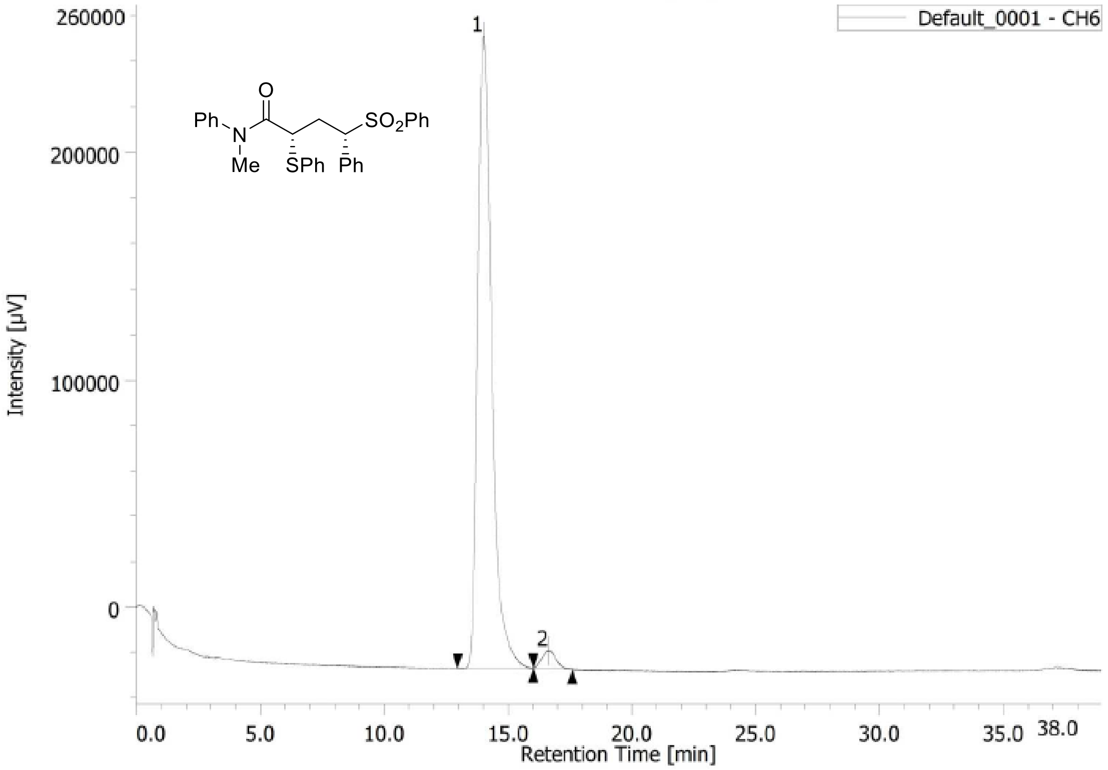

4aa

Peak	1	2
Retention Time (min)	14.0	16.7
Area (%)	97.1	2.9

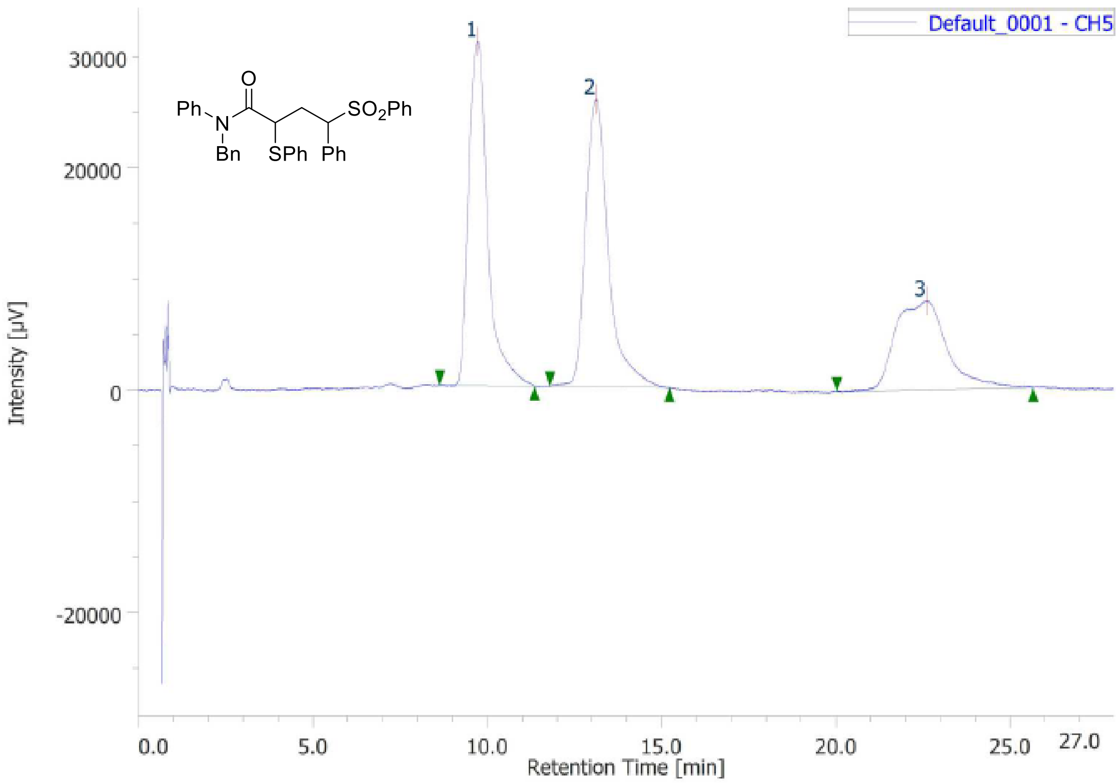

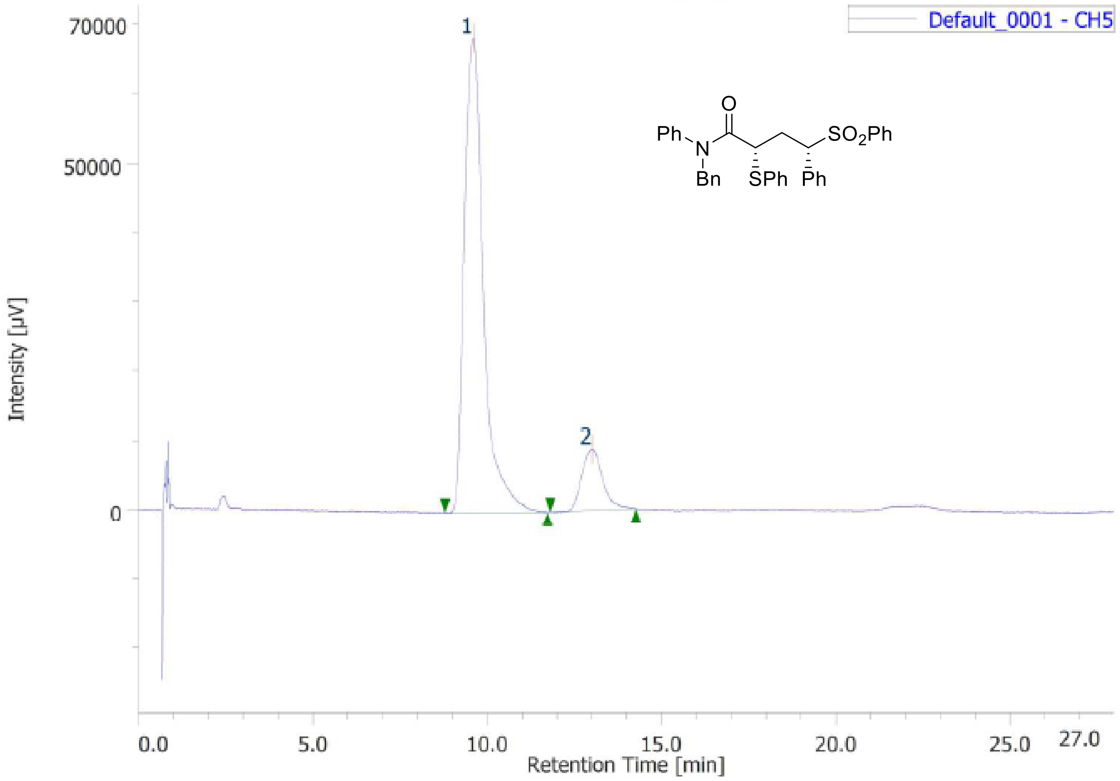

4ba

Peak	1	2
Retention Time (min)	9.6	13.0
Area (%)	87.7	12.3

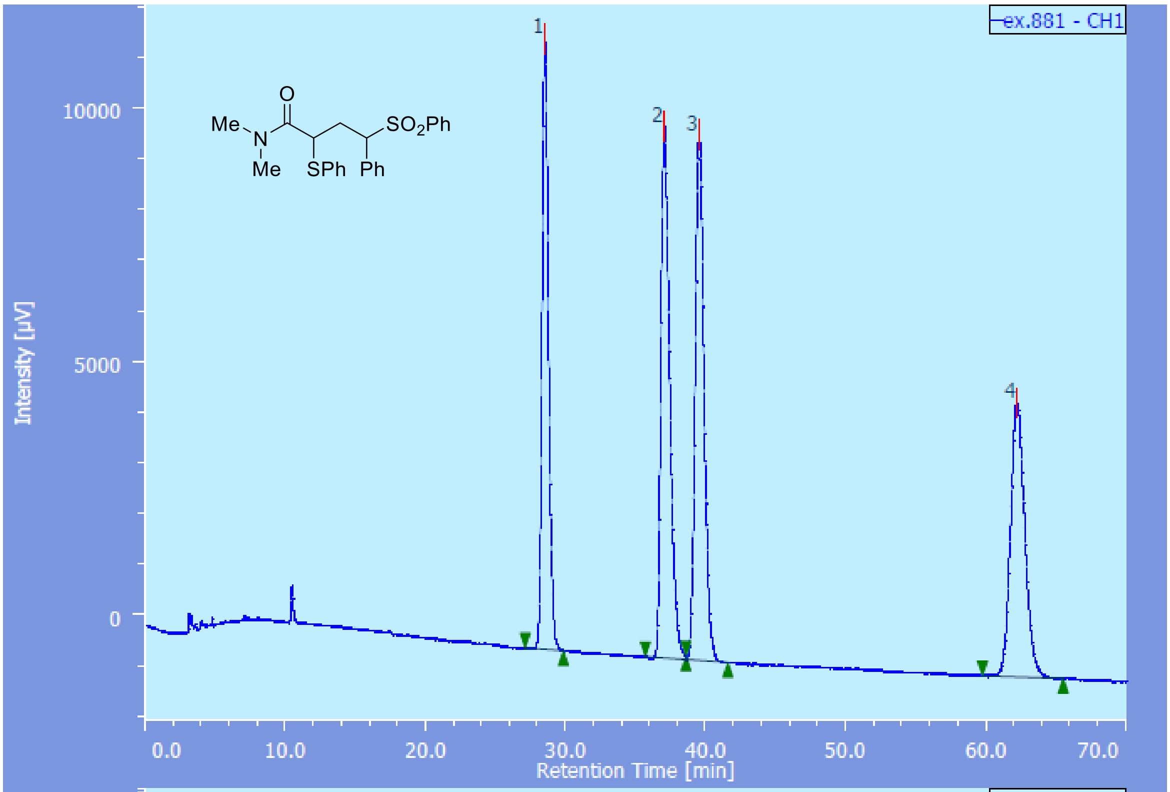

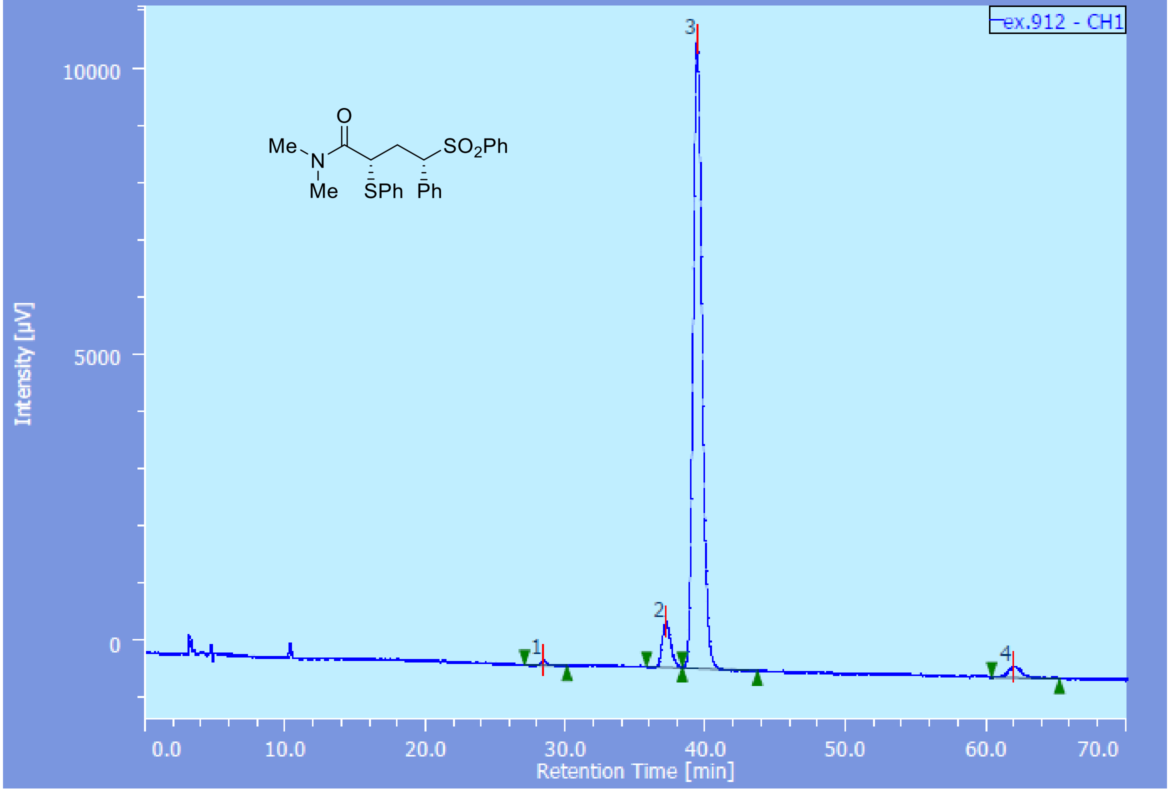

4ca

Peak	2	3
Retention Time (min)	37.2	39.4
Area (%)	6.5	93.5

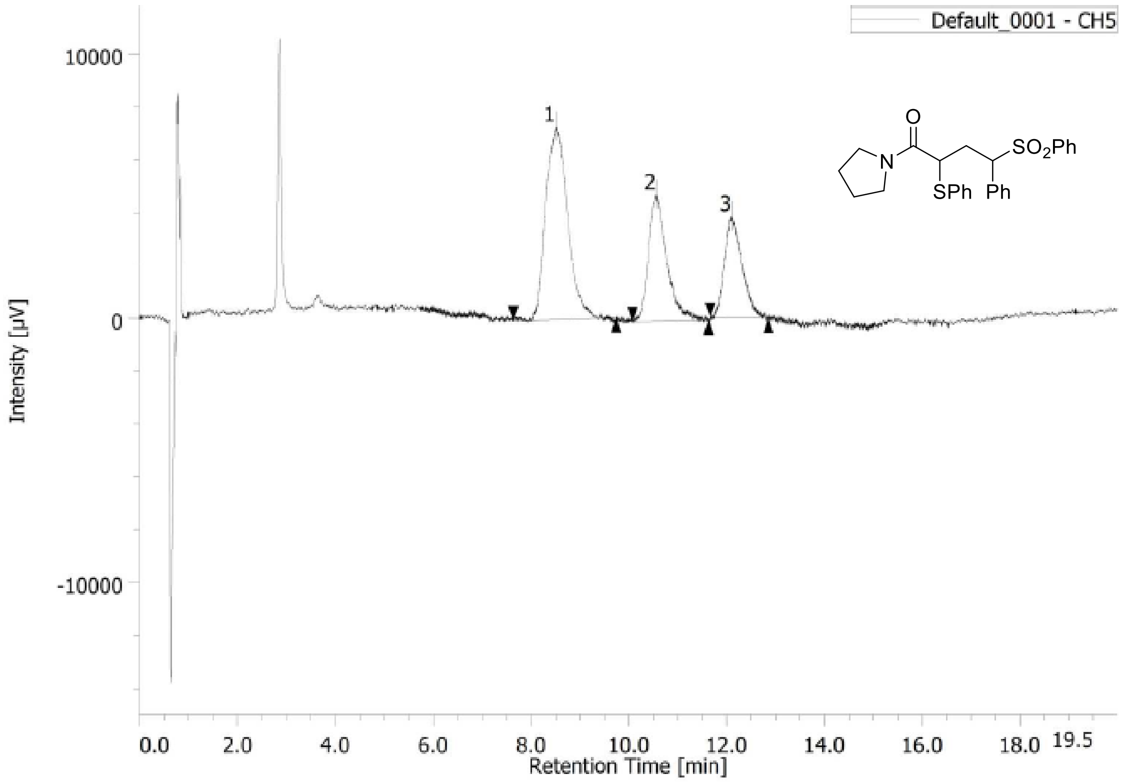

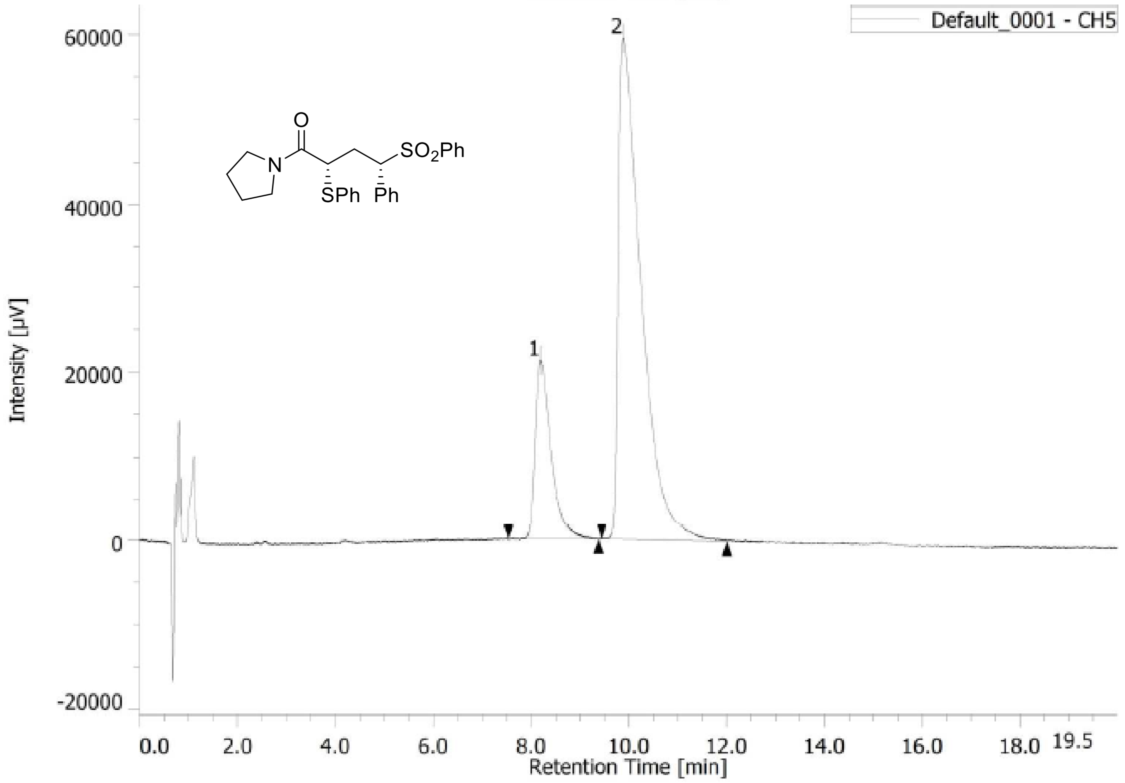

4da

Peak	1	2
Retention Time (min)	8.2	9.9
Area (%)	19.9	80.1

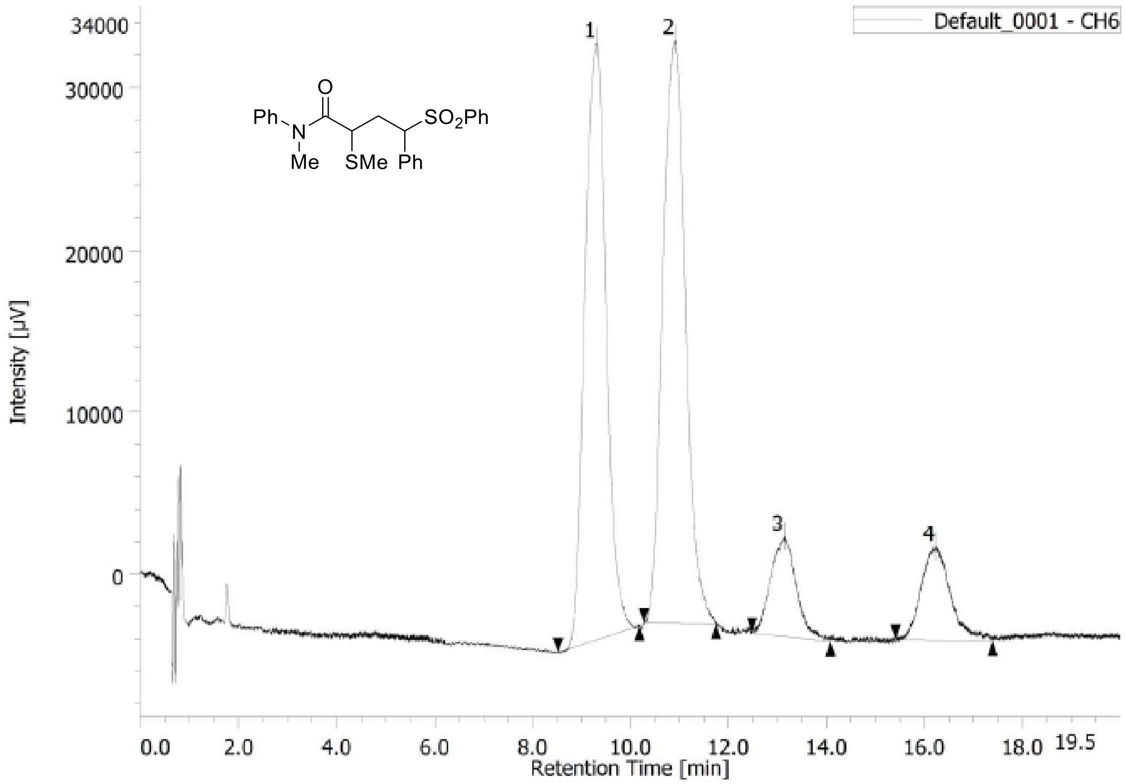

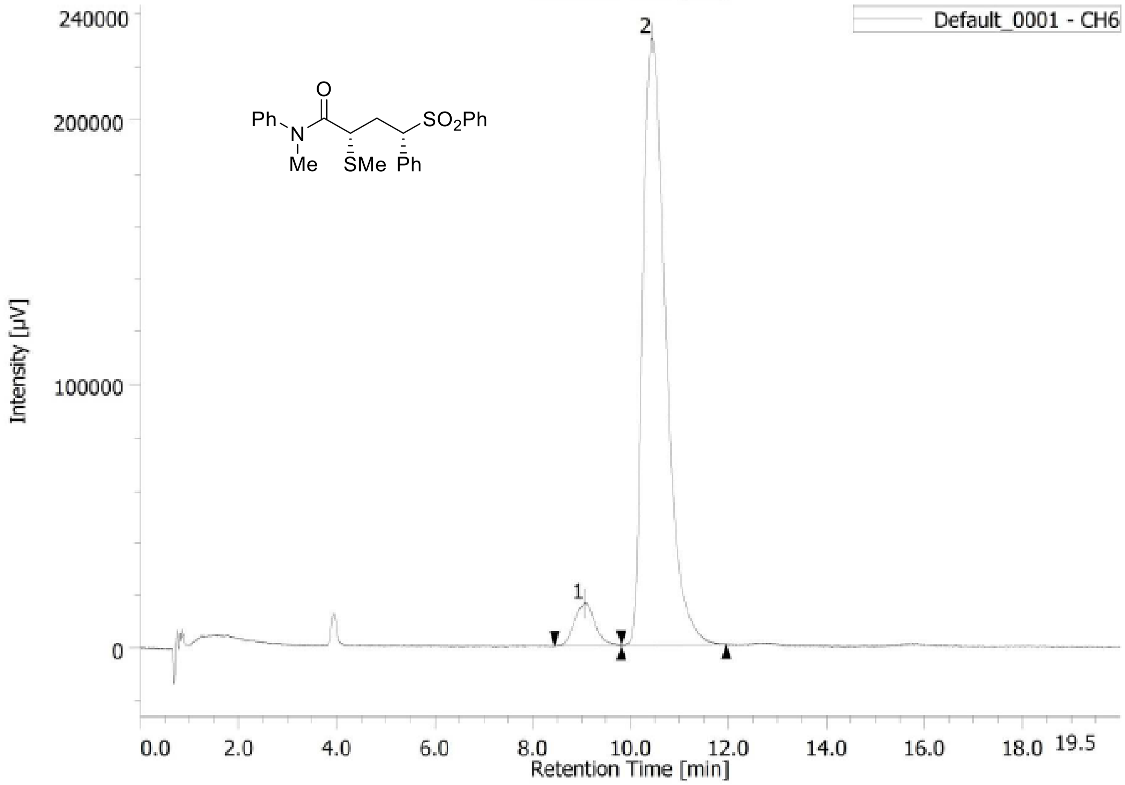

4ea

Peak	1	2
Retention Time (min)	9.1	10.4
Area (%)	5.9	94.1

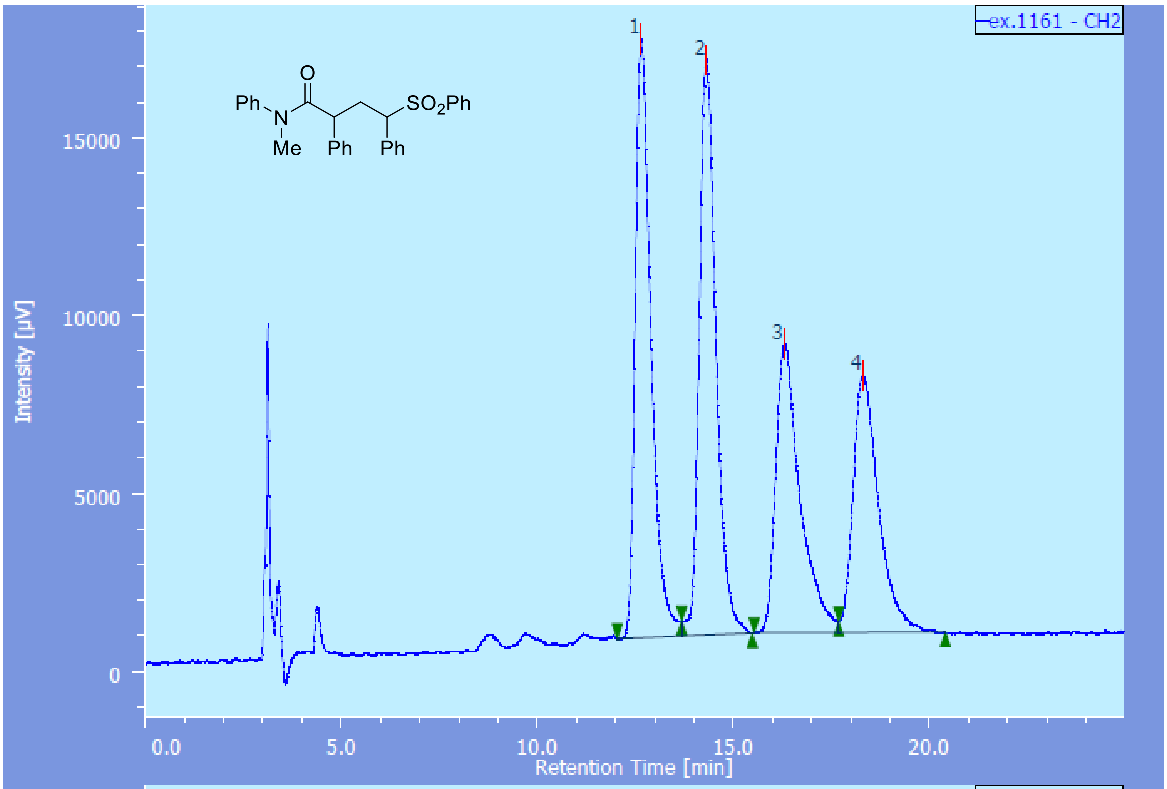

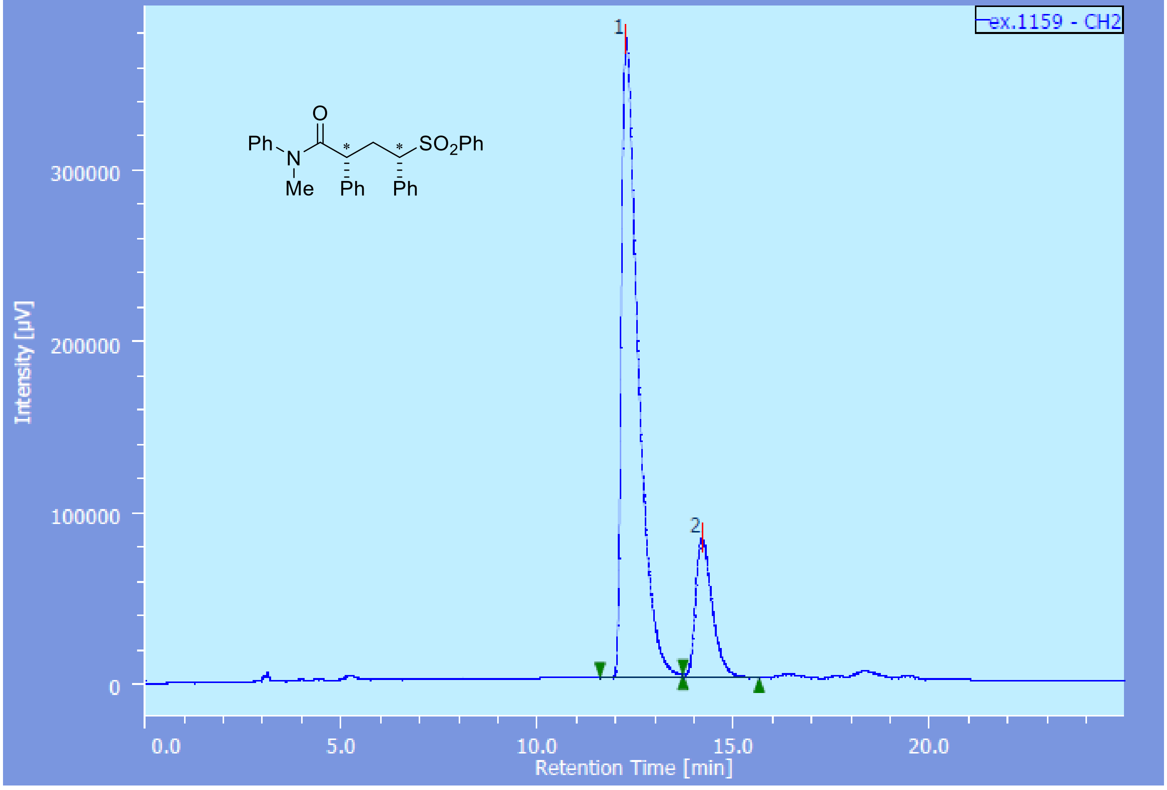

4fa

Peak	1	2
Retention Time (min)	12.3	14.2
Area (%)	82.0	18.0

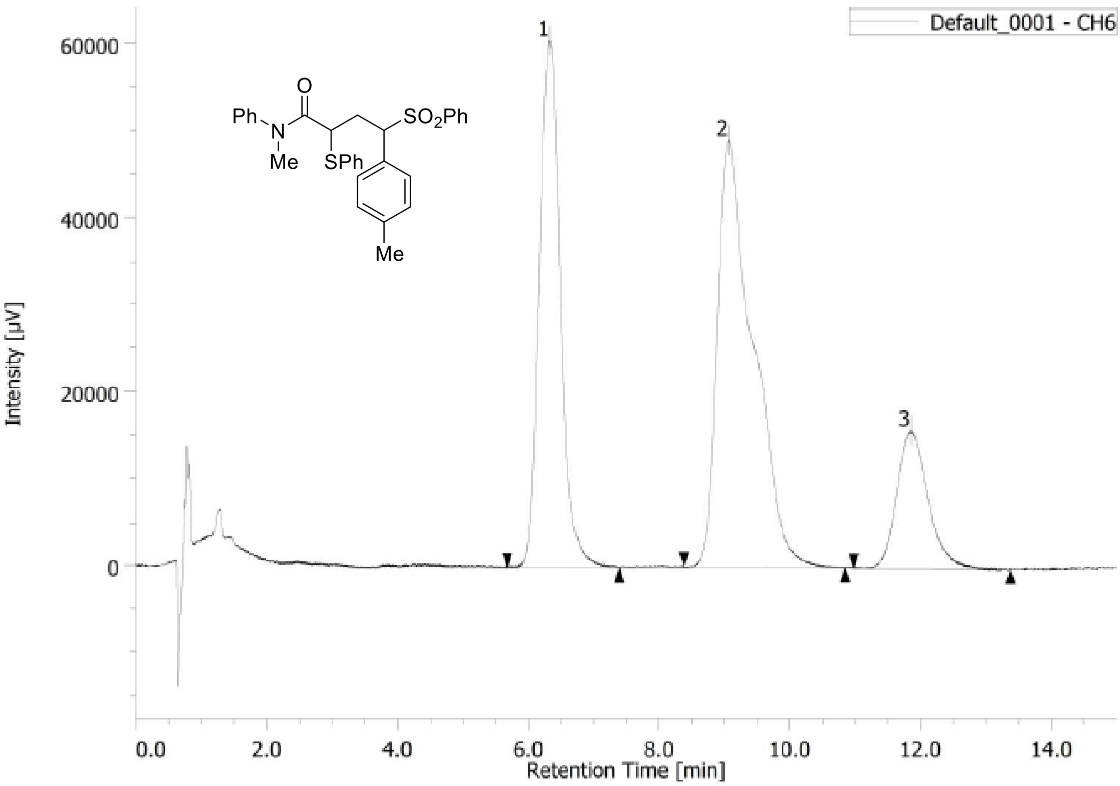

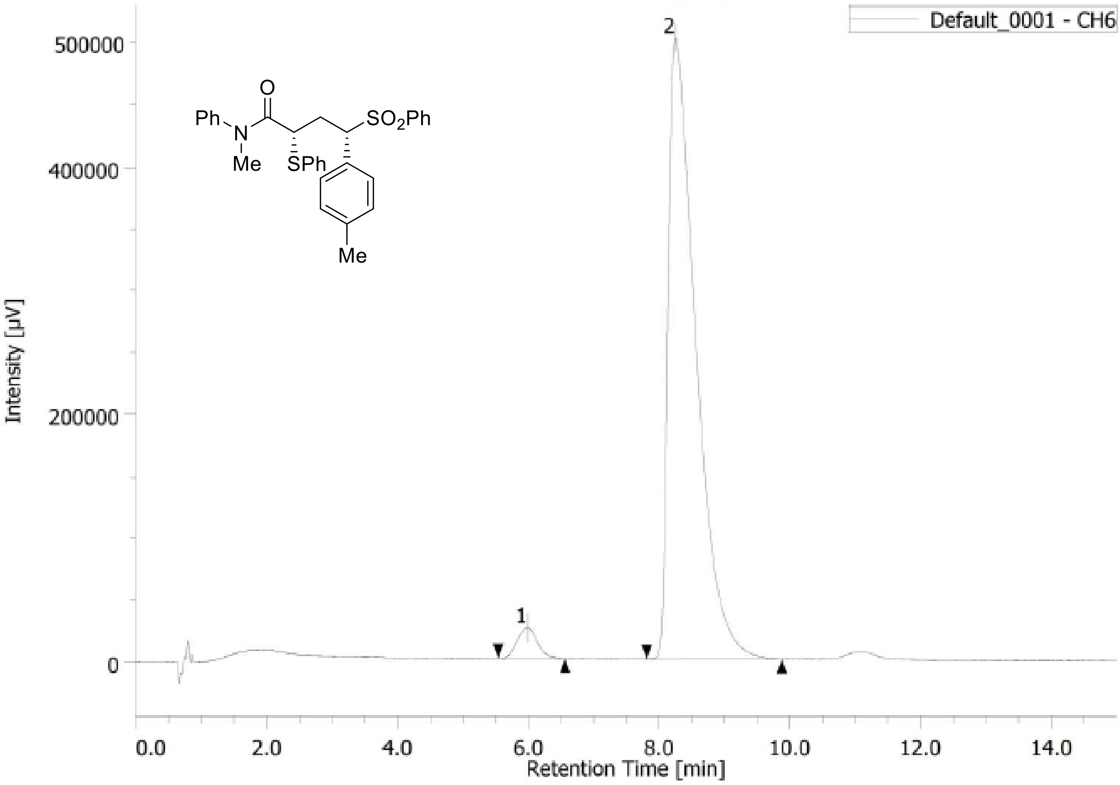

4ab

Peak	1	2
Retention Time (min)	6.0	8.2
Area (%)	3.5	96.5

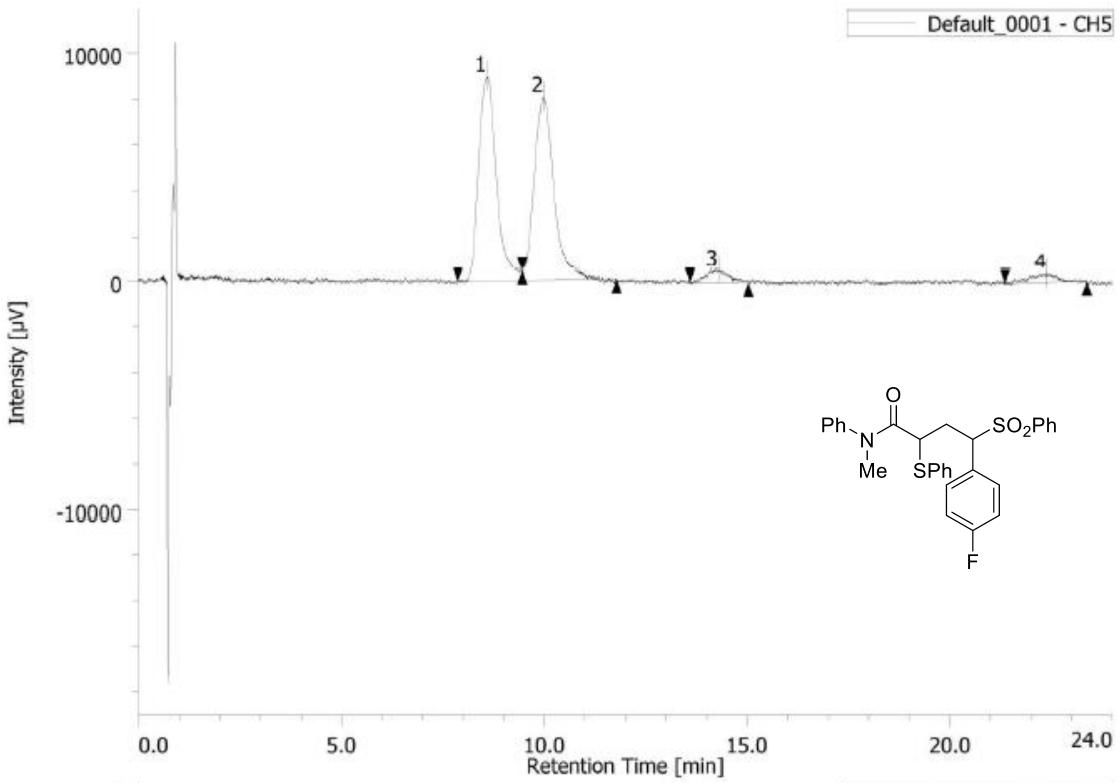

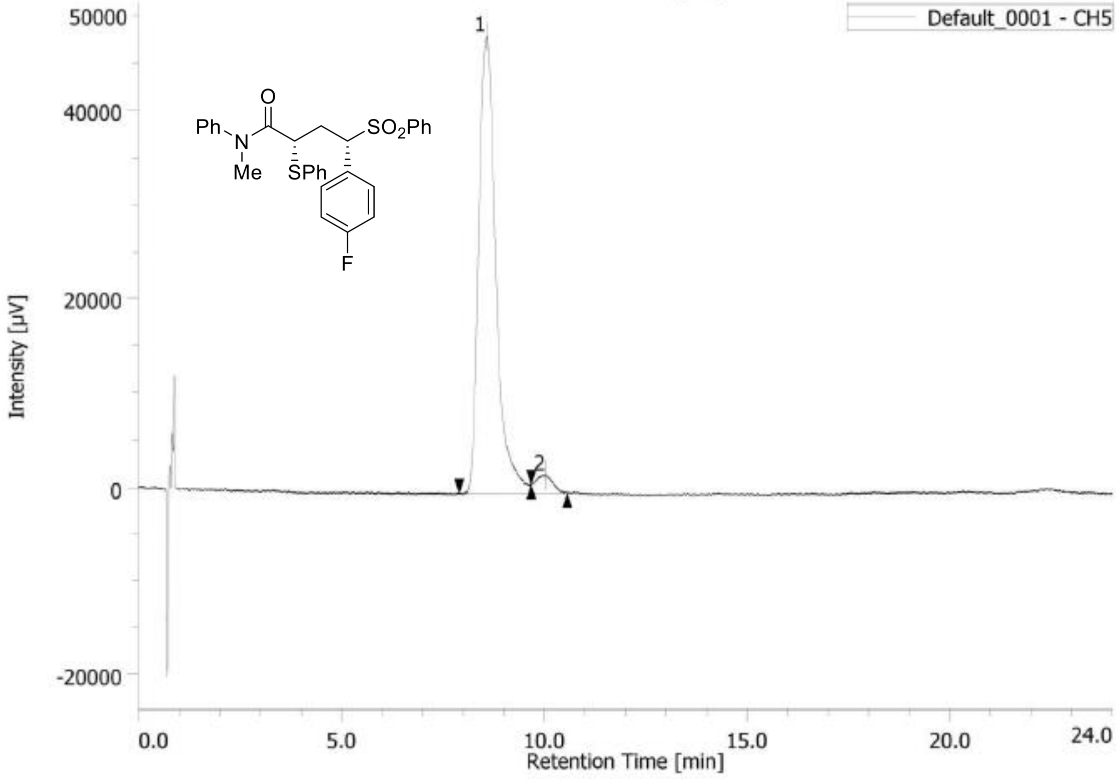

4ac

Peak	1	2
Retention Time (min)	8.6	10.0
Area (%)	96.2	3.8

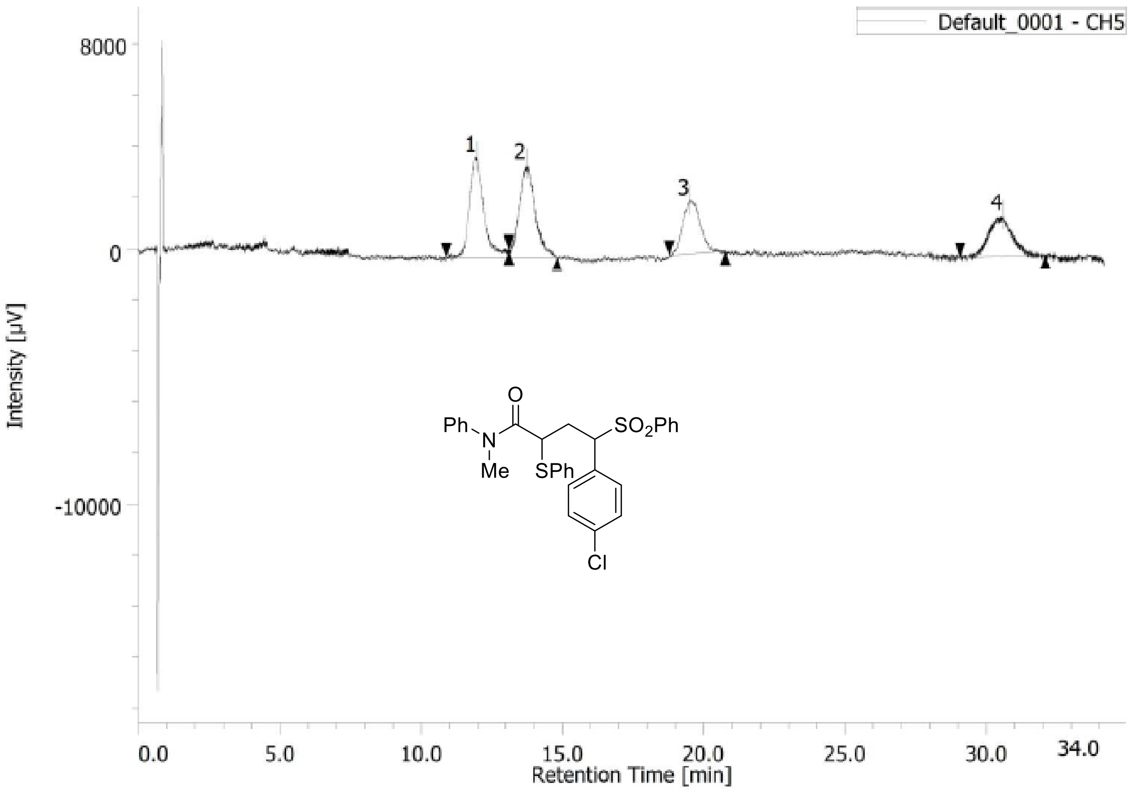

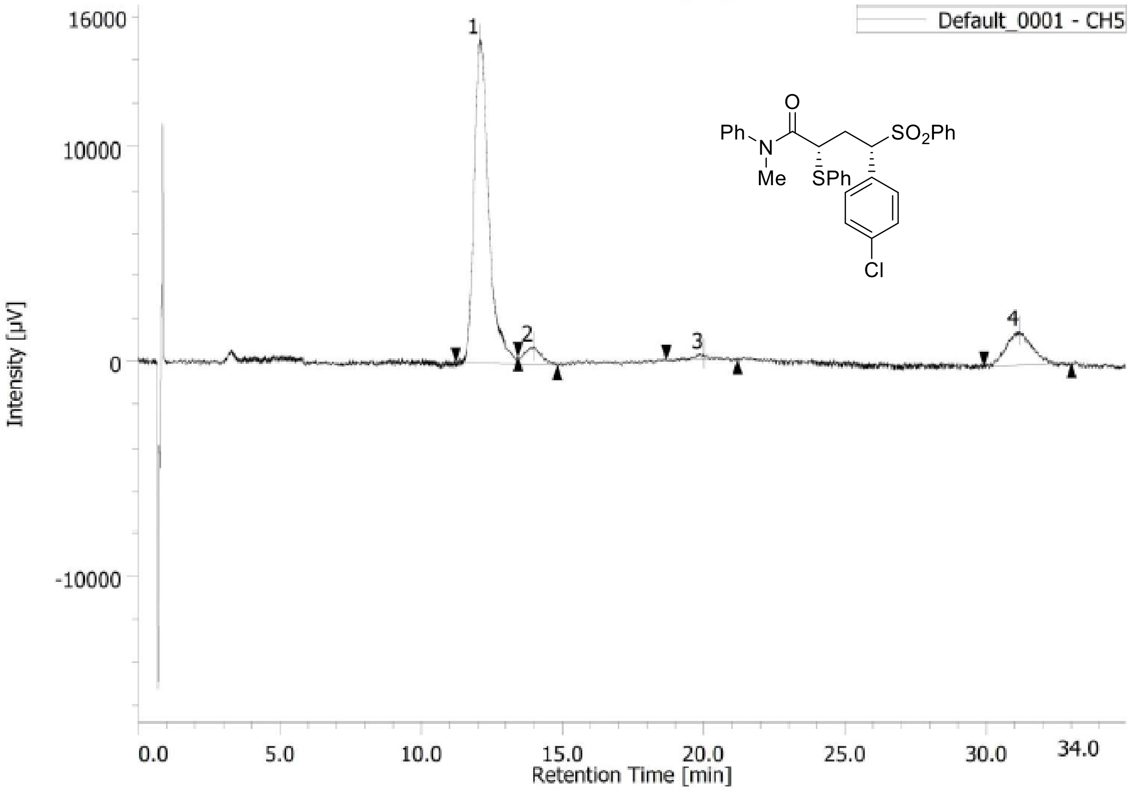

4ad

Peak	1	2
Retention Time (min)	12.1	14.0
Area (%)	94.0	6.0

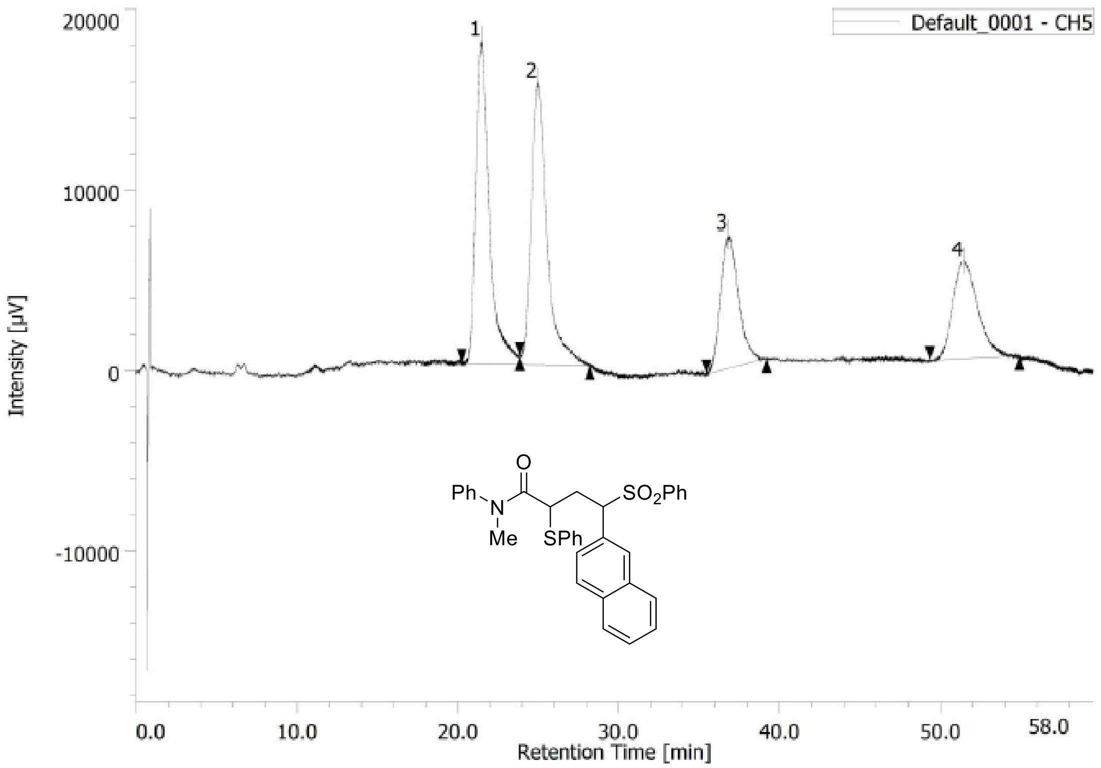

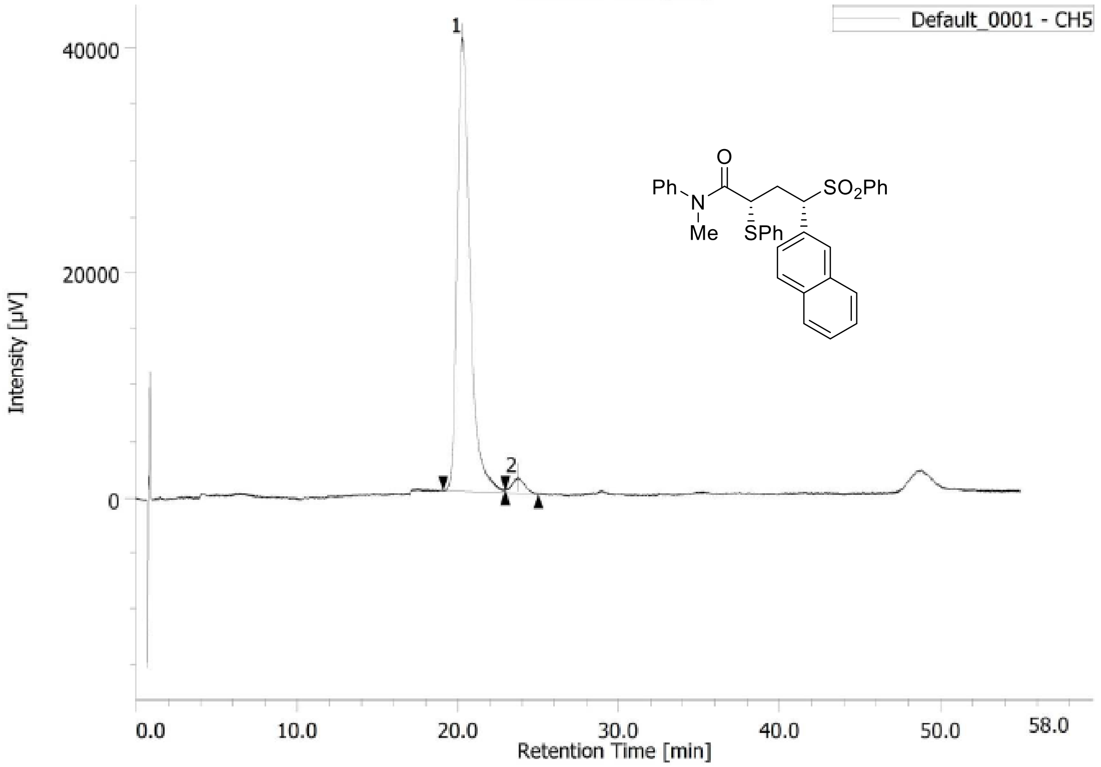

4ae

Peak	1	2
Retention Time (min)	20.3	23.7
Area (%)	96.7	3.3

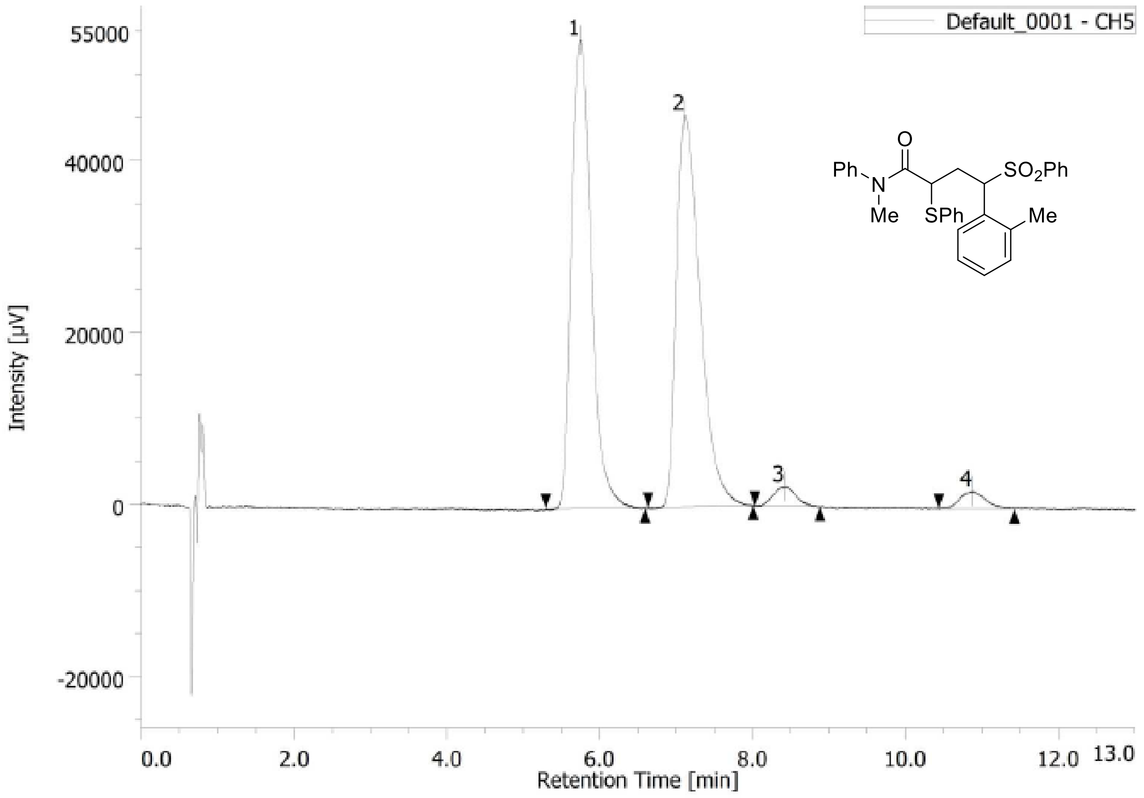

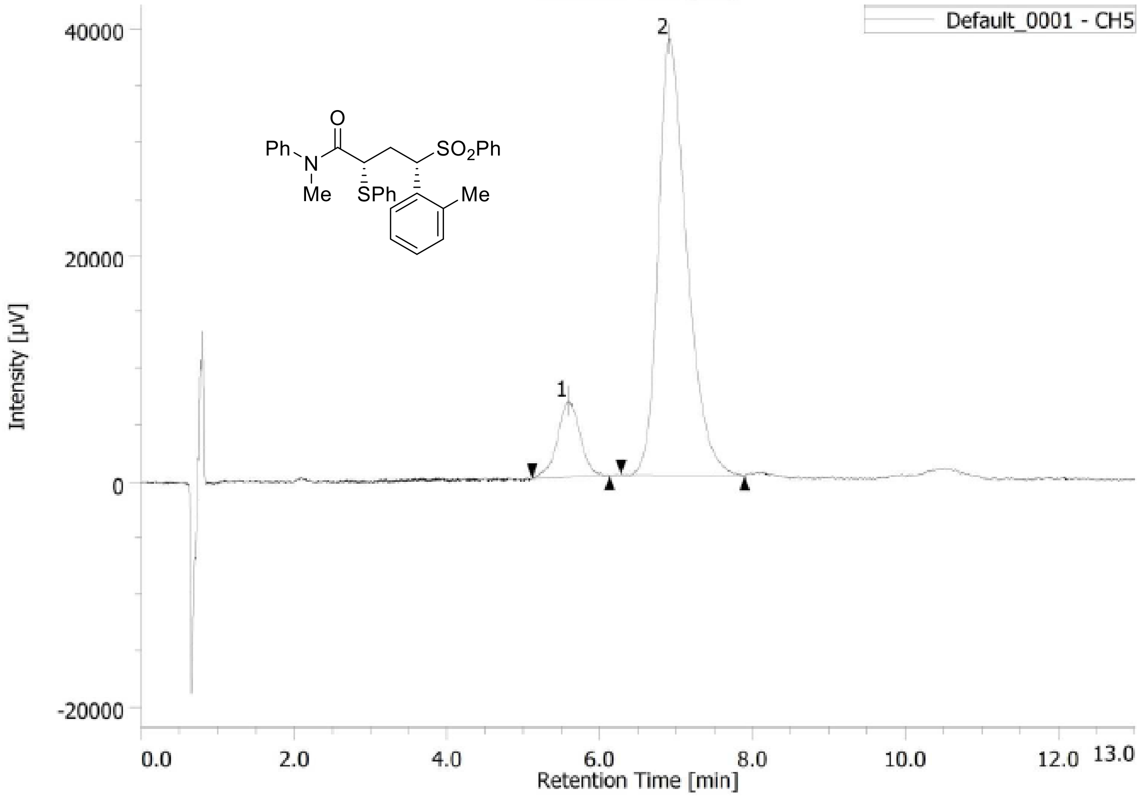

4af

Peak	1	2
Retention Time (min)	5.6	6.9
Area (%)	12.0	88.0

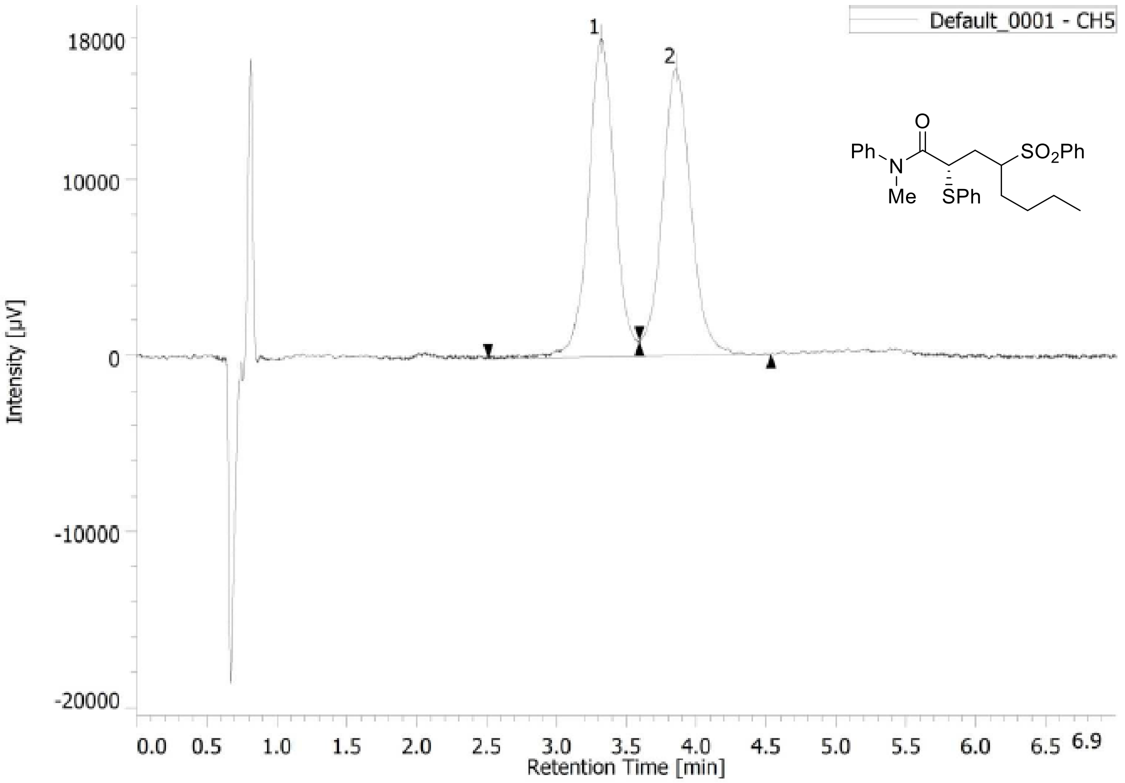

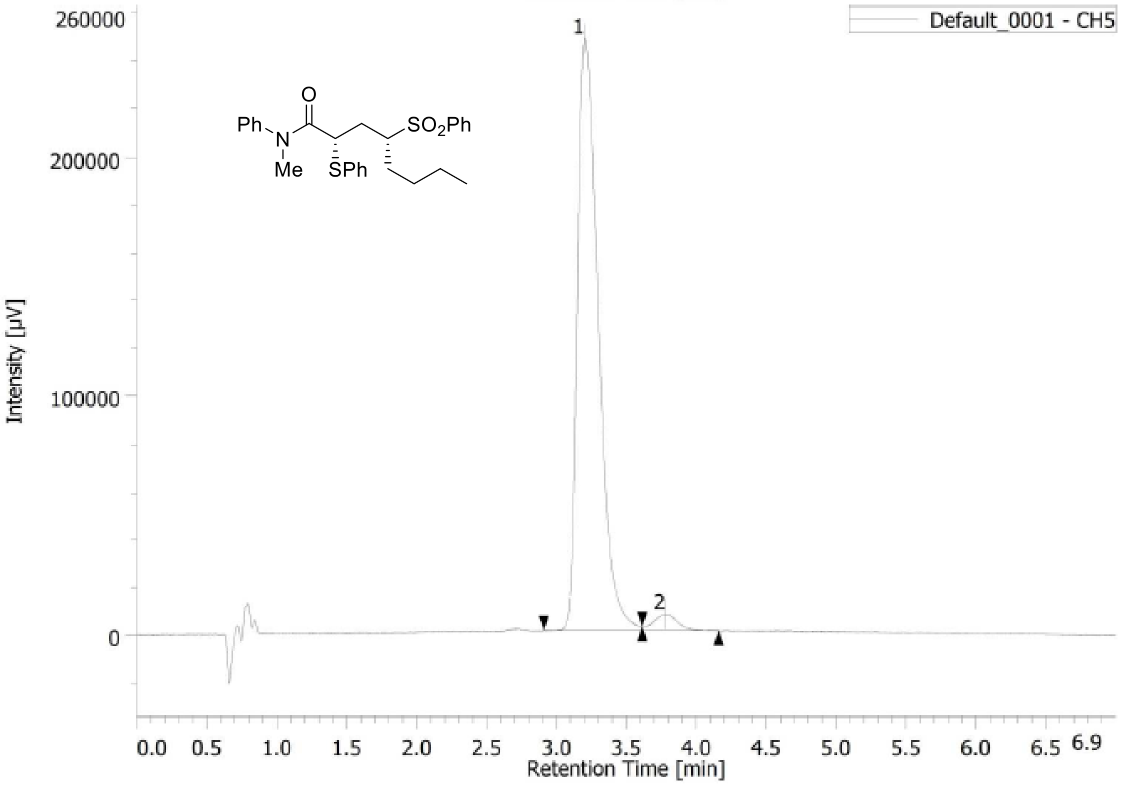

4ag

Peak	1	2
Retention Time (min)	3.2	3.8
Area (%)	97.0	3.0

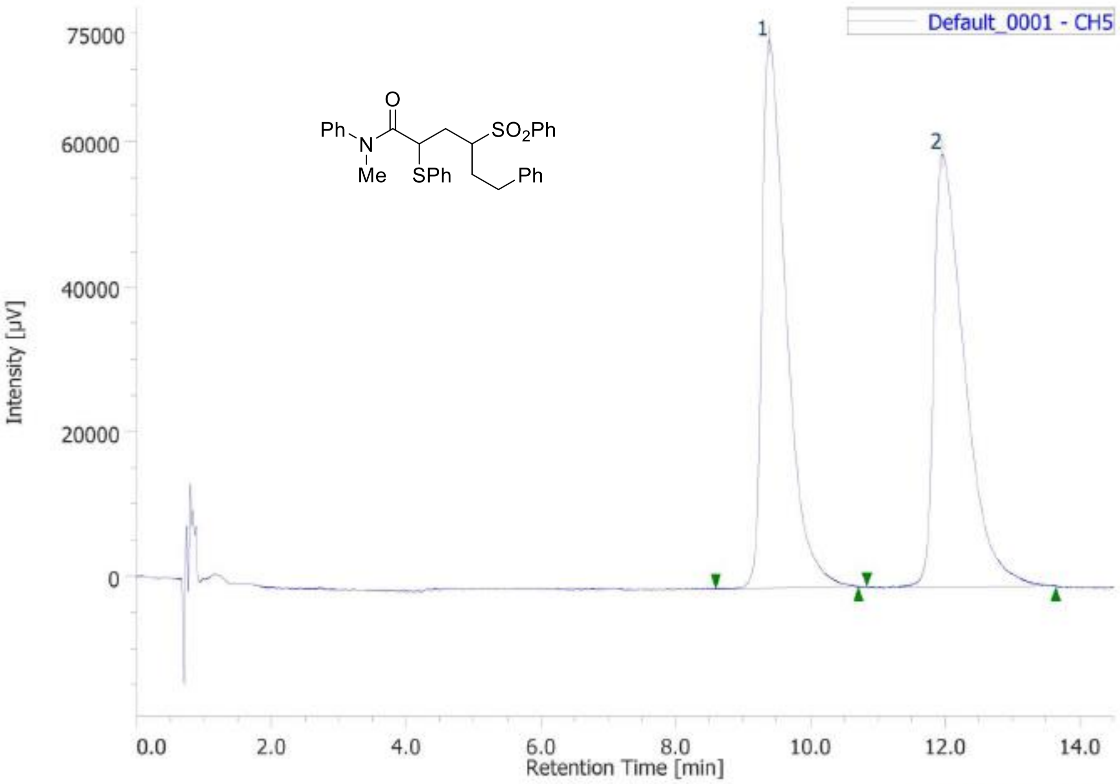

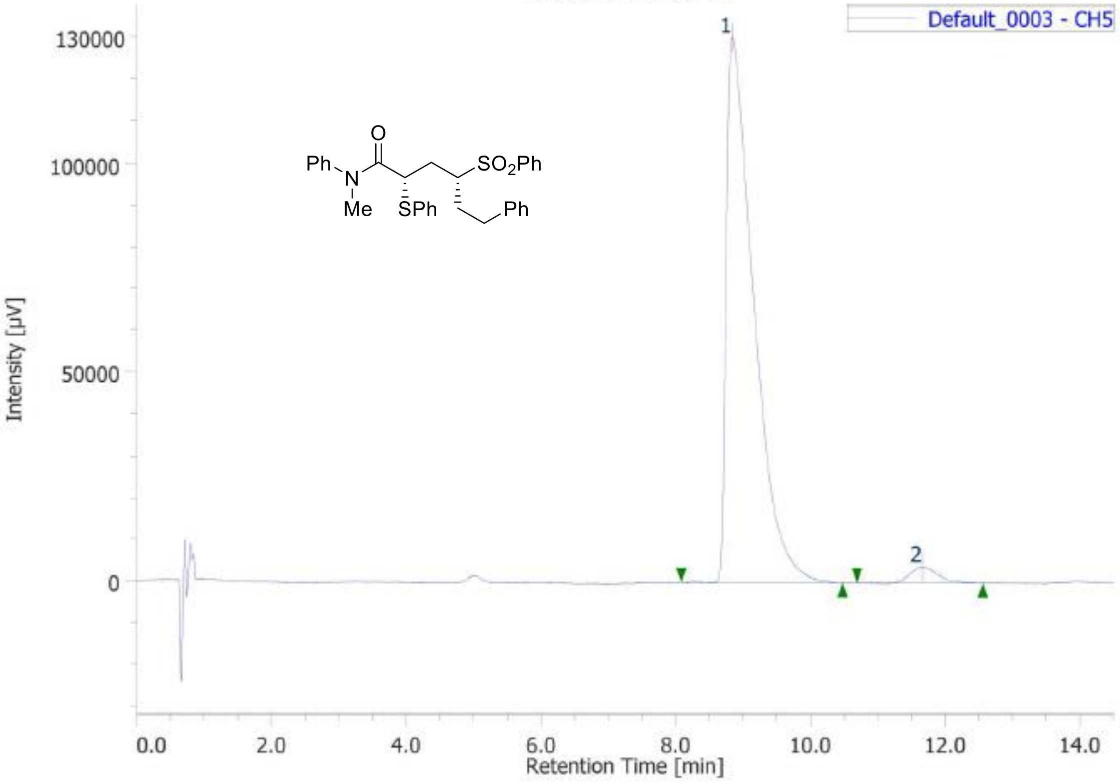

4ah

Peak	1	2
Retention Time (min)	8.8	11.7
Area (%)	97.1	2.9

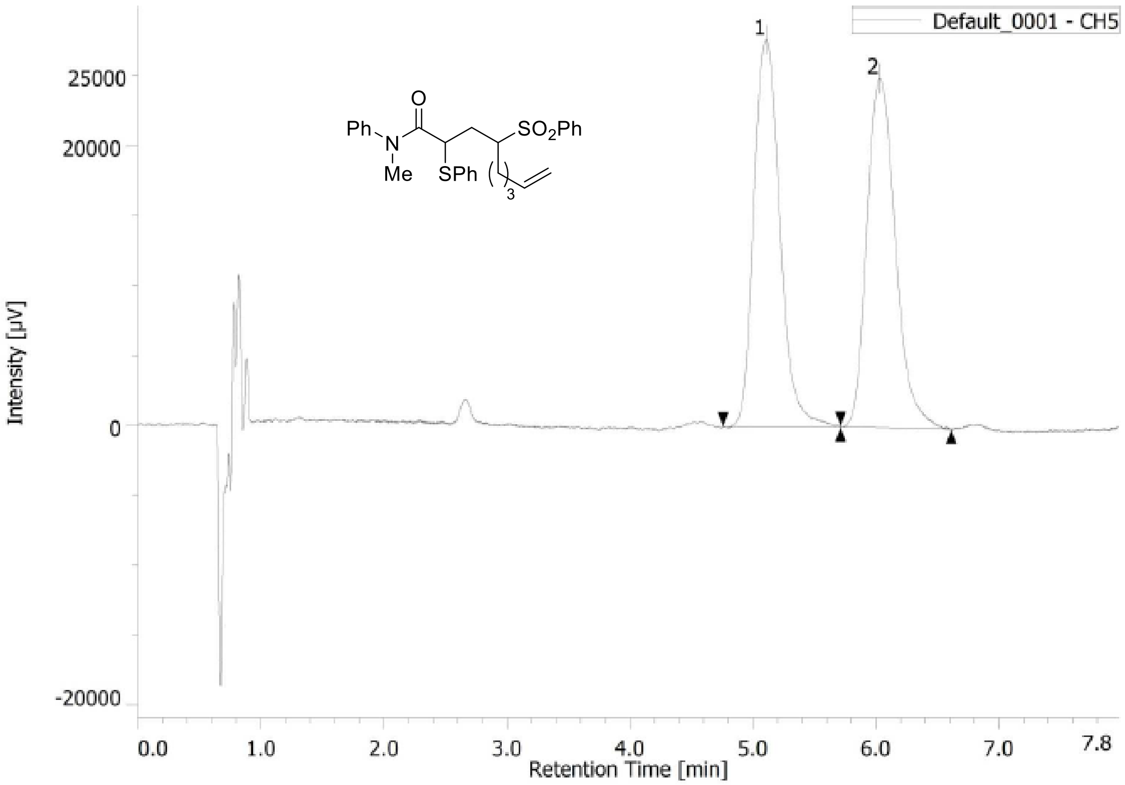

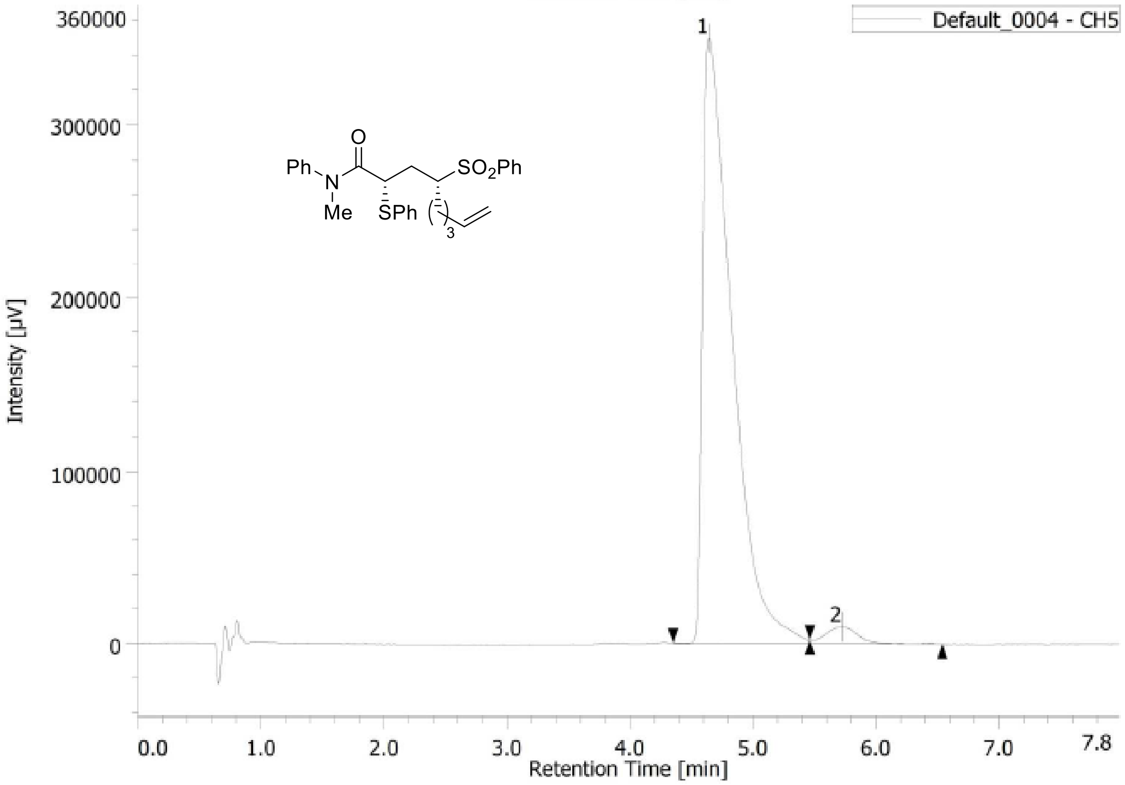

4ai

Peak	1	2
Retention Time (min)	4.6	5.7
Area (%)	97.0	3.0

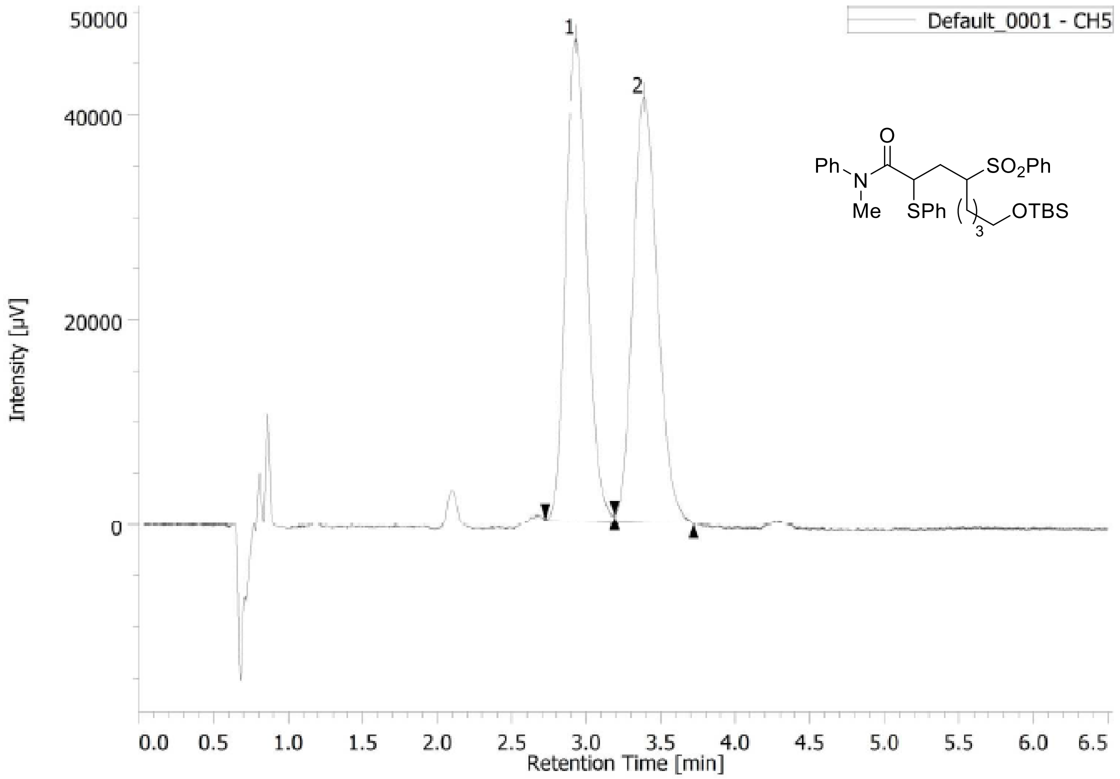

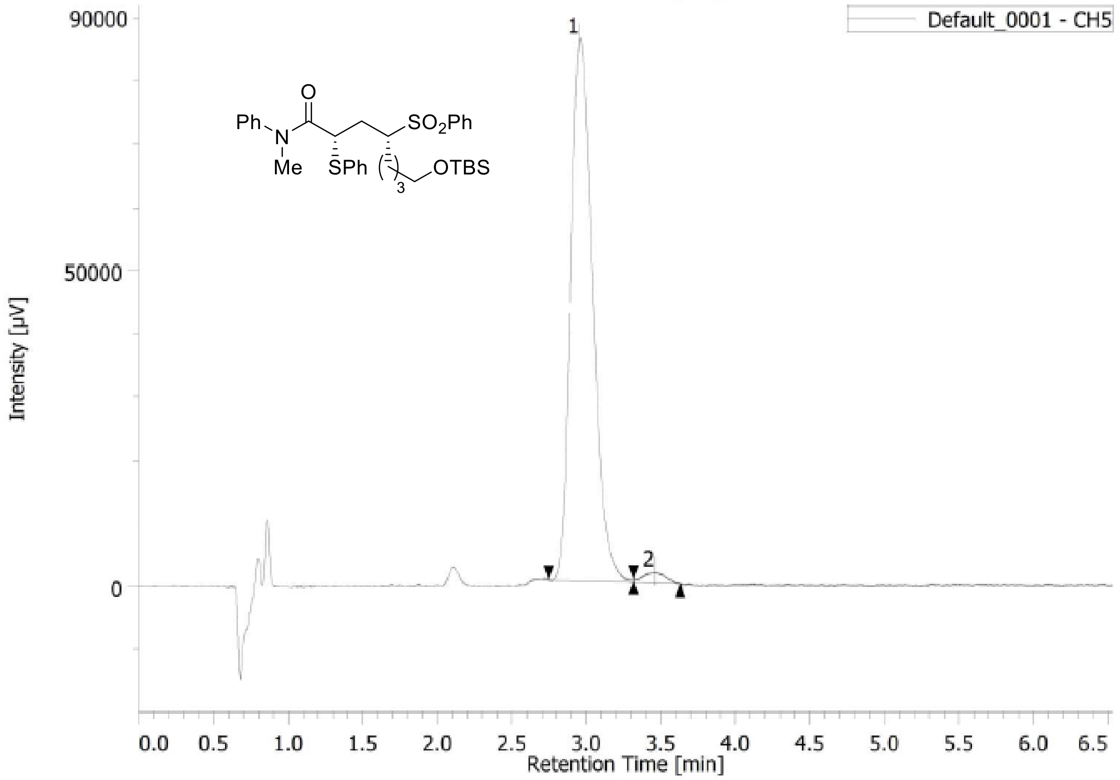

4aj

Peak	1	2
Retention Time (min)	3.0	3.5
Area (%)	98.2	1.8

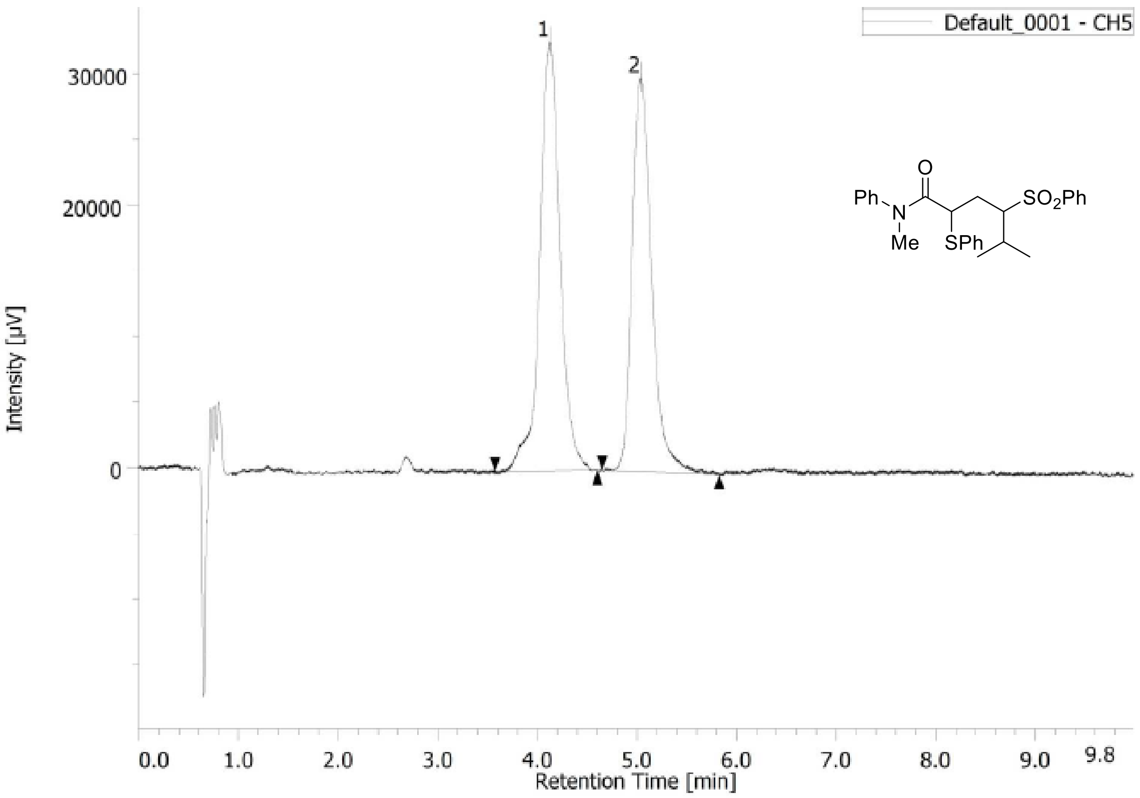

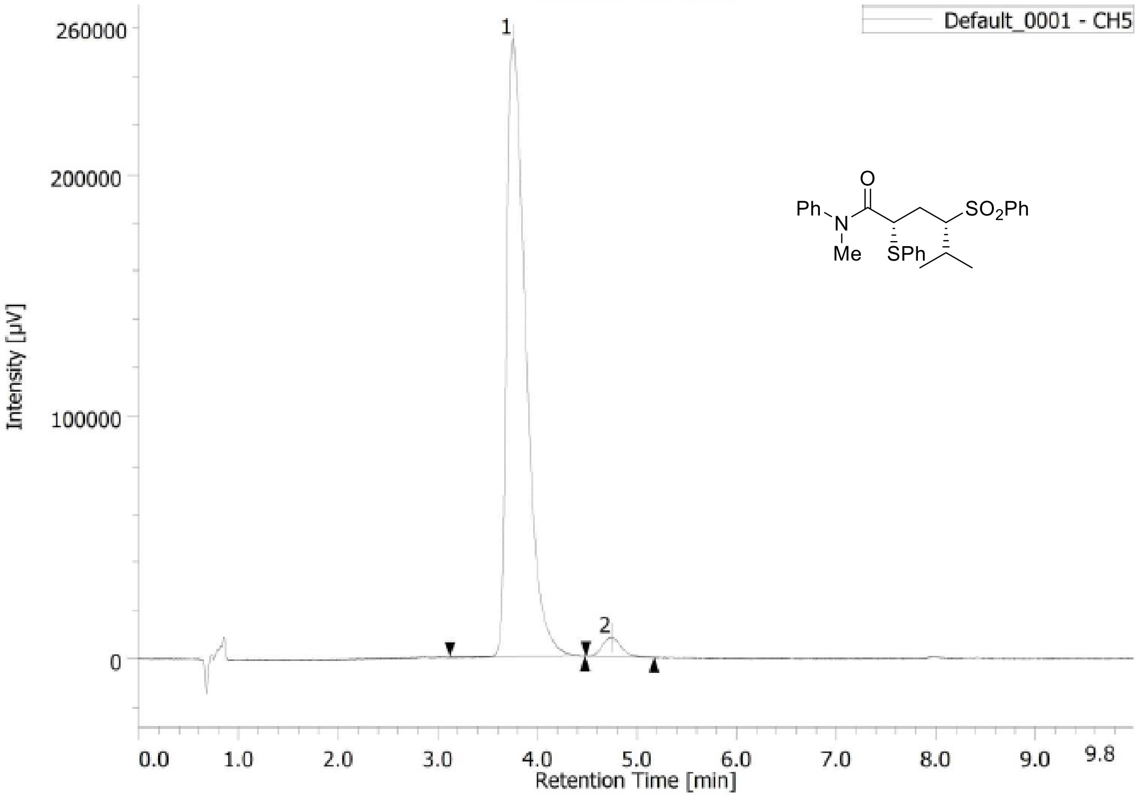

4ak

Peak	1	2
Retention Time (min)	3.8	4.7
Area (%)	97.0	3.0

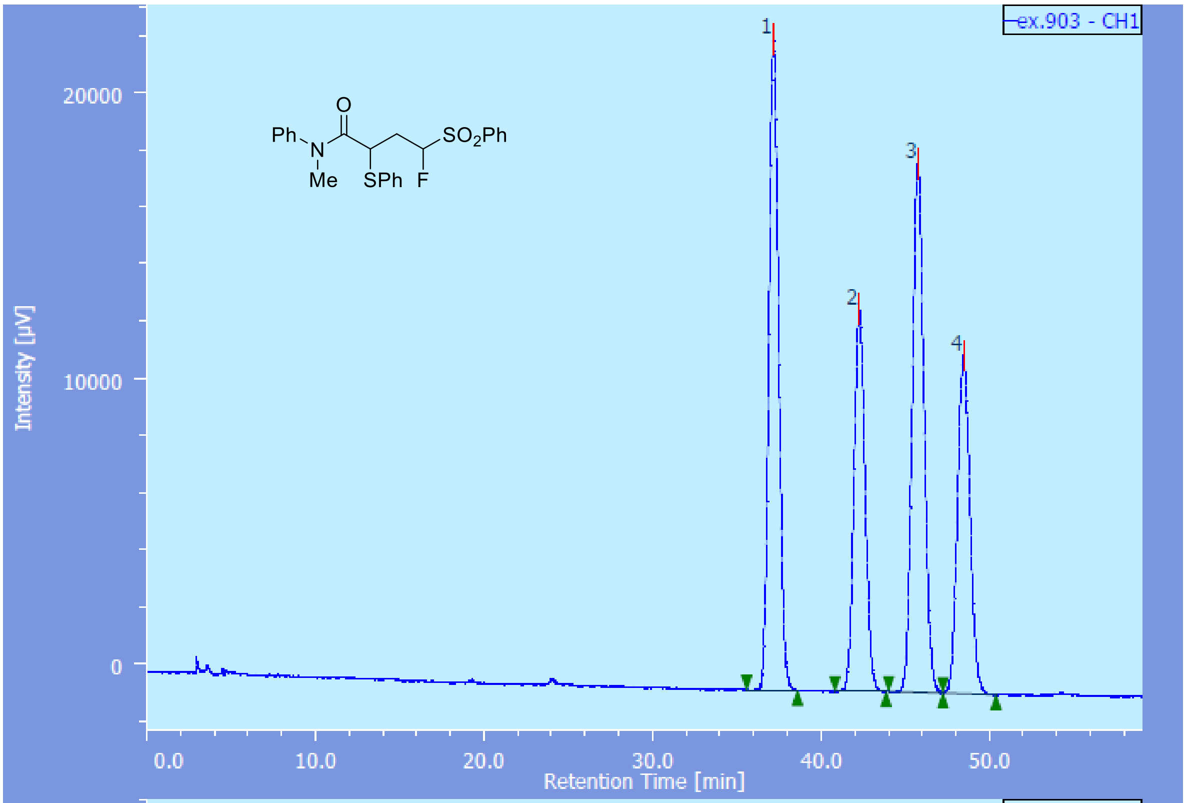

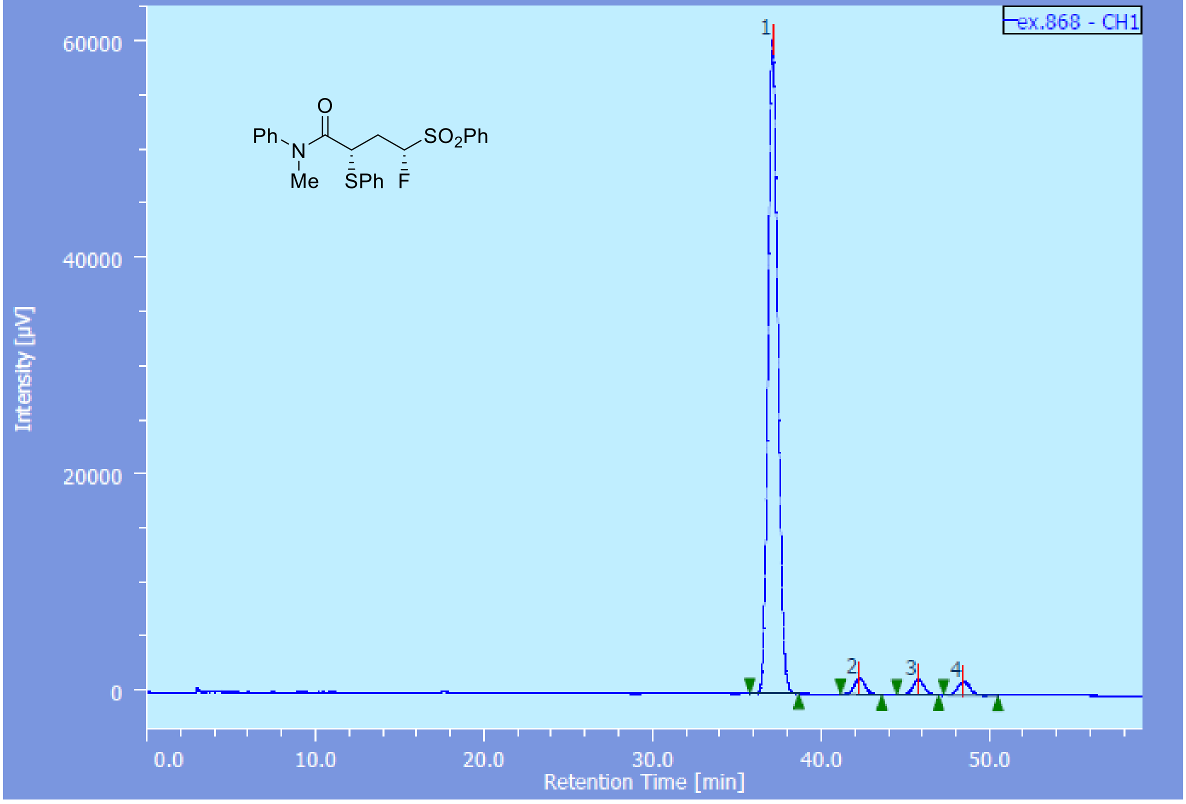

4al

Peak	1	3
Retention Time (min)	37.1	45.7
Area (%)	97.2	2.8

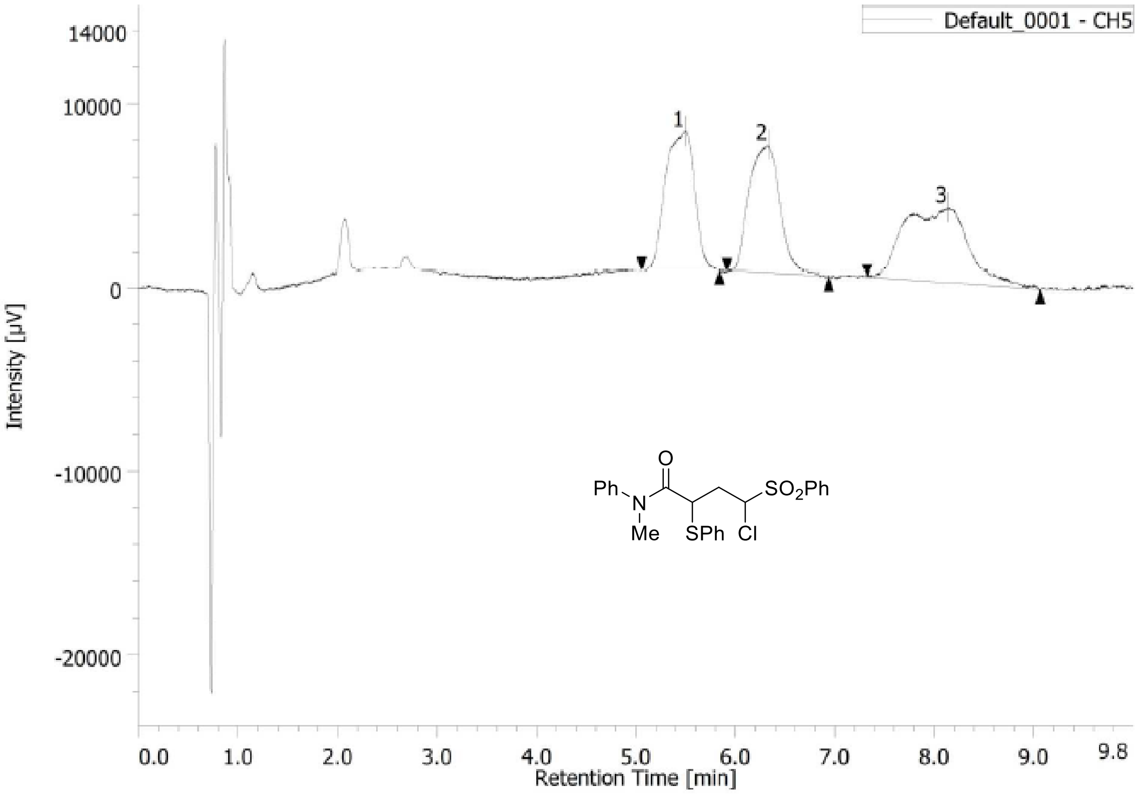

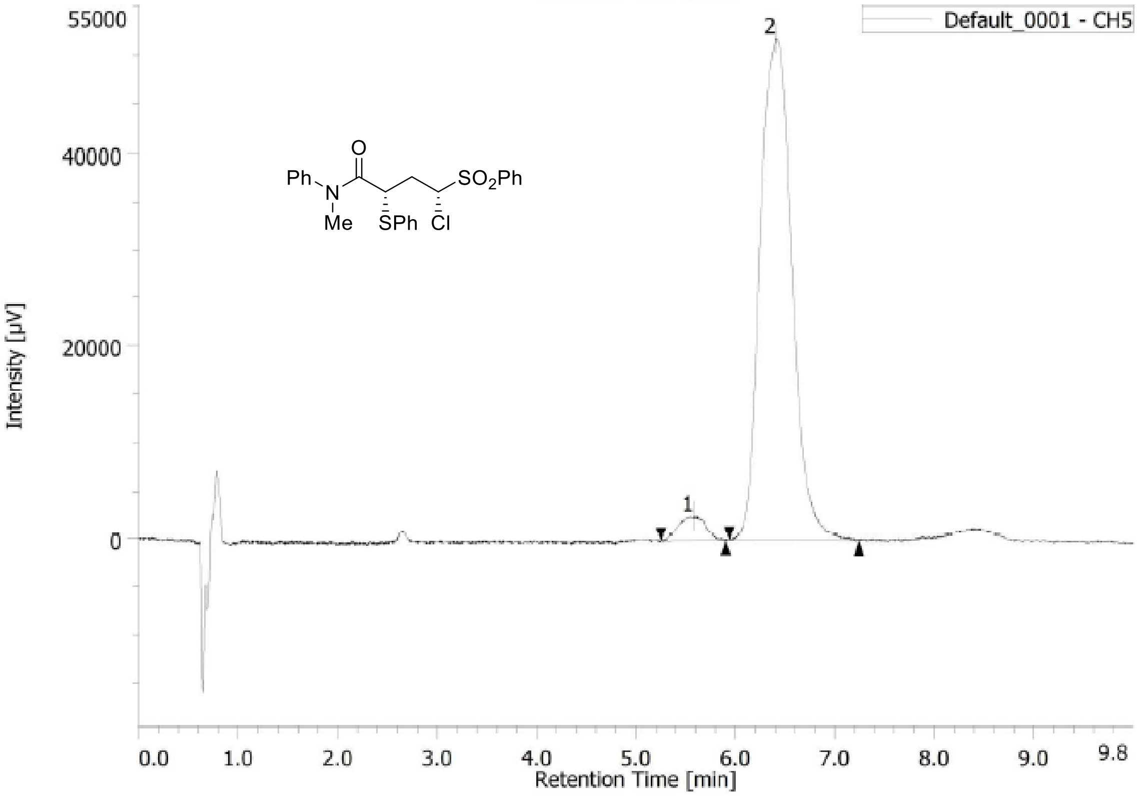

4am

Peak	1	2
Retention Time (min)	5.6	6.4
Area (%)	4.3	95.7

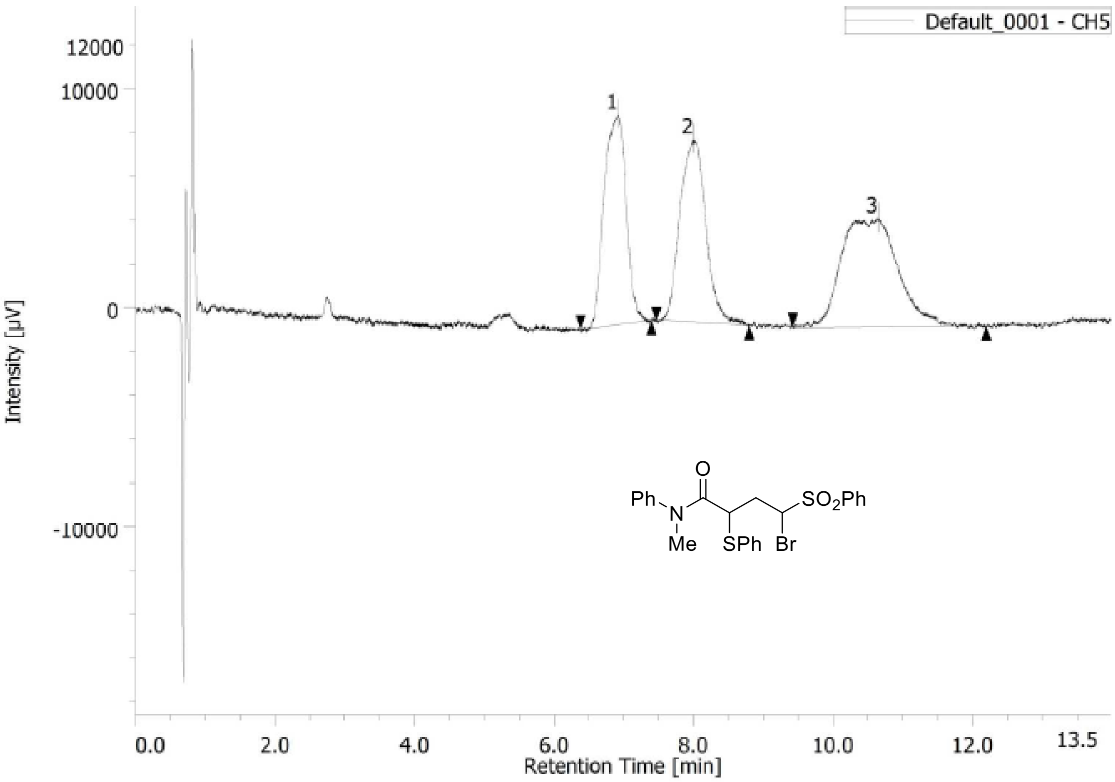

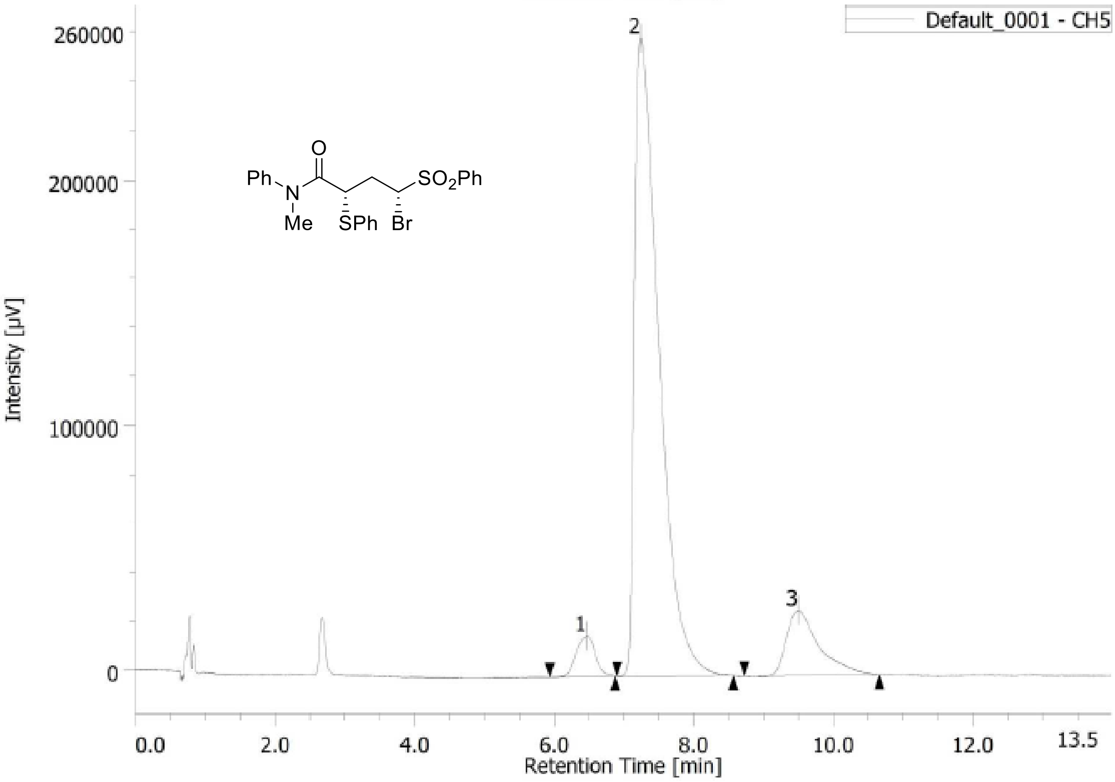

4an

Peak	1	2
Retention Time (min)	6.5	7.2
Area (%)	4.6	95.4

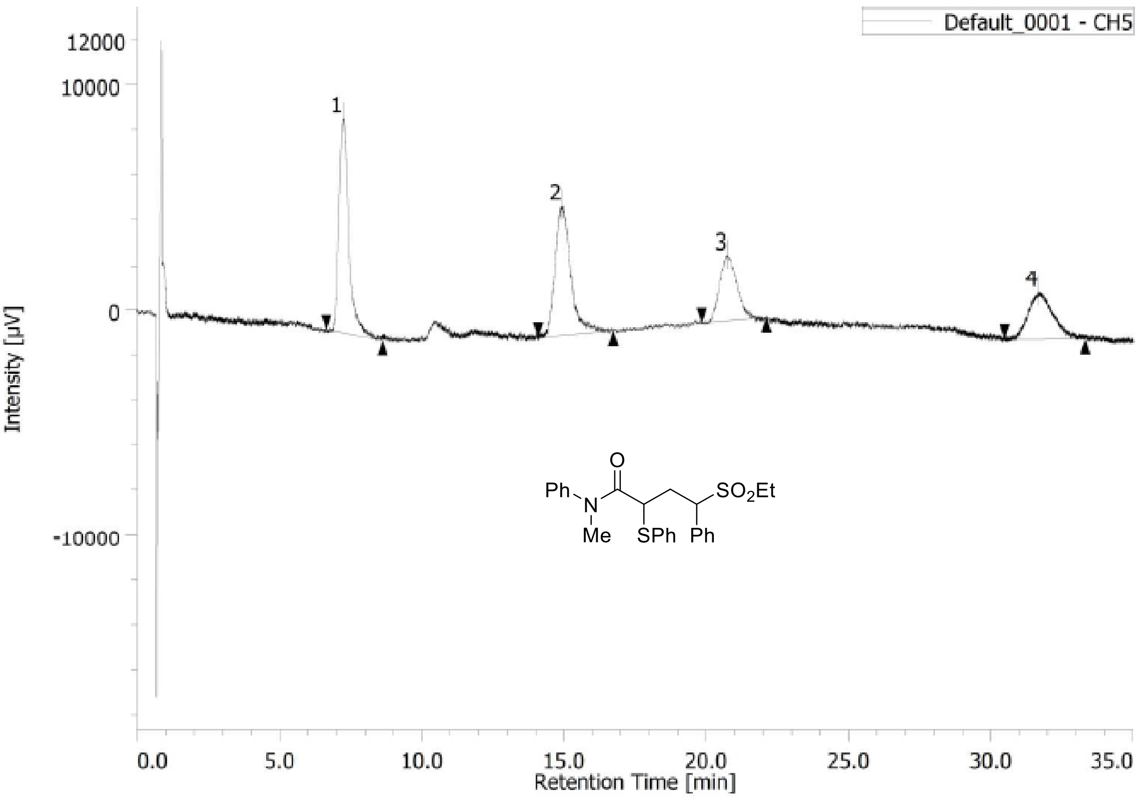

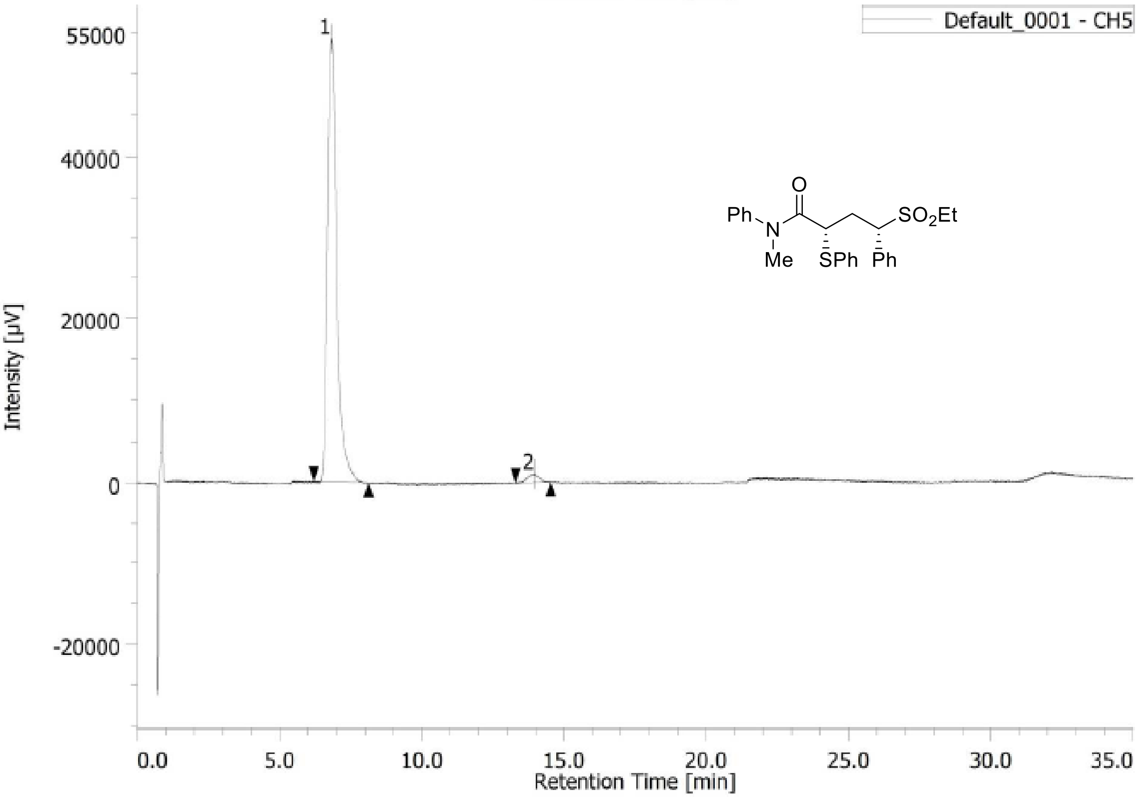

4ao

Peak	1	2
Retention Time (min)	6.8	14.0
Area (%)	97.8	2.2

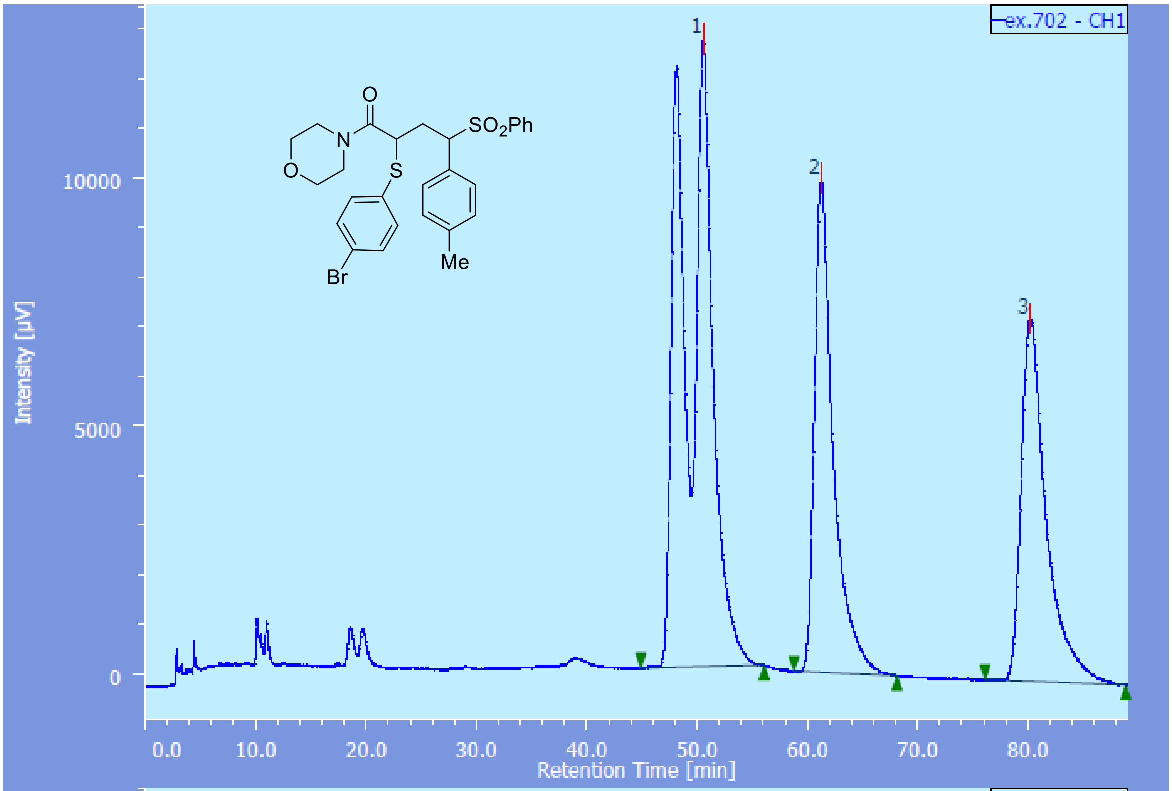

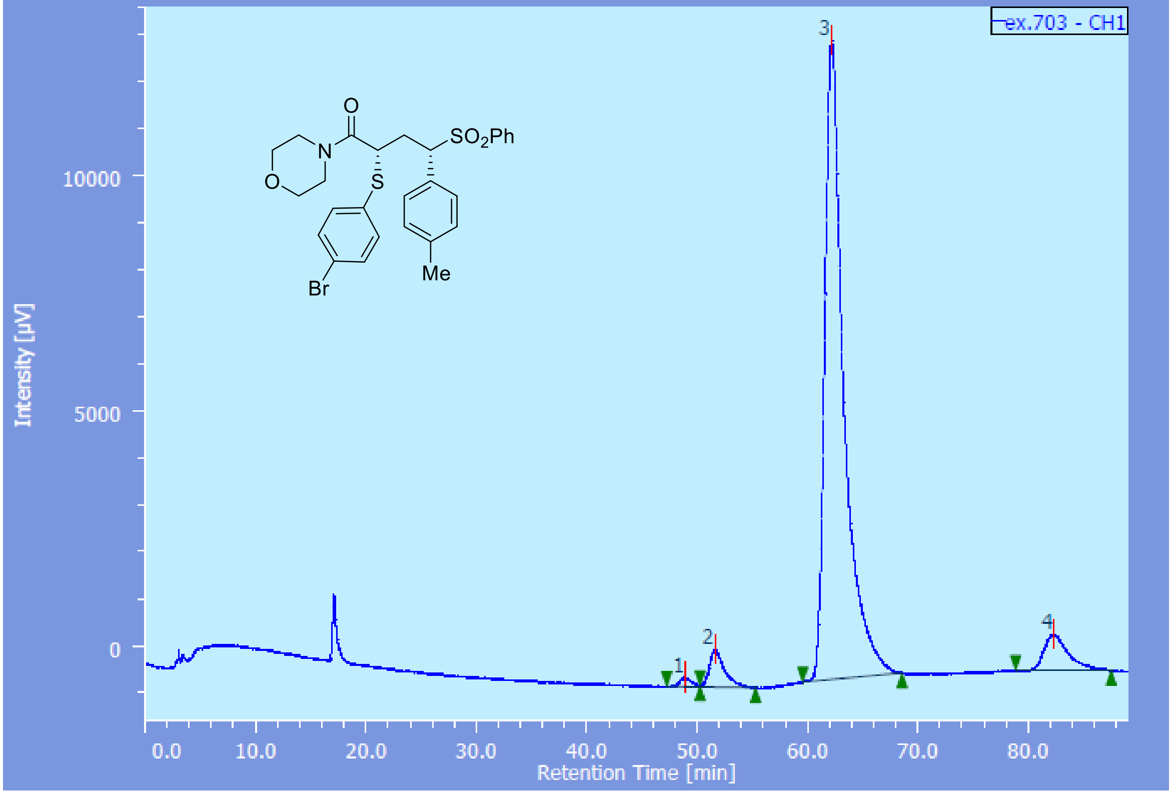

4gb

Peak	2	3
Retention Time (min)	51.6	62.6
Area (%)	95.5	4.5

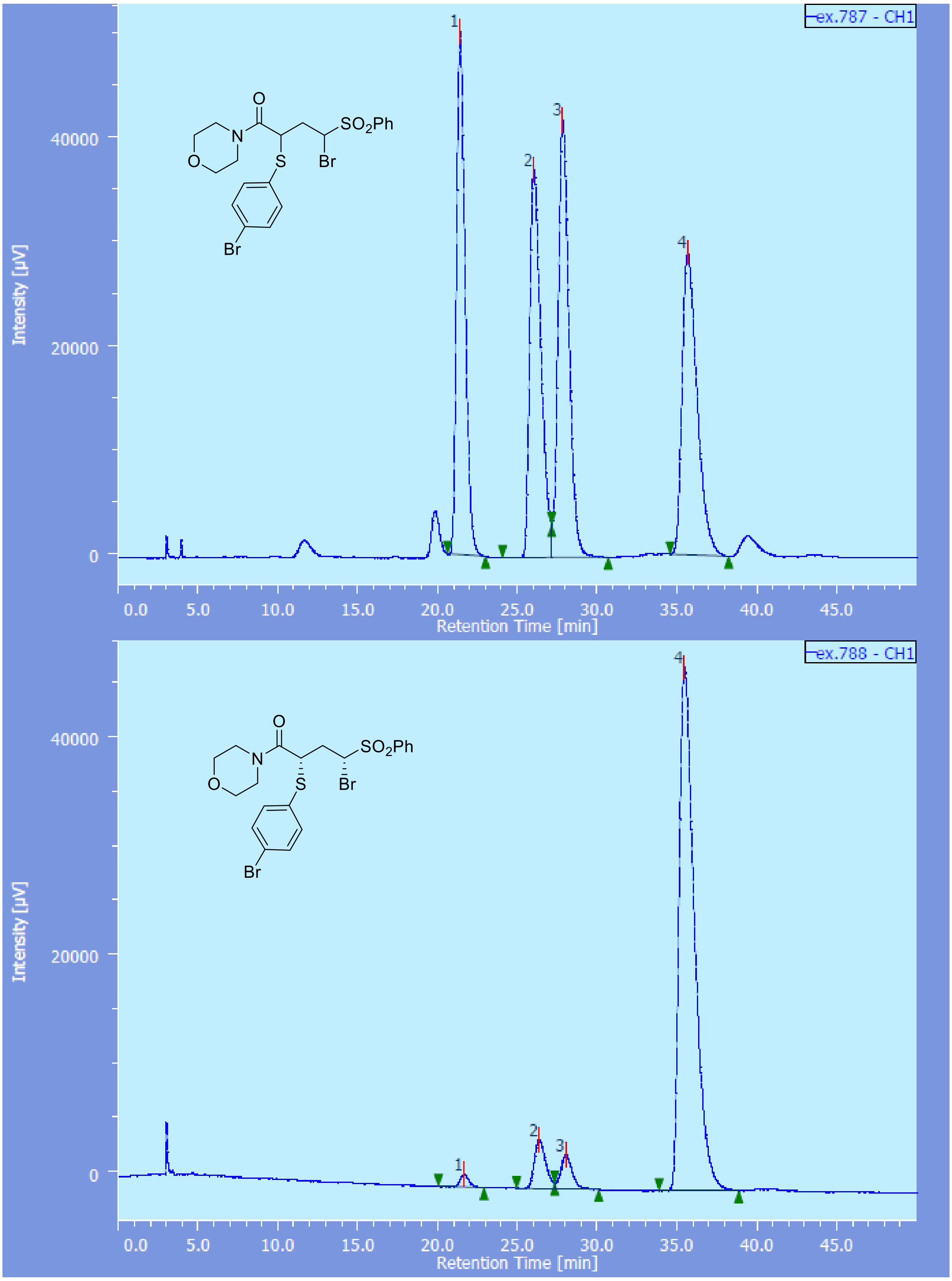

4gn

Peak	3	4
Retention Time (min)	28.0	35.5
Area (%)	95.3	4.7

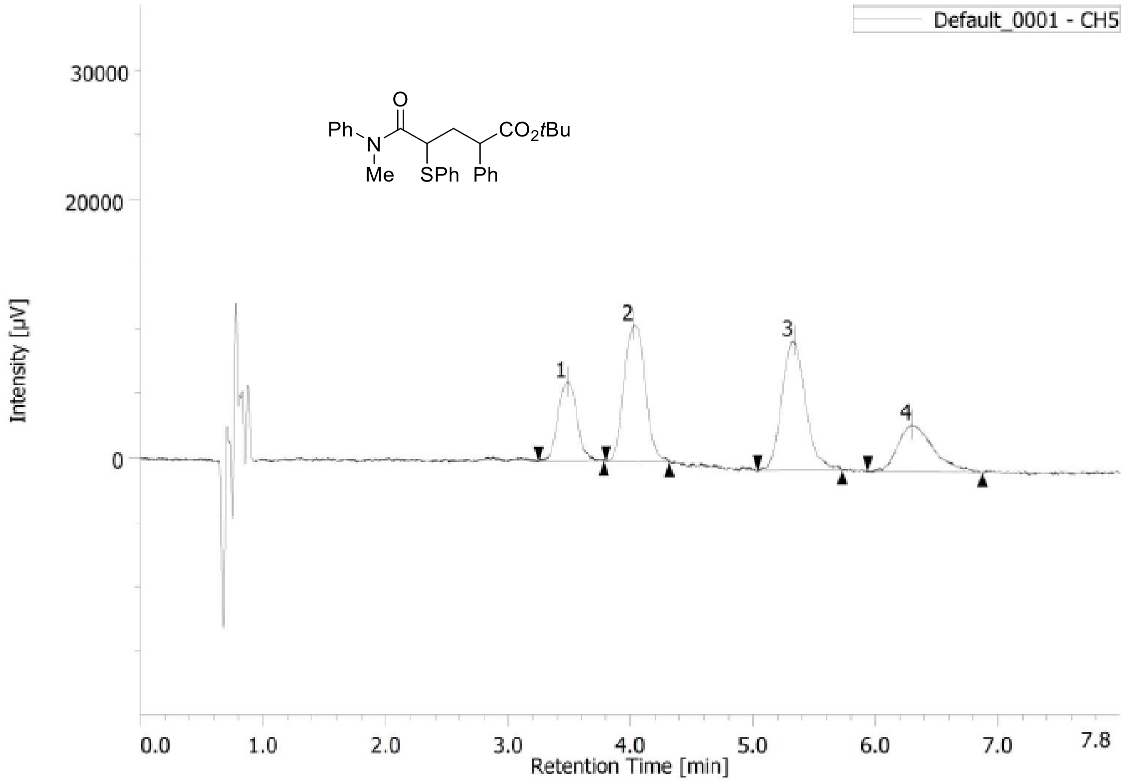

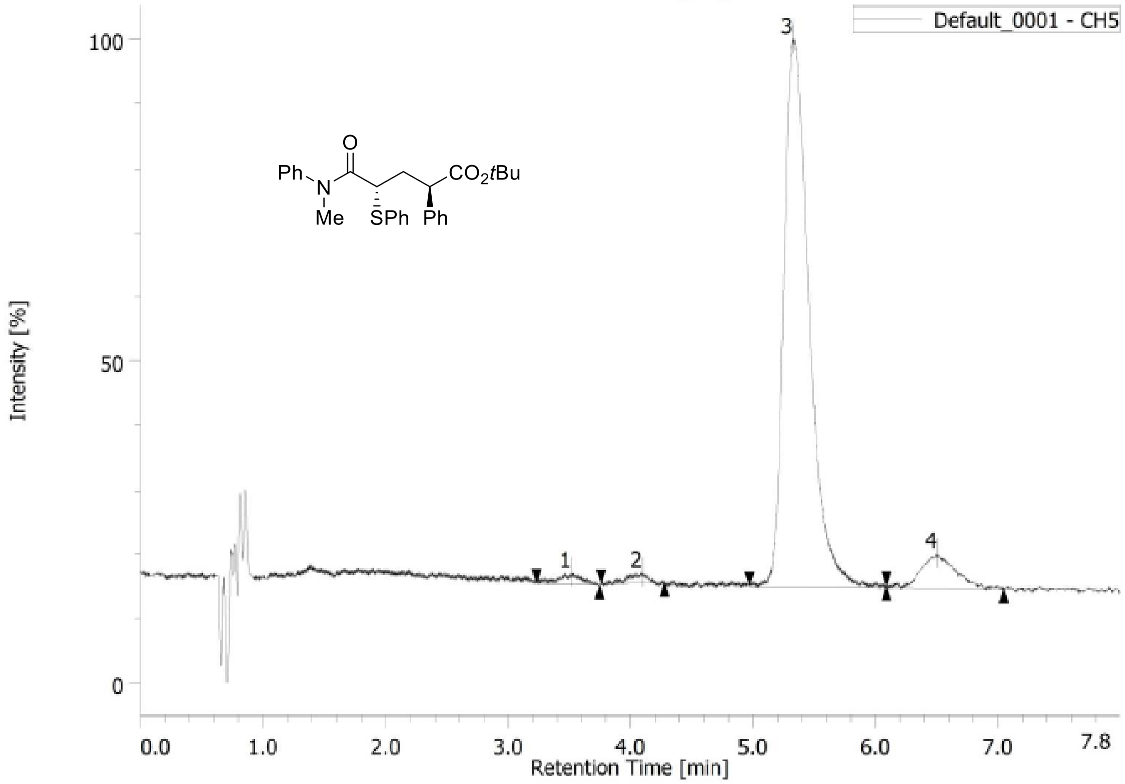

6aa

Peak	2	3
Retention Time (min)	4.1	5.3
Area (%)	98.6	1.4

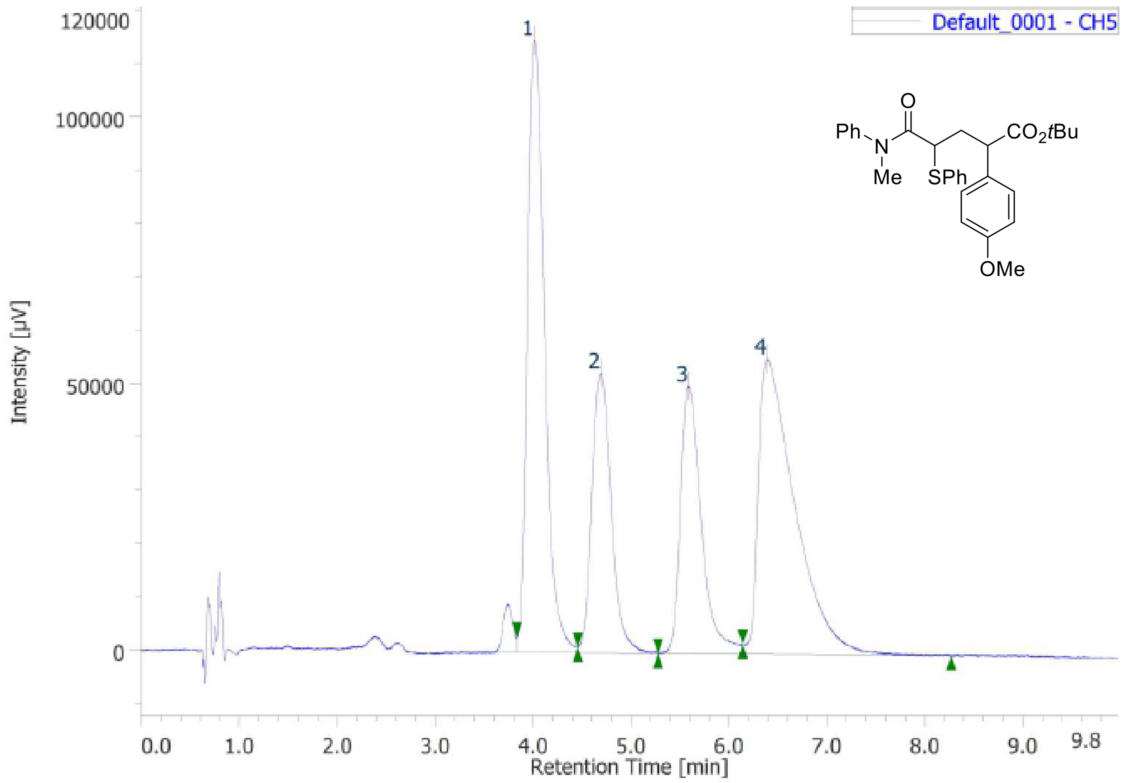

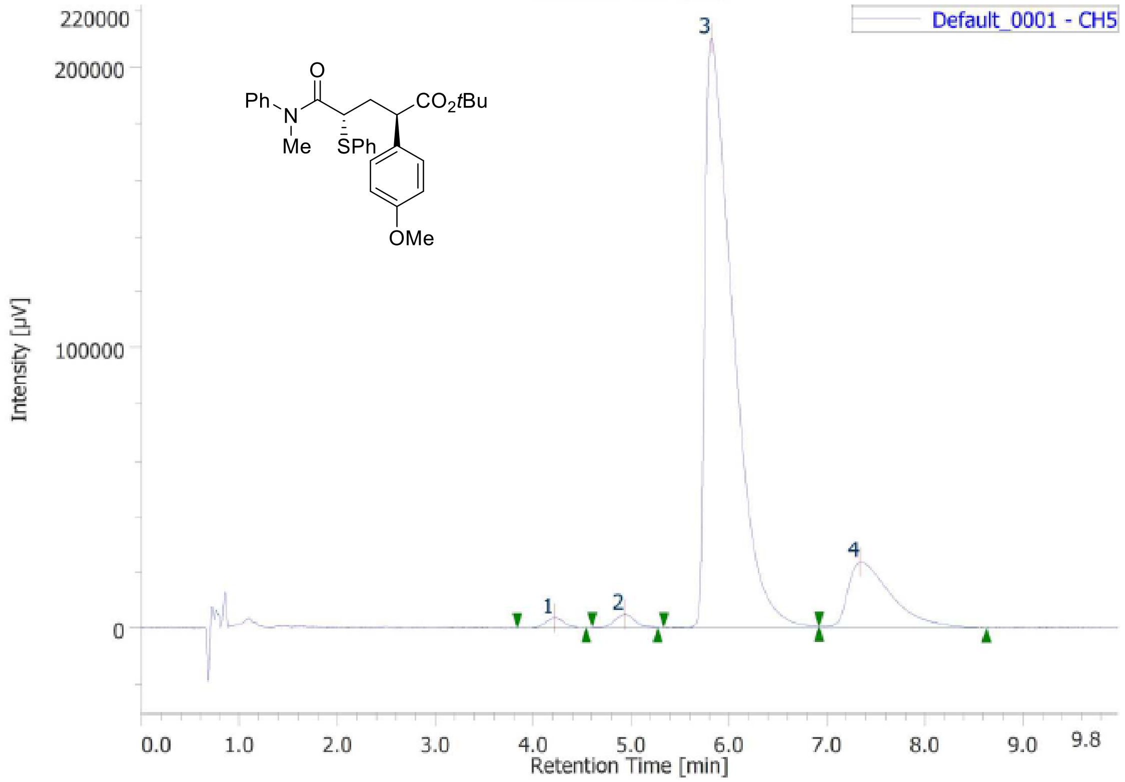

6ab

Peak	2	3
Retention Time (min)	4.9	5.8
Area (%)	98.6	1.4

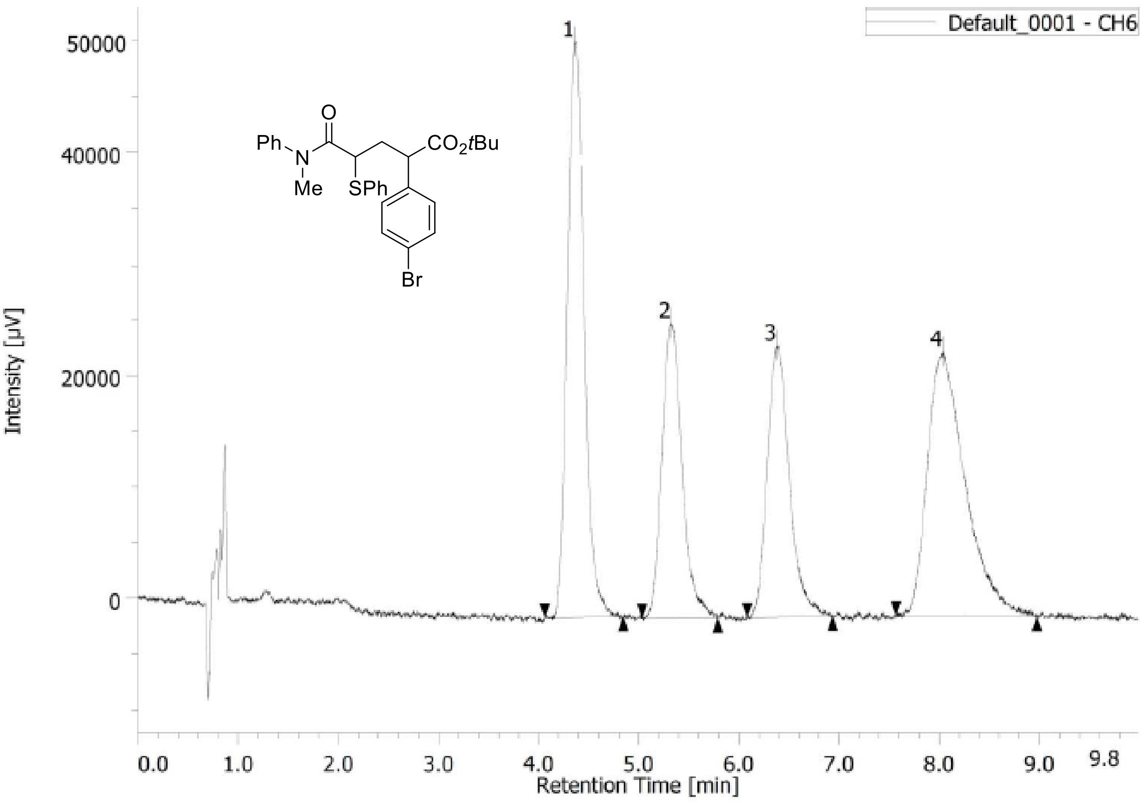

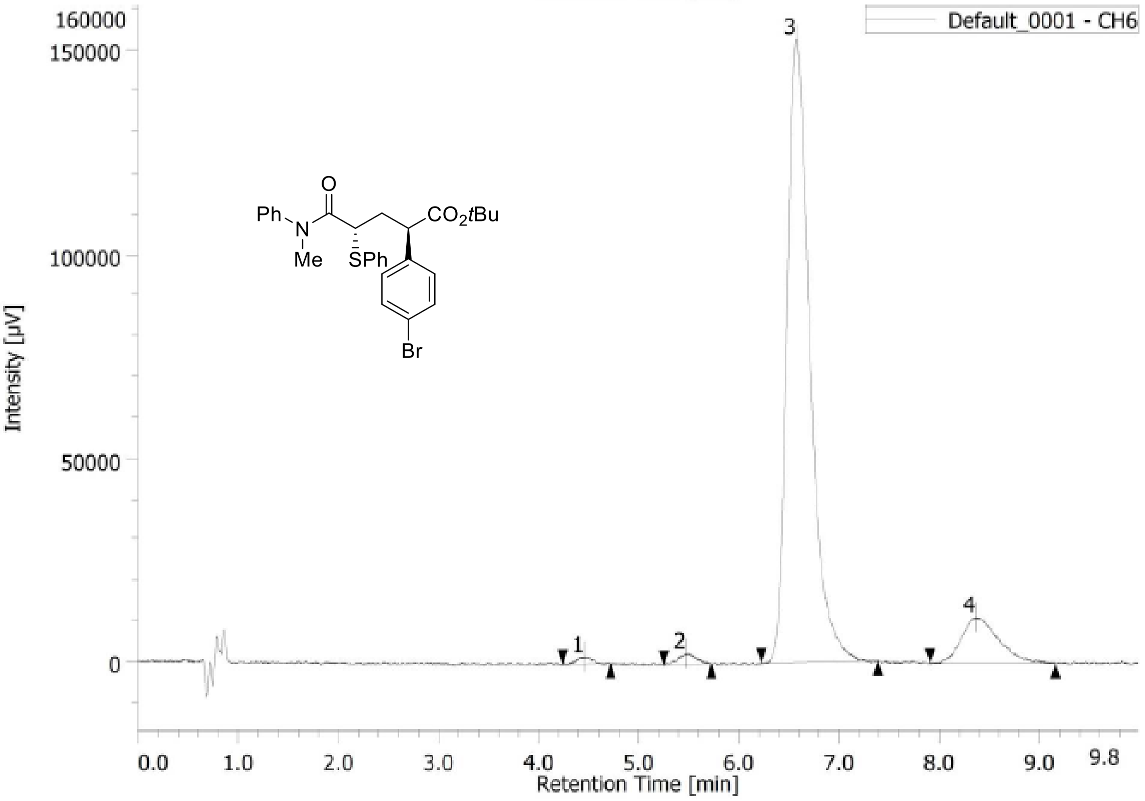

6ac

Peak	2	3
Retention Time (min)	5.5	6.6
Area (%)	98.6	1.4

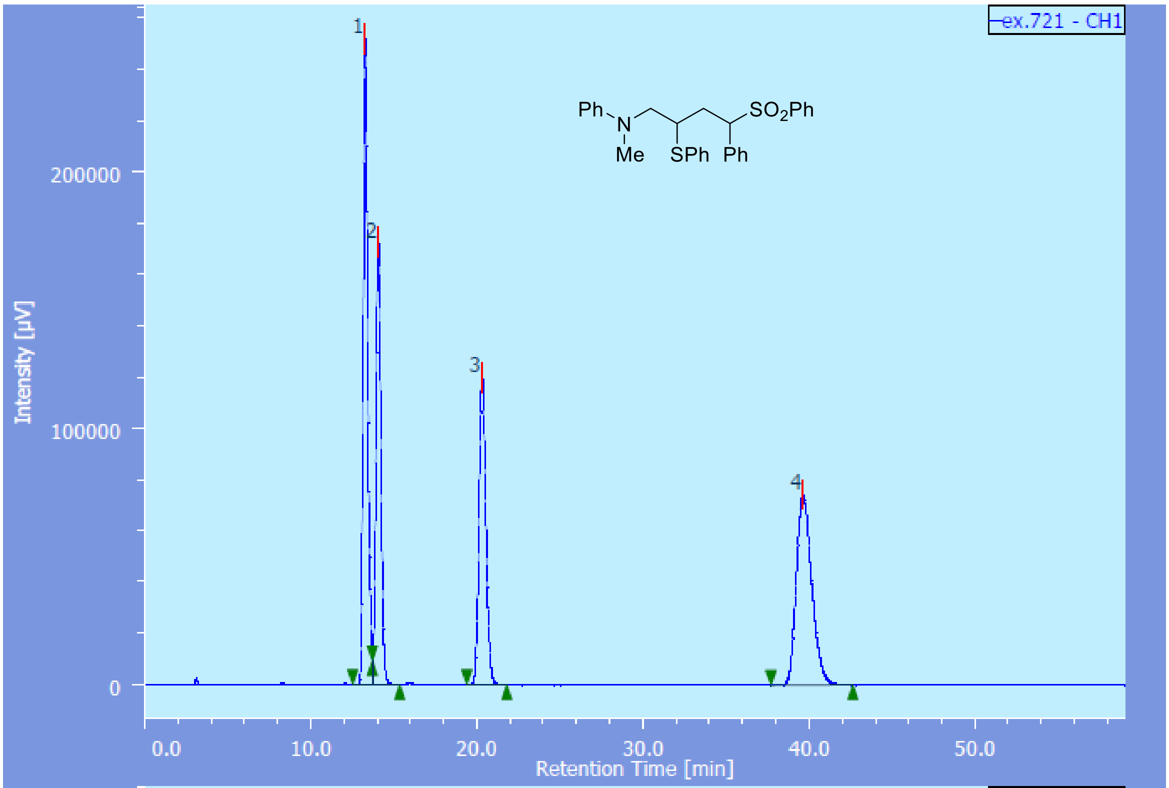

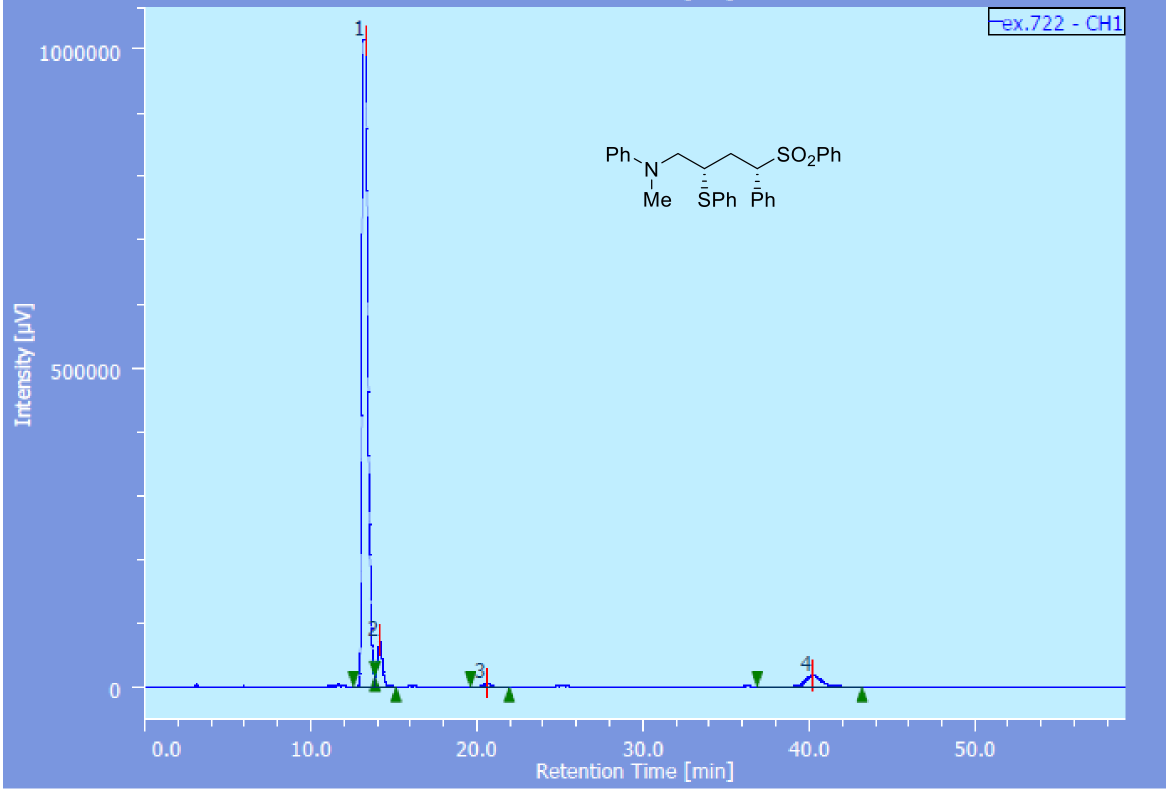

7aa

Peak	1	4
Retention Time (min)	13.3	40.2
Area (%)	95.6	4.4

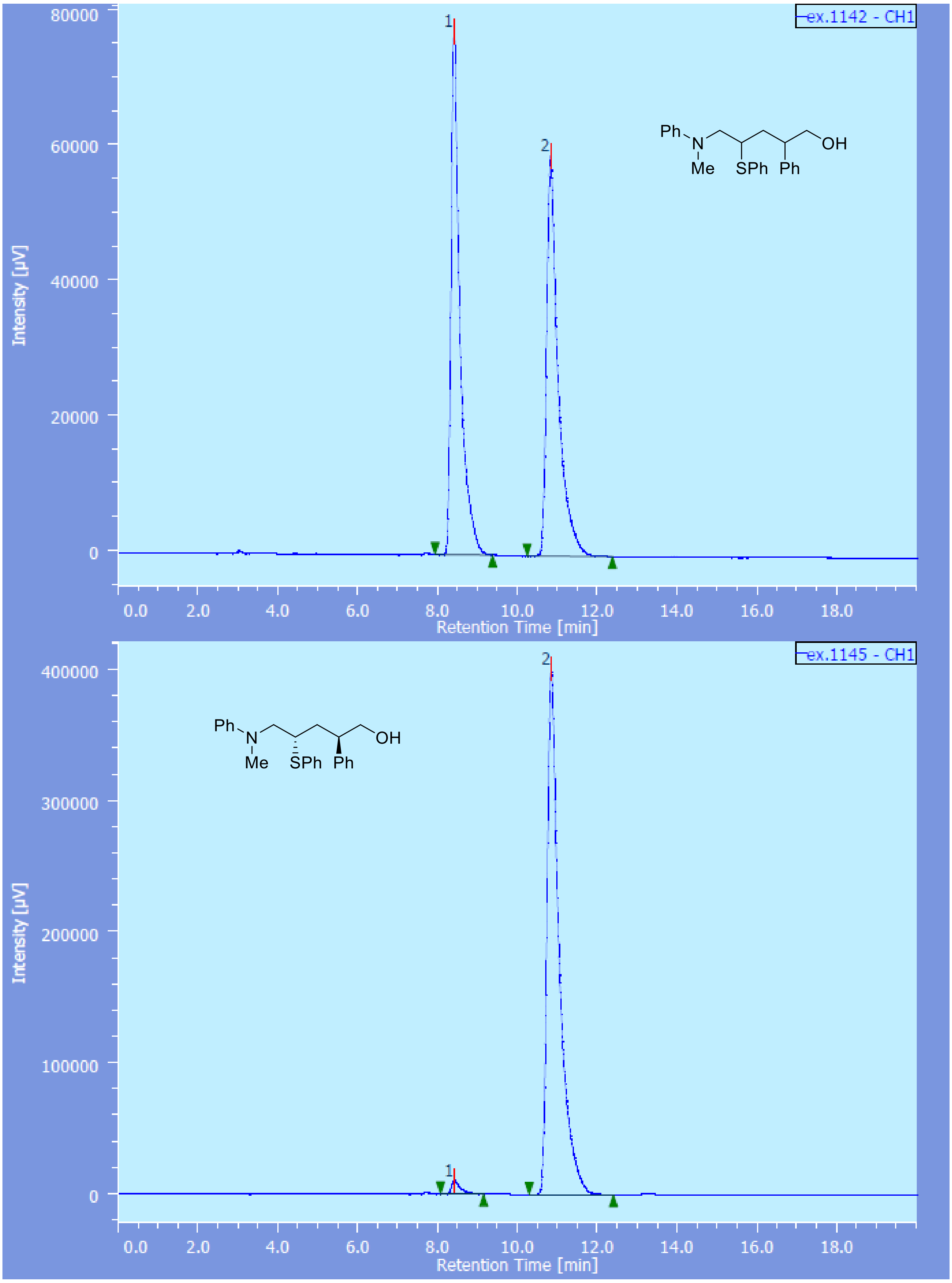

8aa

Peak	1	2
Retention Time (min)	8.4	10.9
Area (%)	2.0	98.0

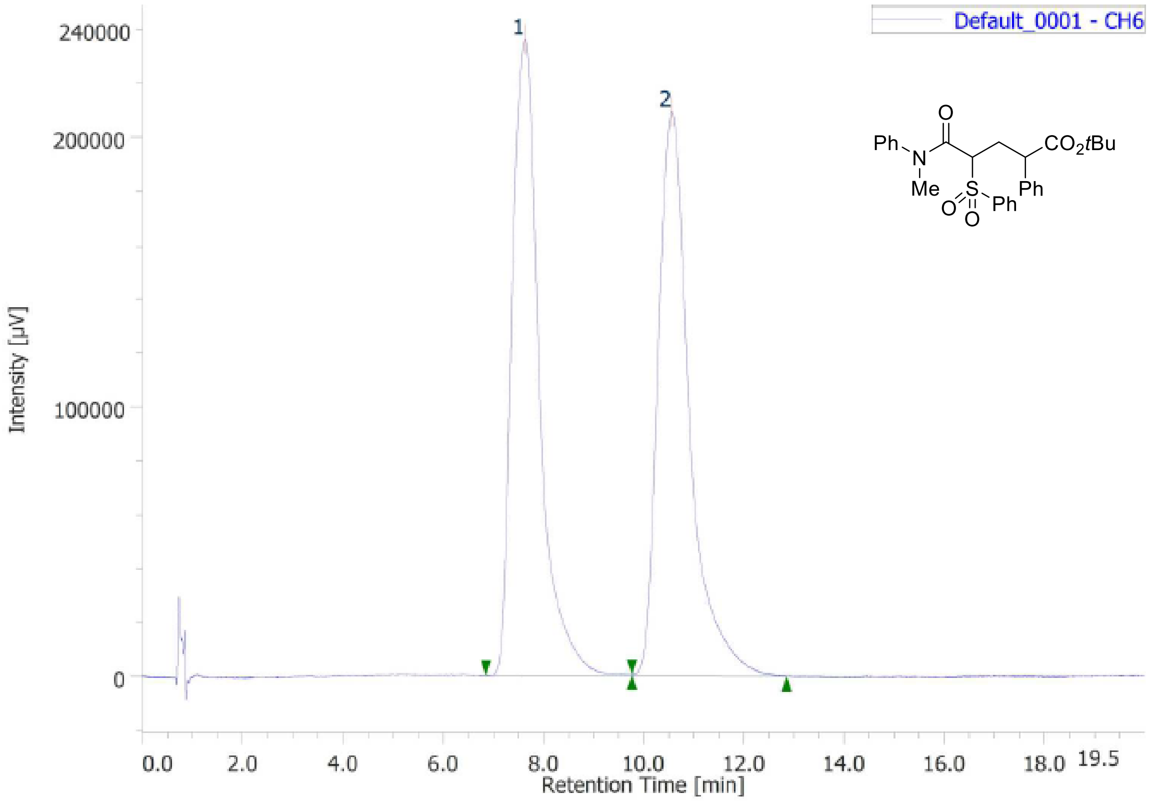

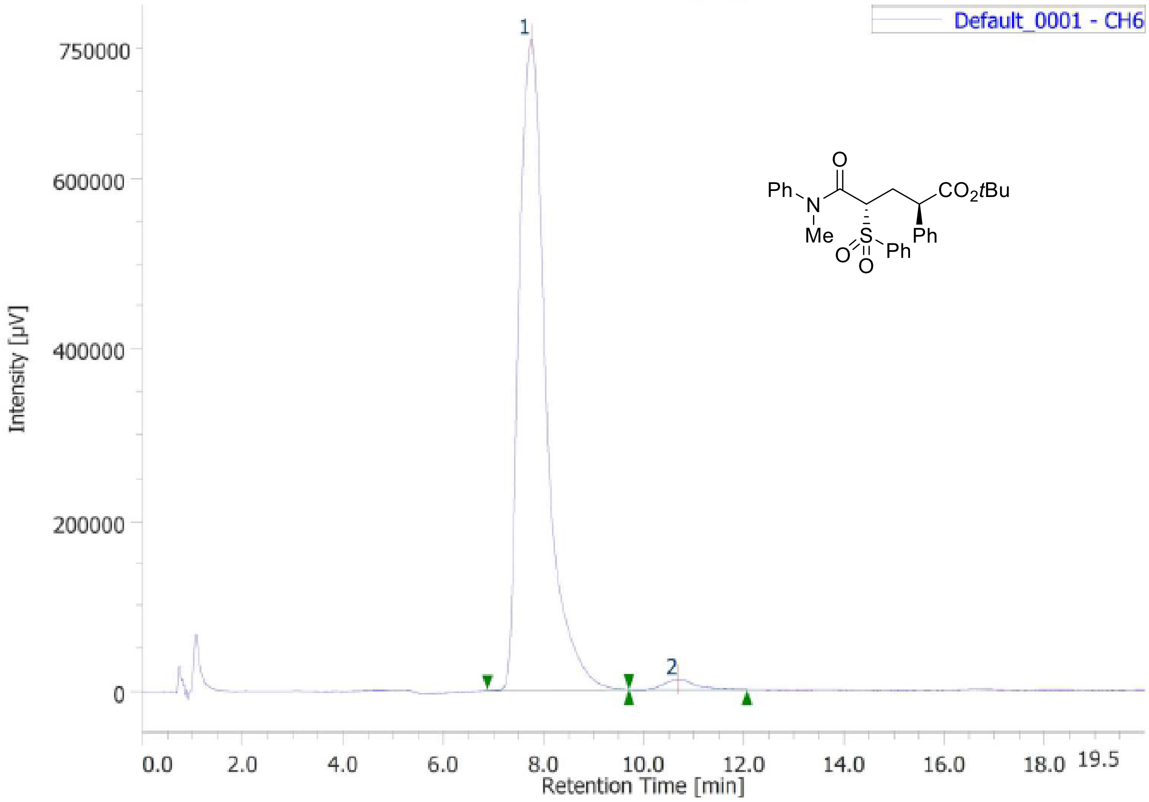

9aa

Peak	1	2
Retention Time (min)	7.8	10.7
Area (%)	98.5	1.5

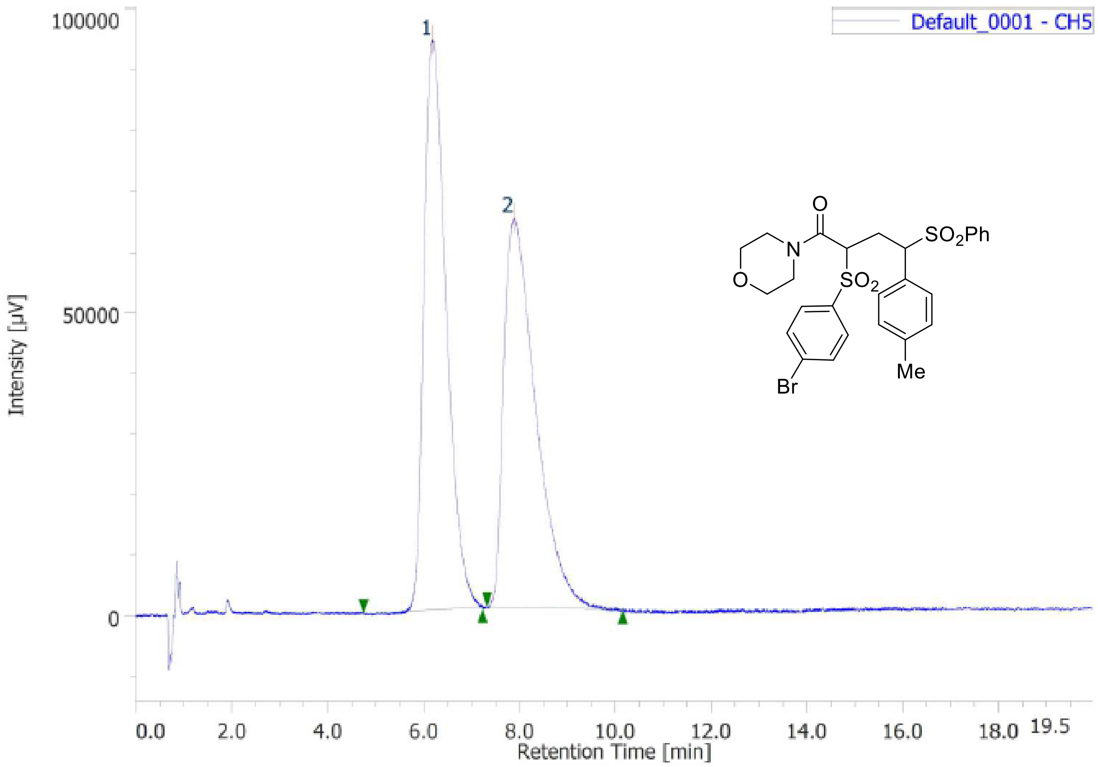

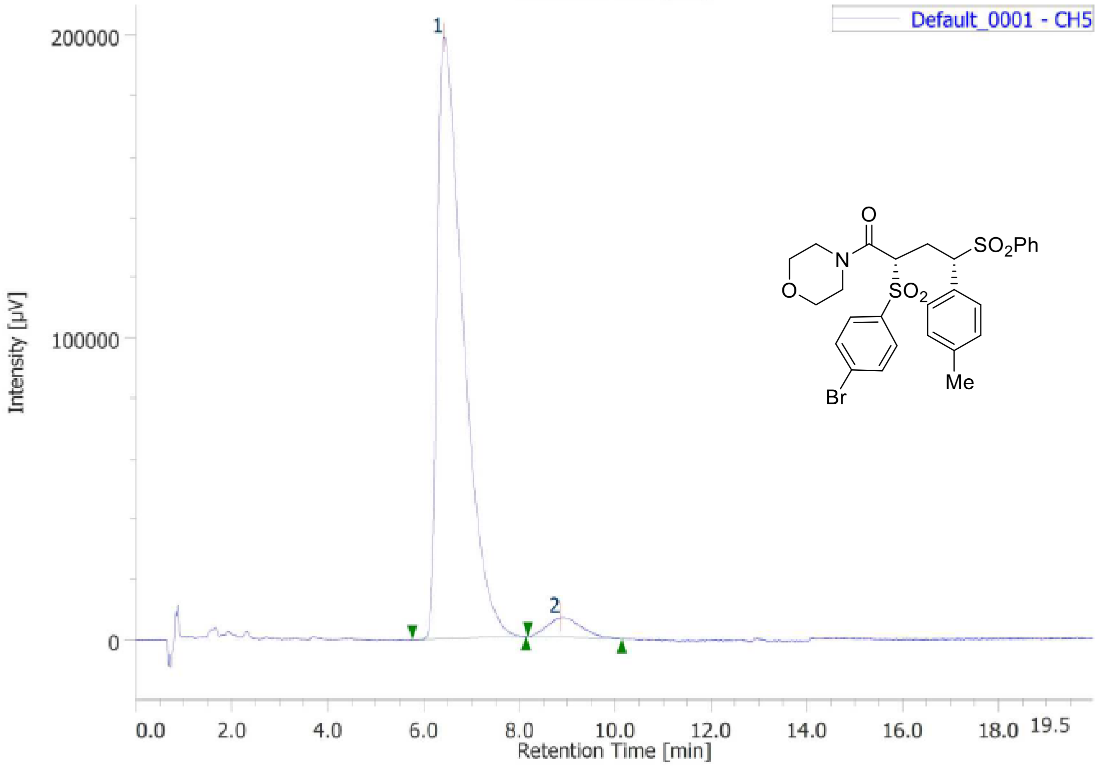

10gb

Peak	1	2
Retention Time (min)	6.4	8.9
Area (%)	95.8	4.2

6. X-ray Structure Reports

6-1. Report for 10gb

Table S1 Crystal data and structure refinement for 10gb.

Identification code	10gb
Empirical formula	$C_{27}H_{28}BrNO_6S_2$
Formula weight	606.53
Temperature/K	100.15
Crystal system	orthorhombic
Space group	$P2_12_12_1$
a/Å	9.1675(2)
b/Å	13.9765(3)
c/Å	20.7344(7)
α/°	90
β/°	90
γ/°	90
Volume/Å^3	2656.69(12)
Z	4
ρ_{calc}g/cm^3	1.516
μ/mm^{-1}	1.748
F(000)	1248.0
Crystal size/mm^3	0.16 × 0.06 × 0.03
Radiation	MoKα (λ = 0.71073)
2Θ range for data collection/°	4.858 to 54.97
Index ranges	$-11 \leq h \leq 11$, $-17 \leq k \leq 18$, $-23 \leq l \leq 25$
Reflections collected	40125
Independent reflections	6026 [R_{int} = 0.0775, R_{sigma} = 0.0572]
Data/restraints/parameters	6026/0/335
Goodness-of-fit on F^2	1.022
Final R indexes [I>=2σ (I)]	R_1 = 0.0321, wR_2 = 0.0626
Final R indexes [all data]	R_1 = 0.0410, wR_2 = 0.0668
Largest diff. peak/hole / e Å^{-3}	0.34/-0.31
Flack parameter	0.023(5)

Table S2 Fractional Atomic Coordinates (×10^4) and Equivalent Isotropic Displacement Parameters (Å^2× 10^3) for 10gb. U_{eq} is defined as 1/3 of the trace of the orthogonalised U_{IJ} tensor.

**Atom**	***x***	***y***	***z***	**U(eq)**
Br01	6500.0(4)	8770.3(2)	6248.1(2)	27.93(11)
S002	4949.6(9)	4640.1(6)	5143.2(4)	15.65(18)
S003	6545.9(10)	5889.5(6)	2751.0(4)	16.68(17)
O004	8332(3)	5521.9(15)	4805.6(12)	19.7(5)
O005	5262(3)	3907.5(17)	5609.9(11)	21.4(5)
O006	7604(3)	5787.1(17)	2243.1(12)	24.2(6)
O007	5050(3)	5650.5(16)	2619.5(12)	22.0(5)
O008	3542(3)	4650.4(17)	4836.2(12)	22.2(5)
O009	9925(3)	2627.8(17)	5859.9(12)	24.7(6)
N00A	8589(3)	3940.1(18)	5011.4(13)	16.1(6)
C00B	7838(4)	4704(2)	4800.9(16)	14.7(7)
C00C	6563(4)	3551(2)	2988.6(15)	16.6(7)
C00D	6938(4)	2603(2)	2896.3(17)	17.0(7)
C00E	6608(4)	7087(2)	3034.9(16)	16.1(7)
C00F	5246(4)	5768(2)	5501.5(16)	15.4(7)
C00G	7569(4)	4190(2)	3243.8(16)	14.7(7)
C00H	8321(4)	2259(2)	3050.2(16)	17.2(7)
C00I	9332(4)	2900(2)	3295.7(17)	18.5(7)
C00J	5554(4)	8390(2)	3622.5(19)	23.6(8)
C00K	6203(4)	5839(2)	6021.2(16)	18.6(8)
C00L	8976(3)	3862(2)	3386.7(17)	17.4(7)
C00M	6524(4)	6738(2)	6255.7(18)	20.2(7)
C00N	5929(4)	7532(2)	5962.9(18)	20.3(8)
C00O	5466(4)	7457(2)	3396.6(18)	20.2(8)
C00P	7828(4)	7638(3)	2892.4(19)	23.1(8)
C00Q	8076(4)	2950(2)	5053.3(17)	17.1(8)
C00R	7168(4)	5205(2)	3438.8(16)	14.3(7)
C00S	6310(4)	4541(2)	4520.3(16)	14.1(7)
C00T	10124(4)	4046(2)	5190.8(18)	21.2(8)
C00U	6760(4)	8942(2)	3474.7(19)	26.7(9)
C00V	5993(4)	5245(2)	3969.8(16)	16.5(7)
C00W	4934(4)	7467(2)	5461.1(17)	21.4(8)

Table S2 Fractional Atomic Coordinates (×10^4) and Equivalent Isotropic Displacement Parameters (Å^2× 10^3) for 10gb. U_{eq} is defined as 1/3 of the trace of the orthogonalised U_{IJ} tensor.

Atom	*x*	*y*	*z*	U(eq)
C00X	8396(4)	2585(2)	5727.4(17)	21.5(7)
C00Y	8678(4)	1212(2)	2979.7(17)	22.0(7)
C00Z	4590(4)	6565(2)	5230.3(18)	18.9(7)
C010	7896(4)	8577(3)	3110(2)	27.5(9)
C011	10406(4)	3605(3)	5843.1(18)	23.5(8)

Table S3 Anisotropic Displacement Parameters (Å^2×10^3) for 10gb. The Anisotropic displacement factor exponent takes the form: $-2\pi^2[h^2a^{*2}U_{11}+2hka^*b^*U_{12}+\cdots]$.

Atom	U_{11}	U_{22}	U_{33}	U_{23}	U_{13}	U_{12}
Br01	31.8(2)	18.55(16)	33.4(2)	-8.49(16)	-4.17(19)	0.88(17)
S002	14.5(4)	14.1(4)	18.4(4)	-0.2(3)	3.0(4)	-0.5(3)
S003	19.6(4)	14.9(4)	15.6(4)	1.6(3)	-1.4(4)	0.1(4)
O004	20.4(12)	14.8(11)	23.9(13)	1.2(10)	-2.6(12)	-3.8(11)
O005	27.3(14)	16.2(12)	20.5(13)	3.3(10)	4.8(11)	0.2(11)
O006	30.1(14)	24.9(13)	17.5(14)	1.6(11)	4.0(12)	1.7(12)
O007	22.5(12)	18.2(12)	25.3(14)	0.9(10)	-7.3(12)	-0.2(11)
O008	15.5(11)	22.2(12)	29.1(14)	-4.3(11)	0.9(12)	-1.3(12)
O009	27.6(13)	18.6(12)	27.9(15)	2.1(11)	-10.0(12)	2.6(12)
N00A	15.0(13)	13.8(13)	19.5(15)	1.2(10)	-2.2(12)	-1.6(12)
C00B	16.6(16)	16.1(15)	11.5(18)	1.4(14)	3.2(14)	1.3(14)
C00C	15.5(15)	20.6(17)	13.7(17)	1.5(12)	-0.6(15)	3.0(15)
C00D	16.3(17)	17.1(16)	17.4(19)	0.7(13)	-0.9(14)	-4.6(14)
C00E	19.3(17)	13.5(15)	15.4(17)	2.0(12)	-4.4(15)	0.6(15)
C00F	14.1(17)	16.0(16)	16.0(18)	-3.4(13)	2.4(14)	1.9(14)
C00G	17.0(16)	14.8(16)	12.4(18)	1.4(13)	2.2(14)	-0.9(14)
C00H	23.5(18)	17.8(16)	10.2(17)	0.4(12)	2.6(15)	0.0(16)
C00I	14.7(17)	20.7(17)	20(2)	-0.7(15)	0.4(14)	1.2(15)
C00J	23.8(19)	18.4(17)	29(2)	0.4(16)	0.0(16)	6.0(15)
C00K	20.2(19)	18.2(16)	17.5(19)	0.9(13)	1.7(14)	5.7(14)
C00L	16.5(16)	17.2(16)	18.4(19)	-1.1(14)	0.0(13)	-0.2(14)
C00M	19.2(16)	24.9(17)	16.5(18)	-3.3(15)	-0.3(18)	3.5(16)
C00N	25.2(19)	16.3(17)	19(2)	-6.6(14)	0.9(15)	0.7(15)
C00O	17.9(17)	19.2(17)	23(2)	4.1(15)	-0.3(15)	0.4(14)

Table S3 Anisotropic Displacement Parameters (Å^2×10^3) for 10gb. The Anisotropic displacement factor exponent takes the form: -2π^2[h^{2}a*^{2}U$_{11}$+2hka*b*U$_{12}$+⋯].

Atom	U_{11}	U_{22}	U_{33}	U_{23}	U_{13}	U_{12}
C00P	21.4(18)	22.3(18)	25(2)	2.6(15)	1.3(16)	1.2(16)
C00Q	17.5(17)	10.9(15)	23(2)	0.7(13)	-2.5(14)	2.0(13)
C00R	14.3(16)	12.6(15)	16.0(18)	1.7(13)	-1.9(14)	1.5(13)
C00S	15.0(17)	12.3(14)	15.1(17)	0.6(12)	-0.1(14)	2.3(14)
C00T	14.2(16)	19.1(17)	30(2)	1.7(15)	-3.6(16)	1.9(14)
C00U	28(2)	16.4(17)	36(2)	-3.2(15)	-8.0(17)	-0.4(16)
C00V	17.0(16)	14.9(16)	17.5(19)	2.8(13)	0.6(14)	2.9(14)
C00W	25.7(19)	18.6(17)	20(2)	-2.5(14)	-1.9(16)	6.8(16)
C00X	24.4(18)	16.0(16)	24(2)	3.6(14)	-2.0(17)	-0.5(16)
C00Y	24.8(18)	17.4(16)	23.9(19)	-0.1(15)	-2.8(16)	2.5(17)
C00Z	19.3(17)	19.6(17)	17.8(19)	-2.5(14)	-0.5(15)	5.2(14)
C010	26(2)	19.1(19)	38(2)	4.9(16)	-2.1(18)	-5.6(16)
C011	24.8(19)	20.8(19)	25(2)	1.4(15)	-9.2(16)	-1.7(16)

Table S4 Bond Lengths for 10gb.

Atom	Atom	Length/Å	Atom	Atom	Length/Å
Br01	C00N	1.902(3)	C00E	C00P	1.390(5)
S002	O005	1.438(2)	C00F	C00K	1.393(5)
S002	O008	1.439(3)	C00F	C00Z	1.386(5)
S002	C00F	1.763(3)	C00G	C00L	1.401(5)
S002	C00S	1.800(3)	C00G	C00R	1.520(4)
S003	O006	1.439(3)	C00H	C00I	1.386(5)
S003	O007	1.438(3)	C00H	C00Y	1.507(5)
S003	C00E	1.775(3)	C00I	C00L	1.397(5)
S003	C00R	1.810(3)	C00J	C00O	1.388(5)
O004	C00B	1.230(4)	C00J	C00U	1.383(5)
O009	C00X	1.430(4)	C00K	C00M	1.379(5)
O009	C011	1.436(4)	C00M	C00N	1.378(5)
N00A	C00B	1.343(4)	C00N	C00W	1.386(5)
N00A	C00Q	1.463(4)	C00P	C010	1.389(5)
N00A	C00T	1.463(4)	C00Q	C00X	1.516(5)
C00B	C00S	1.534(5)	C00R	C00V	1.541(5)

Table S4 Bond Lengths for 10gb.

Atom	Atom	Length/Å	Atom	Atom	Length/Å
C00C	C00D	1.382(4)	C00S	C00V	1.535(4)
C00C	C00G	1.389(5)	C00T	C011	1.509(5)
C00D	C00H	1.392(5)	C00U	C010	1.385(5)
C00E	C00O	1.387(5)	C00W	C00Z	1.385(5)

Table S5 Bond Angles for 10gb.

Atom	Atom	Atom	Angle/˚	Atom	Atom	Atom	Angle/˚
O005	S002	O008	118.91(15)	C00L	C00G	C00R	118.2(3)
O005	S002	C00F	108.79(15)	C00D	C00H	C00Y	120.7(3)
O005	S002	C00S	106.86(14)	C00I	C00H	C00D	118.0(3)
O008	S002	C00F	108.40(15)	C00I	C00H	C00Y	121.2(3)
O008	S002	C00S	107.74(15)	C00H	C00I	C00L	121.1(3)
C00F	S002	C00S	105.32(16)	C00U	C00J	C00O	119.7(3)
O006	S003	C00E	108.35(16)	C00M	C00K	C00F	118.2(3)
O006	S003	C00R	108.17(15)	C00I	C00L	C00G	120.1(3)
O007	S003	O006	118.77(16)	C00N	C00M	C00K	119.6(3)
O007	S003	C00E	108.21(16)	C00M	C00N	Br01	119.1(3)
O007	S003	C00R	109.10(15)	C00M	C00N	C00W	122.6(3)
C00E	S003	C00R	103.13(15)	C00W	C00N	Br01	118.3(3)
C00X	O009	C011	109.6(3)	C00E	C00O	C00J	119.3(3)
C00B	N00A	C00Q	127.3(3)	C010	C00P	C00E	119.4(3)
C00B	N00A	C00T	119.7(3)	N00A	C00Q	C00X	108.1(3)
C00T	N00A	C00Q	112.9(3)	C00G	C00R	S003	111.1(2)
O004	C00B	N00A	123.2(3)	C00G	C00R	C00V	113.2(3)
O004	C00B	C00S	118.5(3)	C00V	C00R	S003	108.9(2)
N00A	C00B	C00S	118.3(3)	C00B	C00S	S002	110.4(2)
C00D	C00C	C00G	120.3(3)	C00B	C00S	C00V	111.1(3)
C00C	C00D	C00H	121.7(3)	C00V	C00S	S002	110.7(2)
C00O	C00E	S003	120.4(3)	N00A	C00T	C011	110.6(3)
C00O	C00E	C00P	121.0(3)	C00J	C00U	C010	121.1(3)
C00P	C00E	S003	118.5(3)	C00S	C00V	C00R	112.1(3)
C00K	C00F	S002	119.2(3)	C00Z	C00W	C00N	118.0(3)
C00Z	C00F	S002	118.8(3)	O009	C00X	C00Q	110.6(3)

Table S5 Bond Angles for 10gb.

Atom	Atom	Atom	Angle/˚	Atom	Atom	Atom	Angle/˚
C00Z	C00F	C00K	122.0(3)	C00W	C00Z	C00F	119.6(3)
C00C	C00G	C00L	118.8(3)	C00U	C010	C00P	119.5(3)
C00C	C00G	C00R	122.7(3)	O009	C011	C00T	111.0(3)

Table S6 Torsion Angles for 10gb.

A	B	C	D	Angle/˚	A	B	C	D	Angle/˚
Br01	C00N	C00W	C00Z	176.2(3)	C00D	C00C	C00G	C00R	-171.7(3)
S002	C00F	C00K	C00M	174.1(3)	C00D	C00H	C00I	C00L	0.0(5)
S002	C00F	C00Z	C00W	-173.0(3)	C00E	S003	C00R	C00G	-165.2(2)
S002	C00S	C00V	C00R	178.0(2)	C00E	S003	C00R	C00V	69.5(3)
S003	C00E	C00O	C00J	178.9(3)	C00E	C00P	C010	C00U	1.2(6)
S003	C00E	C00P	C010	179.9(3)	C00F	S002	C00S	C00B	54.5(3)
S003	C00R	C00V	C00S	169.9(2)	C00F	S002	C00S	C00V	-69.0(3)
O004	C00B	C00S	S002	-90.8(3)	C00F	C00K	C00M	C00N	-1.7(5)
O004	C00B	C00S	C00V	32.4(4)	C00G	C00C	C00D	C00H	-0.3(5)
O005	S002	C00F	C00K	24.6(3)	C00G	C00R	C00V	C00S	45.8(4)
O005	S002	C00F	C00Z	-159.5(3)	C00H	C00I	C00L	C00G	1.5(5)
O005	S002	C00S	C00B	-61.1(2)	C00J	C00U	C010	C00P	-0.5(6)
O005	S002	C00S	C00V	175.4(2)	C00K	C00F	C00Z	C00W	2.7(5)
O006	S003	C00E	C00O	162.8(3)	C00K	C00M	C00N	Br01	-175.1(3)
O006	S003	C00E	C00P	-17.9(3)	C00K	C00M	C00N	C00W	4.2(6)
O006	S003	C00R	C00G	-50.6(3)	C00L	C00G	C00R	S003	124.0(3)
O006	S003	C00R	C00V	-175.8(2)	C00L	C00G	C00R	C00V	-113.1(3)
O007	S003	C00E	C00O	32.8(3)	C00M	C00N	C00W	C00Z	-3.1(6)
O007	S003	C00E	C00P	-147.9(3)	C00N	C00W	C00Z	C00F	-0.3(5)
O007	S003	C00R	C00G	80.0(3)	C00O	C00E	C00P	C010	-0.7(5)
O007	S003	C00R	C00V	-45.3(3)	C00O	C00J	C00U	C010	-0.7(6)
O008	S002	C00F	C00K	155.2(3)	C00P	C00E	C00O	C00J	-0.5(5)
O008	S002	C00F	C00Z	-28.9(3)	C00Q	N00A	C00B	O004	176.9(3)
O008	S002	C00S	C00B	170.0(2)	C00Q	N00A	C00B	C00S	-5.2(5)
O008	S002	C00S	C00V	46.6(3)	C00Q	N00A	C00T	C011	-52.8(4)
N00A	C00B	C00S	S002	91.2(3)	C00R	S003	C00E	C00O	-82.7(3)
N00A	C00B	C00S	C00V	-145.6(3)	C00R	S003	C00E	C00P	96.6(3)

Table S6 Torsion Angles for 10gb.

A	B	C	D	Angle/˚	A	B	C	D	Angle/˚
N00A	C00Q	C00X	O009	-59.4(4)	C00R	C00G	C00L	C00I	171.4(3)
N00A	C00T	C011	O009	54.2(4)	C00S	S002	C00F	C00K	-89.7(3)
C00B	N00A	C00Q	C00X	-129.2(3)	C00S	S002	C00F	C00Z	86.2(3)
C00B	N00A	C00T	C011	130.7(3)	C00T	N00A	C00B	O004	-7.1(5)
C00B	C00S	C00V	C00R	54.9(4)	C00T	N00A	C00B	C00S	170.8(3)
C00C	C00D	C00H	C00I	-0.6(5)	C00T	N00A	C00Q	C00X	54.6(4)
C00C	C00D	C00H	C00Y	176.6(3)	C00U	C00J	C00O	C00E	1.2(5)
C00C	C00G	C00L	C00I	-2.4(5)	C00X	O009	C011	C00T	-59.7(4)
C00C	C00G	C00R	S003	-62.4(4)	C00Y	C00H	C00I	C00L	-177.2(3)
C00C	C00G	C00R	C00V	60.5(4)	C00Z	C00F	C00K	C00M	-1.7(5)
C00D	C00C	C00G	C00L	1.8(5)	C011	O009	C00X	C00Q	62.9(4)

Table S7 Hydrogen Atom Coordinates (Å×10^4) and Isotropic Displacement Parameters (Å^2×10^3) for 10gb.

Atom	*x*	*y*	*z*	U(eq)
H00C	5613	3766	2877	20
H00D	6235	2175	2723	20
H00I	10282	2681	3404	22
H00J	4788	8648	3878	28
H00K	6623	5284	6209	22
H00L	9690	4295	3546	21
H00M	7152	6809	6617	24
H00O	4634	7075	3489	24
H00P	8609	7375	2649	28
H00A	8584	2550	4730	21
H00B	7015	2922	4967	21
H00R	8067	5520	3610	17
H00S	6270	3877	4341	17
H00E	10382	4734	5202	25
H00F	10745	3731	4863	25
H00U	6810	9583	3626	32
H00G	5945	5902	4146	20
H00H	5031	5093	3779	20
H00W	4502	8025	5281	26

Table S7 Hydrogen Atom Coordinates (Å×10^4) and Isotropic Displacement Parameters (Å^2×10^3) for 10gb.

Atom	*x*	*y*	*z*	U(eq)
H00N	7861	2978	6047	26
H00Q	8053	1916	5767	26
H00T	7881	888	2753	33
H00V	8802	926	3408	33
H00X	9583	1141	2733	33
H00Z	3909	6494	4888	23
H010	8715	8966	3009	33
H01A	11463	3633	5940	28
H01B	9885	3976	6178	28

Experimental

Single crystals of $C_{27}H_{28}BrNO_6S_2$ (**10gb**) were obtained by recrystallization from hexane/AcOEt. A suitable crystal was selected and mounted on a **Bruker D8 goniometer** diffractometer. The crystal was kept at 100.15 K during data collection. Using Olex2 [1], the structure was solved with the olex2.solve [2] structure solution program using Charge Flipping and refined with the SHELXL [3] refinement package using Least Squares minimisation.

1. Dolomanov, O.V., Bourhis, L.J., Gildea, R.J, Howard, J.A.K. & Puschmann, H. (2009), J. Appl. Cryst. 42, 339-341.
2. Bourhis, L.J., Dolomanov, O.V., Gildea, R.J., Howard, J.A.K., Puschmann, H. (2015). Acta Cryst. A71, 59-75.
3. Sheldrick, G.M. (2015). Acta Cryst. C71, 3-8.

Crystal structure determination of 10gb

Crystal Data for $C_{27}H_{28}BrNO_6S_2$ (M =606.53 g/mol): orthorhombic, space group $P2_12_12_1$ (no. 19), a = 9.1675(2) Å, b = 13.9765(3) Å, c = 20.7344(7) Å, V = 2656.69(12) Å^3, Z = 4, T = 100.15 K, μ (MoK α) = 1.748 mm^{-1}, $Dcalc$ = 1.516 g/cm^3, 40125 reflections measured (4.858° $\leq 2\Theta \leq$ 54.97°), 6026 unique (R_{int} = 0.0775, R_{sigma} = 0.0572) which were used in all calculations. The final R_1 was 0.0321 (I > 2 σ (I)) and wR_2 was 0.0668 (all data).

Refinement model description

Number of restraints - 0, number of constraints - unknown.

Details:

1. Fixed Uiso

At 1.2 times of:

All C(H) groups, All C(H,H) groups

At 1.5 times of:

All C(H,H,H) groups

2.a Ternary CH refined with riding coordinates:

C00R(H00R), C00S(H00S)

2.b Secondary CH2 refined with riding coordinates:
C00Q(H00A,H00B), C00T(H00E,H00F), C00V(H00G,H00H), C00X(H00N,H00Q), C011(H01A, H01B)

2.c Aromatic/amide H refined with riding coordinates:
C00C(H00C), C00D(H00D), C00I(H00I), C00J(H00J), C00K(H00K), C00L(H00L), C00M(H00M), C00O(H00O), C00P(H00P), C00U(H00U), C00W(H00W), C00Z(H00Z), C010(H010)

2.d Idealised Me refined as rotating group:
C00Y(H00T,H00V,H00X)

6-2. Report for 4fa

Table S8 Crystal data and structure refinement for 4fa.

Identification code	4fa
Empirical formula	$C_{29}H_{27}NO_3S$
Formula weight	469.57
Temperature/K	100
Crystal system	monoclinic
Space group	C2/c
a/Å	48.285(3)
b/Å	6.2118(4)
c/Å	16.7062(9)
α/°	90
β/°	106.244(2)
γ/°	90
Volume/Å^3	4810.8(5)
Z	8
ρ_{calc}g/cm^3	1.297
μ/mm^{-1}	0.166
F(000)	1984.0
Crystal size/mm^3	0.48 × 0.08 × 0.06
Radiation	MoKα (λ = 0.71073)
2Θ range for data collection/°	4.888 to 50.052
Index ranges	-56 ≤ h ≤ 56, -7 ≤ k ≤ 7, -19 ≤ l ≤ 19
Reflections collected	30674
Independent reflections	4256 [R_{int} = 0.0935, R_{sigma} = 0.0614]

Data/restraints/parameters	4256/0/309
Goodness-of-fit on F^2	1.064
Final R indexes [I>=2σ (I)]	R_1 = 0.0445, wR_2 = 0.0992
Final R indexes [all data]	R_1 = 0.0543, wR_2 = 0.1078
Largest diff. peak/hole / e $Å^{-3}$	0.36/-0.39

Table S9 Fractional Atomic Coordinates (×10^4) and Equivalent Isotropic Displacement Parameters (Å^2×10^3) for 4fa. U_{eq} is defined as 1/3 of the trace of the orthogonalised U_{IJ} tensor.

**Atom**	***x***	***y***	***z***	**U(eq)**
S001	3353.2(2)	2064.5(8)	1815.7(3)	18.01(16)
O002	3955.6(3)	6985(2)	3556.0(9)	20.7(3)
O003	3448.6(3)	2991(2)	1146.1(9)	25.7(4)
O004	3368.3(3)	-244(2)	1925.3(9)	23.5(3)
N005	4087.6(4)	5823(2)	4895.6(10)	16.5(4)
C006	3426.8(4)	2813(3)	3476.8(12)	15.7(4)
C007	4170.0(5)	4191(3)	5531.6(12)	16.6(4)
C008	3456.9(4)	771(3)	3831.5(12)	18.7(4)
C009	3554.2(4)	3358(3)	2771.8(12)	16.1(4)
C00A	4031.0(4)	5503(3)	4060.3(12)	15.7(4)
C00B	4455.6(5)	3569(3)	5837.7(13)	20.4(5)
C00C	3346.8(5)	326(3)	4495.9(13)	21.0(5)
C00D	3280.8(5)	4392(3)	3786.4(13)	19.9(5)
C00E	4073.0(4)	3222(3)	3778.6(12)	15.8(4)
C00F	3168.1(5)	3946(3)	4449.3(13)	22.1(5)
C00G	2989.4(5)	2841(3)	1674.5(12)	20.7(5)
C00H	4384.2(4)	2877(3)	3762.0(12)	16.5(4)
C00I	4526.9(5)	952(3)	4029.4(13)	21.0(5)
C00J	3201.5(5)	1915(3)	4802.5(13)	21.9(5)
C00K	3962.2(5)	3432(3)	5893.5(13)	19.8(5)
C00L	4532.8(5)	2153(3)	6509.7(13)	22.4(5)
C00M	3872.8(4)	2738(3)	2902.6(12)	17.7(4)
C00N	4054.2(5)	7969(3)	5223.3(13)	21.5(5)
C00O	4805.4(5)	615(3)	3975.8(13)	24.3(5)
C00P	2900.7(5)	4882(4)	1373.7(14)	29.2(5)
C00Q	4327.1(5)	1394(3)	6873.4(13)	24.9(5)

Table S9 Fractional Atomic Coordinates (×10[4]) and Equivalent Isotropic Displacement Parameters (Å[2]×10[3]) for 4fa. U_{eq} is defined as 1/3 of the trace of the orthogonalised U_{IJ} tensor.

Atom	*x*	*y*	*z*	U(eq)
C00R	2799.6(5)	1377(4)	1854.0(14)	29.1(5)
C00S	4041.8(5)	2036(3)	6566.2(13)	23.9(5)
C00T	4526.0(5)	4458(3)	3435.6(14)	26.1(5)
C00U	4945.4(5)	2194(4)	3657.4(15)	29.1(5)
C00V	2612.9(6)	5426(4)	1231.5(16)	38.9(6)
C00W	4804.2(5)	4108(4)	3385.3(16)	32.5(6)
C00X	2512.7(5)	1959(4)	1710.7(17)	39.1(6)
C00Y	2419.7(6)	3963(5)	1399.7(17)	42.6(7)

Table S10 Anisotropic Displacement Parameters (Å[2]×10[3]) for 4fa. The Anisotropic displacement factor exponent takes the form: $-2\pi^2[h^2a^{*2}U_{11}+2hka^*b^*U_{12}+\dots]$.

Atom	U_{11}	U_{22}	U_{33}	U_{23}	U_{13}	U_{12}
S001	17.2(3)	22.8(3)	14.0(3)	-2.26(19)	4.3(2)	-0.6(2)
O002	25.1(9)	18.3(7)	18.4(7)	3.7(6)	5.4(7)	1.9(6)
O003	25.8(9)	38.7(9)	14.1(7)	-1.7(6)	8.2(7)	-5.3(7)
O004	23.5(9)	22.4(8)	22.7(8)	-5.6(6)	3.0(7)	1.4(6)
N005	21.5(10)	13.6(8)	14.5(8)	-0.7(6)	5.1(7)	0.2(7)
C006	10.7(10)	20.9(10)	14.1(10)	-2.8(8)	0.9(8)	-1.7(8)
C007	21.8(12)	15.6(10)	11.7(9)	-3.5(8)	3.7(9)	-0.8(8)
C008	17.0(11)	19.7(10)	18.0(10)	-1.7(8)	2.8(9)	1.1(8)
C009	16.9(11)	17.6(10)	13.0(10)	-2.5(8)	2.9(9)	-0.6(8)
C00A	11.6(10)	18.8(10)	17.4(10)	1.2(8)	5.3(8)	-1.8(8)
C00B	20.5(12)	21.7(10)	18.9(11)	-1.4(8)	5.5(9)	-1.6(9)
C00C	18.9(12)	24.2(11)	18.6(11)	2.6(9)	3.0(9)	-3.1(9)
C00D	21.4(12)	19.5(10)	18.4(10)	-0.9(8)	4.6(9)	1.0(9)
C00E	17.6(11)	17.1(10)	13.3(10)	1.1(8)	5.4(9)	0.5(8)
C00F	20.9(12)	28.1(12)	18.3(11)	-6.1(9)	7.2(9)	2.6(9)
C00G	18.0(12)	26.9(11)	15.5(10)	-1.7(8)	2.0(9)	-0.9(9)
C00H	16.4(11)	20.8(10)	11.2(9)	-2.2(8)	2.0(9)	-0.6(8)
C00I	25.3(12)	20.3(11)	18.2(11)	-0.5(8)	7.4(10)	0.3(9)
C00J	17.6(12)	33.9(12)	14.6(10)	-2.5(9)	5.3(9)	-4.1(9)
C00K	20.5(12)	20.0(10)	20.4(11)	-3.4(8)	8.1(9)	-1.7(9)
C00L	22.7(12)	21.1(11)	18.9(11)	-1.6(8)	-1.4(9)	3.8(9)

Table S10 Anisotropic Displacement Parameters (Å^2×10^3) for 4fa. The Anisotropic displacement factor exponent takes the form: -2π^2[h^{2}a*^{2}U$_{11}$+2hka*b*U$_{12}$+…].

Atom	U$_{11}$	U$_{22}$	U$_{33}$	U$_{23}$	U$_{13}$	U$_{12}$
C00M	19.1(11)	20.1(10)	14.7(10)	-1.5(8)	6.0(9)	-0.5(8)
C00N	26.4(12)	16.8(10)	21.3(11)	-3.1(8)	6.8(10)	-0.3(9)
C00O	23.5(12)	23.6(11)	24.5(11)	-1.2(9)	4.4(10)	6.1(9)
C00P	29.7(14)	29.3(12)	28.3(12)	5.5(10)	7.4(11)	2.9(10)
C00Q	39.5(15)	18.8(10)	14.8(10)	0.6(8)	5.0(10)	2.0(10)
C00R	24.0(13)	31.2(12)	30.6(13)	2.6(10)	5.2(11)	-2.3(10)
C00S	34.9(14)	20.1(11)	20.2(11)	-1.3(9)	13.3(10)	-5.1(9)
C00T	20.1(12)	24.0(11)	36.1(13)	7.5(10)	11.1(11)	2.8(9)
C00U	18.5(12)	33.8(13)	35.7(13)	-1.8(10)	8.8(11)	3.2(10)
C00V	32.5(15)	42.0(15)	38.5(15)	7.0(12)	3.7(12)	14.8(12)
C00W	23.8(13)	29.5(13)	48.2(15)	6.6(11)	16.7(12)	-2.2(10)
C00X	18.5(13)	53.7(17)	42.9(15)	4.4(12)	4.9(12)	-5.0(12)
C00Y	20.3(14)	63.3(18)	40.7(15)	0.8(13)	2.8(12)	8.0(13)

Table S11 Bond Lengths for 4fa.

Atom	Atom	Length/Å	Atom	Atom	Length/Å
S001	O003	1.4435(14)	C00E	C00H	1.526(3)
S001	O004	1.4449(15)	C00E	C00M	1.541(3)
S001	C009	1.809(2)	C00F	C00J	1.383(3)
S001	C00G	1.772(2)	C00G	C00P	1.387(3)
O002	C00A	1.232(2)	C00G	C00R	1.382(3)
N005	C007	1.442(2)	C00H	C00I	1.390(3)
N005	C00A	1.359(2)	C00H	C00T	1.393(3)
N005	C00N	1.467(2)	C00I	C00O	1.388(3)
C006	C008	1.390(3)	C00K	C00S	1.386(3)
C006	C009	1.512(3)	C00L	C00Q	1.384(3)
C006	C00D	1.389(3)	C00O	C00U	1.380(3)
C007	C00B	1.385(3)	C00P	C00V	1.385(3)
C007	C00K	1.391(3)	C00Q	C00S	1.388(3)
C008	C00C	1.386(3)	C00R	C00X	1.386(3)
C009	C00M	1.541(3)	C00T	C00W	1.387(3)
C00A	C00E	1.524(3)	C00U	C00W	1.383(3)

Table S11 Bond Lengths for 4fa.

Atom	Atom	Length/Å	Atom	Atom	Length/Å
C00B	C00L	1.392(3)	C00V	C00Y	1.386(4)
C00C	C00J	1.389(3)	C00X	C00Y	1.375(4)
C00D	C00F	1.392(3)			

Table S12 Bond Angles for 4fa.

Atom	Atom	Atom	Angle/°	Atom	Atom	Atom	Angle/°
O003	S001	O004	118.56(9)	C00A	C00E	C00M	111.86(16)
O003	S001	C009	107.36(9)	C00H	C00E	C00M	108.33(15)
O003	S001	C00G	107.93(10)	C00J	C00F	C00D	119.79(19)
O004	S001	C009	109.48(9)	C00P	C00G	S001	119.42(17)
O004	S001	C00G	107.46(9)	C00R	C00G	S001	118.98(17)
C00G	S001	C009	105.28(9)	C00R	C00G	C00P	121.6(2)
C007	N005	C00N	113.60(15)	C00I	C00H	C00E	121.18(17)
C00A	N005	C007	126.06(16)	C00I	C00H	C00T	118.59(19)
C00A	N005	C00N	120.28(16)	C00T	C00H	C00E	120.15(18)
C008	C006	C009	121.30(17)	C00O	C00I	C00H	120.75(19)
C00D	C006	C008	119.27(17)	C00F	C00J	C00C	120.09(18)
C00D	C006	C009	119.41(17)	C00S	C00K	C007	119.6(2)
C00B	C007	N005	120.62(17)	C00Q	C00L	C00B	120.4(2)
C00B	C007	C00K	120.67(19)	C00E	C00M	C009	114.96(15)
C00K	C007	N005	118.34(19)	C00U	C00O	C00I	120.4(2)
C00C	C008	C006	120.37(18)	C00V	C00P	C00G	118.6(2)
C006	C009	S001	110.70(14)	C00L	C00Q	C00S	119.97(19)
C006	C009	C00M	116.19(16)	C00G	C00R	C00X	118.8(2)
C00M	C009	S001	106.37(13)	C00K	C00S	C00Q	120.10(19)
O002	C00A	N005	121.92(18)	C00W	C00T	C00H	120.3(2)
O002	C00A	C00E	121.51(17)	C00O	C00U	C00W	119.2(2)
N005	C00A	C00E	116.54(16)	C00P	C00V	C00Y	120.3(2)
C007	C00B	C00L	119.22(19)	C00U	C00W	C00T	120.8(2)
C008	C00C	C00J	119.99(19)	C00Y	C00X	C00R	120.4(2)
C006	C00D	C00F	120.48(19)	C00X	C00Y	C00V	120.2(2)
C00A	C00E	C00H	110.70(16)				

Table S13 Hydrogen Atom Coordinates (Å×10^4) and Isotropic Displacement Parameters (Å^2×10^3) for 4fa.

**Atom**	***x***	***y***	***z***	**U(eq)**
H008	3553.42	-325.02	3617.3	22
H009	3537.23	4950.01	2681.53	19
H00B	4597.21	4102.01	5592.07	24
H00C	3370.72	-1065.47	4741.61	25
H00D	3257.81	5787.66	3543.76	24
H00E	4028.69	2182.84	4182.87	19
H00F	3068.55	5032.06	4658.36	27
H00I	4432.87	-144.74	4251.38	25
H00J	3124.97	1606.51	5255.55	26
H00K	3766.73	3868.47	5680.89	24
H00L	4727.8	1705.99	6719.96	27
H00A	3882.99	1178.68	2791.84	21
H00G	3946.34	3517.42	2487.03	21
H00H	3980.84	8968.98	4758.52	32
H00M	4241.59	8477.07	5569.86	32
H00N	3917.85	7894.7	5560.54	32
H00O	4900.01	-710.42	4159.27	29
H00P	3034.55	5885.83	1267.43	35
H00Q	4381.25	432.33	7333.18	30
H00R	2864.67	-3.93	2071.73	35
H00S	3900.77	1518.92	6817.27	29
H00T	4431.65	5781.03	3246.65	31
H00U	5136.68	1969.27	3625.57	35
H00V	2547.43	6809.39	1017.71	47
H00W	4899	5197.04	3161.31	39
H00X	2379.47	966.58	1828.16	47
H00Y	2222.49	4345.79	1299.7	51

Experimental

Single crystals of $C_{29}H_{27}NO_3S$ (**4fa**) were obtained by recrystallization from hexane/methanol. A suitable crystal was selected and mounted on a **Bruker D8 goniometer** diffractometer. The crystal was kept at 100 K during data collection. Using Olex2 [1], the structure was solved with the SHELXT [2] structure solution program using Intrinsic Phasing and refined with the SHELXL [3] refinement package using Least Squares minimisation.

1. Dolomanov, O.V., Bourhis, L.J., Gildea, R.J, Howard, J.A.K. & Puschmann, H. (2009), J. Appl. Cryst. 42, 339-341.
2. Sheldrick, G.M. (2015). Acta Cryst. A71, 3-8.
3. Sheldrick, G.M. (2015). Acta Cryst. C71, 3-8.

Crystal structure determination of 4fa

Crystal Data for $C_{29}H_{27}NO_3S$ (M =469.57 g/mol): monoclinic, space group C2/c (no. 15), a = 48.285(3) Å, b = 6.2118(4) Å, c = 16.7062(9) Å, β = 106.244(2)°, V = 4810.8(5) Å^3, Z = 8, T = 100 K, μ(MoKα) = 0.166 mm^{-1}, $Dcalc$ = 1.297 g/cm^3, 30674 reflections measured (4.888° ≤ 2Θ ≤ 50.052°), 4256 unique (R_{int} = 0.0935, R_{sigma} = 0.0614) which were used in all calculations. The final R_1 was 0.0445 (I > 2σ(I)) and wR_2 was 0.1078 (all data).

Refinement model description

Number of restraints - 0, number of constraints - unknown.

Details:

1.a Ternary CH refined with riding coordinates:
C009(H009), C00E(H00E)

1.b Secondary CH2 refined with riding coordinates:
C00M(H00A,H00G)

1.c Aromatic/amide H refined with riding coordinates:
C008(H008), C00B(H00B), C00C(H00C), C00D(H00D), C00F(H00F), C00I(H00I), C00J(H00J), C00K(H00K), C00L(H00L), C00O(H00O), C00P(H00P), C00Q(H00Q), C00R(H00R), C00S(H00S), C00T(H00T), C00U(H00U), C00V(H00V), C00W(H00W), C00X(H00X), C00Y(H00Y)

1.d Idealised Me refined as rotating group:
C00N(H00H,H00M,H00N)

6-3. Report for 10gn

Table S14 Crystal data and structure refinement for 10gn.

Identification code	10gb
Empirical formula	$C_{22.04}H_{25.08}Br_2NO_{6.51}S_2$
Formula weight	632.11
Temperature/K	100(2)
Crystal system	orthorhombic
Space group	Pbca
a/Å	19.7839(8)
b/Å	10.7284(4)
c/Å	23.4186(9)
α/°	90

β /°	90
γ /°	90
Volume/Å3	4970.6(3)
Z	8
ρ calcg/cm3	1.689
μ /mm-1	6.066
F(000)	2547.0
Crystal size/mm3	0.2 × 0.17 × 0.01
Radiation	CuKα (λ = 1.54184)
2Θ range for data collection/°	7.55 to 149.726
Index ranges	-24 ≤ h ≤ 24, -13 ≤ k ≤ 13, -29 ≤ l ≤ 29
Reflections collected	124321
Independent reflections	5099 [Rint = 0.0713, Rsigma = 0.0277]
Data/restraints/parameters	5099/0/328
Goodness-of-fit on F2	1.050
Final R indexes [I>=2σ (I)]	R1 = 0.0300, wR2 = 0.0787
Final R indexes [all data]	R1 = 0.0304, wR2 = 0.0792
Largest diff. peak/hole / e Å-3	1.74/-0.79

Table S15 Fractional Atomic Coordinates (×10^4) and Equivalent Isotropic Displacement Parameters (Å^2× 10^3) for 10gn. U_{eq} is defined as 1/3 of the trace of the orthogonalised U_{IJ} tensor.

**Atom**	***x***	***y***	***z***	**U(eq)**
Br01	4630.0(2)	5100.1(2)	8727.9(2)	24.25(8)
Br02	7399.8(2)	9526.4(2)	5796.0(2)	28.75(9)
S003	5963.8(2)	4891.7(4)	7002.7(2)	13.66(11)
S004	5811.0(2)	6666.7(4)	9177.6(2)	13.66(11)
O005	5078.3(7)	7552.8(13)	7342.2(6)	16.3(3)
O006	6481.8(8)	4213.5(14)	7304.0(7)	20.2(3)
O007	5576.9(8)	4269.0(14)	6566.9(6)	20.5(3)
O008	6292.9(8)	7590.5(14)	8989.6(6)	20.0(3)
O009	5355.3(7)	6963.0(14)	9637.3(6)	19.8(3)
O00A	2957.9(8)	5678.9(15)	6862.3(7)	23.1(3)
N00B	4366.0(8)	6091.4(15)	6985.2(7)	14.3(3)
C00C	6257.3(10)	5294.6(19)	9356.0(8)	14.4(4)
C00D	6362.0(10)	7748(2)	5951.6(9)	17.8(4)

Table S15 Fractional Atomic Coordinates (×10^4) and Equivalent Isotropic Displacement Parameters (Å^2×10^3) for 10gn. U_{eq} is defined as 1/3 of the trace of the orthogonalised U_{IJ} tensor.

**Atom**	***x***	***y***	***z***	**U(eq)**
C00E	5375.8(9)	5448.8(17)	7535.9(8)	11.6(4)
C00F	5767.6(10)	5929.8(18)	8055.1(8)	13.1(4)
C00G	4915.2(10)	6451.3(17)	7279.0(8)	12.0(4)
C00H	6342.3(10)	6224.2(18)	6698.3(8)	13.9(4)
C00I	5315.4(10)	6335.7(19)	8548.0(8)	14.7(4)
C00J	6965.2(11)	8187.2(19)	6172.7(9)	17.3(4)
C00K	6045.0(10)	6755(2)	6223.4(9)	16.6(4)
C00L	6907.4(11)	5128(2)	9138.3(9)	18.6(4)
C00M	6945.6(10)	6677(2)	6922.8(9)	17.6(4)
C00N	3932.5(10)	7027.5(18)	6713.1(9)	16.2(4)
C00O	5965.6(11)	4432(2)	9723.2(9)	18.7(4)
C00P	4098.3(11)	4823.5(19)	6914.1(10)	18.9(4)
C00Q	3378.5(12)	4781(2)	7137.8(10)	22.8(4)
C00R	6979.1(13)	3204(2)	9660.8(10)	26.6(5)
C00S	7265.4(12)	4074(2)	9293.7(10)	24.1(5)
C00T	7260.0(11)	7681(2)	6658.8(9)	20.0(4)
C00U	3222.1(11)	6906(2)	6949.0(10)	20.5(4)
C00V	6330.3(12)	3382(2)	9875.5(10)	24.3(5)
C1	4821(7)	5277(18)	5228(8)	22(3)
O3	5237.2(17)	4193(4)	4403.7(15)	31.5(9)
C2	5928(17)	4910(20)	5012(9)	37(6)
C3	5360(6)	4760(16)	4843(6)	15(3)
C4	3961(16)	4990(20)	5006(8)	30(4)

Table S16 Anisotropic Displacement Parameters (Å^2×10^3) for 10gn. The Anisotropic displacement factor exponent takes the form: $-2\pi^2[h^2a^{*2}U_{11}+2hka^*b^*U_{12}+\cdots]$.

**Atom**	**U_{11}**	**U_{22}**	**U_{33}**	**U_{23}**	**U_{13}**	**U_{12}**
Br01	20.97(13)	33.75(15)	18.04(13)	-1.97(9)	3.15(8)	-13.37(9)
Br02	35.53(15)	26.15(14)	24.58(14)	7.47(9)	-1.58(9)	-14.16(10)
S003	16.0(2)	10.4(2)	14.5(2)	-1.37(16)	2.16(17)	0.86(17)
S004	15.2(2)	13.3(2)	12.4(2)	-1.64(16)	-0.45(16)	-1.43(17)
O005	18.8(7)	8.8(6)	21.3(7)	-1.5(5)	-0.9(6)	-1.1(5)
O006	20.5(7)	16.5(7)	23.7(7)	3.6(6)	3.8(6)	6.9(6)

Table S16 Anisotropic Displacement Parameters (Å^2×10^3) for 10gn. The Anisotropic displacement factor exponent takes the form: $-2\pi^2[h^2a^{*2}U_{11}+2hka^*b^*U_{12}+\cdots]$.

**Atom**	**U_{11}**	**U_{22}**	**U_{33}**	**U_{23}**	**U_{13}**	**U_{12}**
O007	25.0(8)	17.6(7)	19.0(7)	-6.4(6)	3.2(6)	-5.5(6)
O008	23.4(7)	15.6(7)	20.9(7)	1.5(6)	-2.7(6)	-6.5(6)
O009	21.3(7)	21.8(7)	16.4(7)	-4.7(6)	2.8(6)	2.8(6)
O00A	17.5(7)	20.6(8)	31.1(9)	-2.7(6)	-7.1(6)	-2.1(6)
N00B	16.2(8)	8.5(7)	18.3(8)	-0.2(6)	-3.6(6)	0.9(6)
C00C	15.8(9)	14.8(9)	12.7(9)	-1.5(7)	-1.7(7)	-0.1(7)
C00D	20.0(10)	20.0(10)	13.3(9)	1.5(8)	-1.1(8)	0.1(8)
C00E	12.4(9)	10.9(8)	11.6(9)	0.0(7)	0.9(7)	-0.4(7)
C00F	12.3(8)	14.0(9)	13.1(9)	-0.1(7)	-0.6(7)	-1.3(7)
C00G	13.7(9)	10.8(8)	11.4(8)	-0.2(7)	2.2(7)	0.7(7)
C00H	14.5(9)	13.8(9)	13.4(9)	-1.1(7)	2.9(7)	0.2(7)
C00I	13.3(9)	17.1(9)	13.6(9)	-0.1(7)	-0.6(7)	-2.5(7)
C00J	20.3(10)	16.0(9)	15.5(9)	0.5(8)	2.5(8)	-2.0(8)
C00K	14.4(9)	20.5(10)	14.8(9)	-2.9(8)	-1.6(7)	-1.4(8)
C00L	15.9(10)	25.0(11)	14.8(10)	-1.6(8)	1.3(8)	-1.7(8)
C00M	17.1(9)	20.2(10)	15.5(9)	1.4(8)	-2.1(8)	-0.2(8)
C00N	17.9(9)	13.1(9)	17.6(9)	2.1(7)	-3.5(8)	3.2(8)
C00O	19.4(10)	19.7(10)	17.0(10)	-0.8(8)	2.2(8)	-2.3(8)
C00P	21.3(10)	10.0(9)	25.3(11)	-3.4(8)	-8.3(9)	-1.1(8)
C00Q	22.4(11)	16.8(9)	29.1(12)	1.7(9)	-7.3(9)	-5.6(8)
C00R	33.6(12)	19.8(10)	26.4(12)	-3.6(9)	-7.5(10)	8.2(9)
C00S	19.8(10)	32.0(12)	20.5(10)	-7.1(9)	-1.9(8)	6.0(9)
C00T	18.1(10)	22.9(10)	18.9(10)	0.2(8)	-2.9(8)	-5.4(8)
C00U	18.2(10)	18.1(10)	25.2(11)	-3.3(8)	-2.1(8)	1.0(8)
C00V	33.7(12)	16.8(10)	22.4(11)	2.4(8)	0.4(9)	-1.5(9)
C1	19(7)	22(3)	23(4)	0(3)	-9(4)	-3(5)
O3	24.7(17)	47(2)	22.8(17)	-14.1(16)	-4.4(14)	-0.5(15)
C2	62(13)	13(4)	37(6)	-8(4)	26(6)	-4(5)
C3	15(7)	16(3)	15(4)	1(3)	-4(4)	-2(4)
C4	43(8)	26(7)	21(5)	6(3)	-1(5)	-2(5)

Table S17 Bond Lengths for 10gn.

Atom	Atom	Length/Å	Atom	Atom	Length/Å
Br01	C00I	1.942(2)	C00D	C00K	1.390(3)
Br02	C00J	1.892(2)	C00E	C00F	1.531(3)
S003	O006	1.4413(15)	C00E	C00G	1.533(3)
S003	O007	1.4401(15)	C00F	C00I	1.524(3)
S003	C00E	1.8084(19)	C00H	C00K	1.381(3)
S003	C00H	1.764(2)	C00H	C00M	1.392(3)
S004	O008	1.4440(15)	C00J	C00T	1.390(3)
S004	O009	1.4397(15)	C00L	C00S	1.382(3)
S004	C00C	1.767(2)	C00M	C00T	1.389(3)
S004	C00I	1.806(2)	C00N	C00U	1.516(3)
O005	C00G	1.234(2)	C00O	C00V	1.385(3)
O00A	C00Q	1.427(3)	C00P	C00Q	1.518(3)
O00A	C00U	1.431(3)	C00R	C00S	1.390(4)
N00B	C00G	1.343(3)	C00R	C00V	1.392(3)
N00B	C00N	1.466(2)	C1	C3	1.502(8)
N00B	C00P	1.469(2)	C1	C4	1.81(4)
C00C	C00L	1.395(3)	O3	C3	1.219(15)
C00C	C00O	1.389(3)	C2	C3	1.20(4)
C00D	C00J	1.384(3)			

Table S18 Bond Angles for 10gn.

Atom	Atom	Atom	Angle/˚	Atom	Atom	Atom	Angle/˚
O006	S003	C00E	106.63(9)	N00B	C00G	C00E	118.72(16)
O006	S003	C00H	107.77(9)	C00K	C00H	S003	118.60(15)
O007	S003	O006	119.38(9)	C00K	C00H	C00M	121.70(19)
O007	S003	C00E	107.50(9)	C00M	C00H	S003	119.62(16)
O007	S003	C00H	108.37(9)	S004	C00I	Br01	109.64(10)
C00H	S003	C00E	106.52(9)	C00F	C00I	Br01	112.27(13)
O008	S004	C00C	108.30(9)	C00F	C00I	S004	110.85(13)
O008	S004	C00I	104.15(9)	C00D	C00J	Br02	118.43(16)
O009	S004	O008	119.33(9)	C00D	C00J	C00T	122.36(19)
O009	S004	C00C	108.67(9)	C00T	C00J	Br02	119.21(16)
O009	S004	C00I	108.30(9)	C00H	C00K	C00D	119.50(19)

Table S18 Bond Angles for 10gn.

Atom	Atom	Atom	Angle/°	Atom	Atom	Atom	Angle/°
C00C	S004	C00I	107.49(9)	C00S	C00L	C00C	118.8(2)
C00Q	O00A	C00U	110.11(16)	C00T	C00M	C00H	119.13(19)
C00G	N00B	C00N	119.94(16)	N00B	C00N	C00U	108.98(17)
C00G	N00B	C00P	128.02(17)	C00V	C00O	C00C	119.0(2)
C00N	N00B	C00P	111.96(16)	N00B	C00P	C00Q	109.06(17)
C00L	C00C	S004	118.77(16)	O00A	C00Q	C00P	111.78(18)
C00O	C00C	S004	119.60(16)	C00S	C00R	C00V	120.5(2)
C00O	C00C	C00L	121.6(2)	C00L	C00S	C00R	120.2(2)
C00J	C00D	C00K	118.61(19)	C00M	C00T	C00J	118.67(19)
C00F	C00E	S003	109.51(13)	O00A	C00U	C00N	111.45(17)
C00F	C00E	C00G	112.10(15)	C00O	C00V	C00R	120.0(2)
C00G	C00E	S003	110.08(13)	C3	C1	C4	115.6(11)
C00I	C00F	C00E	113.61(16)	O3	C3	C1	123.3(9)
O005	C00G	N00B	123.26(18)	C2	C3	C1	114.7(13)
O005	C00G	C00E	118.00(17)	C2	C3	O3	121.9(16)

Table S19 Torsion Angles for 10gb.

A	B	C	D	Angle/°	A	B	C	D	Angle/°
Br02	C00J	C00T	C00M	-178.33(16)	C00E	S003	C00H	C00M	92.39(17)
S003	C00E	C00F	C00I	-175.85(13)	C00E	C00F	C00I	Br01	48.40(19)
S003	C00E	C00G	O005	-94.83(18)	C00E	C00F	C00I	S004	171.40(13)
S003	C00E	C00G	N00B	83.49(19)	C00F	C00E	C00G	O005	27.3(2)
S003	C00H	C00K	C00D	-175.22(16)	C00F	C00E	C00G	N00B	-154.36(17)
S003	C00H	C00M	C00T	176.17(16)	C00G	N00B	C00N	C00U	-121.30(19)
S004	C00C	C00L	C00S	177.80(17)	C00G	N00B	C00P	C00Q	121.5(2)
S004	C00C	C00O	C00V	-177.74(17)	C00G	C00E	C00F	C00I	61.7(2)
O006	S003	C00E	C00F	41.74(15)	C00H	S003	C00E	C00F	-73.14(15)
O006	S003	C00E	C00G	165.41(13)	C00H	S003	C00E	C00G	50.52(15)
O006	S003	C00H	C00K	155.23(16)	C00H	C00M	C00T	C00J	-1.0(3)
O006	S003	C00H	C00M	-21.72(19)	C00I	S004	C00C	C00L	92.20(17)
O007	S003	C00E	C00F	170.87(13)	C00I	S004	C00C	C00O	-90.04(18)
O007	S003	C00E	C00G	-65.46(15)	C00J	C00D	C00K	C00H	-1.0(3)
O007	S003	C00H	C00K	24.74(19)	C00K	C00D	C00J	Br02	179.30(15)

Table S19 Torsion Angles for 10gb.

A	B	C	D	Angle/˚	A	B	C	D	Angle/˚
O007	S003	C00H	C00M	-152.21(16)	C00K	C00D	C00J	C00T	-0.7(3)
O008	S004	C00C	C00L	-19.77(19)	C00K	C00H	C00M	C00T	-0.7(3)
O008	S004	C00C	C00O	157.99(16)	C00L	C00C	C00O	C00V	0.0(3)
O008	S004	C00I	Br01	-179.70(10)	C00M	C00H	C00K	C00D	1.7(3)
O008	S004	C00I	C00F	55.79(16)	C00N	N00B	C00G	O005	0.9(3)
O009	S004	C00C	C00L	-150.80(16)	C00N	N00B	C00G	C00E	-177.30(17)
O009	S004	C00C	C00O	26.96(19)	C00N	N00B	C00P	C00Q	-55.2(2)
O009	S004	C00I	Br01	-51.71(12)	C00O	C00C	C00L	C00S	0.1(3)
O009	S004	C00I	C00F	-176.22(13)	C00P	N00B	C00G	O005	-175.56(19)
N00B	C00N	C00U	O00A	-57.4(2)	C00P	N00B	C00G	C00E	6.2(3)
N00B	C00P	C00Q	O00A	56.5(2)	C00P	N00B	C00N	C00U	55.7(2)
C00C	S004	C00I	Br01	65.53(12)	C00Q	O00A	C00U	C00N	59.5(2)
C00C	S004	C00I	C00F	-58.98(16)	C00S	C00R	C00V	C00O	0.0(3)
C00C	C00L	C00S	C00R	-0.1(3)	C00U	O00A	C00Q	C00P	-59.1(2)
C00C	C00O	C00V	C00R	0.0(3)	C00V	C00R	C00S	C00L	0.0(3)
C00D	C00J	C00T	C00M	1.7(3)	C4	C1	C3	O3	0.9(10)
C00E	S003	C00H	C00K	-90.66(17)	C4	C1	C3	C2	-176(3)

Table S20 Hydrogen Atom Coordinates (Å×10^4) and Isotropic Displacement Parameters (Å^2×10^3) for 10gn.

Atom	*x*	*y*	*z*	U(eq)
H00D	6172.62	8108.53	5627.92	21
H00E	5093.52	4747.34	7658.41	14
H00A	6068.66	5277.99	8188.19	16
H00B	6044.1	6631.55	7938.2	16
H00I	5084.76	7105.23	8434.56	18
H00K	5635.74	6450.73	6086.44	20
H00L	7096.36	5713.79	8893.1	22
H00M	7135.58	6312.84	7245.37	21
H00C	3927.86	6901.21	6302.93	19
H00F	4106.42	7856.3	6789.42	19
H00O	5531.72	4558.18	9864.69	22
H00G	4376.96	4235.68	7122.99	23
H00H	4105.49	4593.38	6513.6	23

Table S20 Hydrogen Atom Coordinates (Å×10^4) and Isotropic Displacement Parameters (Å^2×10^3) for 10gn.

Atom	*x*	*y*	*z*	U(eq)
H00J	3193.59	3954.94	7076.38	27
H00N	3380.97	4939.86	7545.56	27
H00R	7222.77	2497.8	9763.62	32
H00S	7699.27	3947.89	9152.26	29
H00T	7659.88	8007.66	6804.44	24
H00P	3226.36	7092.27	7354.36	25
H00Q	2930.14	7508.27	6762.69	25
H00V	6141.93	2796.34	10121.08	29
H1A	4885.22	6170.55	5257.84	26
H1B	4883.2	4926.51	5605.93	26
H2A	6026.5	5779.95	5033.51	56
H2B	6238.34	4511.6	4753.4	56
H2C	5973.1	4539	5384.07	56
H4A	3899.23	5276.58	4621.91	45
H4B	3656.18	5419.12	5255.71	45
H4C	3870.68	4107.53	5022.8	45

Table S21 Atomic Occupancy for 10gn.

Atom	Occupancy	Atom	Occupancy	Atom	Occupancy
C1	0.510(3)	H1A	0.510(3)	H1B	0.510(3)
O3	0.510(3)	C2	0.510(3)	H2A	0.510(3)
H2B	0.510(3)	H2C	0.510(3)	C3	0.510(3)
C4	0.510(3)	H4A	0.510(3)	H4B	0.510(3)
H4C	0.510(3)				

Experimental

Single crystals of $C_{22.04}H_{25.08}Br_2NO_{6.51}S_2$ (**10gn**) were obtained by recrystallization from hexane/2-butanone. A suitable crystal was selected and mounted on a **Bruker D8 venture** diffractometer. The crystal was kept at 100(2) K during data collection. Using Olex2 [1], the structure was solved with the SHELXT [2] structure solution program using Intrinsic Phasing and refined with the SHELXL [3] refinement package using Least Squares minimisation.

1. Dolomanov, O.V., Bourhis, L.J., Gildea, R.J, Howard, J.A.K. & Puschmann, H. (2009), J. Appl. Cryst. 42, 339-341.

2. Bourhis, L.J., Dolomanov, O.V., Gildea, R.J., Howard, J.A.K., Puschmann, H. (2015). Acta Cryst. A71, 59-75.
3. Sheldrick, G.M. (2015). Acta Cryst. C71, 3-8.

Crystal structure determination of 10gn

Crystal Data for $C_{22.04125}H_{25.0825}Br_2NO_{6.51}S_2$ (*M* =632.11 g/mol): orthorhombic, space group Pbca (no. 61), *a* = 19.7839(8) Å, *b* = 10.7284(4) Å, *c* = 23.4186(9) Å, *V* = 4970.6(3) Å^3, *Z* = 8, *T* = 100(2) K, μ (CuK α) = 6.066 mm^{-1}, *Dcalc* = 1.689 g/cm^3, 124321 reflections measured (7.55° ≤ 2Θ ≤ 149.726°), 5099 unique (R_{int} = 0.0713, R_{sigma} = 0.0277) which were used in all calculations. The final R_1 was 0.0300 (I > 2 σ (I)) and wR_2 was 0.0792 (all data).

Refinement model description

Number of restraints - 0, number of constraints - unknown.

Details:

1. Fixed Uiso
  At 1.2 times of:
    All C(H) groups, All C(H,H) groups
  At 1.5 times of:
    All C(H,H,H) groups
2. Others
  Sof(C1)=Sof(H1A)=Sof(H1B)=Sof(O3)=Sof(C2)=Sof(H2A)=Sof(H2B)=Sof(H2C)=Sof(C3)=
  Sof(C4)=Sof(H4A)=Sof(H4B)=Sof(H4C)=FVAR(1)

3.a Ternary CH refined with riding coordinates:
  C00E(H00E), C00I(H00I)

3.b Secondary CH2 refined with riding coordinates:
  C00F(H00A,H00B), C00N(H00C,H00F), C00P(H00G,H00H), C00Q(H00J,H00N), C00U(H00P,
  H00Q), C1(H1A,H1B)

3.c Aromatic/amide H refined with riding coordinates:
  C00D(H00D), C00K(H00K), C00L(H00L), C00M(H00M), C00O(H00O), C00R(H00R),
  C00S(H00S), C00T(H00T), C00V(H00V)

3.d Idealised Me refined as rotating group:
  C2(H2A,H2B,H2C), C4(H4A,H4B,H4C)

6-4. Report for 9aa

Table S22 Crystal data and structure refinement for 9aa.

Identification code	9aa
Empirical formula	$C_{28}H_{31}NO_5S$
Formula weight	493.60
Temperature/K	100.15

Crystal system	orthorhombic
Space group	$P2_12_12_1$
a/Å	11.0610(4)
b/Å	12.1022(3)
c/Å	18.7914(6)
α/°	90
β/°	90
γ/°	90
Volume/Å^3	2515.46(14)
Z	4
ρ_{calc}g/cm^3	1.303
μ/mm^{-1}	0.168
F(000)	1048.0
Crystal size/mm^3	0.244 × 0.178 × 0.082
Radiation	MoKα (λ = 0.71073)
2Θ range for data collection/°	4.272 to 54.966
Index ranges	-12 ≤ h ≤ 13, -14 ≤ k ≤ 15, -21 ≤ l ≤ 24
Reflections collected	37895
Independent reflections	5691 [R_{int} = 0.0782, R_{sigma} = 0.0492]
Data/restraints/parameters	5691/432/321
Goodness-of-fit on F^2	1.027
Final R indexes [I>=2σ (I)]	R_1 = 0.0397, wR_2 = 0.0857
Final R indexes [all data]	R_1 = 0.0453, wR_2 = 0.0898
Largest diff. peak/hole / e Å^{-3}	0.24/-0.29
Flack parameter	-0.03(4)

Table S23 Fractional Atomic Coordinates (×10^4) and Equivalent Isotropic Displacement Parameters (Å^2× 10^3) for 9aa. U_{eq} is defined as 1/3 of the trace of the orthogonalised U_{IJ} tensor.

**Atom**	***x***	***y***	***z***	**U(eq)**
S001	4091.5(6)	815.1(5)	6426.2(3)	20.30(15)
O002	1892.6(16)	4334.9(14)	8054.3(9)	20.0(4)
O003	4634.7(19)	-106.5(14)	6789.6(10)	29.0(5)
O004	6112.2(17)	1784.5(15)	7534.0(10)	27.7(4)
O005	1494.5(19)	2933.1(17)	7297.0(11)	32.9(5)
O006	2852.3(18)	733.7(16)	6205.2(10)	31.0(5)

Table S23 Fractional Atomic Coordinates (×10^4) and Equivalent Isotropic Displacement Parameters (Å^2× 10^3) for 9aa. U_{eq} is defined as 1/3 of the trace of the orthogonalised U_{IJ} tensor.

**Atom**	***x***	***y***	***z***	**U(eq)**
N007	5943.5(19)	3284.7(15)	6834.7(11)	18.5(4)
C008	5501(2)	2353.9(19)	7135.6(13)	18.8(5)
C009	2094(2)	3341(2)	7765.2(13)	19.7(5)
C00A	5368(2)	3981.2(18)	6316.4(13)	18.1(5)
C00B	2876(2)	2488.4(19)	8877.9(13)	20.1(5)
C00C	3536(2)	1749.5(19)	7685.3(13)	19.0(5)
C00D	6214(2)	924(2)	5690.7(13)	21.4(5)
C00E	4975(2)	1127.7(18)	5672.5(13)	18.7(5)
C00F	4511(3)	5483(2)	5322.4(14)	26.1(6)
C00G	5872(3)	4026(2)	5638.2(13)	23.4(5)
C00H	7173(2)	3616(2)	7041.4(16)	25.7(6)
C00I	4189(2)	2029.9(18)	6986.8(12)	17.4(5)
C00J	4417(3)	1553(2)	5070.1(14)	25.1(6)
C00K	3549(3)	2918(2)	9435.5(14)	24.9(6)
C00L	5451(3)	4781(2)	5143.7(14)	27.1(6)
C00M	3181(2)	2783.4(19)	8111.6(13)	18.4(5)
C00N	3998(3)	5432(2)	5990.0(14)	24.7(6)
C00O	1937(3)	1763(2)	9033.8(15)	28.2(6)
C00P	6895(3)	1173(2)	5092.6(15)	29.0(6)
C00Q	1680(3)	1476(2)	9732.1(16)	33.5(7)
C00R	4429(2)	4686.0(19)	6495.5(14)	21.1(5)
C00S	3284(3)	2633(2)	10136.3(15)	32.4(7)
C00T	2353(3)	1914(2)	10286.4(15)	33.8(7)
C00U	797(3)	4973(2)	7866.0(14)	29.2(6)
C00V	6346(3)	1596(2)	4489.9(15)	31.8(7)
C00W	5119(3)	1773(2)	4477.7(15)	30.6(6)
C00X	882(3)	5972(2)	8343.1(15)	30.8(6)
C00Y	-312(3)	4301(3)	8047(2)	52.8(10)
C00Z	892(4)	5349(3)	7094.2(17)	62.1(12)

Table S24 Anisotropic Displacement Parameters (Å^2×10^3) for 9aa. The Anisotropic displacement factor exponent takes the form: -2π^2[h^{2}a*^{2}U$_{11}$+2hka*b*U$_{12}$+⋯].

**Atom**	**U_{11}**	**U_{22}**	**U_{33}**	**U_{23}**	**U_{13}**	**U_{12}**
S001	22.1(3)	16.2(3)	22.6(3)	-2.2(2)	4.4(3)	-2.1(2)
O002	19.1(9)	19.2(8)	21.8(9)	-2.0(7)	-2.7(7)	3.7(7)
O003	44.2(13)	17.3(8)	25.4(10)	2.2(7)	9.7(9)	4.0(8)
O004	22.9(11)	28.8(9)	31.3(10)	9.4(8)	-4.3(8)	1.5(8)
O005	29.7(12)	38.0(11)	30.9(11)	-17.6(9)	-10.5(9)	10.7(9)
O006	21.4(11)	33.2(10)	38.4(11)	-13.0(9)	4.8(8)	-8.1(8)
N007	15.9(11)	17.1(9)	22.4(10)	-1.4(8)	-1.4(9)	-3.4(8)
C008	19.3(13)	18.5(11)	18.6(11)	-1.1(9)	1.0(10)	1.8(9)
C009	19.8(13)	22.0(11)	17.2(11)	-2.0(9)	2.2(10)	1.8(10)
C00A	20.2(12)	14.3(10)	19.8(11)	-1.0(8)	-2.7(10)	-4.3(8)
C00B	19.6(13)	18.1(11)	22.4(12)	0.2(9)	0.7(10)	3.9(9)
C00C	20.3(13)	16.9(11)	19.8(11)	0.9(9)	3.7(10)	0.3(9)
C00D	21.7(14)	21.3(11)	21.4(12)	-0.9(10)	0.5(10)	3.9(9)
C00E	22.6(13)	14.7(10)	18.8(11)	-2.5(9)	-1.4(10)	0.0(9)
C00F	32.3(16)	22.2(12)	23.7(12)	2.7(10)	-7.5(11)	-5.0(10)
C00G	24.2(14)	21.6(11)	24.2(12)	-2.5(9)	3.0(11)	-2.4(10)
C00H	18.9(15)	20.7(12)	37.7(15)	-2.1(11)	-5.0(12)	-3.3(10)
C00I	18.1(13)	15.8(10)	18.2(11)	-0.6(8)	1.7(10)	1.0(9)
C00J	28.6(16)	21.7(11)	25.1(13)	0.5(10)	-7.2(11)	-0.8(10)
C00K	26.9(15)	23.3(12)	24.5(13)	0.7(10)	-0.7(11)	3.0(10)
C00L	32.2(16)	26.1(12)	22.9(12)	1.3(10)	2.5(11)	-6.2(11)
C00M	18.5(13)	18.2(10)	18.4(11)	-0.9(9)	0.2(10)	0.2(9)
C00N	27.0(14)	19.1(11)	27.8(13)	-0.6(9)	-1.9(12)	1.9(10)
C00O	27.7(15)	27.9(12)	29.0(13)	-1.1(11)	4.9(12)	-1.5(11)
C00P	27.9(16)	29.4(13)	29.6(14)	-6.7(11)	7.3(12)	0.2(11)
C00Q	37.1(17)	28.0(13)	35.5(15)	5.8(11)	12.0(13)	1.6(12)
C00R	22.1(14)	19.4(10)	21.9(12)	-1.2(9)	0.3(10)	-2.7(9)
C00S	41.3(18)	32.6(14)	23.2(14)	-2.5(11)	-3.2(13)	6.5(12)
C00T	46.3(18)	30.7(13)	24.3(13)	5.3(11)	10.4(13)	13.5(12)
C00U	24.4(15)	34.6(13)	28.5(13)	-8.0(11)	-7.1(12)	15.3(12)
C00V	43.9(18)	31.5(14)	20.0(13)	-3.0(11)	8.2(12)	-9.0(12)
C00W	45.8(18)	26.0(13)	20.0(13)	3.2(10)	-8.0(12)	-4.6(12)

Table S24 Anisotropic Displacement Parameters (Å^2×10^3) for 9aa. The Anisotropic displacement factor exponent takes the form: -2π^2[h^{2}a*^{2}U$_{11}$+2hka*b*U$_{12}$+⋯].

**Atom**	**U$_{11}$**	**U$_{22}$**	**U$_{33}$**	**U$_{23}$**	**U$_{13}$**	**U$_{12}$**
C00X	30.2(16)	27.1(13)	35.0(15)	-7.1(11)	-3.8(13)	7.8(12)
C00Y	18.8(17)	53(2)	86(3)	-38(2)	-4.2(17)	4.6(15)
C00Z	90(3)	67(2)	29.2(16)	-2.0(15)	-10.8(19)	53(2)

Table S25 Bond Lengths for 9aa.

**Atom**	**Atom**	**Length/Å**	**Atom**	**Atom**	**Length/Å**
S001	O003	1.4391(19)	C00C	C00M	1.537(3)
S001	O006	1.436(2)	C00D	C00E	1.393(4)
S001	C00E	1.762(3)	C00D	C00P	1.387(4)
S001	C00I	1.812(2)	C00E	C00J	1.388(3)
O002	C009	1.339(3)	C00F	C00L	1.384(4)
O002	C00U	1.480(3)	C00F	C00N	1.378(4)
O004	C008	1.222(3)	C00G	C00L	1.384(4)
O005	C009	1.207(3)	C00J	C00W	1.383(4)
N007	C008	1.352(3)	C00K	C00S	1.393(4)
N007	C00A	1.437(3)	C00N	C00R	1.394(3)
N007	C00H	1.470(3)	C00O	C00Q	1.387(4)
C008	C00I	1.529(4)	C00P	C00V	1.383(4)
C009	C00M	1.524(4)	C00Q	C00T	1.386(5)
C00A	C00G	1.392(3)	C00S	C00T	1.378(4)
C00A	C00R	1.385(3)	C00U	C00X	1.508(4)
C00B	C00K	1.387(4)	C00U	C00Y	1.510(5)
C00B	C00M	1.521(3)	C00U	C00Z	1.524(4)
C00B	C00O	1.391(4)	C00V	C00W	1.375(5)
C00C	C00I	1.536(3)			

Table S26 Bond Angles for 9aa.

**Atom**	**Atom**	**Atom**	**Angle/˚**	**Atom**	**Atom**	**Atom**	**Angle/˚**
O003	S001	C00E	108.44(12)	C00N	C00F	C00L	120.2(2)
O003	S001	C00I	109.16(11)	C00L	C00G	C00A	120.4(3)
O006	S001	O003	118.86(12)	C008	C00I	S001	111.77(17)
O006	S001	C00E	108.16(12)	C008	C00I	C00C	110.3(2)
O006	S001	C00I	106.30(12)	C00C	C00I	S001	106.83(16)

Table S26 Bond Angles for 9aa.

Atom	Atom	Atom	Angle/˚	Atom	Atom	Atom	Angle/˚
C00E	S001	C00I	105.08(11)	C00W	C00J	C00E	118.6(3)
C009	O002	C00U	120.6(2)	C00B	C00K	C00S	120.6(3)
C008	N007	C00A	127.7(2)	C00G	C00L	C00F	119.7(3)
C008	N007	C00H	116.8(2)	C009	C00M	C00C	109.8(2)
C00A	N007	C00H	115.4(2)	C00B	C00M	C009	109.5(2)
O004	C008	N007	121.7(2)	C00B	C00M	C00C	111.04(19)
O004	C008	C00I	119.5(2)	C00F	C00N	C00R	120.6(3)
N007	C008	C00I	118.7(2)	C00Q	C00O	C00B	120.7(3)
O002	C009	C00M	110.9(2)	C00V	C00P	C00D	120.4(3)
O005	C009	O002	124.9(2)	C00T	C00Q	C00O	120.3(3)
O005	C009	C00M	124.3(2)	C00A	C00R	C00N	119.3(2)
C00G	C00A	N007	117.8(2)	C00T	C00S	C00K	120.5(3)
C00R	C00A	N007	121.9(2)	C00S	C00T	C00Q	119.3(3)
C00R	C00A	C00G	119.9(2)	O002	C00U	C00X	103.0(2)
C00K	C00B	C00M	120.5(2)	O002	C00U	C00Y	109.3(2)
C00K	C00B	C00O	118.6(2)	O002	C00U	C00Z	109.1(2)
C00O	C00B	C00M	120.9(2)	C00X	C00U	C00Y	110.4(3)
C00I	C00C	C00M	112.69(19)	C00X	C00U	C00Z	108.8(3)
C00P	C00D	C00E	118.5(2)	C00Y	C00U	C00Z	115.5(3)
C00D	C00E	S001	119.2(2)	C00W	C00V	C00P	120.3(3)
C00J	C00E	S001	119.3(2)	C00V	C00W	C00J	120.7(3)
C00J	C00E	C00D	121.5(2)				

Table S27 Torsion Angles for 9aa.

A	B	C	D	Angle/˚	A	B	C	D	Angle/˚
S001	C00E	C00J	C00W	-178.67(19)	C00E	C00D	C00P	C00V	-1.1(4)
O002	C009	C00M	C00B	68.1(3)	C00E	C00J	C00W	C00V	-1.4(4)
O002	C009	C00M	C00C	-169.7(2)	C00F	C00N	C00R	C00A	-1.0(4)
O003	S001	C00E	C00D	-30.4(2)	C00G	C00A	C00R	C00N	0.2(4)
O003	S001	C00E	C00J	148.47(19)	C00H	N007	C008	O004	-3.4(3)
O003	S001	C00I	C008	62.43(19)	C00H	N007	C008	C00I	174.8(2)
O003	S001	C00I	C00C	-58.3(2)	C00H	N007	C00A	C00G	64.9(3)
O004	C008	C00I	S001	-71.9(3)	C00H	N007	C00A	C00R	-107.8(3)

Table S27 Torsion Angles for 9aa.

A	B	C	D	Angle/°	A	B	C	D	Angle/°
O004	C008	C00I	C00C	46.8(3)	C00I	S001	C00E	C00D	86.2(2)
O005	C009	C00M	C00B	-111.4(3)	C00I	S001	C00E	C00J	-94.9(2)
O005	C009	C00M	C00C	10.8(4)	C00I	C00C	C00M	C009	75.1(3)
O006	S001	C00E	C00D	-160.59(19)	C00I	C00C	C00M	C00B	-163.7(2)
O006	S001	C00E	C00J	18.3(2)	C00K	C00B	C00M	C009	-120.4(3)
O006	S001	C00I	C008	-168.22(17)	C00K	C00B	C00M	C00C	118.2(3)
O006	S001	C00I	C00C	71.07(19)	C00K	C00B	C00O	C00Q	0.0(4)
N007	C008	C00I	S001	109.8(2)	C00K	C00S	C00T	C00Q	0.0(4)
N007	C008	C00I	C00C	-131.5(2)	C00L	C00F	C00N	C00R	0.9(4)
N007	C00A	C00G	C00L	-172.0(2)	C00M	C00B	C00K	C00S	-178.9(2)
N007	C00A	C00R	C00N	172.7(2)	C00M	C00B	C00O	C00Q	178.5(3)
C008	N007	C00A	C00G	-113.2(3)	C00M	C00C	C00I	S001	-161.38(18)
C008	N007	C00A	C00R	74.1(3)	C00M	C00C	C00I	C008	77.0(3)
C009	O002	C00U	C00X	175.9(2)	C00N	C00F	C00L	C00G	0.1(4)
C009	O002	C00U	C00Y	58.5(3)	C00O	C00B	C00K	C00S	-0.4(4)
C009	O002	C00U	C00Z	-68.7(3)	C00O	C00B	C00M	C009	61.1(3)
C00A	N007	C008	O004	174.6(2)	C00O	C00B	C00M	C00C	-60.3(3)
C00A	N007	C008	C00I	-7.1(4)	C00O	C00Q	C00T	C00S	-0.4(4)
C00A	C00G	C00L	C00F	-1.0(4)	C00P	C00D	C00E	S001	179.90(19)
C00B	C00K	C00S	C00T	0.4(4)	C00P	C00D	C00E	C00J	1.0(4)
C00B	C00O	C00Q	C00T	0.4(4)	C00P	C00V	C00W	C00J	1.3(4)
C00D	C00E	C00J	C00W	0.2(4)	C00R	C00A	C00G	C00L	0.8(4)
C00D	C00P	C00V	C00W	0.0(4)	C00U	O002	C009	O005	7.0(4)
C00E	S001	C00I	C008	-53.70(19)	C00U	O002	C009	C00M	-172.5(2)
C00E	S001	C00I	C00C	-174.41(17)					

Table S28 Hydrogen Atom Coordinates (Å×10^4) and Isotropic Displacement Parameters (Å^2×10^3) for 9aa.

Atom	*x*	*y*	*z*	U(eq)
H00A	4070	1280	7981	23
H00B	2798	1319	7576	23
H00D	6583	621	6103	26
H00F	4218	6001	4984	31
H00G	6509	3536	5514	28

Table S28 Hydrogen Atom Coordinates (Å×10^4) and Isotropic Displacement Parameters (Å^2×10^3) for 9aa.

**Atom**	***x***	***y***	***z***	**U(eq)**
H00C	7763	3197	6761	39
H00E	7295	3463	7549	39
H00H	7280	4407	6952	39
H00I	3766	2660	6749	21
H00J	3572	1691	5065	30
H00K	4197	3411	9339	30
H00L	5806	4817	4684	33
H00M	3876	3311	8110	22
H00N	3346	5909	6107	30
H00O	1466	1462	8658	34
H00P	7744	1053	5097	35
H00Q	1039	976	9831	40
H00R	4082	4661	6958	25
H00S	3749	2937	10514	39
H00T	2174	1720	10765	41
H00V	6820	1764	4082	38
H00W	4749	2050	4058	37
H00U	1641	6364	8248	46
H00X	863	5736	8842	46
H00Y	198	6466	8249	46
H00Z	-1034	4766	8004	79
H	-246	4025	8536	79
HA	-376	3674	7718	79
H00	814	4708	6779	93
HB	1678	5702	7016	93
HC	245	5878	6991	93

Experimental

Single crystals of $C_{28}H_{31}NO_5S$ (**9aa**) were obtained by recrystallization from hexane/methanol. A suitable crystal was selected and mounted on a **Bruker D8 goniometer** diffractometer. The crystal was kept at 100.15 K during data collection. Using Olex2 [1], the structure was solved with the olex2.solve [2] structure solution program using Charge Flipping and refined with the SHELXL [3] refinement package using Least Squares minimisation.

1. Dolomanov, O.V., Bourhis, L.J., Gildea, R.J, Howard, J.A.K. & Puschmann, H. (2009), J. Appl. Cryst. 42, 339-341.

2. Bourhis, L.J., Dolomanov, O.V., Gildea, R.J., Howard, J.A.K., Puschmann, H. (2015). Acta Cryst. A71, 59-75.
3. Sheldrick, G.M. (2015). Acta Cryst. C71, 3-8.

Crystal structure determination of 9aa

Crystal Data for $C_{28}H_{31}NO_5S$ (M =493.60 g/mol): orthorhombic, space group $P2_12_12_1$ (no. 19), a = 11.0610(4) Å, b = 12.1022(3) Å, c = 18.7914(6) Å, V = 2515.46(14) Å^3, Z = 4, T = 100.15 K, μ (MoK α) = 0.168 mm^{-1}, $Dcalc$ = 1.303 g/cm^3, 37895 reflections measured (4.272° ≤ 2Θ ≤ 54.966°), 5691 unique (R_{int} = 0.0782, R_{sigma} = 0.0492) which were used in all calculations. The final R_1 was 0.0397 (I > 2 σ (I)) and wR_2 was 0.0898 (all data).

Refinement model description

Number of restraints - 432, number of constraints - unknown.

Details:

1. Fixed Uiso

At 1.2 times of:

All C(H) groups, All C(H,H) groups

At 1.5 times of:

All C(H,H,H) groups

2. Uiso/Uaniso restraints and constraints

All non-hydrogen atoms have similar U: within 2A with sigma of 0.005 and sigma
for terminal atoms of 0.01 within 2A

All non-hydrogen atoms restrained to be isotropic: with sigma of 0.005 and
sigma for terminal atoms of 0.01

3.a Ternary CH refined with riding coordinates:

C00I(H00I), C00M(H00M)

3.b Secondary CH2 refined with riding coordinates:

C00C(H00A,H00B)

3.c Aromatic/amide H refined with riding coordinates:

C00D(H00D), C00F(H00F), C00G(H00G), C00J(H00J), C00K(H00K), C00L(H00L),
C00N(H00N), C00O(H00O), C00P(H00P), C00Q(H00Q), C00R(H00R), C00S(H00S),
C00T(H00T), C00V(H00V), C00W(H00W)

3.d Idealised Me refined as rotating group:

C00H(H00C,H00E,H00H), C00X(H00U,H00X,H00Y), C00Y(H00Z,H,HA), C00Z(H00,HB,HC)
